# Design, Bioactivity and structure-activity of 3-Arylpropionate Derivatives as Potential High-Efficient Acaricides against *Psoroptes Cuniculi*

**DOI:** 10.1038/s41598-018-20140-7

**Published:** 2018-01-29

**Authors:** Dongdong Chen, Ye Tian, Mingxuan Xu, Xinyuan Wang, Ding Li, Fang Miao, Xinjuan Yang, Le Zhou

**Affiliations:** 1 College of Chemistry & Pharmacy, Northwest A&F University, Yangling, 712100 Shaanxi Province People’s Republic of China; 2 College of Life Science, Northwest A&F University, Yangling, Shaanxi People’s Republic of China; 3 Zhengzhou Railway Vocational and Technical College, Zhengzhou, Henan People’s Republic of China

## Abstract

A series of 3-aryl propionic esters and their analogues were designed and evaluated for acaricidal activity *in vitro* against *Psoroptes cuniculi*, a mange mite. The structure–activity relationship (SAR) was also discussed. The results showed that 6 compounds possessed the excellent activity (LC_50_ = 0.17–0.24 mM, LT_50_ = 1.5–2.9 h), superior to ivermectin (LC_50_ = 0.28 mM, LT_50_ = 8.9 h) (*P* < 0.05), a standard drug. Furthermore, 7 compounds showed the good activity (LC_50_ = 0.25–0.37 mM, LT_50_ < 3.9 h), slightly lower or close to that of ivermectin. One compound displayed super-fast acaricidal property, far superior to ivermectin. SAR analysis found that the ester group is vital for the activity and the small steric hindrance adjacent to the ester group is advantageous for the high activity. The <C4 linear alcohol esters can give the higher activity. The substituents on the 3-phenyl ring or replacement of the 3-phenyl with heterocyclic aryl generally decreases the activity. The position of the ester group in the ester chain also influences the activity, where the 3-phenyl propionate and the benzoate had the highest and lowest activity, respectively. Thus, 3-arylpropionates emerged as new and promising high-efficient acaricide candidates.

## Introduction

Acariasis is a skin disease caused by mites, which are widely parasitic on the body surfaces or epidermises of animals or human. *Psoroptes cuniculi* is an animal ear mite living in the ear canal of animals, and can infect rabbits, goats, horses, buffalo, sheep and so on^1^. More importantly, *Psoroptic* acariasis is a highly contagious disease. The infestation can cause intense pruritus, inflammation, serous exudations, the formation of crusts and scabs, reduction of weight gain, and even death^2^. Therefore, psoroptic acariasis has been a global disease causing serious economic losses for the animal industry^3^.

Therapy and control of both human scabies and animal mange mainly depend on the use of drugs and chemicals^4^. Among them, organochlorine, organophosphates, pyrethrins^5^, ivermectin and abamectin^6^ have been more commonly used drugs. However, continuous use of these drugs has presented some serious problems such as drug-resistance^7,8^, toxicity and environmental damage^9^. Therefore, it is urgent to develop new effective and safe acaricidal agents for treatment and control of animal acariasis.

Cinnamic acids are a class of natural phenylpropanoid compounds widely distributed in plants in the form of free acid and ester^10^. In the past decades, cinnamic acid derivatives have been attracting much attention due to their common occurrence in plants^10^, low toxicity^11^ and various important biological activities^12^, such as anticancer^13^, antimicrobial^14^, antioxidative^15,16^, anti-inflammatory^17^, anti-Mycobactrium tuberculosis^18^, anti-HIV^19^, antidiabetic^20^, anticholesterolemic^21^, hepatoprotective^22^, inducing neural progenitor cell proliferation^23^ and leishmanicidal activities^24^. In addition, we recently also found cinnamic acid esters had excellent acarcidal activity against *P. cuniculi*^25,26^ and strong inhibition activity against plant pathogenic fungi^27^. Thus, cinnamic acid is one promising molecular scaffold for the development of new drugs.

As one dihydrogen derivative of cinnamic acid, 3-phenylpropanoic acid is also one natural compound^28^. Its esters extensively exist in some plants^29,30^, rice wine^31^ and propolis^32^ as odor-active components. However, unlike cinnamic acid esters, few of study has been found on the bioactivity of 3-phenylpropanoic acid esters until now.

Especially interesting for us is that as the derivate of ethyl cinnamate, ethyl 3-phenylpropanoate showed greater acaricidal potential than ethyl cinnamate^25^. This result strongly suggests that it is necessary to take a systematical investigate on acaricidal activity of 3-phenylpropanoic acid esters in order to discover more potent acaricidal agents. As our continuing study, herein, a series of 3-aryl propionic acid esters and their analogues were reported for design, syntheses, acaricidal activity against *P. cuniculi* and preliminary structure-activity relationship (SAR).

## Results and Discussion

### Design of compounds

In order to explore SAR and find more potent compounds, based on the principle of structural similarity and the consideration of molecular diversity, four series of the target compounds were designed by using ethyl 3-phenylpropinate (**2**) as a lead compound. The first series is a group of 3-phenylpropionic acid esters (**1**–**31**) derived from various alcohols or phenols to explore the effect of various alkyl groups of the alcoholic moiety on the activity. The second series is a group of methyl 3-arylpropanoates (**32**–**42**) with various substituents on the benzene ring in order to explore the effect of the substitution pattern of the benzene ring on the activity. The substituents include electron-donating groups like Me, OMe and OH and electron-withdrawing groups like halogen atoms and NO_2_. The substitution position involves the *o*-, *m*- or *p*-site. The third series is a class of heteroaryl analogues (**43**–**49**) of ethyl 3-phenylpropionate in order to know the effect of different aromatic rings on the activity. The last group is comprised of the other compounds (**50**–**58**) with the aim of exploring the effect of the ester group itself and its location on the ester chain on the activity. The structures of the compounds are showed in Table 1. Since the bio-target and action mechanism of 3-phenylpropionate are yet unknown, we aimed mainly to gain insights into structural motifs that influence the biological activity of 3-phenylpropionic acid ester.

**Table 1:** Structures and preliminary acaricidal activity of the compounds against *P. cuniculi*. ^a^The differences between data with the different lowercases within a column are significant (*P* < 0.05).

**1–31**		**Mortality % (mean ± S.D.)**		**32–41**		**Mortality % (mean ± S.D.)**	
**No**.	**R**	**0.5 mg/mL**	**0.25 mg/mL**	**No**.	**R**	**0.5 mg/mL**	**0.25 mg/mL**
**1**	CH_3_	100.0 ± 0.0a	100.0 ± 0.0a	**32**	*p*-Me	100.0 ± 0.0a	98.0 ± 4.5a
**2**	CH_2_CH_3_	100.0 ± 0.0a	100.0 ± 0.0a	**33**	*p*-OH	54.0 ± 5.5i	30.0 ± 7.1mnop
**3**	CH_2_CH_2_CH_3_	100.0 ± 0.0a	100.0 ± 0.0a	**34**	*p*-F	76.0 ± 5.5de	50.0 ± 7.1gh
**4**	CH_2_(CH_2_)_2_CH_3_	100.0 ± 0.0a	98.3 ± 4.1a	**35**	*p*-Cl	78.0 ±4.5 cd	48.0 ± 4.5ghi
**5**	CH_2_(CH_2_)_3_CH_3_	100.0 ± 0.0a	98.3 ± 4.1a	**36**	*p*-Br	74.0 ±5.5de	48.0 ± 8.4ghi
**6**	CH_2_(CH_2_)_4_CH_3_	91.7 ± 4.1b	75.0 ± 5.5c	**37**	*o*-OMe	100.0 ± 0.0a	96.0 ± 5.5ab
**7**	CH_2_(CH_2_)_5_CH_3_	98.0 ± 4.5ab	48.0 ± 4.5ghi	**38**	*m*-OMe	100.0 ± 0.0a	96.0 ± 5.5ab
**8**	CH_2_(CH_2_)_6_CH_3_	46.0 ± 5.5jk	24.0 ± 5.5pqr	**39**	*p*-OMe	100.0 ± 0.0a	98.0 ± 4.5a
**9**	CH_2_(CH_2_)_7_CH_3_	32.0 ± 4.5mn	12.0 ± 4.5 u	**40**	*o*-NO_2_	100.0 ± 0.0a	100.0 ± 0.0a
**10**		100.0 ± 0.0a	100.0 ± 0.0a	**41**	*m*-NO_2_	100.0 ± 0.0a	100.0 ± 0.0a
**11**		100.0 ± 0.0a	100.0 ± 0.0a	**42**	*p*-NO_2_	100.0 ± 0.0a	100.0 ± 0.0a
**12**		42.0 ± 8.4kl	10.0 ± 7.1u	**42–48** (R = heterocyclic aryl)			
**13**		30.0 ± 7.1no	10.0 ± 7.1u				
**14**		80.0 ± 10.0cd	42.0 ± 4.5ijk	**43**		100.0 ± 0.0a	98.0 ± 4.5a
**15**		58.0 ± 4.5hi	36.0 ± 5.5klm	**44**		80.0 ± 7.1cd	54.0 ± 5.5fg
**16**		66.0 ± 5.5fg	48.0 ± 4.5ghi	**45**		100.0 ± 0.0a	58.0 ± 5.5ef
**17**		70.0 ± 7.1ef	52.0 ± 8.4fg	**46**		100.0 ± 0.0a	100.0 ± 0.0a
**18**		84.0 ± 5.5c	66.0 ± 5.5d	**47**		100.0 ± 0.0 a	100.0 ± 0.0 a
**19**		44.0 ± 5.5kl	26.0 ± 5.5opqr	**48**		100.0 ± 0.0a	90.0 ± 7.1b
**20**		46.0 ± 5.5jk	28.0 ±5.5nopq	**49**		78.0 ± 4.5cd	58.0 ± 4.5ef
**21**		54.0 ± 5.5i	34.0 ± 5.5lmn	**50**		45.0 ± 5.5jk	14.0 ± 5.5 tu
**22**		62.00 ± 4.5gh	38.0 ± 5.5jkl	**51**		100.0 ± 0.0a	100.0 ± 0.0a
**23**		76.0 ± 5.5de	62.0 ± 5.5de	**52**		100.0 ± 0.0a	100.0 ± 0.0a
**24**		96.0 ± 5.5ab	68.0 ± 4.5d	**53**		100.0 ± 0.0a	100.0 ± 0.0a
**25**		46.0 ± 5.5jk	22.0 ± 4.5qrs	**54**		46.7±5.2jk	16.7 ± 5.2stu
**26**		76.0 ± 5.5de	44.0 ±5.5hij	**55**		25.0±5.5o	15.0 ± 5.5stu
**27**		100.0 ± 0.0a	96.0 ± 5.5ab	**56**		66.0 ± 5.5fg	36.0 ± 5.5klm
**28**		52.0 ± 4.5ij	20.0 ± 7.1rst	**57**		42.0 ± 5.5kl	14.0 ± 5.5tu
**29**		38.0 ± 4.5lm	14.0 ± 5.5tu	**58**		35.0±5.5mn	10.0±6.3u
**30**		46.0 ± 5.5jk	24.0 ± 5.5pqr	Ivermectin		75.0 ± 5.5de	68.3 ± 7.5d
**31**		54.0 ± 5.5i	32.0 ±4.5lmno	Control		0.0 ± 0.0p	0.0 ± 0.0v

### Synthesis of compounds

**Figure 1:**
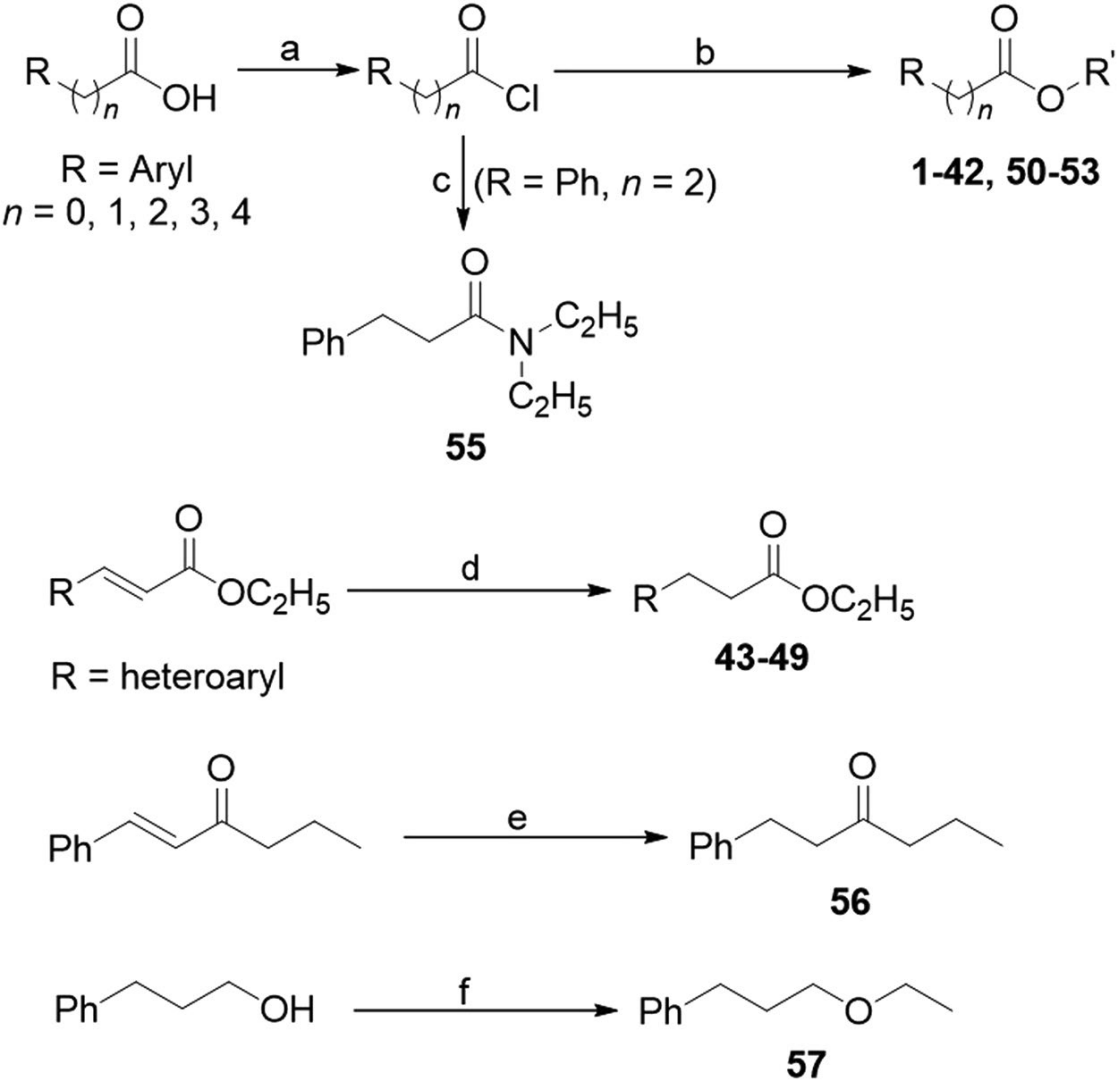
Synthesis of compounds **1**‒**53** and **55**‒**57**. Reagents and conditions. (**a**) SOCl_2_, 75 °C, 2 h; (**b**) R′OH, DCM, 0 °C, 1 h, 70–97% yield; (**c**) Et_2_NH/water, 0 °C, 1 h, 82% yield; (**d**) NaBH_4_-CuCl/EtOH, rt, 1 h, 63–90% yield; (**e**) NaBH_4_, Pd/C, CH_3_COOH, toluene, 0 °C, 2 h, 87% yield; (**f**) NaH, THF, 0 °C, 0.5 h; then CH_3_CH_2_I, 0 °C, 12 h, 53%.

As shown in Fig. 1, commercial available aryl fatty acids as starting materials were treated by thionyl chloride to provide the corresponding acyl chlorides. The latter reacted with the corresponding alcohols or phenols to obtain compounds **1**‒**42** and **50**‒**53** in 70–97% yields. 3-Phenylpropanoyl chloride reacted with diethylamine to afford compound **55** in 82% yield. Compounds **43**‒**49** were obtained by selective reduction of the carbon carbon double bond of the corresponding ethyl (*E*)-3-heteroarylpropionates with NaBH_4_ in the presence of CuCl in 63–90% yields. Compound **56** was prepared by selective reduction of the carbon carbon double bond of (*E*)-1-phenylhex-1-en-3-one with NaBH_4_ in the presence of Pd/C and CH_3_CO_2_H in 87% yield. Compound **57** was obtained by Williamson ether synthesis from 3-phenylpropan-1-ol and ethyl iodide in 53% yield.

The known compounds were confirmed by comparison of the NMR data and those reported in literature. The new compounds (**13**, **29** and **30**) were identified by ^1^H NMR, ^13^C NMR and HRMS analysis. The purity of all the compounds was determined to be >98% by ^1^H NMR analysis.

### Acaricidal activity

The synthesized compounds along with commercial available 3-phenylpropionic acid (**58**) and *N*-ethyl-3-phenylpropanamide (**54**) were screened for acaricidal activity *in vitro* against *P. cuniculi* according to our previous method^25,26^. Ivermectin, an acaricidal drug standard, was used as a reference control. The results listed in Table 1 showed that all the compounds showed some activity at 0.5 or 0.25 mg/mL. Among them, twenty-five compounds (**1**–**6**, **10**, **11**, **18**, **24**, **27**, **32**, **37**–**43**, **46**–**48**, **51**–**53**) displayed the good to excellent activity with mite mortality rates of >66% at 0.25 mg/mL, higher or equal to that of ivermectin (68.3%) (*p* ≤ 0.05), and twenty-one compounds (**1**–**5**, **10**, **11**, **27**, **32**, **37**–**43**, **46**, **47**, **51**–**53**) showed the mortality rates of ≥96%.

**Figure 2:**
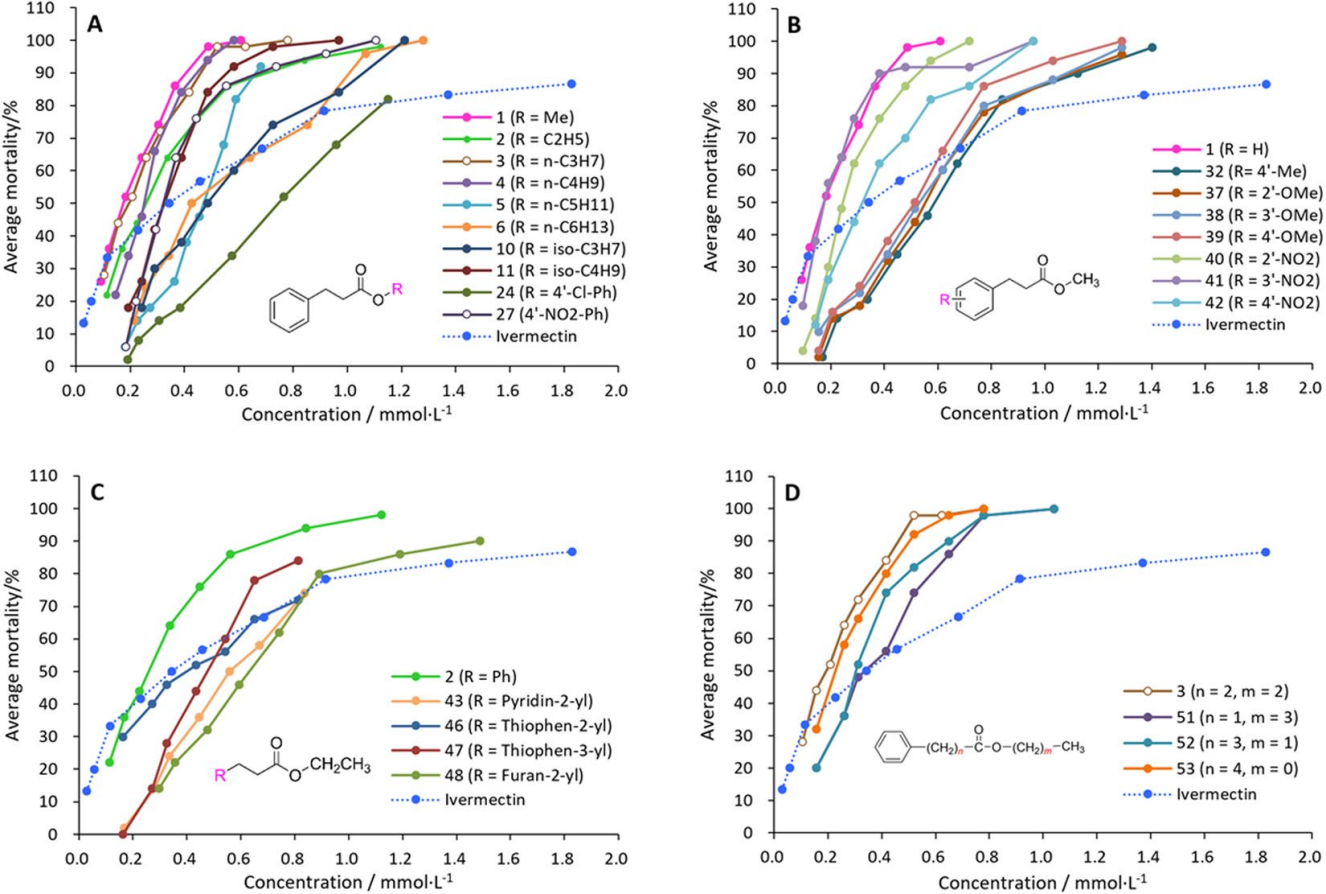
Effects of test concentrations of the compounds on the acaricidal activity against *P. cuniculi* (24 h).

**Figure 3:**
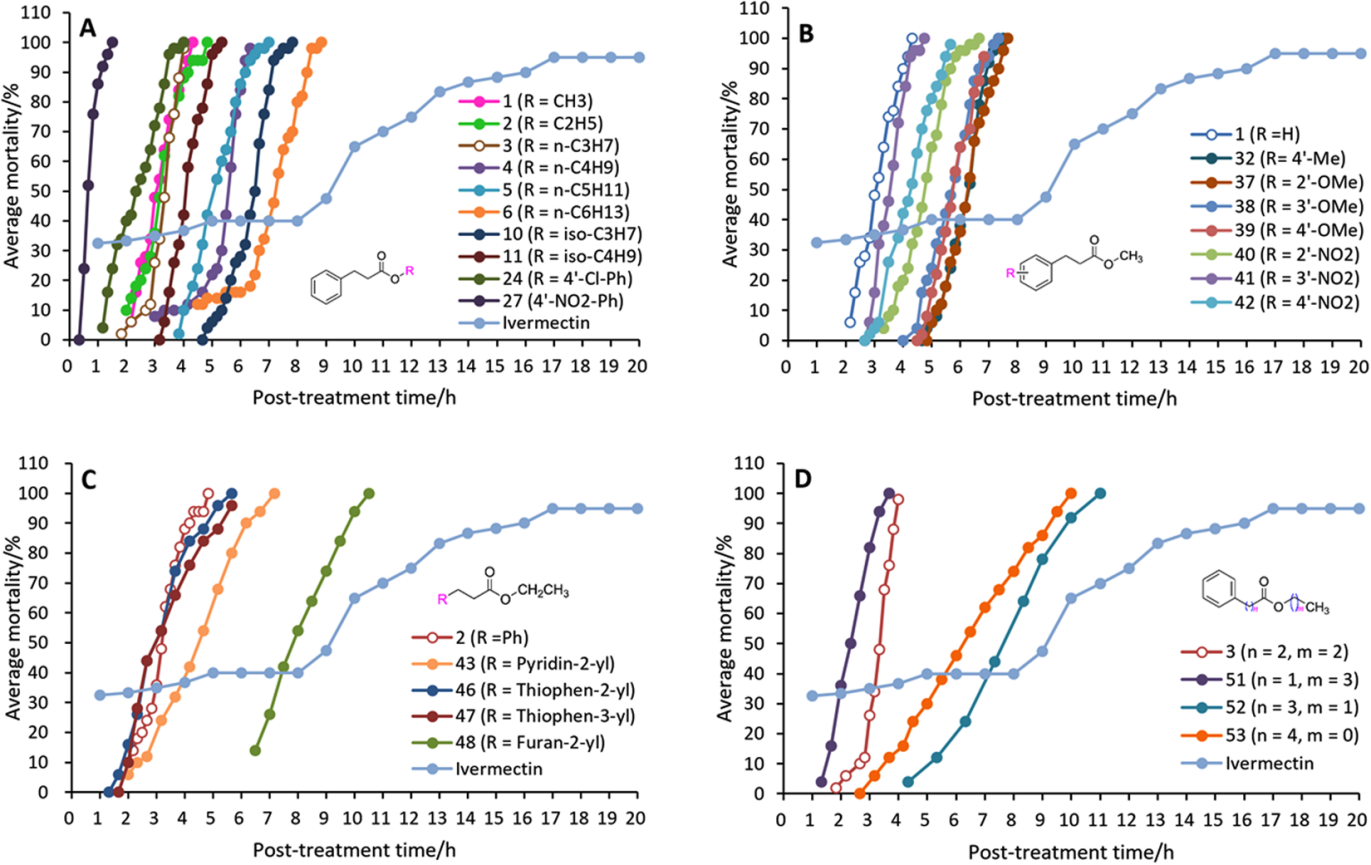
Effects of treatment times (**B**) of the compounds (4.5 mM) on the acaricidal activity against *P. cuniculi*. (a) compound **27** was tested at 2.0 mM (Fig. 3A). The other compounds were tested at 4.5 mM.

According to the same method described above, the compounds with the higher initial activity in Table 1 were further determined for median lethal concentrations (LC_50_) and median lethal times (LT_50_) in order to get insight into their acaricidal potency. Ivermectin was used as a reference drug. The mortality rates caused by treatment of various concentrations of the compounds for 24 h or various treatment times of each the compound at 4.5 mM are shown in Figs 2 and 3, respectively. Figures 2 and 3 showed that the activities of all the compounds were concentration- and time-independent, but the change trend (or steepness) of the various curves is different, indicating that the mites had different susceptibility to the concentration and treatment time of the various compounds. It was found from the steepness of the various curves that the mites were more sensitive to the compounds than ivermectin in two aspects of concentration effect and treatment-time effect. Meanwhile, most of the test compounds showed the higher activity in most of the test concentrations or treatment times than ivermectin (Figs 2 and 3). As far as concentration effect was concerned, compounds **1** showed the highest activity followed by compounds **3** and **41** (Fig. 2) while for treatment-time effect, compound **27** displayed the highest activity followed by **24**, **51**, **1**, **2**, **3**, **46** and **47** (Fig. 3). It was worth noting that the result of compound **27** in Fig. 3A was obtained at 2.0 mM but not 4.5 mM for the other compounds. Interestingly, two compounds **6** and **10**, **1** and **41** or **37** and **38** showed the very similar concentration-mortality curve (Fig. 2A,B). Similar cases were also observed for the time-mortality curves of both **1** and **2**, **32** and **37**, **38** and **39** or **46** and **47** (Fig. 3).

The results above were further confirmed by LC_50_ and LT_50_ values of the various compounds (Table 2). Seven compounds (**1**‒**4**, **41** and **53**) showed smaller LC_50_ values of 0.17‒0.25 mM than ivermectin (LC_50_ = 0.28 mM) (*P* < 0.05). Compounds **1**, **3** and **41** showed the highest activity (LC_50_ = ca. 0.17, *P* < 0.05), and their relative activities reach more than 1.5-fold of ivermectin. Compounds **2**, **4**, **40** and **53** showed the second highest activity (LC_50_ = ca. 0.24, *P* < 0.05). Additionally, compounds **11**, **27**, **42**, **51** and **52** also showed the good activity (LC_50_ 0.29‒0.32 mM), slightly lower or close to that of ivermectin. It is worth noting that the molecular weight of ivermectin (MW 875) is much higher than that of the test compounds (MWs 164–271). Therefore, when LC_50_ values are expressed in mass concentration, all the compounds possessed the smaller LC_50_ values of 27.3–184.0 *μ*g/mL than ivermectin (LC_50_ 247.4 *µ*g/mL) (Table 2). On the other hand, all the compounds in Table 2 also showed faster acaricidal action with LT_50_ values of <8.4 h than ivermectin (LT_50_ = 8.9 h) at the same concentration of 4.5 mM. Especially, compound **27** showed super-fast acaricidal action (LT_50_ < 0.7 h, at 2.0 mM), far superior to the other compounds or ivermectin. Even if at 1.0 mM, its relative activity (LT_50_ = 3.6 h) still reached up to 2.5-fold that of ivermectin at 4.5 mM.

**Table 2:** Median lethal concentration values (LC_50_) and median lethal time values (LT_50_) of the compounds (24 h). ^a^The mites were treated by the solution of the compounds for 24 h. ^b^Relative activity = LC_50_ of ivermectin/LC_50_ (mM) of the tested compound. ^c^The test concentration of the compounds was 4.5 mM. ^d^Relative activity = LT_50_ of ivermectin/LT_50_ of the tested compound. ^*^The test concentration of the compound was 2.0 mM. ^**^The test concentration of the compound was 1.0 mM.

Compound	LC_50_ (mM (*μ*g/mL))^a^	95% Confidence interval (mM)	RA^b^	LT_50_ (h)^c^	95% Confidence interval	RA^d^
**1**	0.17 a (27.3)	0.15–0.19	1.65	3.0 b	3.0–3.1	2.9
**2**	0.24 b (41.9)	0.23–0.25	1.17	3.2 c	3.1–3.2	2.8
**3**	0.18 a (35.1)	0.16–0.19	1.56	3.3 d	3.2–3.4	2.7
**4**	0.23 b (47.8)	0.20–0.26	1.22	5.5 h	5.5–5.6	1.6
**5**	0.46 h (100.2)	0.43–0.48	0.61	5.1 g	5.0–5.1	1.7
**6**	0.47 g (109.8)	0.43–0.51	0.60	7.2 k	7.2–7.2	1.2
**10**	0.50 i (96.4)	0.47–0.53	0.56	6.4 j	6.3–6.5	1.4
**11**	0.32 ef (65.1)	0.29–0.34	0.88	4.1 e	4.0–4.1	2.2
**24**	0.71 l (184.0)	0.64–0.77	0.39	2.3 a	2.2–2.4	3.9
**27**	0.32 f (87.6)	0.30–0.34	0.88	<0.7^*^		å 12.7
				3.6^**^	3.6–3.6	2.5
**32**	0.55 j (98.8)	0.51–0.58	0.51	6.1 i	6.1–6.2	1.5
**37**	0.53 j (102.5)	0.50–0.57	0.53	6.2 i	6.19–6.25	1.4
**38**	0.51 j (99.0)	0.47–0.54	0.55	5.6 h	5.5–5.7	1.6
**39**	0.47 i (92.1)	0.43–0.51	0.60	5.6 h	5.5–5.6	1.6
**40**	0.25 bc (52.7)	0.24–0.26	1.12	4.7 g	4.6–4.8	1.9
**41**	0.18 a (37.3)	0.16–0.19	1.56	3.5 d	3.4–3.6	2.5
**42**	0.31ef (65.2)	0.29–0.33	0.90	4.2 ef	4.2–4.3	2.1
**43**	0.56 j (100.5)	0.53–0.59	0.50	4.3 f	4.1–4.6	2.1
**46**	0.37 g (69.0)	0.34–0.41	0.76	2.9 bc	2.8–3.0	3.1
**47**	0.46 h (85.4)	0.43–0.49	0.61	3.1 c	2.9–3.2	2.9
**48**	0.60 k (101.3)	0.56–0.64	0.47	8.4 m	8.2–8.5	1.1
**51**	0.32 f (61.9)	0.28–0.36	0.88	2.3 a	2.2–2.3	3.9
**52**	0.29 de (55.8)	0.26–0.32	0.97	7.3 l	7.0–7.7	1.2
**53**	0.23 b (43.4)	0.20–0.26	1.22	6.1 i	5.8–6.2	1.5
Ivermectin	0.28 cd (247.4)	0.23–0.33	1.00	8.9 n	8.8–9.0	1.0

### Discussions—Structure-activity relationship

By comparison of the activity and structures of the various compounds in Tables 1 and 2 coupling with Figs 2 and 3, we found some clear and important SARs for 3-aryllpropoionic acid esters. (1) For the alcohol esters, the type and steric hindrance of the alcoholic alkyls (R) significantly influence the LC_50_ values of the compounds. Generally, the activity of the compounds decreases with elongation of the linear R (**1**–**9**, Tables 1 and 2). Compared with the ≥C5 alkyl groups (**5**–**9**, LC_50_>100 *µ*g/mL), the ≤C4 alkyl (**1**–**4**, <50 *µ*g/mL) can dramatically improve the activity. Compared with the secondary or tertiary alcohol esters, the primary alcohol esters can give the higher activity (**3** vs **10**; **4** vs **12**; **5** vs **13**). A similar case was also found for the iso-butyl ester (**11**) (a primary alcohol ester) and the iso-propyl ester (**10**) (a secondary alcohol ester) (Table 2) although the former possesses a larger spatial volum than the latter. The results above suggest that the steric hindrance around the *α*-C of the alcoholic moiety or near the ester group can significantly impact the activity. The small steric hindrance adjacent to the ester group is helpful for the high activity. The opinion above can be aslo supported by the fact that the *n*-hexyl ester (**6**) is more active than the cyclohexyl ester (**14**). Interestingly, the SARs above is very similar to that of cinnamic acid esters^26^.

On the other hand, the alcoholic alkyls also have the similar influence trend for the LT_50_ values of the compounds. The methyl, ethyl and *n*-propyl esters (**1**‒**3**) showed the very close-by LT_50_ values, superior to that of the *n*-butyl, *n*-amyl, *n*-hexyl esters (**4**‒**6**). The iso-butyl ester (**11**) also showed the smaller LT_50_ values than the iso-propyl ester (**10**). Interestingly, compounds **1**‒**3** displayed very similar time-effect curves in Fig. 3A.

2) The introduction of substituents to the 3-phenyl ring of the methyl propionate (**1**) leads to decrease of the activity (**32**‒**40**, **42** *vs* **1**) except 3ʹ-NO_2_ (**41**). A similar case was also observed for cinnamates^25^. Since the substituents not only include various groups with different electron effect like OH, OMe, Me, halogen atoms or NO_2_ but also involve hydrogen-bond acceptors (NO_2_, OMe) and hydrogen-bond donors (OH), we speculated that the effect of substituents on the 3-phenyl ring on the activity should be mainly steric effect but not electron effect or hydrogen-bond effect.

3) Compared with the *n*-hexyl ester (**6**) or the cyclohexyl ester (**14**), the phenyl ester (**15**) showed the lower mite mortality (Table 1). Some similarly, the benzyl ester (**28**) was less active than the *n*-heptyl ester (**7**). The results above suggest that the fatty or alicyclic alcohol esters have the higher activity than the phenol or aromatic alcohol esters. However, it is worth noting that for the phenolic esters, the substituents on the phenolic ring can significantly impact the activity (**15**–**31**). Compared with the phenyl ester (**15**), the introduction of methyl, chlorine atom or nitro to the phenyl ring leads to significant improvement of the activity (**16**–**18**, **22**–**24**, **26**, **27** *vs* **15**) (Table 1). A similar case was also found in cinnamic acid esters^26^. Remarkably, among the compounds in Table 2, the 4-nitrophenyl ester (**27**) and 4-chlorophenyl ester (**24**) gave the lowest and second lowest LT_50_ values, respectively. The results above showed that the presence of *p*-cholor or *p*-nitro on the phenolic ring can endow the phenolic esters with the fast-acting property to the mites.

4) Replacement of the phenyl of ethyl 3-phenylpropionate (**2**) by heterocyclic aryls like pyridyl, thienyl or furyl leads to slight decrease of the activity (**43**–**49** *vs* **2**). This result is similar but completely the same as that of the heterocyclic analogues of cinnamates, in which the pyridin-2-yl or furan-2-yl analogues of ethyl cinnamate were more active than ethyl cinnamate^26^.

5) Compounds **3** and **50**–**53** have the same ester chain length consisting of seven atoms. Their structural difference only lies in the position of the ester group on the chain. From the activities of these compounds, it was found that the position of the ester group on the ester chain is able to influence the activity also. Among them, the propionate (**3**) has the highest activity although the acetate, butyrate and pentanoate isomers (**51**–**53**) also showed the good activity. Unusually, the benzoate (**50**) showed the much lower activity than the other isomers (**3**, **51**–**53**). This result is probably attributed to the π–π conjugation effect existing between the ester carbonyl group and the benzene ring in **50**. Similarly, the lower activity of ethyl cinnamate than its dihydro-derivate (**2**) may be also related with the same reason^25^.

6) Compounds **54**–**57** may be considered as analogues or derivatives of ethyl 3-phenypropionate (**2)**. These compounds have the same chain length consisting of six atoms. Obviously, the ester (**2**) were much more active than its corresponding amides (**54**, **55**), ketone (**56**), ether (**57**) or acid (**58**) (Table 1). These results show that the ester group is vital to the activity. On the other hand, the ketone **56** being more active than its corresponding ether **57** suggests that the carbonyl of the ester group should be more important than its saturated oxygen for the activity. The results above are the same as that of cinnamates^26^. Based on the results above, we conjectured that the activity of the esters very likely involves an acyl transfer mechanism, in which the oxygen atom of the ester carbonyl probably acts as a hydrogen bond receptor or nucleophile when the compounds interact with their biological receptor. If so, the lower electron density on the carbonyl carbon should be helpful for the activity. The opinion above can be supported by the fact that the ester (**2**) was more active than the amides (**54**, **55**) to some extent. At present, the study on the acaricidal bio-target and mechanism of these esters has been in our plan.

In conclusion, a series of 3-arylpropionate derivatives were designed and evaluated for acaricidal activity *in vitro* against *Psoroptes cuniculi*. Twenty-four compounds were found to possess the lower LT_50_ values than ivermectin, a standard drug, and of which seven compounds also showed the higher LC_50_ activity at the same time than ivermectin. Besides having the excellent LC_50_ activity, compound **27** also showed a super-fast acaricidal property, far superior to ivermection. Compared with ivermectin, the present compounds displayed some obviously attractive advantages, such as natural compounds or natural compound framework, the higher activity, the quick-acting property, simple structure, easy preparation and low-cost. Thus, these compounds possess great potential for development of novel acaricidal agents. Additionally, some important SARs were derived. The ester group was found to be crucial for the activity. The type of the alcoholic alkyls and its steric hindrance around the alcoholic *α*-C or near the carbonyl group are two main influence factors on the activity. The fatty alcohol esters were generally more active than the phenol esters or aromatic alcohol esters. The distance between the ester group and the phenyl ring slightly affects the activity except the benzoate with the lower activity. The introduction of substituents to the 3-phenyl ring or replacement of the 3-phenyl by heterocyclic aryls leads to decrease of the activity. Further research is very necessary on the acaricidal mechanism, therapeutic effect and toxicity of the compounds. At present, these works are partly underway in our lab.

## Methods

### Chemicals

3-Phenylpropanoic acid (**58**), *N*-ethyl-3-phenylpropanamide (**54**) and ivermectin (≥91% 22,23-dihydroavermectin B1 consisting of 95% avermectin B1a and 5% avermectin B1b) was purchased from Sigma-Aldrich Trading Co., Ltd., Shanghai, China. Other chemicals used in the present study were purchased from J&K Chemical Ltd., Beijing, China, and used without further purification.

### Instruments

Melting points (m.p.) were determined on an XT-4 micro-melting point apparatus and uncorrected. ^1^H- and ^13^C-NMR spectra were recorded on a Bruker AVANCE III (Bruker, Karlsruhe, Germany) operating at 500 and 125 MHz, respectively and using tetramethylsilane (TMS) as an internal standard. Chemical shifts (*δ* values) and coupling constants (*J* values) are given in ppm and Hz, respectively. Mass spectra (HR-MS) were carried out with a Micromass Auto Spec-3000 instrument.

### Synthesis

#### Synthesis of compounds **1**–**42** and **50**–**53**

The general procedure is as follows. The mixture of aryl- or heteroaryl-substituted fatty acid (10 mmol) and thionyl chloride (41 mmol, 3 mL) was refluxed for 2 h. The excess thionyl chloride was removed under reduced pressure to provide the corresponding acyl chloride. The acyl chloride without further purification was dissolved in 6 mL dry DCM, and cooled to 0 °C. The corresponding alcohol or phenol (10 mmol) was added, and stirred at 0 °C for 1 h. The mixture was diluted by DCM (50 mL), successively washed with water and 5% Na_2_CO_3_ solution, and dried over anhydrous sodium sulfate. After removal of the solvent under reduced pressure, the residue was subjected to silica gel column chromatography using petroleum ether–ethyl acetate (10:1) to afford the desired compounds.

The following known compounds were obtained as a colorless oil in 70–97% yield: methyl 3-phenylpropanoate (**1**), ethyl 3-phenylpropanoate (**2**), propyl 3-phenylpropanoate (**3**), butyl 3-phenylpropanoate (**4**), pentyl 3-phenylpropanoate (**5**), hexyl 3-phenylpropanoate (**6**), heptyl 3-phenylpropanoate (**7**), octyl 3-phenylpropanoate (**8**), nonyl 3-phenylpropanoate (**9**), *iso*-propyl 3-phenylpropanoate (**10**), *iso*-butyl 3-phenylpropanoate (**11**), *t*-butyl 3-phenylpropanoate (**12**), cyclohexyl 3-phenylpropanoate (**14**), phenyl 3-phenylpropanoate (**15**), *p*-tolyl 3-phenylpropanoate (**18**), *m*-methoxyphenyl 3-phenylpropanoate (**20**), *o*-chlorophenyl 3-phenylpropanoate (**22**), benzyl 3-phenylpropanoate (**28**), methyl 3-(4-fluoromophenyl)propanoate (**34**), methyl 3-(4-chlorophenyl)propanoate (**35**), methyl 3-(4-bromophenyl)propanoate (**36**), 3-(2-methoxyphenyl)propanoate (**37**), 3-(3-methoxyphenyl)propanoate (**38**), pentyl benzoate (**50**), butyl 2-phenylacetate (**51**), ethyl 4-phenylbutanoate (**52**), methyl 5-phenylpentanoate (**53**). The known compounds were obtained as a yellow or pale yellow oil in 72%–85%: *m*-tolyl 3-phenylpropanoate (**17**), *p*-methoxyphenyl 4-phenylbutanoate (**21**), *p*-nitrobenzyl 3-phenylpropanoate (**31**) and methyl 3-(4-methoxyphenyl)propanoate (**39**).

Known compounds *p*-chlorophenyl 4-phenylbutanoate (**24**) in 77% yield, m.p. 55–56 °C, methyl 3-(4-methylphenyl)propanoate (**32**) in 84% yield, m.p. 35–36 °C and methyl 3-(4-hydroxyphenyl)propanoate (**33**) in 51% yield, m.p. 39–40 °C were obtained as a white solid; known compound *p*-nitrophenyl 4-phenylbutanoate (**27**) in 83% yield, m.p. 96–97 °C, methyl 3-(2-nitrophenyl)propanoate (**40**) in 76% yield, m.p. 55–56 °C and methyl 3-(4-nitrophenyl)propanoate (**42**) in 80% yield, m.p. 73–74 °C were obtained as a yellow solid.

The ^1^H and/or ^13^C NMR data of the compounds above were showed in Supporting Information and consistent with those in literature.

#### *tert*-Pentyl 3-phenylpropanoate (**13**)

Yield: 70%; a colorless oil; ^1^H NMR (500 MHz, CDCl_3_) *δ*: 7.34–7.31 (m, 2 H, Ar-H), 7.26–7.22 (m, 3 H, Ar-H), 2.96 (t, *J* = 7.8 Hz, 2 H, H-3), 2.60 (t, *J* = 7.8 Hz, 2 H, H-2), 1.79 (q, *J* = 7.5 Hz, 2 H, CH_2_CH_3_), 1.44 (s, 6 H, C(CH_3_)_2_), 0.88 (t, *J* = 7.5 Hz, 3 H, CH_2_CH_3_); ^13^C NMR (125 MHz, CDCl_3_) *δ*: 172.2 (C=O), 140.8 (Ph, C-1′), 128.4 (C-3′, C-5′), 128.3 (C-2′, C-6′), 126.1 (C-4′), 82.8 (O-C), 37.1 (C-2), 33.5 (CH_2_CH_3_), 31.2(C-3), 25.6 (CH_3_ × 2), 8.2 (CH_2_CH_3_). HR-ESI-MS [M+Na]^+^ calcd for C_14_H_20_NaO_2_^+^, 243.1356, found 243.1351.

#### *o*-Tolyl 3-phenylpropanoate (**16**)

Yield: 71%; a yellow oil; ^1^H NMR (500 MHz, CDCl_3_) *δ*: 7.41–7.34 (m, 4 H, Ar-H), 7.32–7.24 (m, 3 H, Ar-H), 7.21–7.18 (m, 1 H, Ar-H), 7.00 (d, *J* = 7.7 Hz, 1 H, H-3″), 3.17 (t, *J* = 7.7 Hz, 2 H, H-3), 2.99 (t, *J* = 7.7 Hz, 2 H, H-2), 2.15 (s, 3 H, CH_3_); ^13^C NMR (125 MHz, CDCl_3_) *δ*: 171.2 (C=O), 149.4 (C-1″), 140.2 (C-1′), 131.2 (C-3″), 130.1 (C-2″), 128.7 (C-3′, C-5′), 128.5 (C-2′, C-6′), 126.9 (C-5″), 126.5 (C-4″), 126.1 (C-4′), 121.9 (C-6″), 35.8 (C-2), 31.1 (C-3), 16.1 (CH_3_).

#### 2-Methoxyphenyl 3-phenylpropanoate (**19**)

Yield: 80%; a yellow oil; ^1^H NMR (500 MHz, CDCl_3_) *δ*: 7.38–7.34 (m, 4 H, Ar-H), 7.31–7.25 (m, 2 H, Ar-H), 7.03–6.99 (m, 3 H, Ar-H), 3.85 (s, 3 H, CH_3_), 3.16 (t, *J* = 7.7 Hz, 2 H, H-3), 2.98 (t, *J* = 7.7 Hz, 2 H, H-2); ^13^C NMR (125 MHz, CDCl_3_) *δ*: 171.0 (C=O), 151.2 (C-2″), 140.4 (C-1″), 139.9 (C-1′), 128.6 (C-3′, C-5′), 128.5 (C-2′, C-6′), 126.9 (C-4″), 126.4 (C-4′), 122.8 (C-5″), 120.8 (C-6″), 112.5 (C-3″), 55.9 (OCH_3_), 35.6 (C-2), 31.0 (C-3).

#### 3-Chlorophenyl 3-phenylpropanoate (**23**)

Yield: 79%; a brown solid; m.p. 34–35 °C; ^1^H NMR (500 MHz, CDCl_3_) *δ*: 7.41–7.38 (m, 2 H, Ar-H), 7.36–7.30 (m, 4 H, Ar-H), 7.29–7.26 (m, 1 H, Ar-H), 7.11 (t, *J* = 1.9 Hz, 1 H, H-2″), 7.01–6.94 (m, 1 H, Ar-H), 3.14 (t, *J* = 7.6 Hz, 2 H, H-3), 2.95 (t, *J* = 7.7 Hz, 2 H, H-2); ^13^C NMR (125 MHz, CDCl_3_) *δ*: 171.0 (C=O), 151.2 (C-1″), 140.0 (C-1′), 134.7 (C-3″), 130.2 (C-5″), 128.7 (C-3′, C-5′), 128.5 (C-2′, C-6′), 126.6 (C-4′), 126.2 (C-4″), 122.3 (C-2″), 120.0 (C-6″), 36.0 (C-2), 30.9 (C-3).

#### 2-Nitrophenyl 3-phenylpropanoate (**25**)

Yield: 81%; a pale yellow solid; m.p. 74–75 °C; ^1^H NMR (500 MHz, CDCl_3_) *δ*: 8.14 (dd, *J* = 8.2, 1.5 Hz, 1 H, H-3″), 7.69 (t × 2, *J* = 7.9, 1.5 Hz, 1 H, H-4″), 7.44 (t × 2, *J* = 7.9, 1.2 Hz, 1 H, H-5″), 7.40–7.37 (m, 2 H), 7.34–7.29 (m, 3 H), 7.21 (d, *J* = 8.1 Hz, 1 H, H-6″), 3.16 (t, *J* = 7.8 Hz, 2 H, H-3), 3.04 (t, *J* = 7.8 Hz, 2 H, H-2); ^13^C NMR (125 MHz, CDCl_3_) *δ*: 170.6 (C=O), 144.1 (C-1″), 141.8 (C-2″), 139.9 (C-1′), 134.8 (C-5″), 128.7 (C-3′, C-5′), 128.4 (C-2′, C-6′), 126.7 (C-6″), 126.5 (C-4′), 125.8 (C-3″), 125.3 (C-4″), 35.7 (C-2), 30.5 (C-3).

#### 3-Nitrophenyl 3-phenylpropanoate (**26**)

Yield: 82%; a yellow solid; m.p. 67–68 °C; ^1^H NMR (500 MHz, CDCl_3_) *δ*: 8.16–8.14 (m, 1 H, H-4″), 7.95 (t, *J* = 2.2 Hz, 1 H, H-2″), 7.58 (t, *J* = 8.2 Hz, 1 H, H-6″), 7.42–7.38 (m, 3 H), 7.35–7.30 (m, 3 H), 3.15 (t, *J* = 7.8 Hz, 2 H, H-3), 3.00 (t, *J* = 7.8 Hz, 2 H, H-2); ^13^C NMR (125 MHz, CDCl_3_) *δ*: 170.8 (C=O), 151.0 (C-1″), 148.8 (C-3″), 139.7 (C-1′), 130.0 (C-5″), 128.7 (C-3′, C-5′), 128.4 (C-2′, C-6′), 128.1 (C-6″), 126.7 (C-4′), 120.8 (C-4″), 117.4 (C-2″), 35.9 (C-2), 30.9 (C-3).

#### 2-Nitrobenzyl 3-phenylpropanoate (**29**)

Yield: 80%; a brown oil; ^1^H NMR (500 MHz, CDCl_3_) *δ*: 8.14 (dd, *J* = 8.2, 1.0 Hz, 1 H, H-3″), 7.62 (t × 2, *J* = 7.7, 1.1 Hz, 1 H, H-5″), 7.51 (t × 2, *J* = 7.7, 1.1 Hz, 1 H, H-4″), 7.43 (d, *J* = 7.7 Hz, 1 H, H-6″), 7.36–7.33 (m, 2 H), 7.28–7.25 (m, 3 H), 5.19 (s, 2 H, OCH_2_), 3.05 (t, *J* = 7.8 Hz, 2 H, H-3), 2.81 (t, *J* = 7.8 Hz, 2 H, H-2); ^13^C NMR (125 MHz, CDCl_3_) *δ*: 172.4 (C=O), 147.7 (C-2″), 143.3 (C-1″, C-6″), 140.1 (C-1′), 128.6 (C-3′, C-5′), 128.34 (C-2′, C-6′), 128.31 (C-4″, C-5″), 126.5 (C-4′), 123.8 (C-3″), 64.8 (OCH_2_), 35.7 (C-2), 30.9 (C-3). HR-ESI-MS [M+Na]^+^ calcd for C_16_H_15_NNaO_4_^+^, 308.0893, found 308.0889.

#### 3-Nitrobenzyl 3-phenylpropanoate (**30**)

Yield: 78%; a pale yellow oil; ^1^H NMR (500 MHz, CDCl_3_) *δ*: 8.23–8.21 (m, 2 H, H-2″, H-4″), 7.64 (d, *J* = 7.7 Hz, 1 H, H-6″), 7.58–7.55 (m, 1 H, H-5″), 7.34–7.31 (m, 2 H), 7.26–7.23 (m, 3 H), 5.23 (s, 2 H, CH_2_), 3.03 (t, *J* = 7.7 Hz, 2 H, H-3), 2.78 (t, *J* = 7.7 Hz, 2 H, H-2); ^13^C NMR (125 MHz, CDCl_3_) *δ*: 172.5 (C=O), 148.4 (C-3″), 140.1 (C-1′), 138.1 (C-1″), 134.0 (C-6″), 129.6 (C-5″), 128.6 (C-3′, C-5′), 128.3 (C-2′, C-6′), 126.4 (C-4′), 123.2 (C-4″), 122.9 (C-2″), 64.8 (OCH_2_), 35.7 (C-2), 30.9 (C-3). HR-ESI-MS [M+Na]^+^ calcd for C_16_H_15_NNaO_4_^+^, 308.0893, found 308.0879.

#### Methyl 3-(3-nitrophenyl)propanoate (**41**)

Yellow solid in 78% yield, m.p. 45–46 °C. ^1^H NMR (500 MHz, CDCl_3_) *δ*: 8.12–8.11 (m, 2 H, H-2′, H-4′), 7.59 (d, *J* = 7.6 Hz, 1 H, H-6′), 7.50 (t, *J* = 8.3 Hz, 1 H, H-5′), 3.72 (s, 3 H, CH_3_), 3.11 (t, *J* = 7.6 Hz, 2 H, H-3), 2.72 (t, *J* = 7.6 Hz, 2 H, H-2); ^13^C NMR (125 MHz, CDCl_3_) *δ*: 172.6 (C=O), 148.4 (C-3′), 142.5 (C-1′), 134.7 (C-6′), 129.4 (C-5′), 123.3 (C-2′), 121.6 (C-4′), 51.8 (OCH_3_), 35.0 (C-2), 30.5 (C-3).

#### The general procedure for synthesis of compounds **43**–**49** is as follows

To the solution of ethyl (*E*)-3-heteroaryl acrylate (10 mmol) and CuCl (6 mmol) in DCM (50 mL) was slowly added NaBH_4_ (25 mmol) at room temperature within 20 minutes. After stirring for 0.5 h, the solution was concentrated under reduced pressure. The residue was purified by silica gel column chromatography using petroleum ether–ethyl acetate (3:1 or 10:1) to afford the desired compounds. Compounds **43**–**49** were obtained as a yellow or pale yellow oil in 63–90%. The ^1^H and/or ^13^C NMR data of the compounds were consistent with those in literature.

#### Synthesis of *N*,*N*-diethyl-3-phenylpropanamide (**55**)

According to the method described above for **1**–**42**, 3-phenylpropanoyl chloride was obtained by reaction of 3-phenyl propionic acid (10 mmol) and thionyl chloride (41 mmol, 3 mL). The acyl chloride was slowly added to the solution of diethylamine (25 mmol) in DCM (50 mL) at 0 °C within 10 minutes. The mixture was stirred at 0 °C for 1 h, and warmed to room temperature. The solution was washed with water, dried over anhydrous sodium sulfate and concentrated. The residue was subjected to column chromatography over silica gel eluting with the mixture of petroleum ether and ethyl acetate (2:1, V:V) to afford *N*,*N*-diethyl-3-phenylpropanamide (**55**) as a yellow oil in 82% yield. The ^1^H and/or ^13^C NMR data match those in literature.

#### Synthesis o*f* 1-phenylhexan-3-one (**56**)

(*E*)-1-phenylhex-1-en-3-one (10 mmol), NaBH_4_ (40 mmol) and Pd/C (12 mmol) was placed in 100 mL round bottomed flask. The flask was evacuated and back filled with argon three times. Toluene of 50 mL was slowly added at 0 °C. After stirring for 5 minutes, acetic acid (20 mmol) was slowly added at 0 °C within 10 minutes. The mixture was stirred for 2 h, filtered and concentrated under reduced pressure. The residue was purified by silica gel column chromatography using petroleum ether–ethyl acetate (10:1) to yield 1-phenylhexan-3-one (**56**) as a yellow oil in 87% yield. The ^1^H and/or ^13^C NMR data match those in literature.

#### Synthesis of (3-ethoxypropyl)benzene (**57**)

To the solution of 3-phenylpropan-1-ol (10 mmol) in dry THF (50 mL) was slowly added NaH (12 mmol) at 0 °C. The mixture was vigorously stirred at 0 °C for 0.5 h. Ethyl iodide (15 mmol) was slowly added at 0 °C, and stirred for 12 h. The mixture was quenched with H_2_O (50.0 mL) and extracted with DCM. The combined organic phase was dried over Na_2_SO_4_, filtered and concentrated under reduced pressure. The residue was purified by silica gel column chromatography using petroleum ether–ethyl acetate (15:1) to afford (3-ethoxypropyl)benzene (**57**) as a yellow oil in 53% yield. The ^1^H and/or ^13^C NMR data match those in literature.

### Acaricidal Assay

*In vitro* acaricidal activity of the synthesized compounds was performed according to our previously reported method^25,26^. In brief, a tested compound was dissolved in a mixed solvent of dimethyl sulfoxide (DMSO), Tween-80 and normal saline (1:1:8, V/V) to prepare a test solution. *Psoroptes cuniculi* adult mites of both sexes isolated from the scabs and the cerumen of naturally infected rabbits were used as tested objects. The mites were placed in 24-well flat-bottomed cell culture plates (10 adult mites each well) and followed by addition of 0.6 mL of the tested solution into each the well. Five replicates were made for each test. The same mixed solvent without tested compounds as above was used as an untreated control. Ivermectin, an acaricidal drug standard, in the same mixed solvent was used as a reference control.

The plates were placed in separate humidity chambers in saturated humidity conditions at 28 °C. After 24 h, the mites in the plate were observed under a stereomicroscope. When the persistent immobile mites were stimulated with a needle, lack of reaction was considered as the indication of death. Mortality was calculated using the following formula and expressed as means ± standard deviation (S. D.):

1 $${\rm{Mortality}}\,( \% )=\frac{{N}_{{\rm{t}}}-{N}_{{\rm{c}}}}{N-{N}_{{\rm{c}}}}\times 100$$

where, *N* is the number of the test mites in each test; *N*_c_ is the number of death mites in the untreated test; *N*_t_ the number of death mites in one treated test.

According to the same procedure as described above, a series of test concentrations of the compound was prepared by diluting the stock solution with the same mixed solvent and used to assay median lethal concentration value (LC_50_). Each concentration test was performed in triplicates. The probit value of the average mortality for each tested concentration and the corresponding lg[concentration] value were used to establish toxicity regression equation for concentration–effect by the linear least-square fitting method. The LC_50_ value of the compound and its confidence interval at 95% probability was calculated from its toxicity regression equation.

The concentration of 4.5 *μ*mol/mL of the compound was used to determine the mortality of the mites at different treatment time. The probit value of the average mortality for each the compound and the corresponding lg[treatment time] value were used to establish toxicity regression equation for time–effect by the linear least-square fitting method. The median lethal time value (LT_50_) value of the compound and its confidence interval at 95% probability was calculated from its toxicity regression equation. median lethal time value (LT_50_).

PRISM software ver. 5.0 (GraphPad Software Inc., San Diego, CA,USA) was used to analyze the data and establish toxicity regression equations. Duncan multiple comparison test was performed on the data to evaluate difference significance between the activities of various compounds.

### Data

Data will be made available upon request to the corresponding authors.

## Electronic supplementary material

Supplementary information
