# Supplementary material for: Design, Bioactivity and structure-activity of 3-Arylpropionate Derivatives as Potential High-Efficient Acaricides against Psoroptes Cuniculi
Source: Sci Rep. 2018 Jan 29;8:1797. doi: 10.1038/s41598-018-20140-7 (PMC5788918; doi:10.1038/s41598-018-20140-7)
Supplement: Supplementary file 1 — Supplementary information [file 41598_2018_20140_MOESM1_ESM.pdf]

Supporting information

Design, Bioactivity and structure-activity of 3-Arylpropionate Derivatives as Potential High-Efficient Acaricides against *Psoroptes Cuniculi*

Dongdong Chen[†,§], Ye Tian[†,♮,§], Mingxuan Xu[†], Xinyuan Wang[‡], Ding Li[†], Fang Miao[*,‡], Xinjuan Yang[*,†] and Le Zhou[*,†]

[†]College of Chemistry & Pharmacy, Northwest A&F University, Yangling 712100, Shaanxi Province, People's Republic of China

[‡]College of Life Science, Northwest A&F University, Yangling, Shaanxi, People's Republic of China

[♮]Zhengzhou Railway Vocational and Technical College, Zhengzhou, Henan, People's Republic of China

[*]Corresponding author (Tel: +86-29-87092226; Fax: +86-29-87092226; E-mail: zhoulechem@nwsuaf.edu.cn (L. Zhou); miaofangmf@163.com (F. Miao); yxjsn2@163.com (X. -J. Yang.)

[§]These authors contributed equally to this work.

Contents

NMR data and physical properties of Compounds **1–53** and **55–57** ……………………………………………………3

^{1}H NMR and ^{13}C NMR spectra of compounds **1–53** and **55–57**……………………………………………………17

HRMS of **13**, **29** and **30**……………………………………………………………………………………………72

MS of **9**, **16**, **17**, **19**, **20−23**, **25** and **43−45**……………………………………………………………………73

References………79

Table………..82

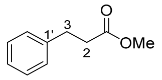

Methyl 3-phenylpropanoate (1). Yield: 96%; a colorless oil; ^{1}H NMR (500 MHz, $CDCl_3$) δ: 7.37–7.33 (m, 2H), 7.28–7.25 (m, 3H), 3.73 (s, 3H, OCH_3), 3.02 (t, J = 7.9 Hz, 2H, H-3), 2.70 (t, J = 7.9 Hz, 2H, H-2); ^{13}C NMR (125 MHz, $CDCl_3$) δ: 173.3 (C=O), 140.6 (C-1'), 128.6 (C-3', C-5'), 128.3 (C-2', C-6'), 126.3 (C-4'), 51.6 (OCH_3), 35.7 (C-2), 31.0 (C-3). The data match those in literature.[1]

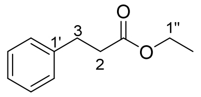

Ethyl 3-phenylpropanoate (2). Yield: 97%; a colorless oil; ^{1}H NMR (500 MHz, $CDCl_3$) δ: 7.30–7.25 (m, 2H, Ar-H), 7.21–7.18 (m, 3H, Ar-H), 4.12 (q, J = 7.1 Hz, 2H, H-1″), 2.95 (t, J = 7.9 Hz, 2H, H-3), 2.62 (t, J = 7.9 Hz, 2H, H-2), 1.23 (t, J = 7.1 Hz, 3H, H-2″); ^{13}C NMR (125 MHz, $CDCl_3$) δ: 172.9 (C=O), 140.6 (C-1'), 128.5 (C-3', C-5'), 128.3 (C-2', C-6'), 126.3 (C-4'), 60.4 (C-1″), 36.0 (C-2), 31.0 (C-3), 14.2 (C-2″). The data match those in literature[1].

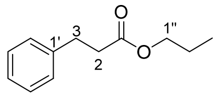

Propyl 3-phenylpropanoate (3). Yield: 92%; a colorless oil; ^{1}H NMR (500 MHz, $CDCl_3$) δ: 7.35–7.31 (m, 2H, Ar-H), 7.26–7.23 (m, 3H, Ar-H), 4.08 (t, J = 6.7 Hz, 2H, H-1″), 3.01 (t, J = 7.8 Hz, 2H, H-3), 2.68 (t, J = 7.8 Hz, 2H, H-2), 1.71–1.63 (m, 2H, H-2″), 0.96 (t, J = 7.4 Hz, 3H, H-3″); ^{13}C NMR (125 MHz, $CDCl_3$) δ: 173.0 (C=O), 140.6 (C-1'), 128.5 (C-3', C-5'), 128.3 (C-2', C-6'), 126.2 (C-4'), 66.1 (C-1″), 36.0 (C-2), 31.0 (C-3), 22.0 (C-2″), 10.4 (C-3″). The data match those in literature.[2]

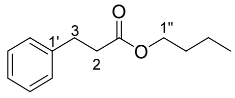

Butyl 3-phenylpropanoate (4). Yield: 90%; a colorless oil; ^{1}H NMR (500 MHz, $CDCl_3$) δ: 7.35–7.32 (m, 2H, Ar-H), 7.26–7.24 (m, 3H, Ar-H), 4.13 (t, J = 6.7 Hz, 2H, H-1″), 3.01 (t, J = 7.8 Hz, 2H, H-3), 2.68 (t, J = 7.8 Hz, 2H, H-2), 1.65–1.62 (m, 2H, H-2″), 1.42–1.37 (m, 2H, H-3″), 0.96 (t, J = 7.4 Hz, 3H, H-4″); ^{13}C NMR (125 MHz, $CDCl_3$) δ: 173.0 (C=O), 140.6 (C-1'), 128.5 (C-3', C-5'), 128.3 (C-2', C-6'), 126.2 (C-4'), 64.4 (C-1″), 36.0 (C-2), 31.1 (C-3), 30.7 (C-2″), 19.1 (C-3″), 13.7 (C-4″). The data match those in literature.[1]

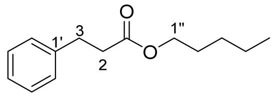

n-Pentyl 3-phenylpropanoate (**5**). Yield: 90%; a colorless oil; ^{1}H NMR (500 MHz, $CDCl_3$) δ: 7.35–7.31 (m, 2H, Ar-H), 7.26–7.23 (m, 3H, Ar-H), 4.11 (t, J = 6.7 Hz, 2H, H-1″), 3.01 (t, J = 7.8 Hz, 2H, H-3), 2.68 (t, J = 7.8 Hz, 2H, H-2), 1.68–1.62 (m, 2H, H-2″), 1.40–1.32 (m, 4H, H-3″, H-4″), 0.96 (t, J = 7.0 Hz, 3H, H-5″), agreement with those in literature.[1,3]

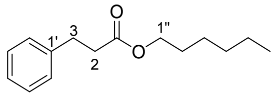

n-Hexyl 3-phenylpropanoate (**6**): Colorless oil in 89% yield. ^{1}H NMR (500 MHz, $CDCl_3$) δ: 7.35–7.31 (m, 2H, Ar-H), 7.27–7.24 (m, 3H, Ar-H), 4.11 (t, J = 6.7 Hz, 2H, H-1″), 3.01 (t, J = 7.9 Hz, 2H, H-3), 2.68 (t, J = 7.9 Hz, 2H, H-2), 1.66–1.62 (m, 2H, H-2″), 1.38–1.32 (m, 6H, H-3″, H-4″, H-5″), 0.94 (t, J = 6.9 Hz, 3H, H-6″); ^{13}C NMR (125 MHz, $CDCl_3$) δ: 173.1 (C=O), 140.6 (C-1'), 128.5 (C-3', C-5'), 128.3 (C-2', C-6'), 126.3 (C-4'), 64.70 (C-1″), 36.0 (C-2), 31.5 (C-4″), 31.0 (C-3), 28.6 (C-2″), 25.6 (C-3″), 22.6 (C-5″), 14.0 (C-6″). The data match those in literature.[1]

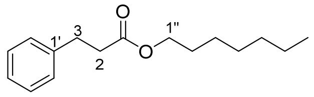

n-Heptyl 3-phenylpropanoate (7). Yield: 85%; a colorless oil; ^{1}H NMR (500 MHz, $CDCl_3$) δ: 7.35–7.31 (m, 2H, Ar-H), 7.26–7.24 (m, 3H, Ar-H), 4.11 (t, J = 6.7 Hz, 2H, H-1″), 3.01 (t, J = 7.8 Hz, 2H, H-3), 2.68 (t, J = 7.8 Hz, 2H, H-2), 1.66–1.63 (m, 2H, H-2″), 1.36–1.32 (m, 8H, H-3″, H-4″, H-5″, H-6″), 0.94 (t, J = 6.7 Hz, 3H, H-7″), agreement with those in literature;[4] ^{13}C NMR (125 MHz, $CDCl_3$) δ: 173.0 (C=O), 140.6 (C-1'), 128.5 (C-3', C-5'), 128.3 (C-2', C-6'), 126.3 (C-4'), 64.7 (C-1″), 36.0 (C-2), 31.7 (C-5″), 31.1 (C-3), 29.0 (C-4″), 28.7 (C-2″), 25.9 (C-3″), 22.6 (C-6″), 14.1 (C-7″).

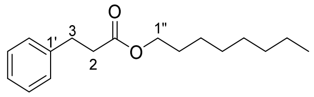

n-Octyl 3-phenylpropanoate (8). Yield: 80%; a colorless oil; ^{1}H NMR (500 MHz, $CDCl_3$) δ: 7.35–7.31 (m, 2H, Ar-H), 7.26–7.23 (m, 3H, Ar-H), 4.11 (t, J = 6.7 Hz, 2H, H-1″), 3.01 (t, J = 7.8 Hz, 2H, H-3), 2.68 (t, J = 7.8 Hz, 2H, H-2), 1.66–1.63 (m, 2H, H-2″), 1.34–1.32 (m, 10H, H-3″, H-4″, H-5″, H-6″, H-7″), 0.94 (t, J = 6.9 Hz, 3H, H-8″); ^{13}C NMR (125 MHz, $CDCl_3$) δ: 173.0 (C=O), 140.6 (C-1'), 128.5 (C-3', C-5'), 128.3 (C-2', C-6'), 126.2 (C-4'), 64.7 (C-1″), 36.0 (C-2), 31.8 (C-6″), 31.1 (C-3), 29.2 (C-4″), 29.2 (C-5″), 28.7 (C-2″), 25.9 (C-3″), 22.6 (C-7″), 14.1 (C-8″). The data match those in literature.[5]

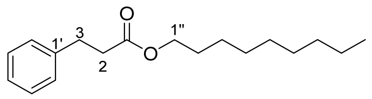

n-Nonyl 3-phenylpropanoate (9). Yield: 80%; a colorless oil; ^{1}H NMR (500 MHz, $CDCl_3$) δ: 7.35–7.31 (m, 2H, Ar-H), 7.26–7.23 (m, 3H, Ar-H), 4.11 (t, J = 6.7 Hz, 2H, H-1″), 3.01 (t, J = 7.8 Hz, 2H, H-3), 2.68 (t, J = 7.8 Hz, 2H, H-2), 1.66–1.63 (m, 2H, H-2″), 1.36–1.32 (m, 12H, H-3″, H-4″, H-5″, H-6″, H-7″, H-8″), 0.94 (t, J = 6.9 Hz, 3H, H-9″); ^{13}C NMR (125 MHz, $CDCl_3$) δ: 173.0 (C=O), 140.6 (C-1'), 128.5 (C-3', C-5'), 128.3 (C-2', C-6'), 126.3 (C-4'), 64.7 (C-1″), 36.0 (C-2), 31.9 (C-7″), 31.1 (C-3), 29.5 (C-5″), 29.3 (C-4″), 29.3 (C-6″), 28.7 (C-2″), 25.3 (C-3″), 22.7 (C-8″), 14.2 (C-9″). The data match those in literature.[6] Positive ESI-MS m/z: 299.11 $[M+Na]^{+}$.

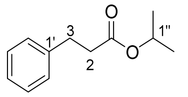

Isopropyl 3-phenylpropanoate (10). Yield: 95%; a colorless oil; ^{1}H NMR (500 MHz, $CDCl_3$) δ: 7.35–7.31 (m, 2H, Ar-H), 7.26–7.23 (m, 3H, Ar-H), 5.08–5.03 (m, 1H, H-1″), 2.99 (t, J = 7.8 Hz, 2H, H-3), 2.64 (t, J = 7.8 Hz, 2H, 2H, H-2), 1.25 (d, J = 6.3 Hz, 6H, 2×CH_3), matching those in literature.[7]

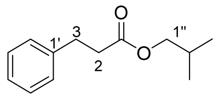

Isobutyl 3-phenylpropanoate (11). Yield: 92%; a colorless oil; ^{1}H NMR (500 MHz, $CDCl_3$) δ: 7.35–7.31 (m, 2H, Ar-H), 7.26–7.23 (m, 3H, Ar-H), 3.91 (d, J = 6.7 Hz, 2H, H-1″), 3.01 (t, J = 7.8 Hz, 2H, H-3), 2.70 (t, J = 7.8 Hz, 2H, H-2), 1.98–1.93 (m, 1H, H-2″), 0.95 (d, J = 6.3 Hz, 6H, 2×CH_3); ^{13}C NMR (125 MHz, $CDCl_3$) δ: 173.0 (C=O), 140.6 (C-1'), 128.5 (C-3', C-5'), 128.3 (C-2', C-6'), 126.3 (C-4'), 70.6 (C-1″), 35.9 (C-2), 31.1 (C-3), 27.7 (C-2″), 19.1 (2×CH_3). The data match those in literature.[1]

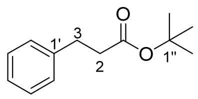

tert-Butyl 3-phenylpropanoate (12). Yield: 82%; a colorless oil; ^{1}H NMR (500 MHz, $CDCl_3$) δ: 7.35–7.31 (m, 2H, Ar-H), 7.26–7.23 (m, 3H, Ar-H), 2.97 (t, J = 7.8 Hz, 2H, H-3), 2.59 (t, J = 7.8 Hz, 2H, H-2), 1.47 (s, 9H, 3×CH_3); ^{13}C NMR (125 MHz, $CDCl_3$) δ: 172.3 (C=O), 140.8 (C-1'), 128.4 (C-3', C-5'), 128.3 (C-2', C-6'), 126.1 (C-4'), 80.3 (C-1″), 37.1 (C-2), 31.2 (C-3), 28.1 (3×CH_3). The data match those in literature.[1]

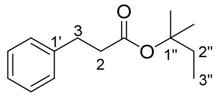

tert-Pentyl 3-phenylpropanoate (13). Yield: 70%; a colorless oil; ^{1}H NMR (500 MHz, $CDCl_3$) δ: 7.34–7.31 (m, 2H, Ar-H), 7.26–7.22 (m, 3H, Ar-H), 2.96 (t, J = 7.8 Hz, 2H, H-3), 2.60 (t, J = 7.8 Hz, 2H, H-2), 1.79 (q, J = 7.5 Hz, 2H, H-2″),

1.44 (s, 6H, 2×CH_3), 0.88 (t, J = 7.5 Hz, 3H, H-3″); ^{13}C NMR (125 MHz, $CDCl_3$) δ: 172.2 (C=O), 140.8 (C-1'), 128.4 (C-3', C-5'), 128.3 (C-2', C-6'), 126.1 (C-4'), 82.8 (C-1″), 37.1 (C-2), 33.5 (C-2″), 31.2 (C-3), 25.6 (2×CH_3), 8.2 (C-3″). HR-ESI-MS $[M+Na]^+$ calcd for $C_{14}H_{20}NaO_2^+$, 243.1356, found 243.1351.

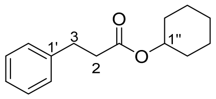

Cyclohexyl 3-phenylpropanoate (14). Yield: 75%; a colorless oil; ^1H NMR (500 MHz, $CDCl_3$) δ: 7.34–7.31 (m, 2H, Ar-H), 7.26–7.23 (m, 3H, Ar-H), 4.82–4.78 (m, 1H, H-1″), 3.00 (t, J = 7.8 Hz, 2H, H-3), 2.66 (t, J = 7.8 Hz, 2H, H-2), 1.86–1.84 (m, 2H, H-2″e, H-6″e), 1.76–1.73 (m, 2H, H-3″e, H-5″e), 1.59–1.56 (m, 1H, H-4″e), 1.44–1.35 (m, 4H, H-2″a, H-6″a, H-3″a, H-5″a), 1.32–1.28 (m, 1H, H-4″a); ^{13}C NMR (125 MHz, $CDCl_3$) δ: 172.4 (C=O), 140.7 (C-1'), 128.5 (C-3', C-5'), 128.4 (C-2', C-6'), 126.2 (C-4'), 72.7 (C-1″), 36.3 (C-2), 31.6 (C-2″, C-6″), 31.1 (C-3), 25.4 (C-4″), 23.7 (C-3″, C-5″). The data match those in literature.[1]

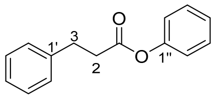

Phenyl 3-phenylpropanoate (15). Yield: 79%; a colorless oil; ^1H NMR (500 MHz, $CDCl_3$) δ: 7.44–7.37 (m, 4H, Ar-H), 7.34–7.26 (m, 4H, Ar-H), 7.08 (d, J = 7.9 Hz, 2H, H-2″, H-6″), 3.14 (t, J = 7.7 Hz, 2H, H-3), 2.95 (t, J = 7.7 Hz, 2H, H-2); ^{13}C NMR (125 MHz, $CDCl_3$) δ: 171.4 (C=O), 150.7 (C-1″), 140.2 (C-1'), 129.4 (C-3″, C-5″), 128.6 (C-3', C-5'), 128.4 (C-2', C-6'), 126.5 (C-4'), 125.8 (C-4″), 121.6 (C-2″, C-6″), 36.0 (C-2), 31.0 (C-3). The data match those in literature.[8]

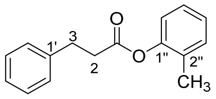

o-Tolyl 3-phenylpropanoate (16). Yield: 71%; a yellow oil; ^1H NMR (500 MHz, $CDCl_3$) δ: 7.41–7.34 (m, 4H, Ar-H), 7.32–7.24 (m, 3H, Ar-H), 7.21–7.18 (m, 1H, Ar-H), 7.00 (d, J = 7.7 Hz, 1H, H-3″), 3.17 (t, J = 7.7 Hz, 2H, H-3), 2.99 (t, J = 7.7 Hz, 2H, H-2), 2.15 (s, 3H, CH_3); ^{13}C NMR (125 MHz, $CDCl_3$) δ: 171.2 (C=O), 149.4 (C-1″), 140.2 (C-1'), 131.2 (C-3″), 130.1 (C-2″), 128.7 (C-3', C-5'), 128.5 (C-2', C-6'), 126.9 (C-5″), 126.5 (C-4″), 126.1 (C-4'), 121.9 (C-6″), 35.8 (C-2), 31.1 (C-3), 16.1 (CH_3). Positive ESI-MS m/z: 263.02 $[M+Na]^+$.

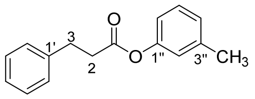

m-Tolyl 3-phenylpropanoate (17). Yield: 78%; a yellow oil; ^1H NMR (500 MHz, $CDCl_3$) δ: 7.41–7.38 (m, 2H, Ar-H), 7.35–7.29 (m, 4H, Ar-H), 7.10 (d, J = 7.3 Hz, 1H, H-4″), 6.89–6.88 (m, 2H), 3.15 (t, J = 7.7 Hz, 2H, H-3), 2.95 (t, J = 7.7

Hz, 2H, H-2), 2.41 (s, 3H, CH_3), matching those in literature;[9] ^{13}C NMR (125 MHz, $CDCl_3$) δ: 171.6 (C=O), 150.7 (C-1″), 140.2 (C-1'), 139.6 (C-3″), 129.2 (C-5″), 128.7 (C-3', C-5'), 128.5 (C-2', C-6'), 126.7 (C-4'), 126.5 (C-5″), 126.2 (C-2″), 118.5 (C-6″), 36.1 (C-2), 31.0 (C-3), 21.4 (CH_3). Positive ESI-MS m/z: 263.06 $[M+Na]^+$.

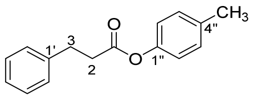

p-Tolyl 3-phenylpropanoate (18). Yield: 73%; a colorless oil; ^{1}H NMR (500 MHz, $CDCl_3$) δ: 7.38 (t, J = 7.6 Hz, 2H, , H-2', H-6'), 7.33–7.28 (m, 3H, Ar-H), 7.21 (d, J = 8.4 Hz, 2H, H-3″, H-5″), 6.95 (d, J = 8.4 Hz, 2H, H-2″, H-6″), 3.13 (t, J = 7.7 Hz, 2H, H-3), 2.93 (t, J = 7.7 Hz, 2H, H-2), 2.39 (s, 3H, CH_3); ^{13}C NMR (125 MHz, $CDCl_3$) δ: 171.6 (C=O), 148.5 (C-1″), 140.2 (C-1'), 135.5 (C-4″), 129.9 (C-3″, C-5″), 128.6 (C-3', C-5'), 128.4 (C-2', C-6'), 126.5 (C-4'), 121.2 (C-2″, C-6″), 36.0 (C-2), 31.0 (C-3), 20.9 (CH_3). The data match those in literature.[10]

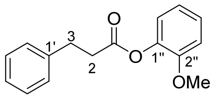

2-Methoxyphenyl 3-phenylpropanoate (19). Yield: 80%; a yellow oil; ^{1}H NMR (500 MHz, $CDCl_3$) δ: 7.38–7.34 (m, 4H, Ar-H), 7.31–7.25 (m, 2H, Ar-H), 7.03–6.99 (m, 3H, Ar-H), 3.85 (s, 3H, CH_3), 3.16 (t, J = 7.7 Hz, 2H, H-3), 2.98 (t, J = 7.7 Hz, 2H, H-2); ^{13}C NMR (125 MHz, $CDCl_3$) δ: 171.0 (C=O), 151.2 (C-2″), 140.4 (C-1″), 139.9 (C-1'), 128.6 (C-3', C-5'), 128.5 (C-2', C-6'), 126.9 (C-4″), 126.4 (C-4'), 122.8 (C-5″), 120.8 (C-6″), 112.5 (C-3″), 55.9 (CH_3), 35.6 (C-2), 31.0 (C-3). Positive ESI-MS m/z: 257.82 $[M+H]^+$.

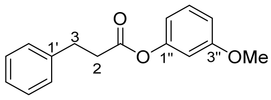

3-Methoxyphenyl 3-phenylpropanoate (20). Yield: 73%; a colorless oil; ^{1}H NMR (500 MHz, $CDCl_3$) δ: 7.41–7.38 (m, 2H, Ar-H), 7.35–7.29 (m, 4H, Ar-H), 6.84 (dd, J = 8.3, 1.7 Hz, 1H, H-6″), 6.69 (dd, J = 8.1, 1.3 Hz, 1H, H-4″), 6.62 (t, J = 2.1 Hz, 1H, H-2″), 3.84 (s, 3H, CH_3), 3.17 (t, J = 7.7 Hz, 2H, H-3), 2.95 (t, J = 7.7 Hz, 2H, H-2), matching those in literature;[9] ^{13}C NMR (125 MHz, $CDCl_3$) δ: 171.4 (C=O), 160.5 (C-3″), 151.7 (C-1″), 140.2 (C-1'), 129.8 (C-5'), 128.7 (C-3', C-5'), 128.5 (C-2', C-6'), 126.5 (C-4'), 113.8 (C-6″), 111.8 (C-4″), 107.6 (C-2″), 55.4 (CH_3), 36.0 (C-2), 31.0 (C-3). Positive ESI-MS m/z: 279.00 $[M+Na]^+$.

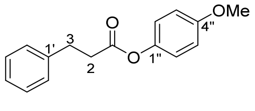

4-Methoxyphenyl 3-phenylpropanoate (21). Yield: 85%; a yellow oil; ^{1}H NMR (500 MHz, $CDCl_3$) δ: 7.31 (t, J = 7.4 Hz, 2H, Ar-H), 7.26–7.23 (m, 3H, Ar-H), 6.92 (d, J = 9.1 Hz, H-3″, H-5″), 6.86 (d, J = 9.1 Hz, H-2″, H-6″), 3.78 (s, 3H, CH_3), 3.06 (t, J = 7.7 Hz, 2H, H-3), 2.86 (t, J = 7.7 Hz, 2H, H-2), matching those in literature;[9] ^{13}C NMR (125 MHz, $CDCl_3$) δ: 171.8 (C=O), 157.3 (C-4″), 144.2 (C-1″), 140.2 (C-1'), 128.6 (C-3', C-5'), 128.4 (C-2', C-6'), 126.5 (C-4'), 122.3 (C-2″, C-6″), 114.5 (C-3″, C-5″), 55.6 (CH_3), 36.0 (C-2), 31.0 (C-3). Positive ESI-MS m/z: 279.04 $[M+Na]^+$.

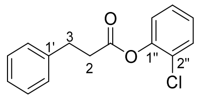

2-Chlorophenyl 3-phenylpropanoate (22). Yield: 79%; a colorless oil; ^{1}H NMR (500 MHz, $CDCl_3$) δ: 7.49 (dd, J = 8.0, 1.3 Hz, 1H, H-3″), 7.41 – 7.29 (m, 6H, Ar-H), 7.24 (t×2, J = 7.7, 1.3 Hz, 1H, H-4″), 7.12 (dd, J = 8.0, 1.3 Hz, 1H, H-6″), 3.18 (t, J = 7.8 Hz, 2H, H-3), 3.02 (t, J = 7.8 Hz, 2H, H-2), matching those in literature;[9] ^{13}C NMR (125 MHz, $CDCl_3$) δ: 170.5 (C=O), 147.1 (C-1″), 140.1 (C-1'), 130.4 (C-3″), 128.7 (C-3', C-5'), 128.5 (C-2', C-6'), 127.8 (C-2″), 127.1 (C-5″), 127.0 (C-4″), 126.5 (C-4'), 123.8 (C-6″), 35.6 (C-2), 30.9 (C-3). Positive ESI-MS m/z: 283.12 $[M+Na]^+$.

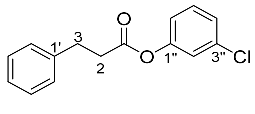

3-Chlorophenyl 3-phenylpropanoate (23). Yield: 79%; a brown solid; m.p. 34–35°C; ^{1}H NMR (500 MHz, $CDCl_3$) δ: 7.41 – 7.38 (m, 2H, Ar-H), 7.36 – 7.30 (m, 4H, Ar-H), 7.29 – 7.26 (m, 1H, Ar-H), 7.11 (t, J = 1.9 Hz, 1H, H-2″), 7.01 – 6.94 (m, 1H, Ar-H), 3.14 (t, J = 7.6 Hz, 2H, H-3), 2.95 (t, J = 7.7 Hz, 2H, H-2); ^{13}C NMR (125 MHz, $CDCl_3$) δ: 171.0 (C=O), 151.2 (C-1″), 140.0 (C-1'), 134.7 (C-3″), 130.2 (C-5″), 128.7 (C-3', C-5'), 128.5 (C-2', C-6'), 126.6 (C-4'), 126.2 (C-4″), 122.3 (C-2″), 120.0 (C-6″), 36.0 (C-2), 30.9 (C-3). Positive ESI-MS m/z: 283.06 $[M+Na]^+$.

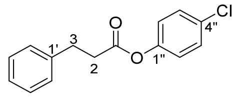

4-Chlorophenyl 3-phenylpropanoate (24). Yield: 77%; a white solid; m.p. 55–56 °C (lit. 57 °C). ^{1}H NMR (500 MHz, $CDCl_3$) δ: 7.40–7.36 (m, 4H, Ar-H), 7.32–7.28 (m, 3H, Ar-H), 7.00 (dd, J = 8.5, 1.5 Hz, 2H, H-2', H-6'), 3.12 (t, J = 7.7 Hz, 2H, H-3), 2.94 (t, J = 7.7 Hz, 2H, H-2); ^{13}C NMR (125 MHz, $CDCl_3$) δ: 171.2 (C=O), 149.1 (C-1″), 140.0 (C-1'), 131.2 (C-4″), 129.5 (C-3″, C-5″), 128.6 (C-3', C-5'), 128.4 (C-2', C-6'), 126.6 (C-4'), 122.9 (C-2″, C-6″), 35.9 (C-2), 30.9 (C-3). The data match those in literature.[8]

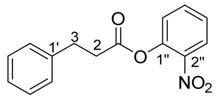

2-Nitrophenyl 3-phenylpropanoate (25). Yield: 81%; a pale yellow solid; m.p. 74–75°C; ^{1}H NMR (500 MHz, $CDCl_3$) δ: 8.14 (dd, J = 8.2, 1.5 Hz, 1H, H-3″), 7.69 (t×2, J = 7.9, 1.5 Hz, 1H, H-4″), 7.44 (t×2, J = 7.9, 1.2 Hz, 1H, H-5″), 7.40–7.37 (m, 2H, Ar-H), 7.34–7.29 (m, 3H, Ar-H), 7.21 (d, J = 8.1 Hz, 1H, H-6″), 3.16 (t, J = 7.8 Hz, 2H, H-3), 3.04 (t, J = 7.8 Hz, 2H, H-2); ^{13}C NMR (125 MHz, $CDCl_3$) δ: 170.6 (C=O), 144.1 (C-1″), 141.8 (C-2″), 139.9 (C-1'), 134.8 (C-5″), 128.7 (C-3', C-5'), 128.4 (C-2', C-6'), 126.7 (C-6″), 126.5 (C-4'), 125.8 (C-3″), 125.3 (C-4″), 35.7 (C-2), 30.5 (C-3). Positive ESI-MS m/z: 294.88 $[M+Na]^+$.

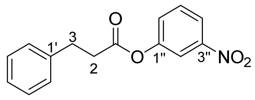

3-Nitrophenyl 3-phenylpropanoate (26). Yield: 82%; a yellow solid; m.p. 67–68°C; ^{1}H NMR (500 MHz, $CDCl_3$) δ: 8.16–8.14 (m, 1H, H-4″), 7.95 (t, J = 2.2 Hz, 1H, H-2″), 7.58 (t, J = 8.2 Hz, 1H, H-6″), 7.42–7.38 (m, 3H, Ar-H), 7.35–7.30 (m, 3H, Ar-H), 3.15 (t, J = 7.8 Hz, 2H, H-3), 3.00 (t, J = 7.8 Hz, 2H, H-2); ^{13}C NMR (125 MHz, $CDCl_3$) δ: 170.8 (C=O), 151.0 (C-1″), 148.8 (C-3″), 139.7 (C-1'), 130.0 (C-5″), 128.7 (C-3', C-5'), 128.4 (C-2', C-6'), 128.1 (C-6″), 126.7 (C-4'), 120.8 (C-4″), 117.4 (C-2″), 35.9 (C-2), 30.9 (C-3).

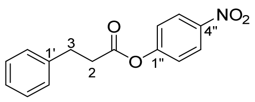

4-Nitrophenyl 3-phenylpropanoate (27). Yield: 83%; a yellow solid; m.p. 96–97°C. ^{1}H NMR (500 MHz, $CDCl_3$) δ: 8.29 (d, J = 9.1 Hz, 2H, H-3″, H-5″), 7.40–7.37 (m, 2H, Ar-H), 7.31 (d, J = 8.0 Hz, 3H), 7.23 (d, J = 9.1 Hz, 2H, H-2″, H-6″), 3.14 (t, J = 7.8 Hz, 2H, H-3), 3.00 (t, J = 7.8 Hz, 2H, H-2), matching those in literature;[11] ^{13}C NMR (125 MHz, $CDCl_3$) δ: 170.5 (C=O), 155.4 (C-4″), 145.4 (C-1″), 139.7 (C-1'), 128.7 (C-3', C-5'), 128.4 (C-2', C-6'), 126.7 (C-4'), 125.2 (C-3″, C-5″), 122.4 (C-2″, C-6″), 36.0 (C-2), 30.8 (C-3).

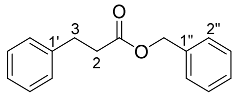

Benzyl 3-phenylpropanoate (28). Yield: 81%; a colorless oil; ^{1}H NMR (500 MHz, $CDCl_3$) δ: 7.43–7.33 (m, 7H, Ar-H), 7.29–7.26 (m, 3H, Ar-H), 5.19 (s, 2H, CH_2), 3.05 (t, J = 7.8 Hz, 2H, H-3), 2.76 (t, J = 7.8 Hz, 2H, H-2); ^{13}C NMR (125 MHz, $CDCl_3$) δ: 172.8 (C=O), 140.5 (C-1'), 136.0 (C-1″), 128.62 (C-3', C-5'), 128.58 (C-3″, C-5″), 128.4 (C-2', C-6'), 128.3 (C-2″, C-6″), 126.4 (C-4', C-4″), 66.4 (CH_2), 36.0 (C-2), 31.0 (C-3). The data match those in literature.[12]

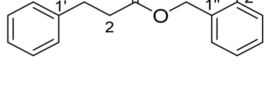

2-Nitrobenzyl 3-phenylpropanoate (29). Yield: 80%; a brown oil; ^{1}H NMR (500 MHz, $CDCl_3$) δ: 8.14 (dd, J = 8.2, 1.0 Hz, 1H, H-3″), 7.62 (t×2, J = 7.7, 1.1 Hz, 1H, H-5″), 7.51 (t×2, J = 7.7, 1.1 Hz, 1H, H-4″), 7.43 (d, J = 7.7 Hz, 1H, H-6″), 7.36–7.33 (m, 2H, Ar-H), 7.28–7.25 (m, 3H, Ar-H), 5.19 (s, 2H, CH_2), 3.05 (t, J = 7.8 Hz, 2H, H-3), 2.81 (t, J = 7.8 Hz, 2H, H-2); ^{13}C NMR (125 MHz, $CDCl_3$) δ: 172.4 (C=O), 147.7 (C-2″), 143.3 (C-1″, C-6″), 140.1 (C-1'), 128.6 (C-3', C-5'), 128.34 (C-2', C-6'), 128.31 (C-4″, C-5″), 126.5 (C-4'), 123.8 (C-3″), 64.8 (CH_2), 35.7 (C-2), 30.9 (C-3). HR-ESI-MS $[M+Na]^+$ calcd for C_{16}H15NNaO_4^+, 308.0893, found 308.0889.

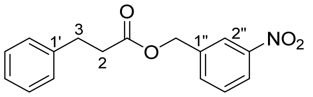

3-Nitrobenzyl 3-phenylpropanoate (30). Yield: 78%; a pale yellow oil; ^{1}H NMR (500 MHz, $CDCl_3$) δ: 8.23–8.21 (m, 2H, H-2″, H-4″), 7.64 (d, J = 7.7 Hz, 1H, H-6″), 7.58–7.55 (m, 1H, H-5″), 7.34–7.31 (m, 2H, Ar-H), 7.26–7.23 (m, 3H, Ar-H), 5.23 (s, 2H, CH_2), 3.03 (t, J = 7.7 Hz, 2H, H-3), 2.78 (t, J = 7.7 Hz, 2H, H-2); ^{13}C NMR (125 MHz, $CDCl_3$) δ: 172.5 (C=O), 148.4(C-3″), 140.1 (C-1'), 138.1 (C-1″), 134.0 (C-6″), 129.6 (C-5″), 128.6 (C-3', C-5'), 128.3 (C-2', C-6'), 126.4 (C-4'), 123.2 (C-4″), 122.9 (C-2″), 64.8 (CH_2), 35.7 (C-2), 30.9 (C-3). HR-ESI-MS $[M+Na]^+$ calcd for C_{16}H15NNaO_4^+, 308.0893, found 308.0879.

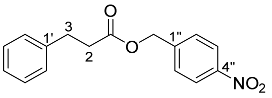

4-Nitrobenzyl 3-phenylpropanoate (31). Yield: 78%; a yellow oil; ^{1}H NMR (500 MHz, $CDCl_3$) δ: 8.22 (d, J = 8.5 Hz, 2H, H-3″, H-5″), 7.44 (d, J = 8.5 Hz, 2H, H-2″, H-4″), 7.35–7.31 (m, 2H, Ar-H), 7.28–7.24 (m, 3H, Ar-H), 5.24 (s, 2H, CH_2), 3.04 (t, J = 7.6 Hz, 2H, H-3), 2.79 (t, J = 7.6 Hz, 2H, H-2). The data match those in literature.[13]

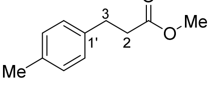

Methyl 3-(p-tolyl)propanoate (32). Yield: 84%; a white solid; m.p. 35–36°C. ^{1}H NMR (500 MHz, $CDCl_3$) δ: 7.16 (s, 4H, Ar-H), 3.73 (s, 3H, OCH_3), 2.98 (t, J = 7.8 Hz, 2H, H-3), 2.68 (t, J = 7.8 Hz, 2H, H-2), 2.38 (s, 3H, CH_3); ^{13}C NMR (125 MHz, $CDCl_3$) δ: 173.5 (C=O), 137.5 (C-1'), 135.8 (C-4'), 129.2 (C-3', C-5'), 128.2 (C-2', C-6'), 51.7 (OCH_3), 35.9 (C-2), 30.6 (C-3), 21.1 (CH_3). The data match those in literature.[14]

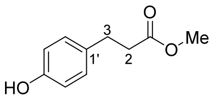

Methyl 3-(4-hydroxyphenyl)propanoate (33). Yield: 51%; a white solid; m.p. 39–40°C. ^{1}H NMR (500 MHz, $CDCl_3$) δ:

7.08 (d, J = 8.3 Hz, 2H, H-2', H-6'), 6.81 (d, J = 8.3 Hz, 2H, H-3', H-5'), 6.38 (s, 1H, OH), 3.72 (s, 3H, CH_3), 2.92 (t, J = 7.7 Hz, 2H, H-3), 2.66 (t, J = 7.7 Hz, 2H, H-2); ^{13}C NMR (125 MHz, $CDCl_3$) δ: 174.4 (C=O), 154.4 (C-4'), 132.2 (C-1'), 129.4 (C-2', C-6'), 115.5 (C-3', C-5'), 51.9 (OCH_3), 36.2 (C-2), 30.1 (C-3). The data match those in literature.[15]

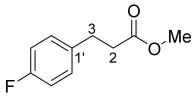

Methyl 3-(4-fluorophenyl)propanoate (34). Yield: 90%; a colorless oil; ^{1}H NMR (500 MHz, $CDCl_3$) δ: 7.20 (t, J = 8.6 Hz, 2H, H-2', H-6'), 7.01 (t, J = 8.6 Hz, 2H, H-3', H-5'), 3.71 (s, 3H, CH_3), 2.97 (t, J = 7.7 Hz, 2H, H-3), 2.65 (t, J = 7.7 Hz, 2H, H-2); ^{13}C NMR (125 MHz, $CDCl_3$) δ: 173.2 (C=O), 161.5 (d, J = 244.0 Hz, C-4'), 136.2 (d, J = 3.3 Hz, C-1'), 129.8 (d, J = 7.8 Hz, C-2', C-6'), 115.3 (d, J = 21.2 Hz, C-3', C-5'), 51.7 (OCH_3), 35.8 (C-2), 30.16 (C-3). The data match those in literature.[16]

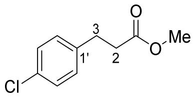

Methyl 3-(4-chlorophenyl)propanoate (35). Yield: 92%; a colorless oil; ^{1}H NMR (500 MHz, $CDCl_3$) δ: 7.29 – 7.26 (m, 2H, H-3', H-5'), 7.21 (d, J = 8.3 Hz, 2H, H-2', H-6'), 3.65 (s, 3H, CH_3), 2.92 (t, J = 7.6 Hz, 2H, H-3), 2.64 (t, J = 7.6 Hz, 2H, H-2); ^{13}C NMR (125 MHz, $CDCl_3$) δ: 173.4 (C=O), 139.4 (C-1'), 131.6 (C-4'), 129.6 (C-2', C-6'), 128.1 (C-3', C-5'), 50.7 (OCH_3), 34.9 (C-2), 29.8 (C-3). The data match those in literature.[14]

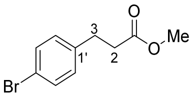

Methyl 3-(4-bromophenyl)propanoate (36). Yield: 90%; a colorless oil; ^{1}H NMR (500 MHz, $CDCl_3$) δ: 7.42 (d, J = 8.0 Hz, 2H, H-3', H-5'), 7.15 (d, J = 8.0 Hz, 2H, H-2', H-6'), 3.65 (s, 3H, CH_3), 2.89 (t, J = 7.5 Hz, 2H, H-3), 2.64 (t, J = 7.5 Hz, 2H, H-2); ^{13}C NMR (125 MHz, $CDCl_3$) δ: 173.4 (C=O), 139.9 (C-1'), 131.1 (C-2', C-6'), 123.0 (C-3', C-5'), 119.5 (C-4'), 50.7 (OCH_3), 34.8 (C-2), 29.9 (C-3). The data match those in literature.[17]

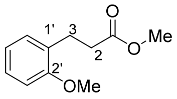

Methyl 3-(2-methoxyphenyl)propanoate (37). Yield: 93%; a colorless oil; ^{1}H NMR (500 MHz, $CDCl_3$) δ: 7.27 – 7.23 (m, 1H, H-5'), 7.20 (dd, J = 7.4, 1.3 Hz, 1H, H-3'), 6.93 (t, J = 7.4 Hz, 1H, H-4'), 6.89 (d, J = 8.2 Hz, 1H, H-6'), 3.87 (s, 3H, Ar-OC$\underline{H}_3$), 3.72 (s, 3H, OCH_3), 3.00 (t, J = 7.6 Hz, 2H, H-3), 2.67 ((t, J = 7.6 Hz, 2H, H-2); ^{13}C NMR (125 MHz, $CDCl_3$) δ: 173.8 (C=O), 157.5 (C-2'), 129.9 (C-1'), 128.9 (C-6'), 127.6 (C-4'), 120.5 (C-5'), 110.2 (C-3'), 55.2 (Ar-O$\underline{C}H_3$),

51.5 (OCH_3), 34.1 (C-2), 26.2 (C-3). The data match those in literature.[17]

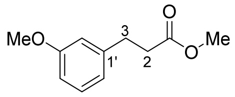

Methyl 3-(3-methoxyphenyl)propanoate (38). Yield: 94%; a colorless oil; ^{1}H NMR (500 MHz, $CDCl_3$) δ: 7.25 (t, J = 8.3 Hz, 1H, H-5'), 6.84 (d, J = 7.4 Hz, 1H, H-6'), 6.81 – 6.79 (m, 2H, H-2', H-4'), 3.84 (s, 3H, Ar-OC$\underline{H}_3$), 3.72 (s, 3H, OCH_3), 2.98 (t, J = 7.9 Hz, 2H, H-3), 2.68 (t, J = 7.9 Hz, 2H, H-2); ^{13}C NMR (125 MHz, $CDCl_3$) δ: 173.4 (C=O), 159.7 (C-3'), 142.2 (C-1'), 129.5 (C-5'), 120.6 (C-6'), 114.1 (C-2'), 111.6 (C-4'), 55.2 (Ar-O$\underline{C}H_3$), 51.7 (OCH_3), 35.7 (C-2), 31.0 (C-3). The data match those in literature.[18]

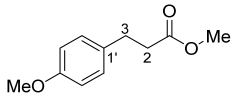

Methyl 3-(4-methoxyphenyl)propanoate (39). Yield: 72%; a yellow oil; ^{1}H NMR (500 MHz, $CDCl_3$) δ: 7.16 (d, J = 8.6 Hz, 2H, H-2', H-6'), 6.88 (d, J = 8.6 Hz, 2H, H-3', H-5'), 3.83 (s, 3H, Ar-OC$\underline{H}_3$), 3.71 (s, 3H, OCH_3), 2.94 (t, J = 7.8 Hz, 2H, H-3), 2.65 (t, J = 7.8 Hz, 2H, H-2); ^{13}C NMR (125 MHz, $CDCl_3$) δ: 173.4 (C=O), 158.1 (C-4'), 132.6 (C-1'), 129.2 (C-2', C-6'), 114.0 (C-3', C-5'), 55.3 (Ar-O$\underline{C}H_3$), 51.6 (OCH_3), 36.0 (C-2), 30.1 (C-3). The data match those in literature.[19]

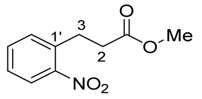

Methyl 3-(2-nitrophenyl)propanoate (40). Yield: 76%; a yellow solid; m.p. 55–56°C; ^{1}H NMR (500 MHz, $CDCl_3$) δ: 7.99 (d, J = 8.2 Hz, 1H, H-3'), 7.58 (t×2, J = 7.5, 1.0 Hz, 1H, H-5'), 7.46–7.41 (m, 2H, H-4', H-6'), 3.72 (s, 3H, OCH_3), 3.27 (t, J = 7.6 Hz, 2H, H-3), 2.78 (t, J = 7.6 Hz, 2H, H-2); ^{13}C NMR (125 MHz, $CDCl_3$) δ: 172.8 (C=O), 149.3 (C-2'), 135.6 (C-1'), 133.2 (C-3'), 132.2 (C-5'), 127.7 (C-6'), 124.9 (C-4'), 51.8 (CH_3), 34.7 (C-2), 28.4 (C-3). The data match those in literature.[20]

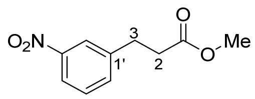

Methyl 3-(3-nitrophenyl)propanoate (41). Yield: 78%; a yellow solid; m.p. 45–46°C; ^{1}H NMR (500 MHz, $CDCl_3$) δ: 8.11 (d, J = 6.8 Hz, 2H, H-2', H-4'), 7.59 (d, J = 7.6 Hz, 1H, H-6'), 7.50 (t, J = 8.3 Hz, 1H, H-5'), 3.72 (s, 3H, OCH_3), 3.11 (t, J = 7.6 Hz, 2H, H-3), 2.72 (t, J = 7.6 Hz, 2H, H-2); ^{13}C NMR (125 MHz, $CDCl_3$) δ: 172.6 (C=O), 148.4 (C-3'), 142.5 (C-1'), 134.7 (C-6'), 129.4 (C-5'), 123.3 (C-2'), 121.6 (C-4'), 51.8 (CH_3), 35.0 (C-2), 30.5 (C-3).

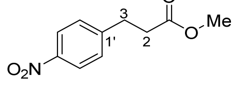

Methyl 3-(4-nitrophenyl)propanoate (42). Yield: 80%; a yellow solid; m.p. 73–74°C (lit.[21] 67–70°C); ^{1}H NMR (500 MHz, $CDCl_3$) δ: 8.19–8.16 (m, 2H, H-3', H-5'), 7.55 (d, J = 8.8 Hz, 2H, H-2', H-6'), 3.61 (s, 3H, OCH_3), 3.02 (t, J = 7.5 Hz, 2H, H-3), 2.72 (t, J = 7.5 Hz, 2H, H-2); ^{13}C NMR (125 MHz, $CDCl_3$) δ: 172.8 (C=O), 149.4 (C-1'), 146.6 (C-4'), 130.1 (C-2', C-6'), 123.9 (C-3', C-5'), 51.9 (CH_3), 34.5 (C-2), 30.4 (C-3). The data match those in literature.[21]

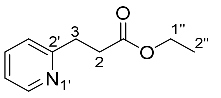

Ethyl 3-(pyridin-2-yl)propanoate (43). Yield: 63%; a yellow oil; ^{1}H NMR (500 MHz, $CDCl_3$) δ: 8.49 (d, J = 10.4 Hz, 2H, H-3', H-6'), 7.55 (d, J = 7.8 Hz, 1H, H-4'), 7.32 – 7.16 (m, H, H-5'), 4.14 (q, J = 7.1 Hz, 2H, H-1″), 2.97 (t, J = 7.6 Hz, 2H, H-3), 2.65 (t, J = 7.6 Hz, 2H, H-2), 1.24 (t, J = 7.1 Hz, 3H, H-2″); ^{13}C NMR (126 MHz, $CDCl_3$) δ: 172.3 (C=O), 149.9 (C-2'), 147.8 (C-6'), 135.8 (C-4'), 123.4 (C-3', C-5'), 60.6 (C-1″), 35.4 (C-2), 28.1 (C-3), 14.2 (C-2″). The data match those in literature.[22,23] Positive ESI-MS m/z: 180.10 [M+H]$^+$.

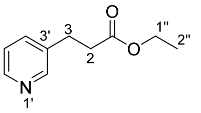

Ethyl 3-(pyridin-3-yl)propanoate (44). Yield: 65%; a yellow oil; ^{1}H NMR (500 MHz, $CDCl_3$) δ: 8.55 (s, 1H, H-2'), 7.62 (t, J = 7.1 Hz, 1H, H-6'), 7.31–7.14 (m, 2H, H-4', H-5'), 4.15 (q, J = 7.1 Hz, 2H, H-1″), 3.14 (t, J = 7.5 Hz, 2H, H-3), 2.82 (t, J = 7.5 Hz, 2H, H-2), 1.25 (t, J = 7.1 Hz, 3H, H-2″); ^{13}C NMR (125 MHz, $CDCl_3$) δ: 173.1 (C=O), 160.1 (C-2'), 149.3 (C-6'), 136.4 (C-3'), 123.0 (C-4'), 121.4 (C-5'), 60.4 (C-1″), 33.5 (C-2), 33.0 (C-3), 14.2 (C-2″). The data match those in literature.[23,24] Positive ESI-MS m/z: 180.08 [M+H]$^+$.

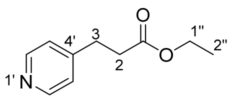

Ethyl 3-(pyridin-4-yl)propanoate (45). Yield: 68%; a yellow oil; ^{1}H NMR (500 MHz, $CDCl_3$) δ: 8.52 (d, J = 6.2 Hz, 2H, H-2', H-6'), 7.38 (d, J = 6.2 Hz, 2H, H-3', H-5'), 4.17 (q, J = 7.1 Hz, 2H, H-1″), 3.09 (t, J = 7.3 Hz, 2H, H-3), 2.73 (t, J = 7.3 Hz, 2H, H-2), 1.27 (t, J = 7.1 Hz, 3H, H-2″); ^{13}C NMR (126 MHz, $CDCl_3$) δ: 171.5 (C=O), 153.9 (C-4'), 147.3 (C-2', C-6'), 125.3 (C-3', C-5'), 61.0 (C-1″), 33.8 (C-2), 30.0 (C-3), 14.2 (C-2″). The data match those in literature.[25]

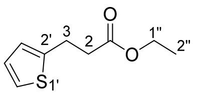

Ethyl 3-(thiophen-2-yl)propanoate (46). Yield: 83%; a pale yellow oil; ^{1}H NMR (500 MHz, $CDCl_3$) δ: 7.17 (dd, J = 5.1, 1.2 Hz, 1H, H-5'), 6.96 (dd, J = 5.1, 3.5 Hz, 1H, H-3'), 6.89 – 6.84 (m, 1H, H-4'), 4.20 (q, J = 7.1 Hz, 2H, H-1″), 3.21 (t, J

= 7.6 Hz, 2H, H-3), 2.72 (t, J = 7.6 Hz, 2H, H-2), 1.30 (t, J = 7.1 Hz, 3H, H-2″); ^{13}C NMR (125 MHz, $CDCl_3$) δ: 172.4 (C=O), 143.2 (C-2'), 126.8 (C-4'), 124.6 (C-5'), 123.5 (C-3'), 60.6 (C-1″), 36.2 (C-2), 25.2 (C-3), 14.2 (C-2″). The data match those in literature.[26]

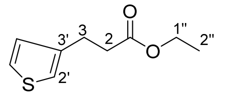

Ethyl 3-(thiophen-3-yl)propanoate (47). Yield: 79%; a pale yellow oil; ^{1}H NMR (500 MHz, $CDCl_3$) δ: 7.31 – 7.29 (m, 1H, H-5'), 7.02 (dd, J = 1.9, 0.9 Hz, 1H, H-2'), 6.99 (dd, J = 4.9, 0.9 Hz, 1H, H-4'), 4.18 (q, J = 7.1 Hz, 2H, H-1″), 3.02 (t, J = 7.7 Hz, 2H, H-3), 2.67 (t, J = 7.7 Hz, 2H, H-2), 1.29 (t, J = 7.1 Hz, 3H, H-2″); ^{13}C NMR (125 MHz, $CDCl_3$) δ: 172.9 (C=O), 140.9 (C-3'), 128.0 (C-4'), 125.6 (C-5'), 120.6 (C-2'), 60.5 (C-1″), 35.2 (C-2), 25.5 (C-3), 14.2 (C-2″). The data match those in literature.[24]

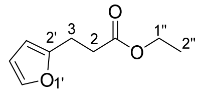

Ethyl 3-(furan-2-yl)propanoate (48). Yield: 88%; a pale yellow oil; ^{1}H NMR (500 MHz, $CDCl_3$) δ: 7.33 (d, J = 0.9 Hz, 1H, H-5'), 6.30 (dd, J = 2.8, 1.9 Hz, 1H, H-4'), 6.05 (dd, J = 1.9, 0.9 Hz, 1H, H-3'), 4.18 (q, J = 7.1 Hz, 2H, H-1″), 3.00 (t, J = 7.6 Hz, 2H, H-3), 2.68 (t, J = 7.6 Hz, 2H, H-2), 1.28 (t, J = 7.1 Hz, 3H, 3H, H-2″); ^{13}C NMR (125 MHz, $CDCl_3$) δ: 172.5 (C=O), 154.2 (C-2'), 141.2 (C-5'), 110.2 (C-4'), 105.3 (C-3'), 60.5 (C-1″), 32.8 (C-2), 23.5 (C-3), 14.2 (C-2″). The data match those in literature.[26]

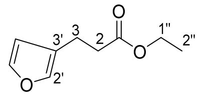

Ethyl 3-(furan-3-yl)propanoate (49). Yield: 90%; a pale yellow oil; ^{1}H NMR (500 MHz, $CDCl_3$) δ: 7.38 (s-like, 1H, H-5'), 7.28 – 7.27 (m, 1H, H-2'), 6.31 (s, 1H, H-4'), 4.17 (q, J = 7.1 Hz, 2H, H-1″), 2.80 (t, J = 7.6 Hz, 2H, H-3), 2.59 (t, J = 7.6 Hz, 2H, H-2), 1.28 (t, J = 7.2 Hz, 3H, H-2″); ^{13}C NMR (125 MHz, $CDCl_3$) δ 172.9 (C=O), 142.9 (C-5'), 139.1 (C-2'), 123.6 (C-3'), 110.8 (C-4'), 60.4 (C-1″), 34.8 (C-2), 20.3 (C-3), 14.2 (C-2″). The data match those in literature.[27]

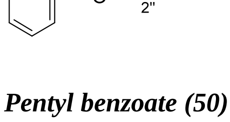

Pentyl benzoate (50). Yield: 90%; a colorless oil; ^{1}H NMR (500 MHz, $CDCl_3$) δ: 8.10 (dd, J = 8.4, 1.3 Hz, H-2', H-6'), 7.59 (ttt, J = 7.4, 1.2 Hz, H-4'), 7.48 (t, J = 7.7 Hz, 2H, H-3', H-5'), 4.36 (t, J = 6.7 Hz, 2H, H-1″), 1.85 – 1.79 (m, 2H, H-2″), 1.49 – 1.41 (m, 4H, H-2″, H-3″), 0.98 (t, J = 7.1 Hz, 3H, H-5″); ^{13}C NMR (125 MHz, $CDCl_3$) δ: 166.7 (C=O), 132.8

(C-4'), 130.6 (C-1'), 129.6 (C-2', C-6'), 128.3 (C-3', C-5'), 65.2 (C-1″), 28.5 (C-2″), 28.2 (C-3″), 22.4 (C-4″), 14.0 (C-5″). The data match those in literature.[28]

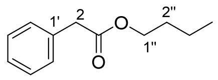

Butyl 2-phenylacetate (51). Yield: 91%; a colorless oil; ^{1}H NMR (500 MHz, $CDCl_3$) δ: 7.39 – 7.31 (m, 5H, Ar-H), 4.15 (t, J = 6.7 Hz, 2H, H-1″), 3.67 (s, 2H, H-2), 1.80 – 1.54 (m, 2H, H-2″), 1.40 (m, 2H, H-3″), 0.97 (t, J = 7.5 Hz, 3H, H-4″); ^{13}C NMR (125 MHz, $CDCl_3$) δ: 171.7 (C=O), 134.3 (C-1'), 129.3 (C-2', C-6'), 128.6 (C-3', C-5'), 127.1 (C-4'), 64.8 (C-1″), 41.5 (C-2), 30.6 (C-2″), 19.1 (C-3″), 13.7 (C-4″). The data match those in literature.[29]

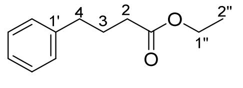

Ethyl 4-phenylbutanoate (52). Yield: 92%; a colorless oil; ^{1}H NMR (500 MHz, $CDCl_3$) δ: 7.35 – 7.31 (m, 2H, Ar-H), 7.26 – 7.23 (m, 3H, Ar-H), 4.18 (q, J = 7.1 Hz, 2H, H-1″), 2.71 (t, J = 7.6 Hz, 2H, H-4), 2.37 (t, J = 7.6 Hz, 2H, H-2), 2.09 – 1.93 (m, 2H, H-3), 1.31 (t, J = 7.1 Hz, 3H, H-2″); ^{13}C NMR (125 MHz, $CDCl_3$) δ: 173.5 (C=O), 141.5 (C-1'), 128.5 (C-3', C-5'), 128.4 (C-2', C-6'), 126.0 (C-4'), 60.3 (C-1″), 35.2 (C-4), 33.7 (C-2), 26.6 (C-3), 14.3 (C-2″). The data match those in literature.[30]

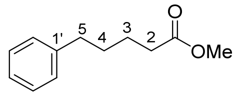

Methyl 5-phenylpentanoate (53). Yield: 93%; a colorless oil; ^{1}H NMR (500 MHz, $CDCl_3$) δ: 7.34 – 7.31 (m, 2H, Ar-H), 7.24 – 7.22 (m, 3H, Ar-H), 3.72 (s, 3H, CH_3), 2.69 (t, J = 7.0 Hz, 2H, H-5), 2.39 (t, J = 7.0 Hz, 2H, H-2), 1.73 – 1.70 (m, 4H, H-3, H-4); ^{13}C NMR (125 MHz, $CDCl_3$) δ: 174.1 (C=O), 142.2 (C-1'), 128.41 (C-3', C-5'), 128.35 (C-2', C-6'), 125.8 (C-4'), 51.5 (CH_3), 35.6 (C-5), 34.0 (C-2), 30.9 (C-4), 24.6 (C-3). The data match those in literature.[31]

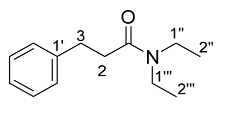

N,N-diethyl-3-phenylpropanamide (55). Yield: 82%; a yellow oil; ^{1}H NMR (500 MHz, $CDCl_3$) δ: 7.34–7.31 (m, 2H, Ar-H), 7.27–7.22 (m, 3H, Ar-H), 3.42 (q, J = 5.6 Hz, 2H, H-1″,), 3.26 (q, J = 5.6 Hz, 2H, H-2‴), 3.03 (t, J = 7.9 Hz, 2H, H-3), 2.64 (t, J = 7.9 Hz, 2H, H-2), 1.15 (t, J = 5.6 Hz, 6H, H-2″, H-2‴); ^{13}C NMR (125 MHz, $CDCl_3$) δ: 171.3 (C=O), 141.6 (C-1′), 128.49 (C-3′, C-5′), 128.48 (C-2′, C-6′), 126.1 (C-4′), 41.9 (C-1″), 40.3 (C-1‴), 35.1 (C-2), 31.7 (C-3), 14.3 (C-2″), 13.1 (C-2‴). The data match those in literature.[32]

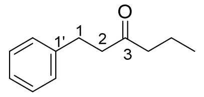

1-Phenylhexan-3-one (56). Yield: 87%; a yellow oil; ^{1}H NMR (500 MHz, $CDCl_3$) δ: 7.34 – 7.31 (m, 2H, Ar-H), 7.25 – 7.22 (m, 3H, Ar-H), 2.95 (t, J = 7.7 Hz, 2H, H-1), 2.77 (t, J = 7.7 Hz, 2H, H-2), 2.42 (t, J = 7.4 Hz, 2H, H-4), 1.68 – 1.61 (m, 2H, H-5), 0.95 (t, J = 7.4 Hz, 3H, H-6); ^{13}C NMR (125 MHz, $CDCl_3$) δ: 210.3 (C=O), 141.2 (C-1'), 128.5 (C-3', C-5'), 128.4 (C-2', C-6'), 126.1 (C-4'), 45.0 (C-2), 44.3 (C-4), 29.8 (C-1), 17.3 (C-5), 13.8 (C-6). The data match those in literature.[33]

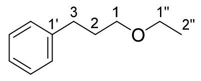

(3-Ethoxypropyl)benzene (57). Yield: 53%; a yellow oil; ^{1}H NMR (500 MHz, $CDCl_3$) δ: 7.36 – 7.31 (m, 2H, Ar-H), 7.27 – 7.23 (m, 3H, Ar-H), 3.54 (q, J = 7.0 Hz, 2H, H-1″), 3.49 (t, J = 6.5 Hz, 2H, H-1′), 2.76 (t, J = 7.7 Hz, 2H, H-3), 2.00 – 1.94 (m, 2H, H-2), 1.28 (t, J = 7.0 Hz, 3H, H-2″); ^{13}C NMR (125 MHz, $CDCl_3$) δ: 142.1 (C-1′), 128.5 (C-3′, C-5′), 128.3 (C-2′, C-6′), 125.8 (C-4′), 69.8 (C-1), 66.1 (C-1″), 32.4 (C-3), 31.4 (C-2), 15.3 (C-2″). The data match those in literature.[34]

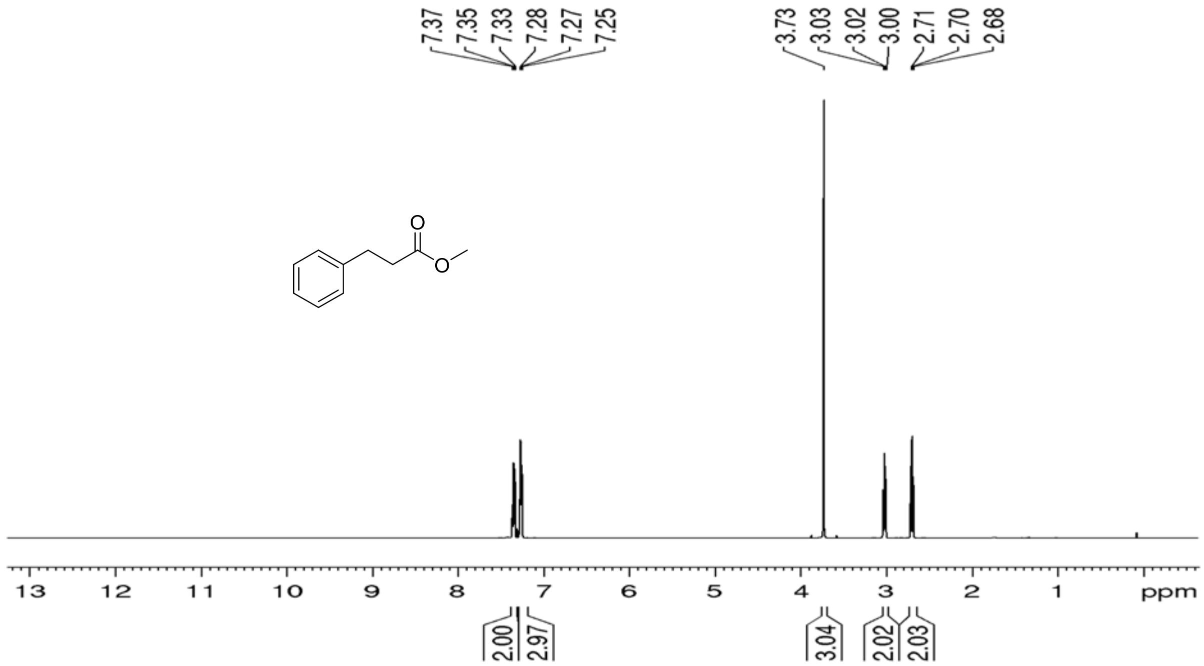

^{1}H NMR of compound **1**

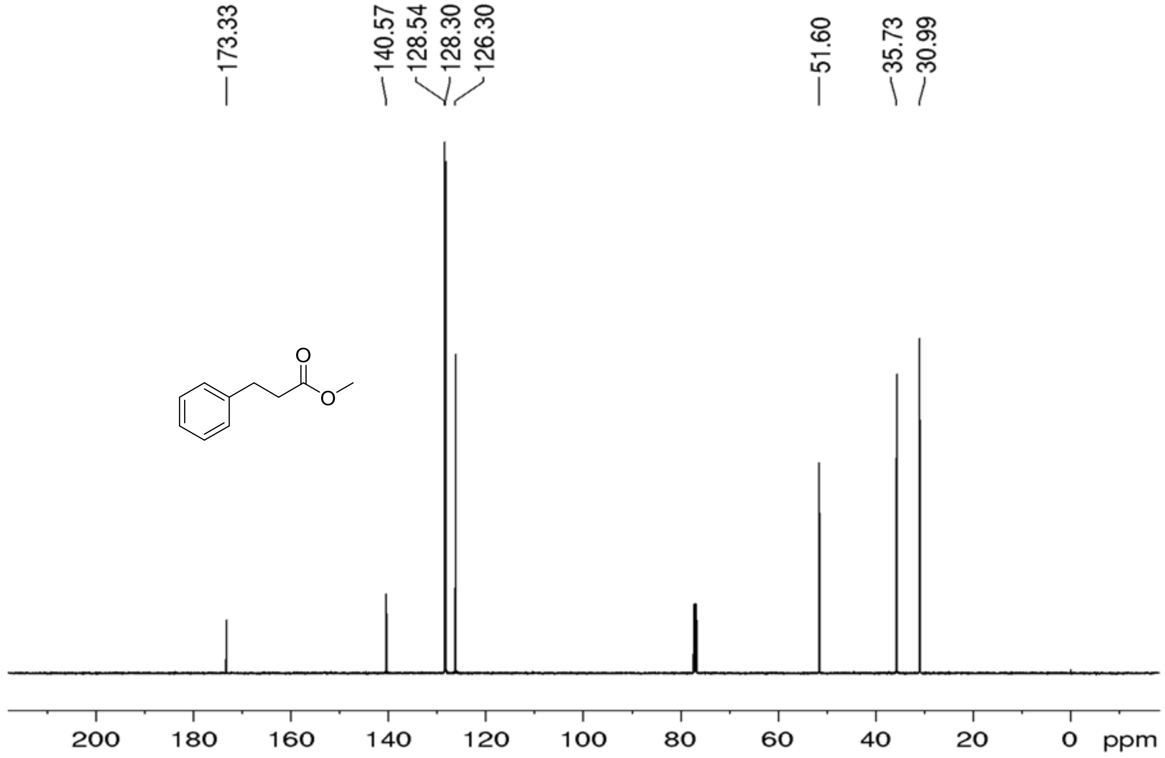

^{13}C NMR of compound **1**

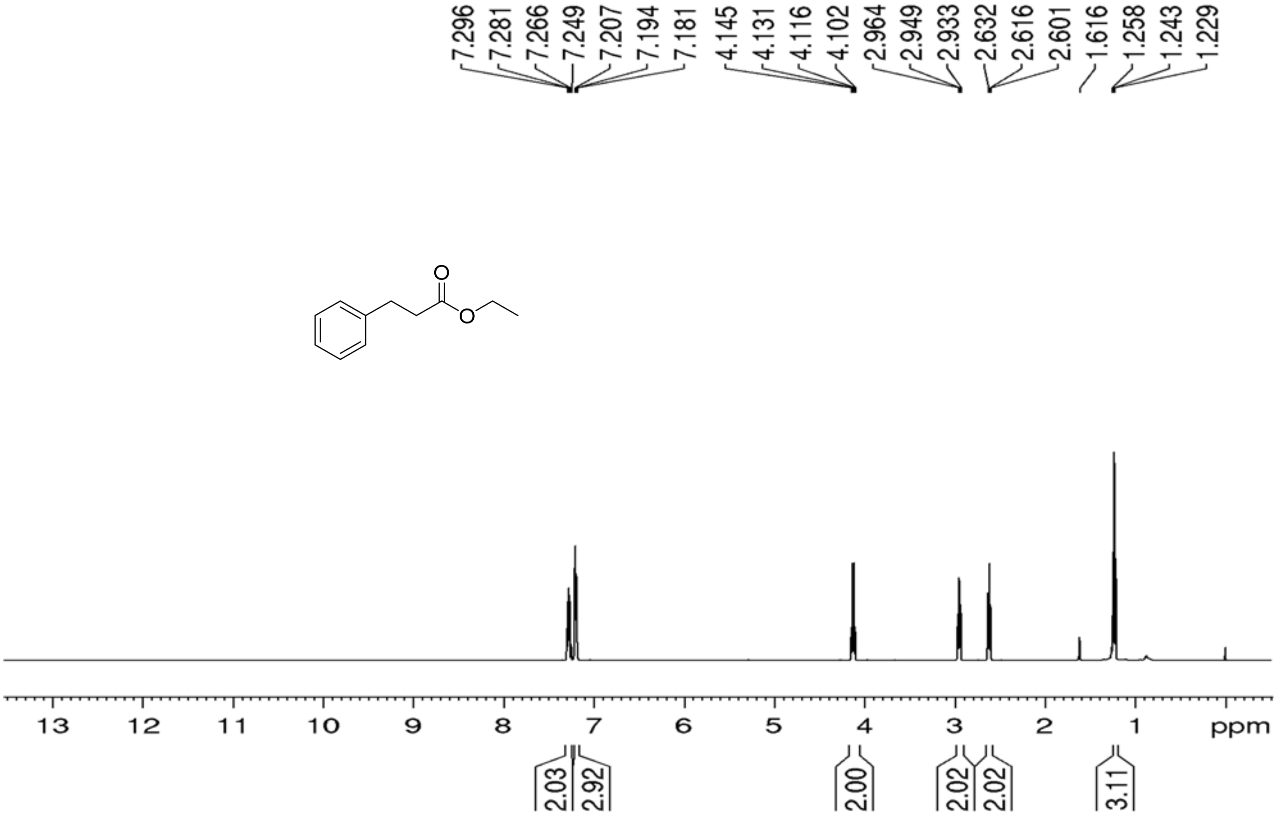

^{1}H NMR of compound **2**

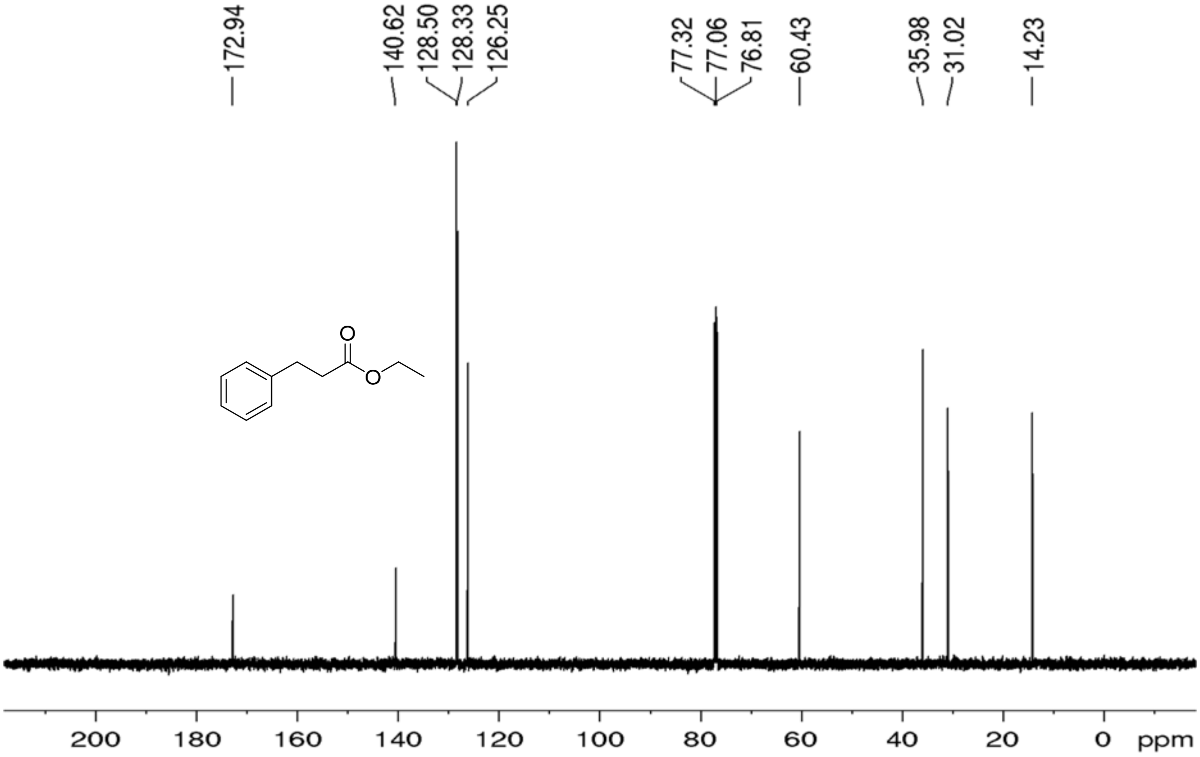

^{13}C NMR of compound **2**

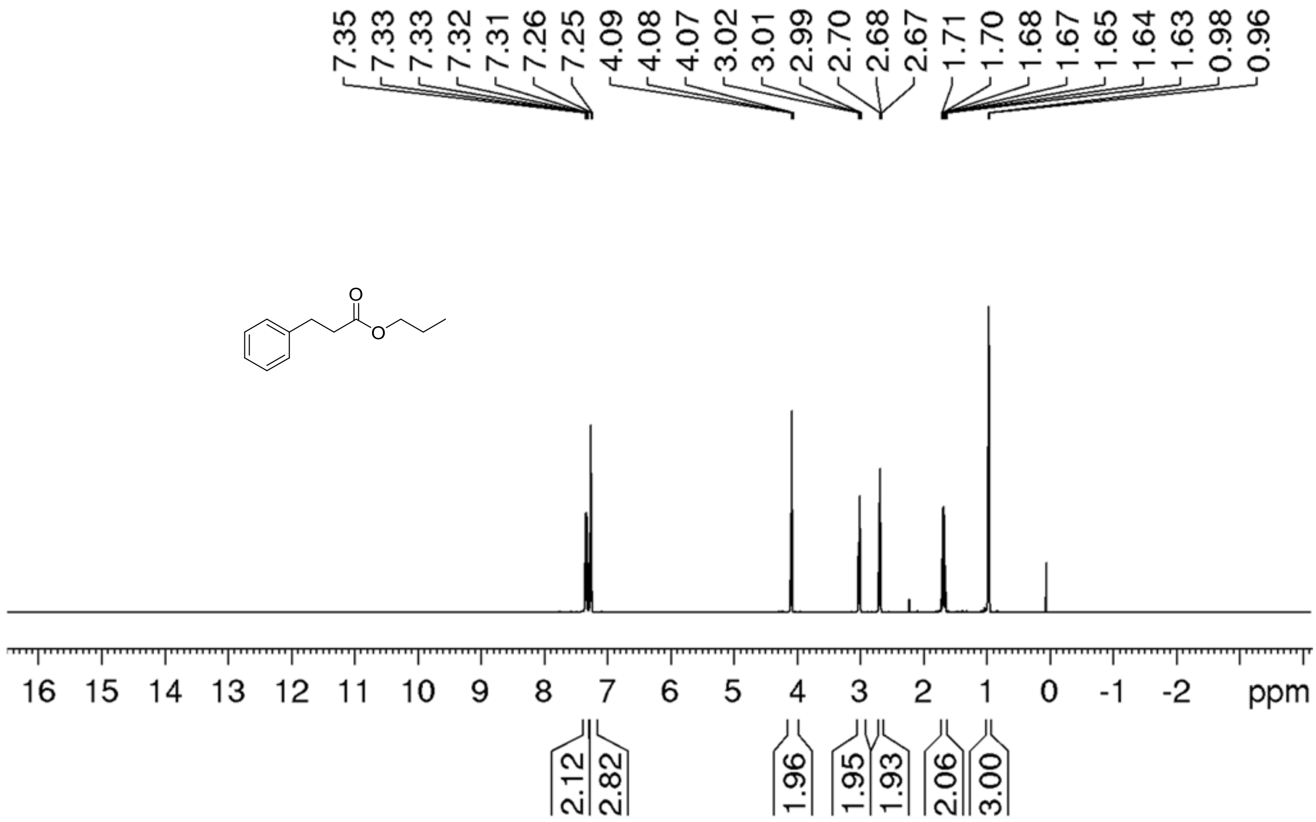

^{1}H NMR of compound **3**

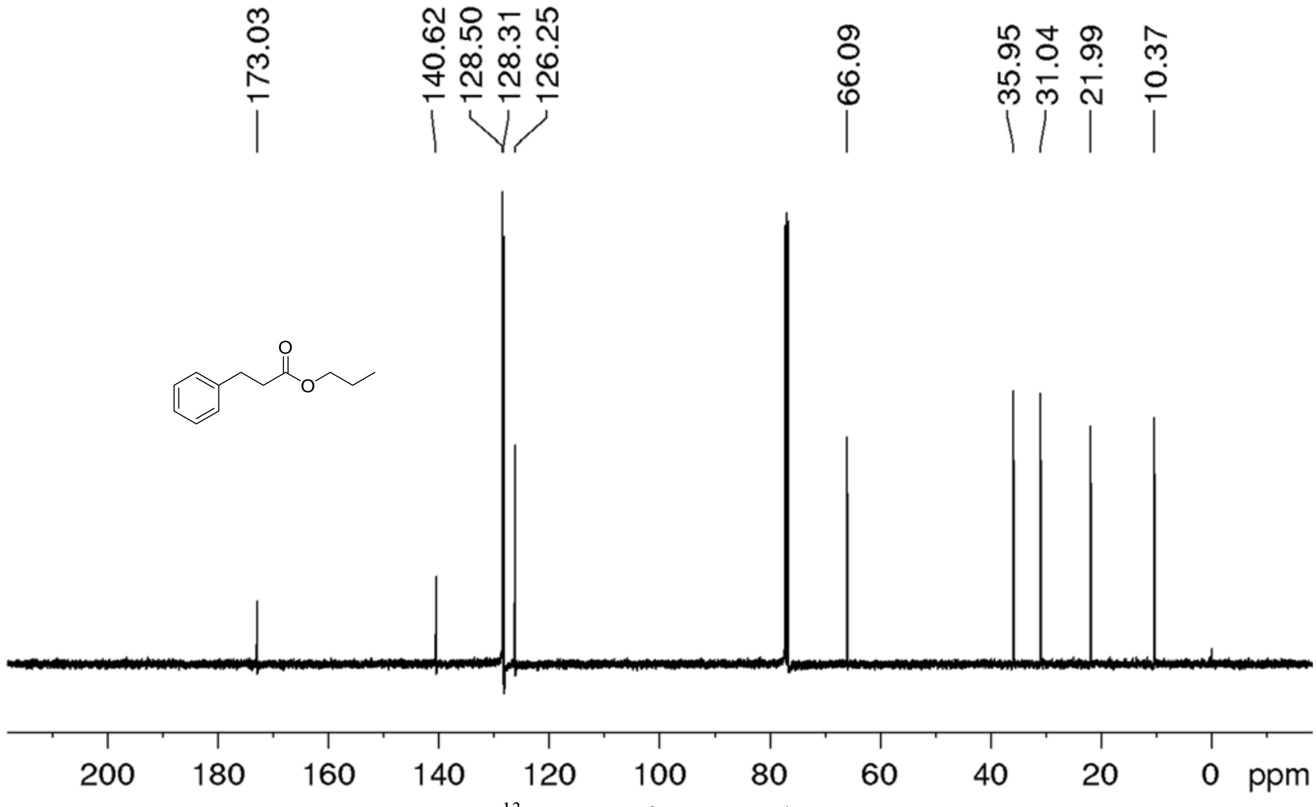

^{13}C NMR of compound **3**

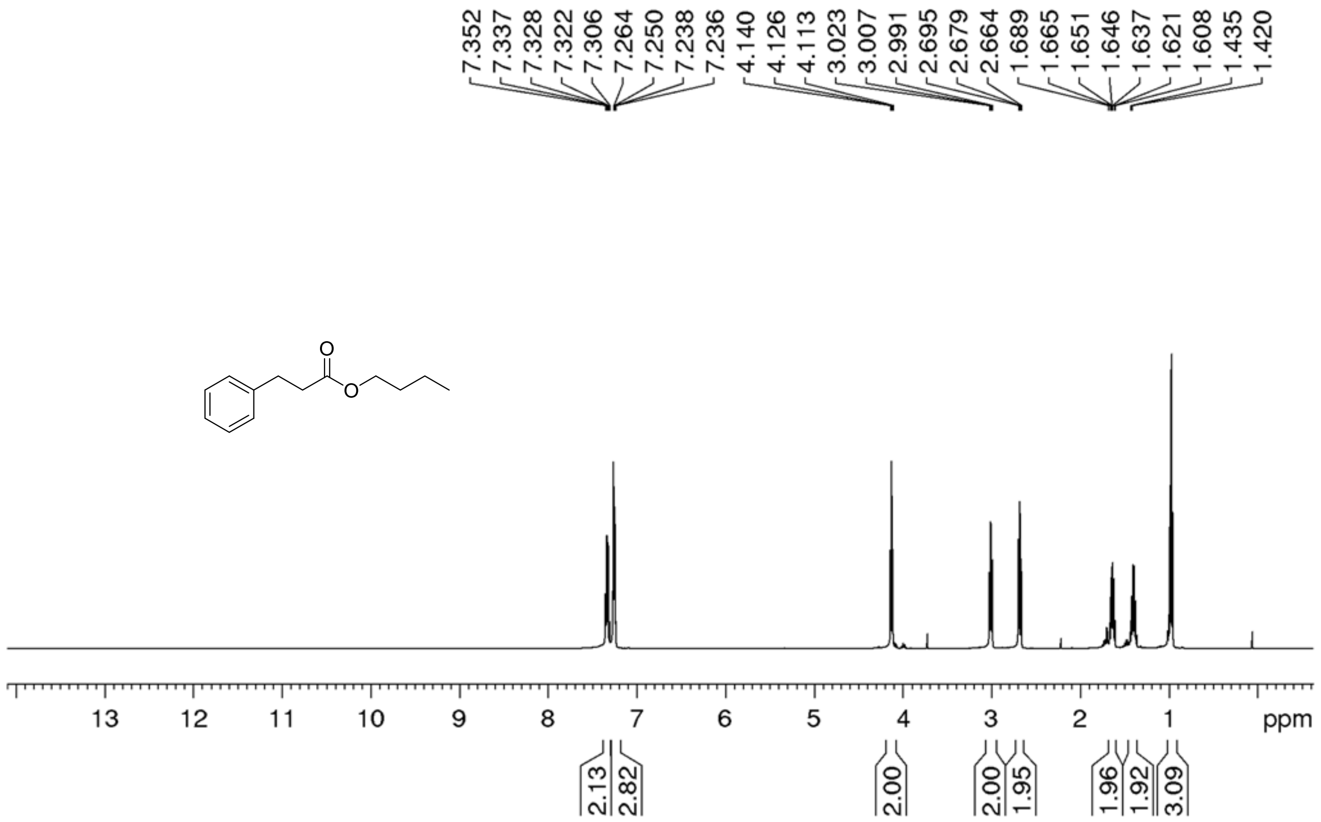

^{1}H NMR of compound **4**

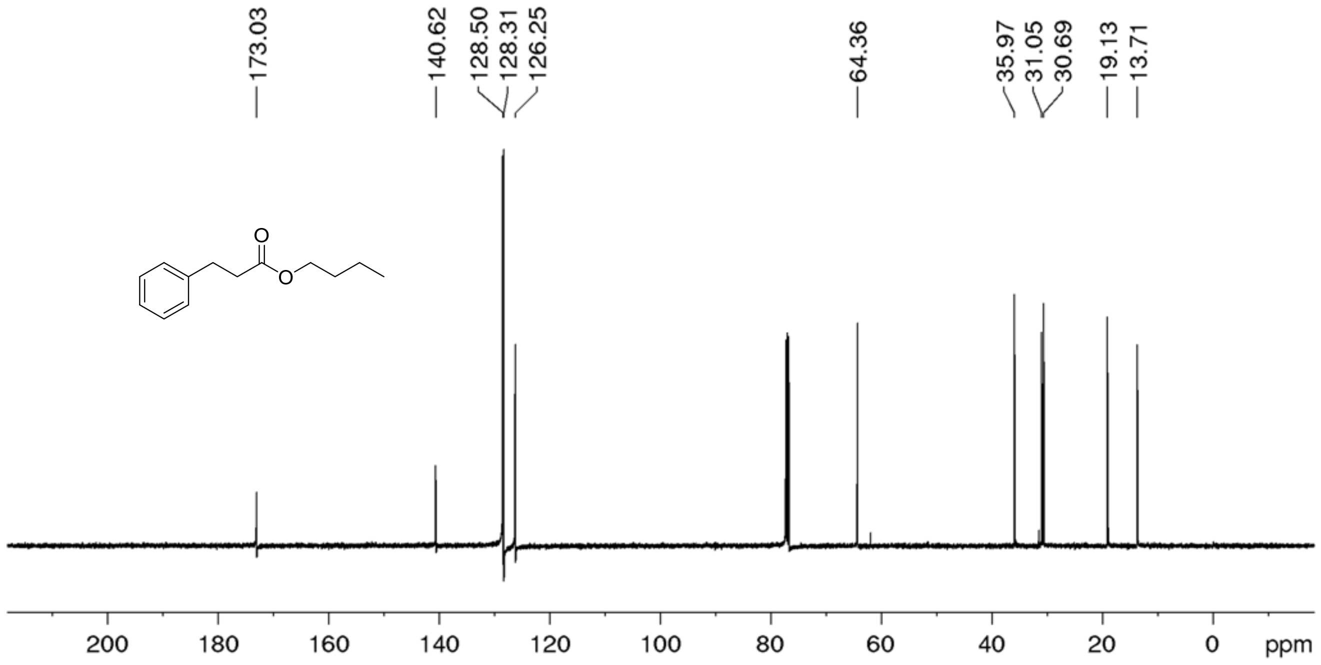

^{13}C NMR of compound **4**

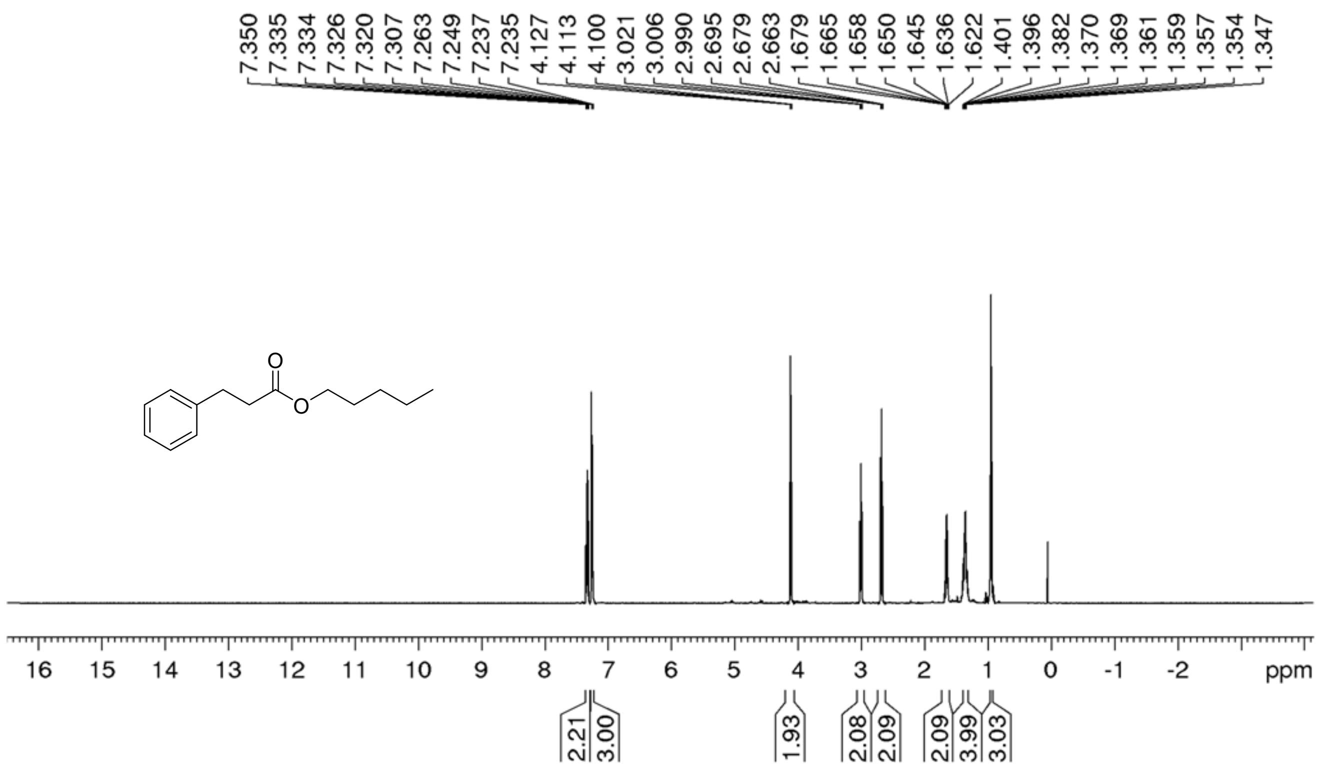

^{1}H NMR of compound **5**

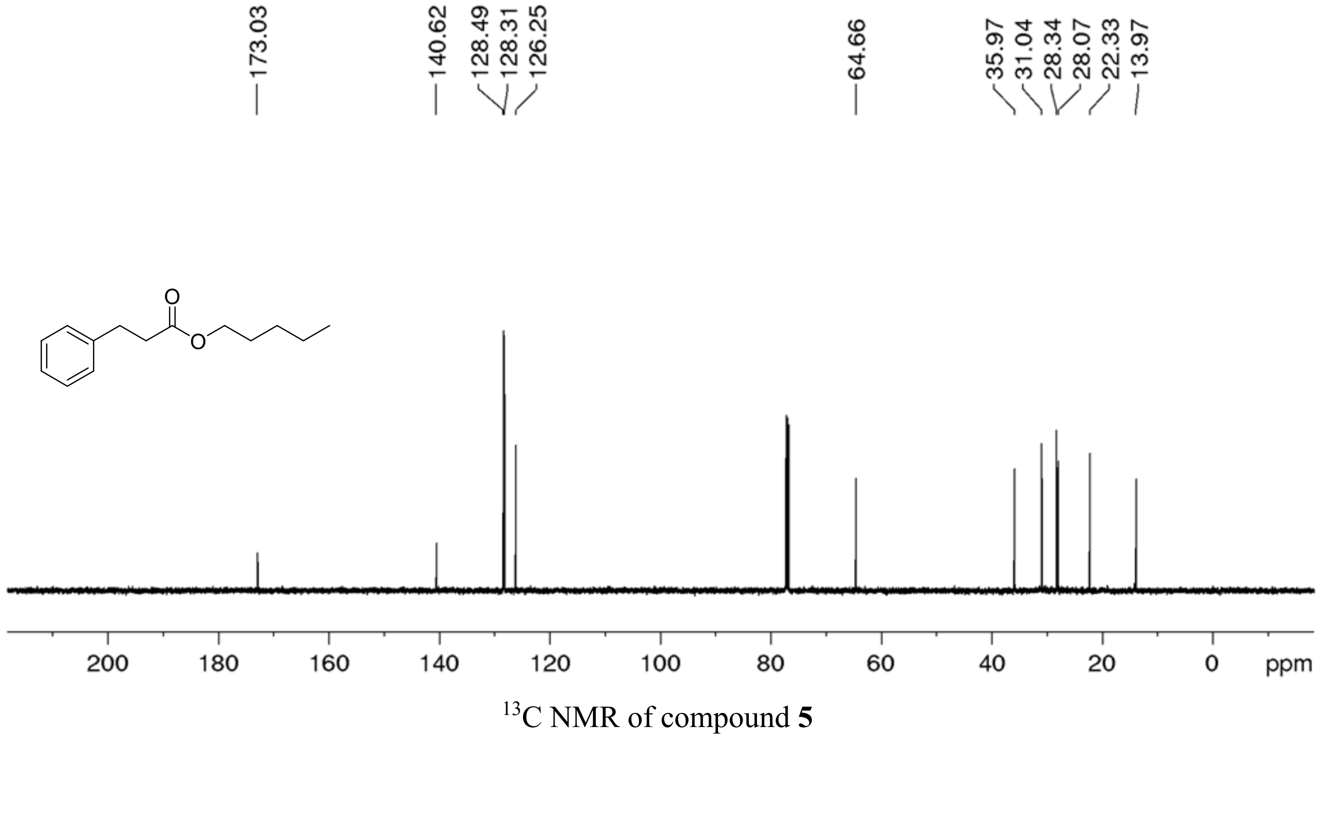

^{13}C NMR of compound **5**

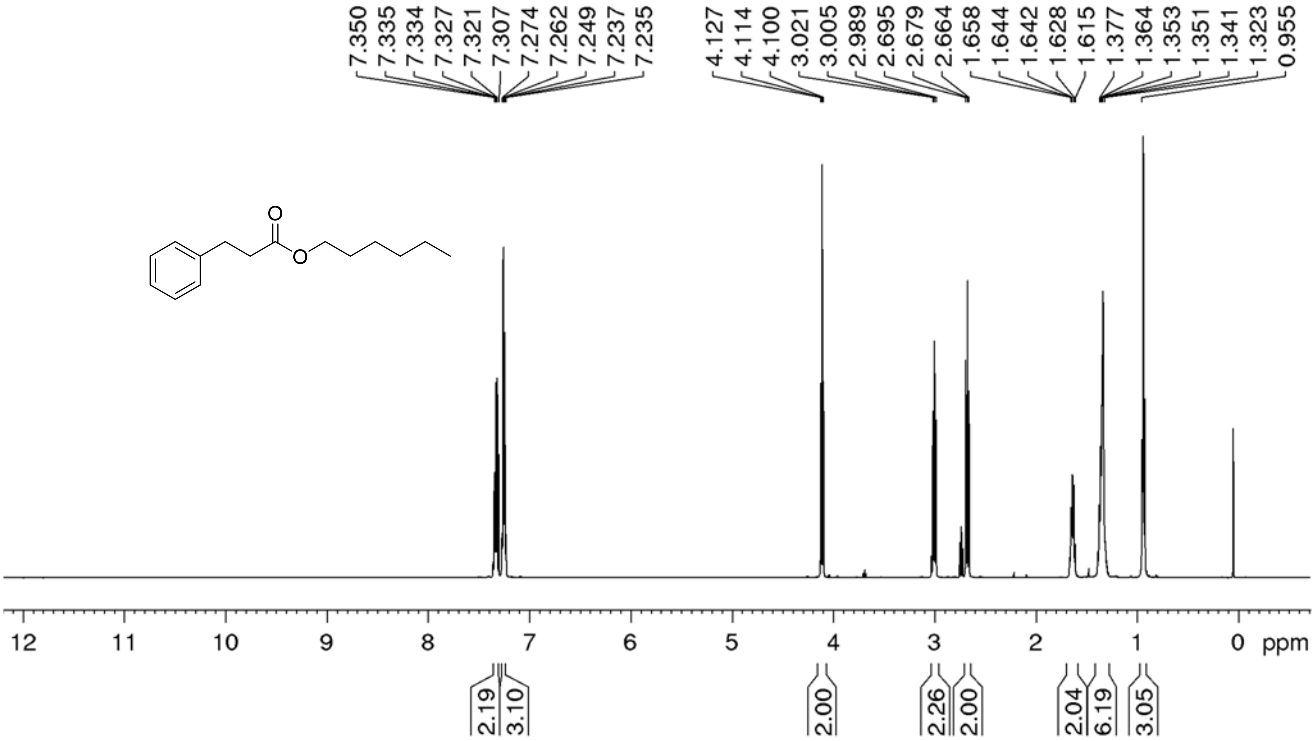

^{1}H NMR of compound **6**

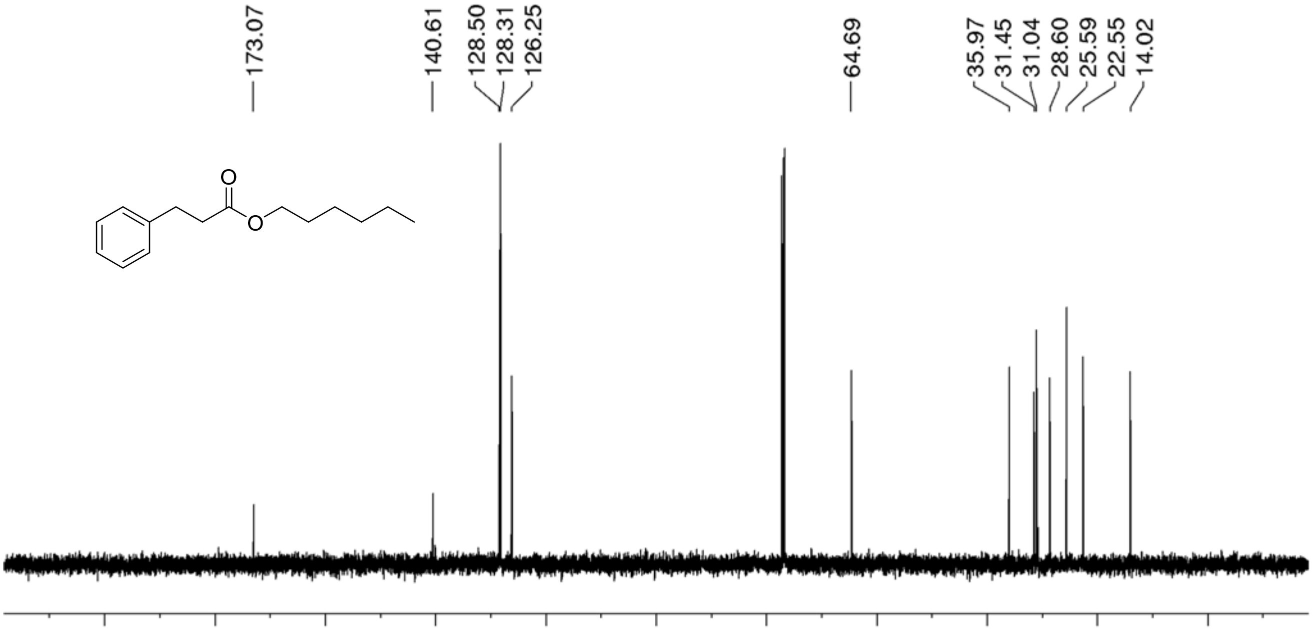

^{13}C NMR of compound **6**

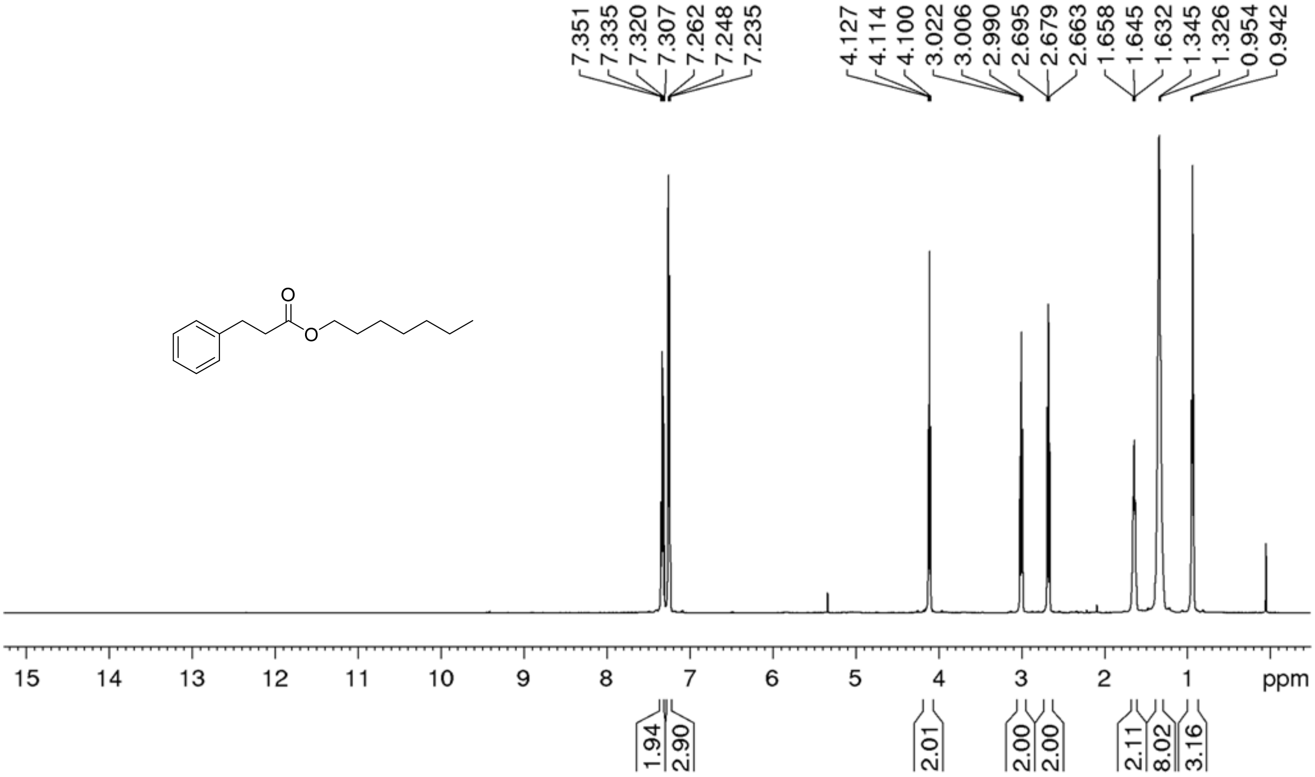

^{1}H NMR of compound **7**

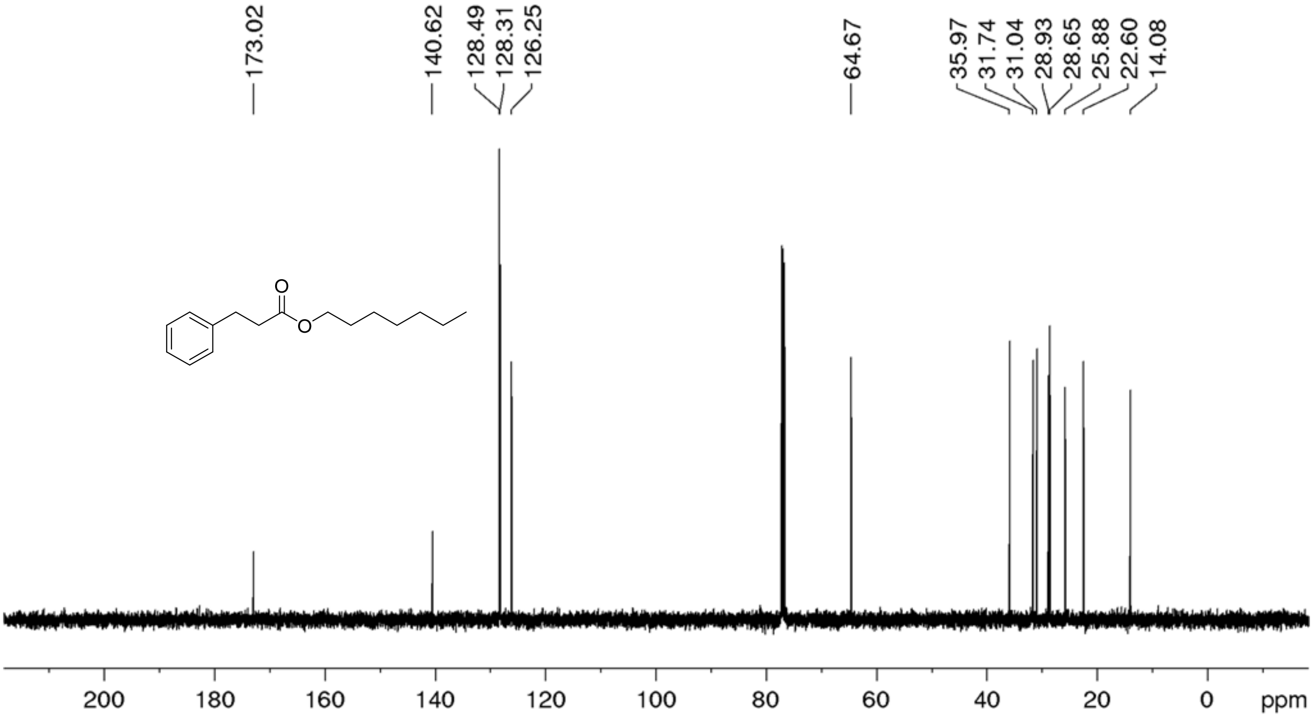

^{13}C NMR of compound **7**

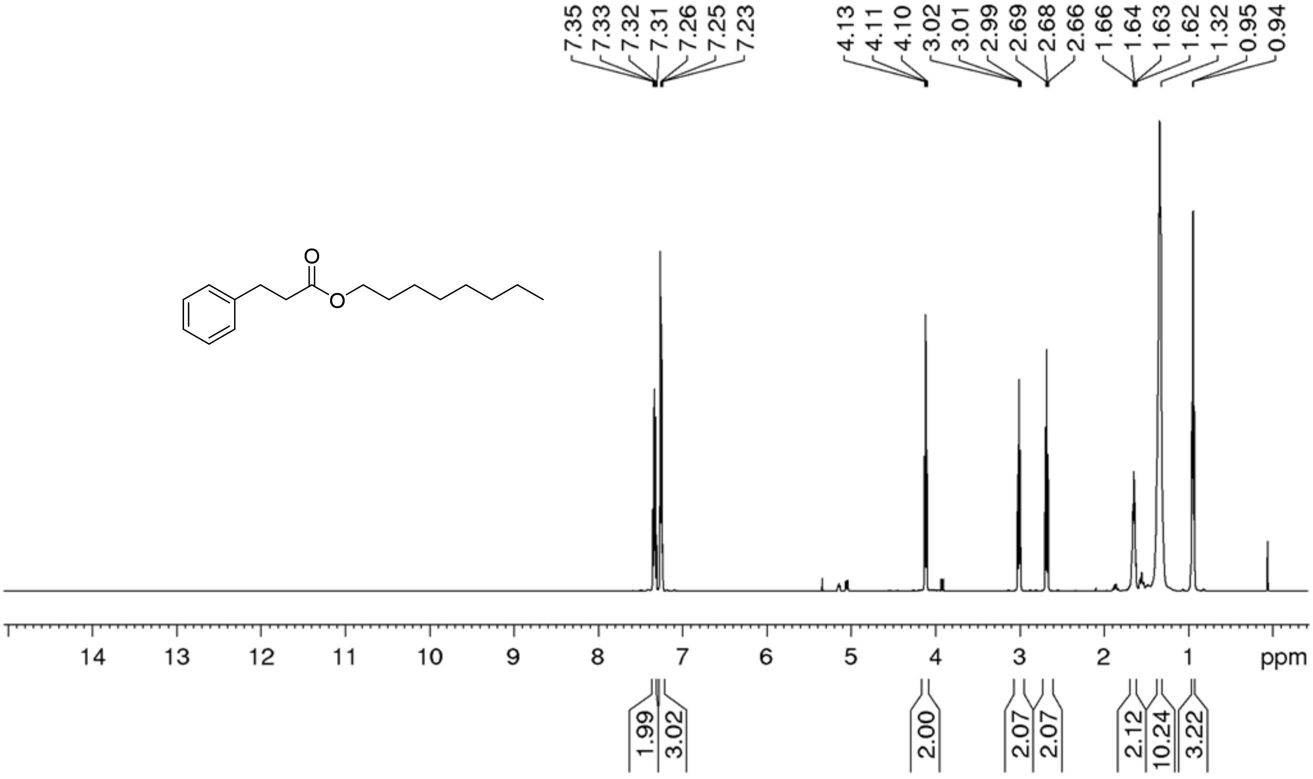

^{1}H NMR of compound **8**

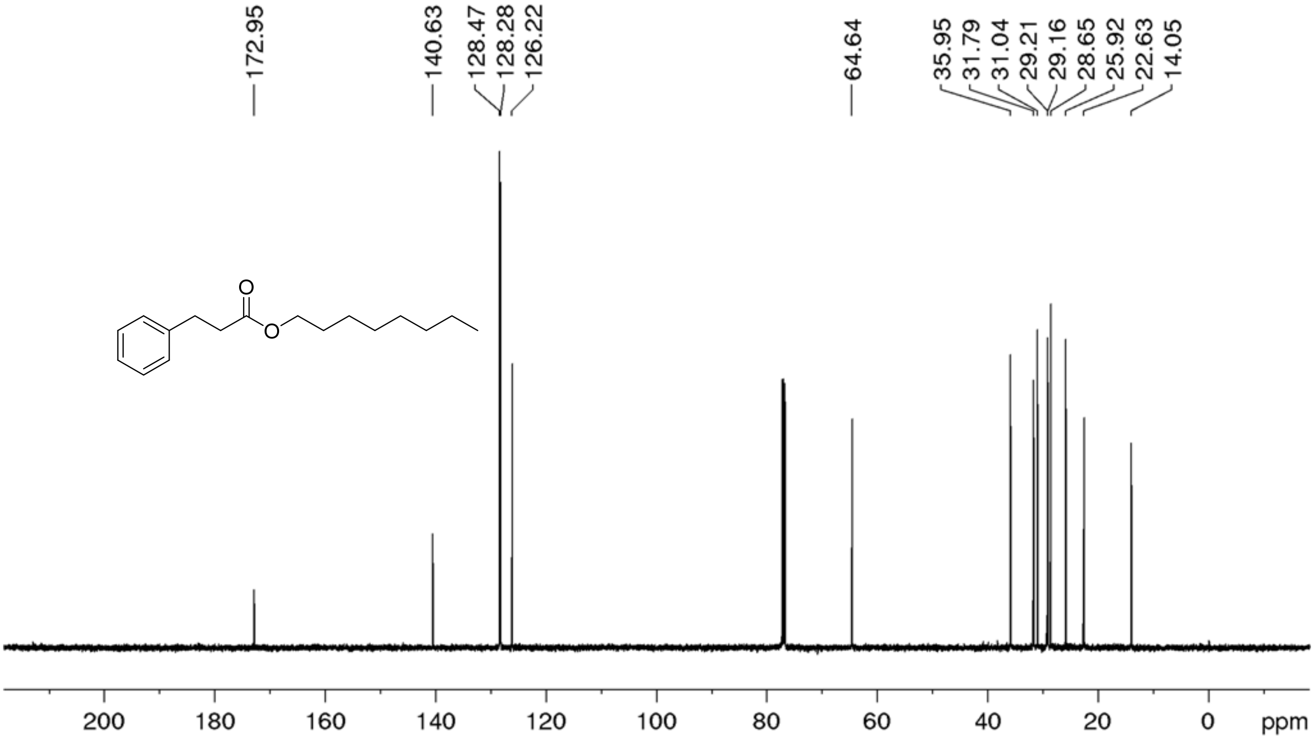

^{13}C NMR of compound **8**

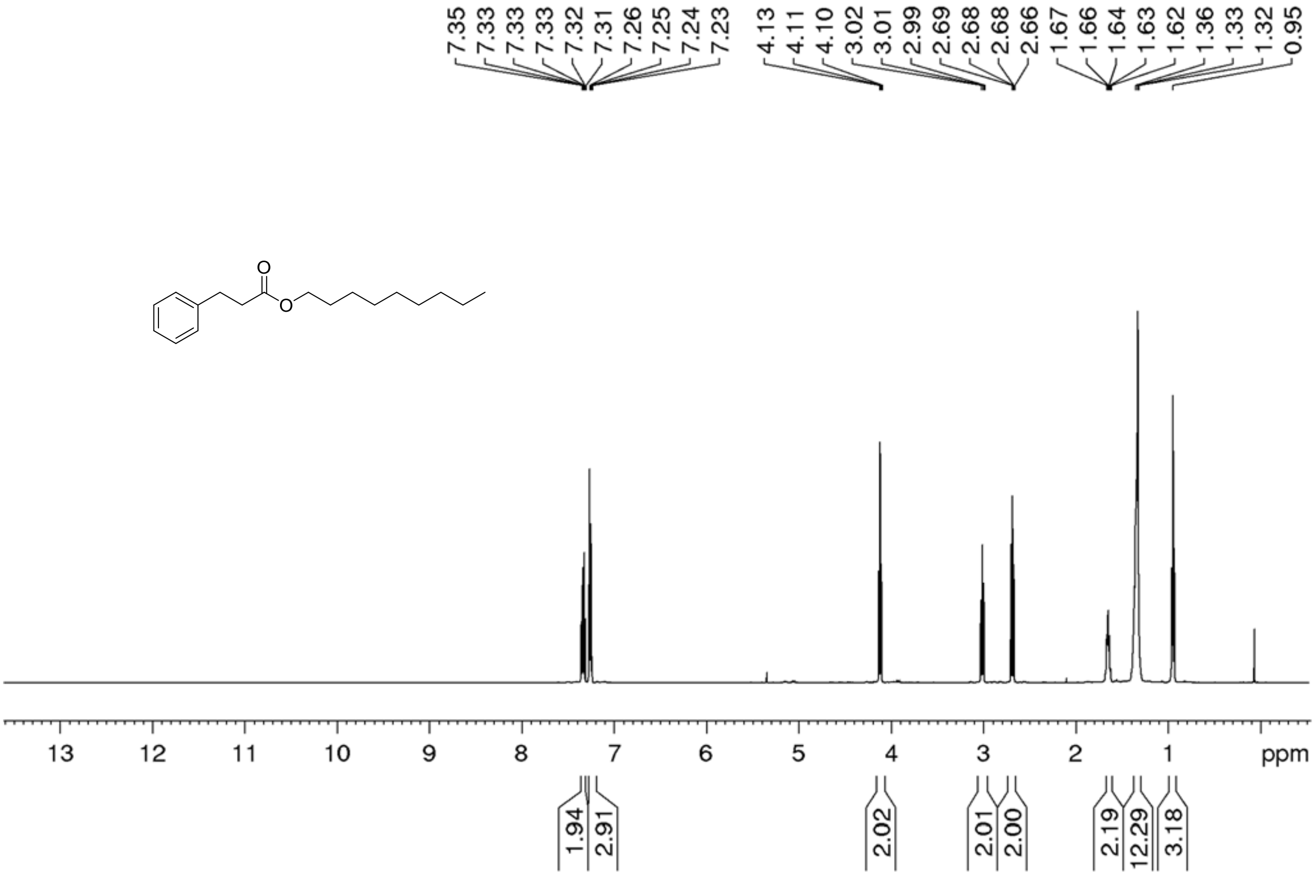

^{1}H NMR of compound **9**

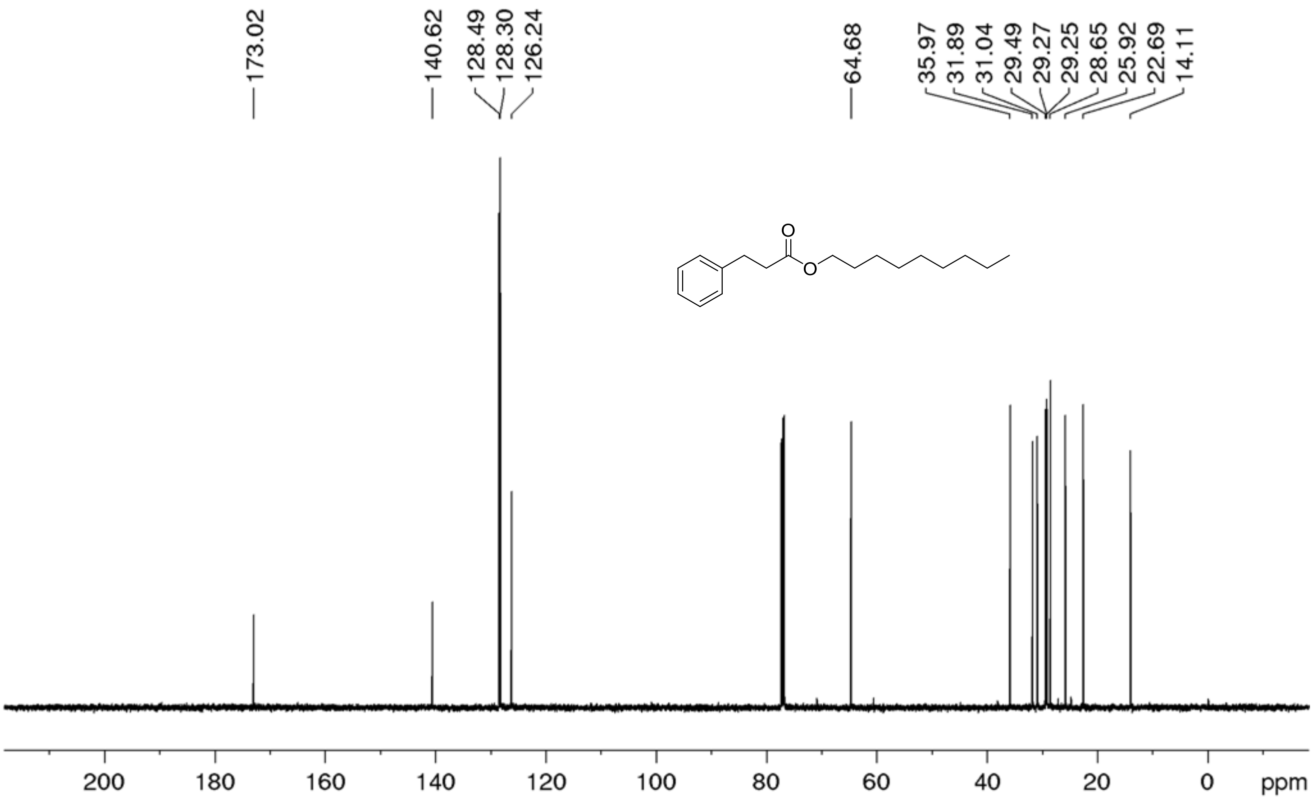

^{13}C NMR of compound **9**

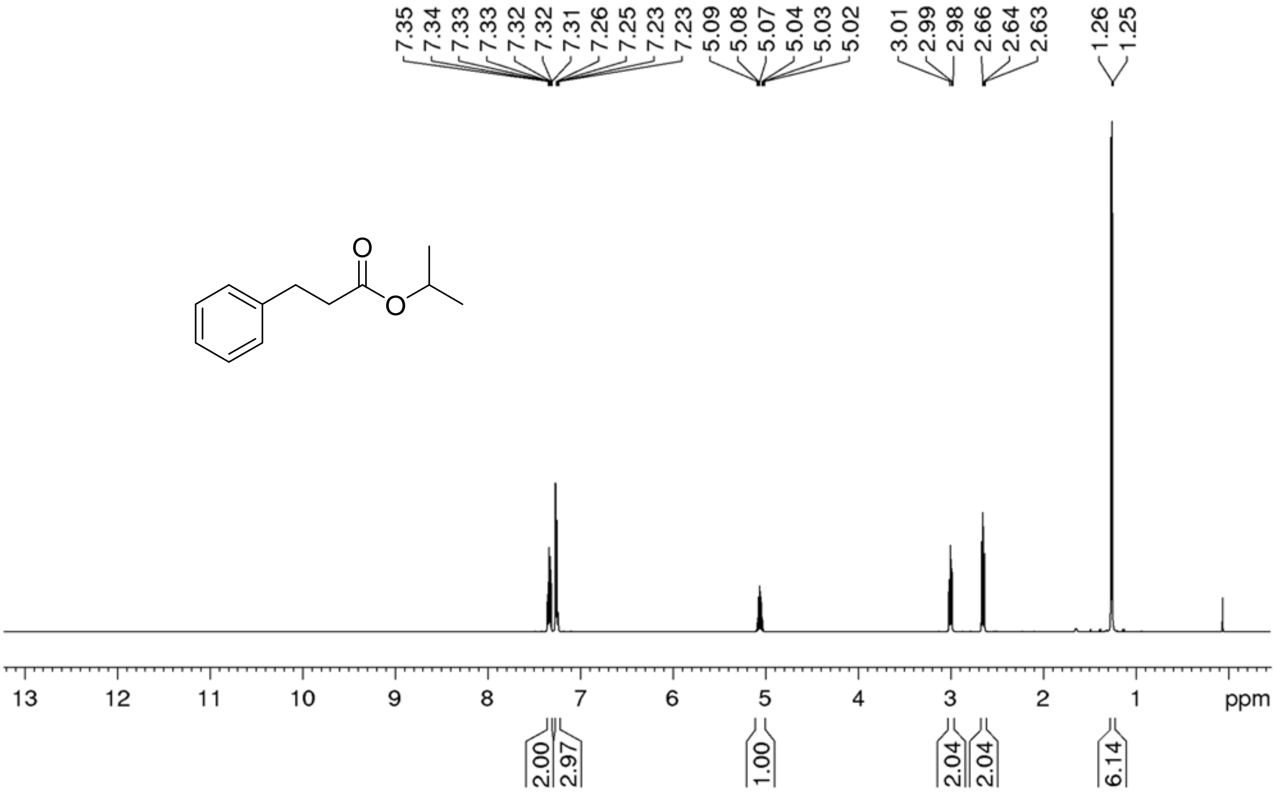

^{1}H NMR of compound **10**

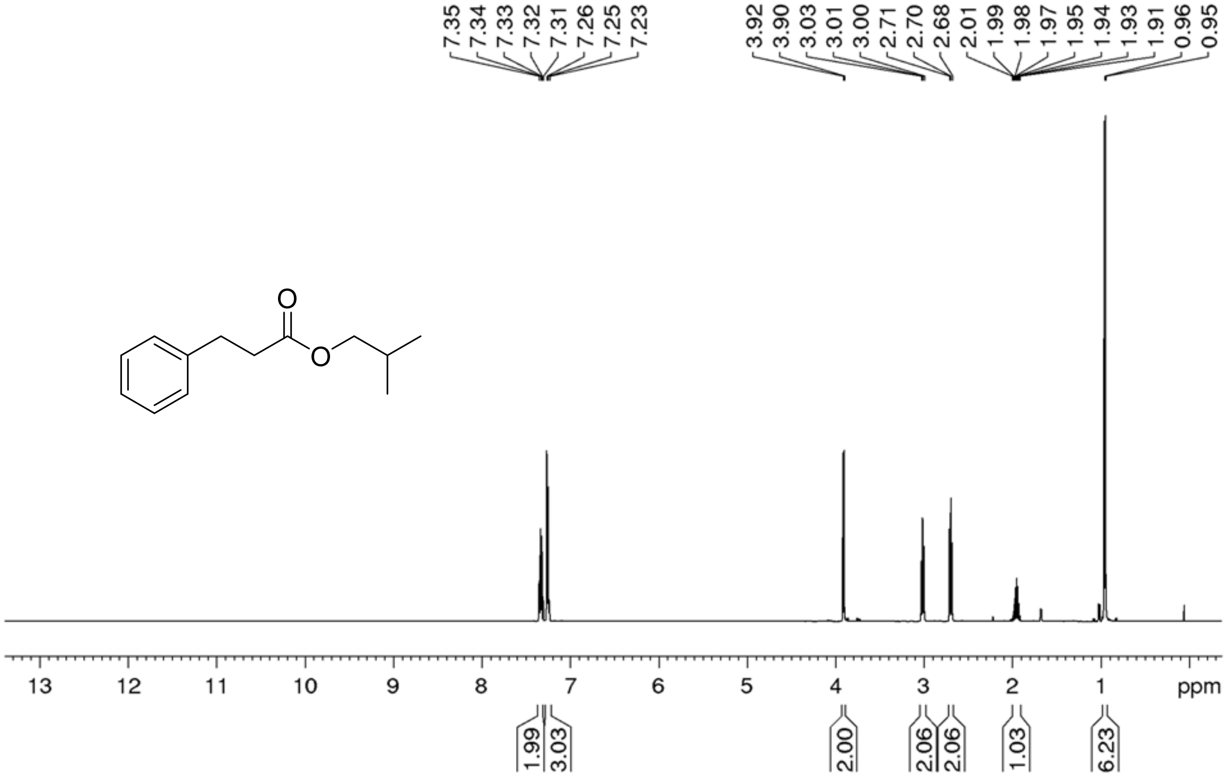

^{1}H NMR of compound **11**

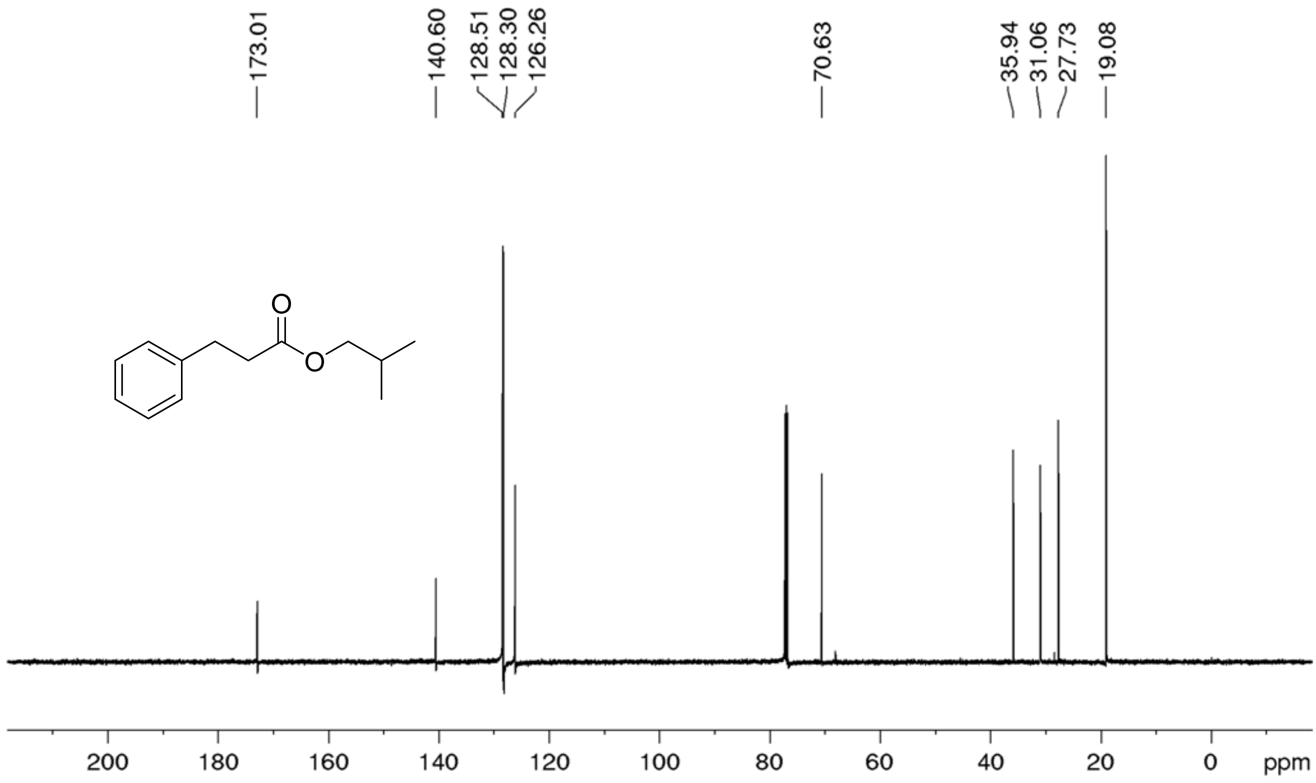

^{13}C NMR of compound **11**

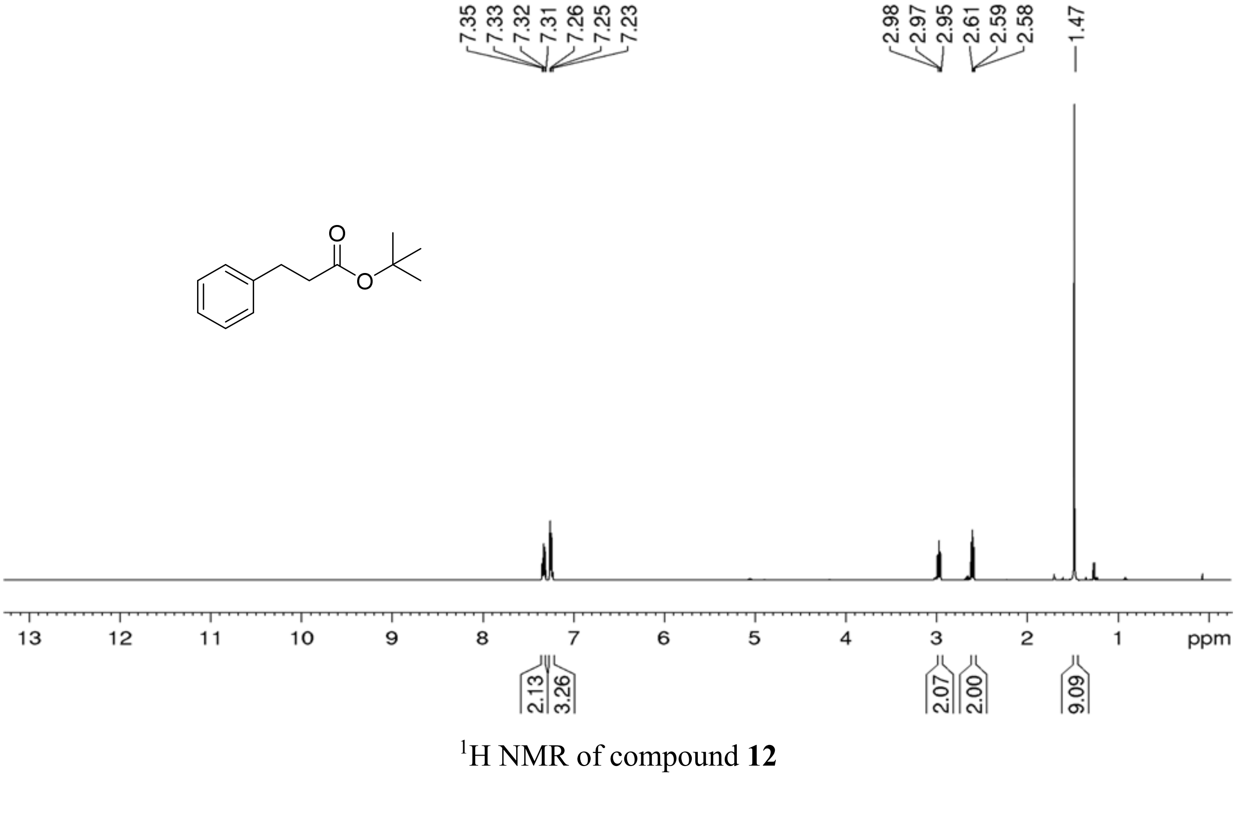

^{1}H NMR of compound **12**

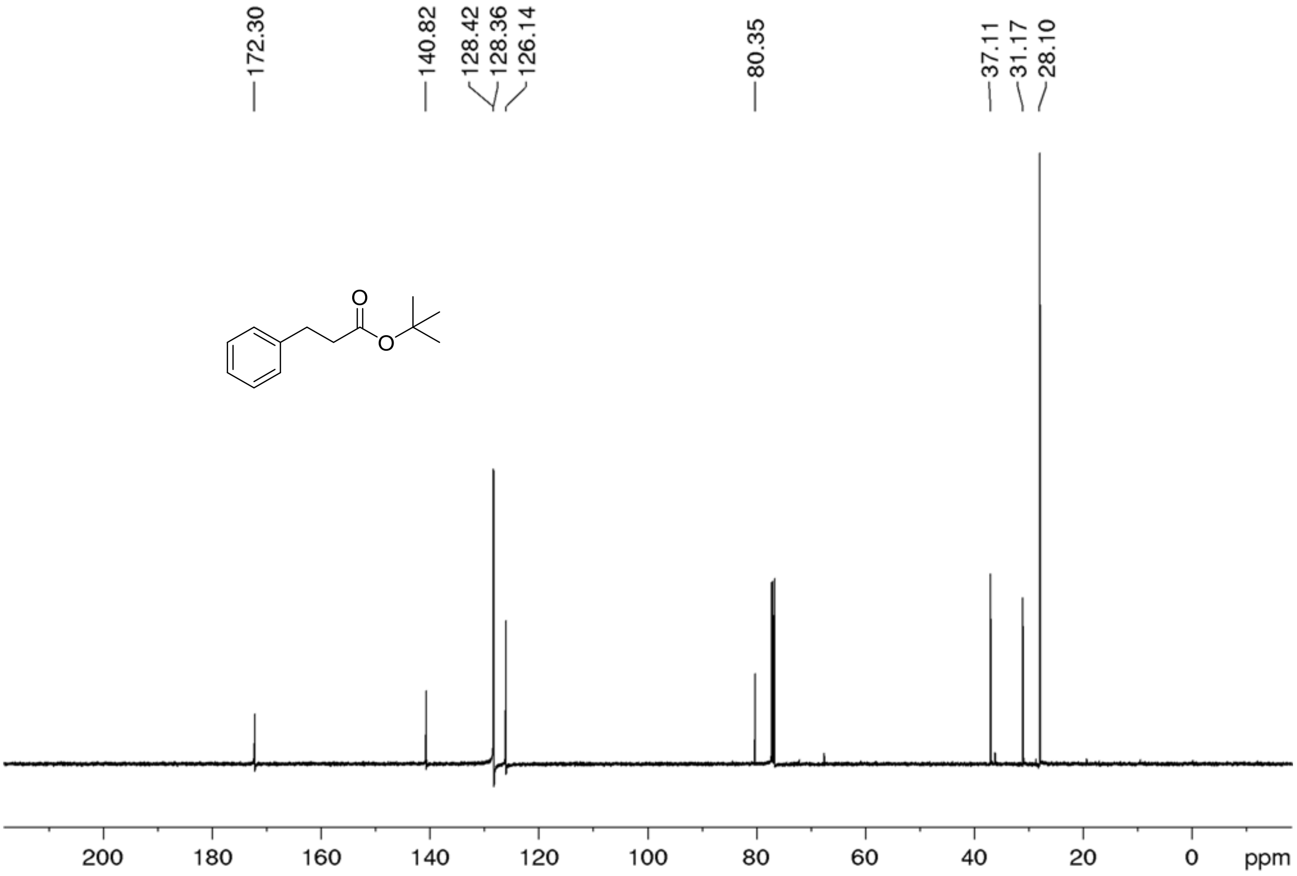

^{13}C NMR of compound **12**

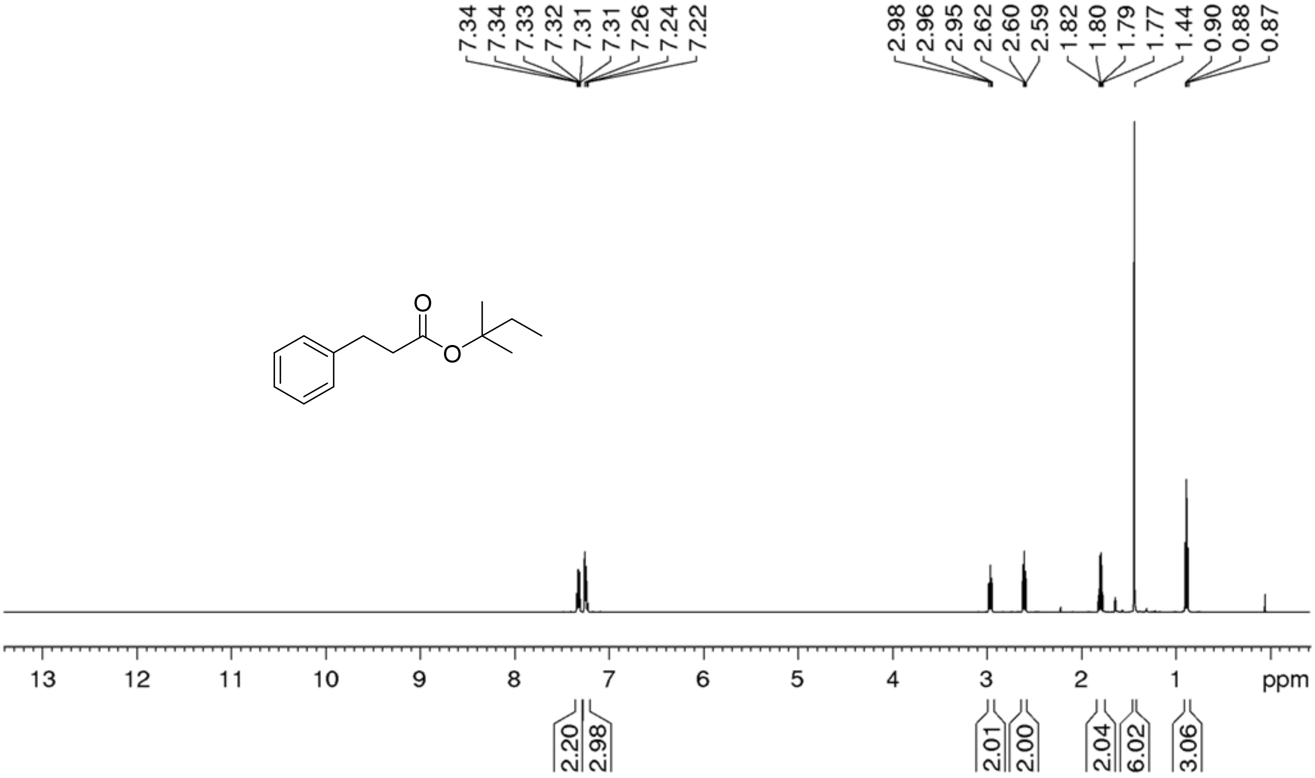

^{1}H NMR of compound **13**

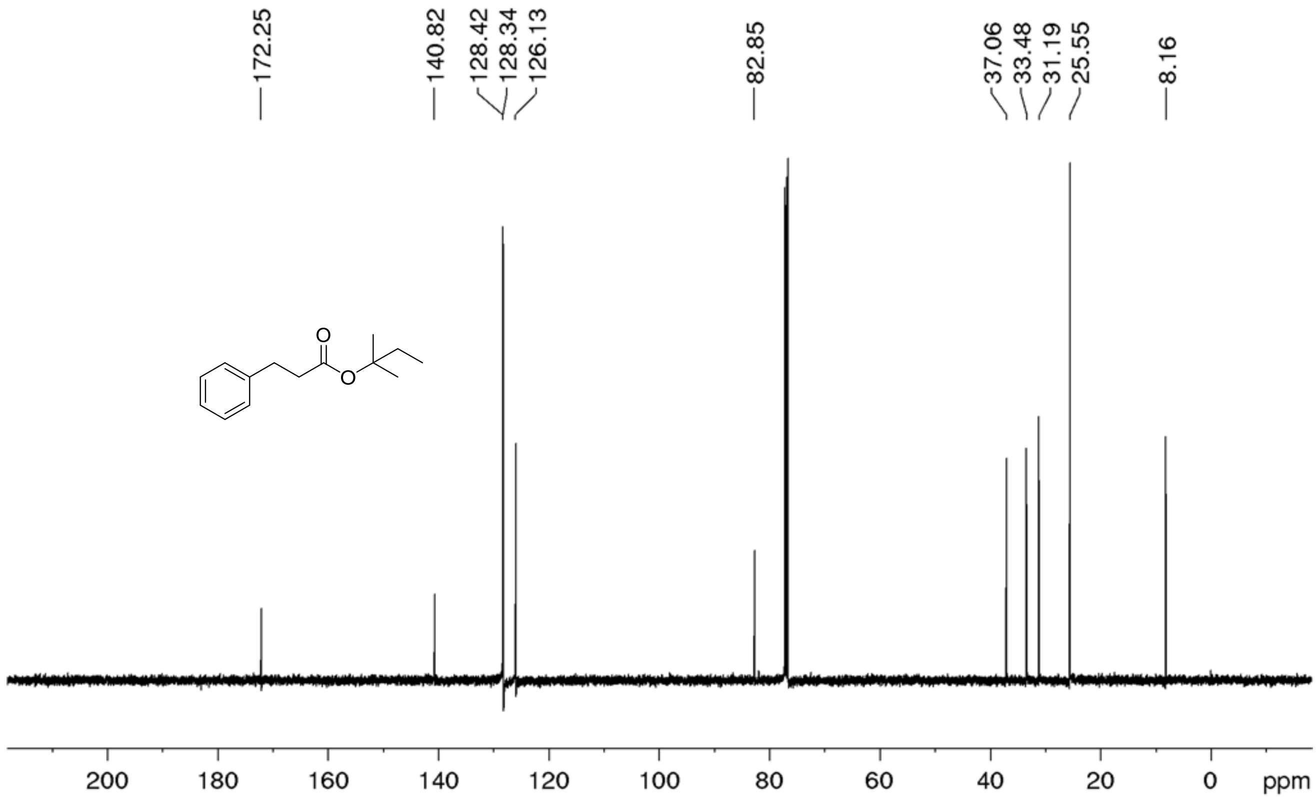

^{13}C NMR of compound **13**

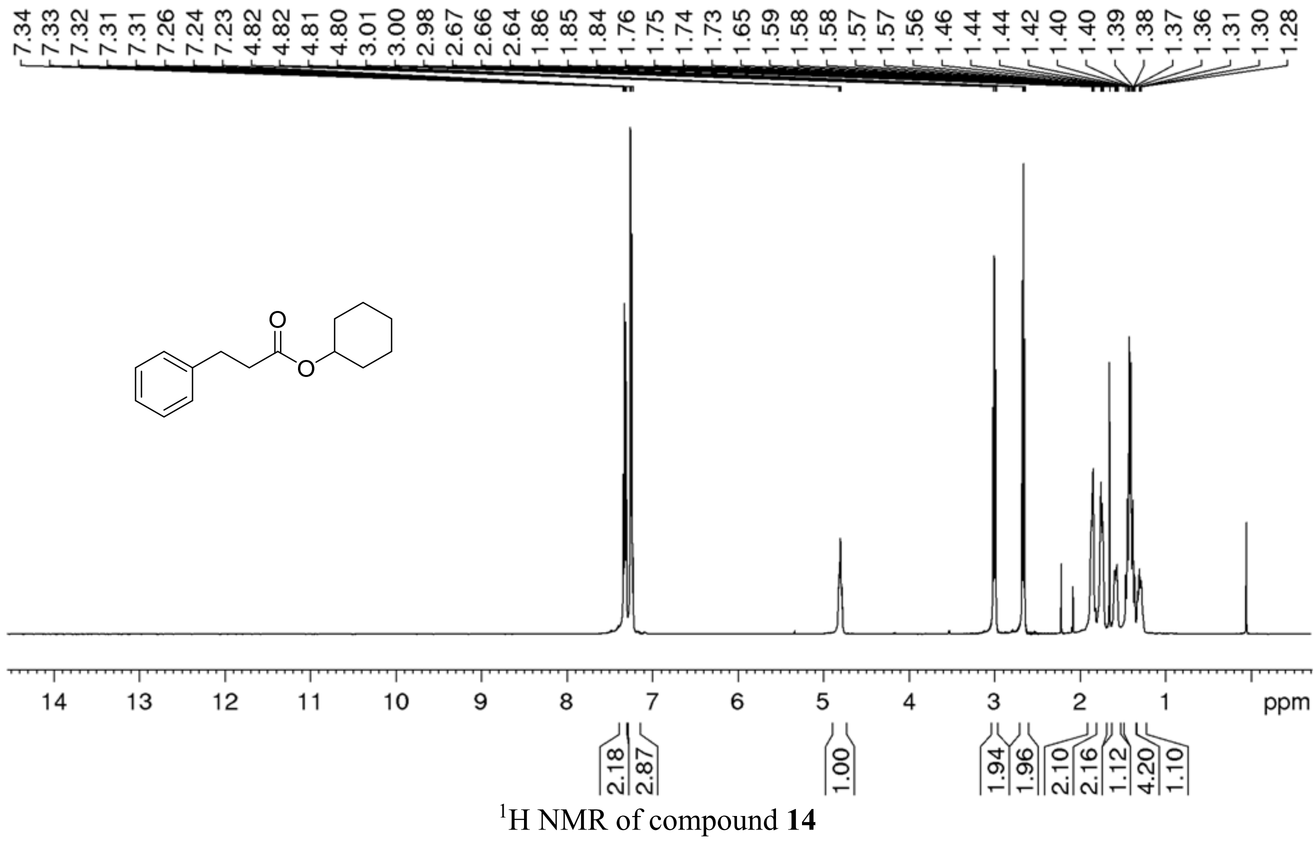

^{1}H NMR of compound **14**

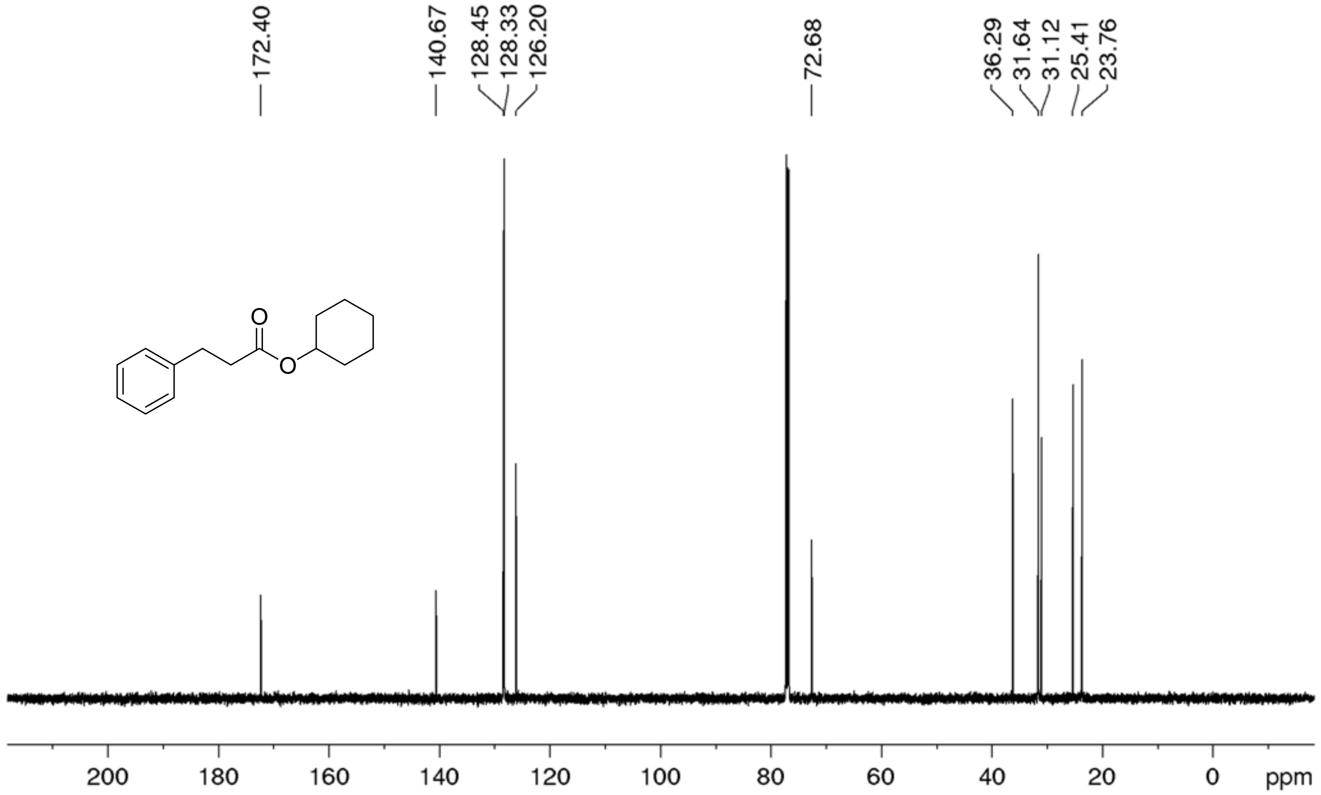

^{13}C NMR of compound **14**

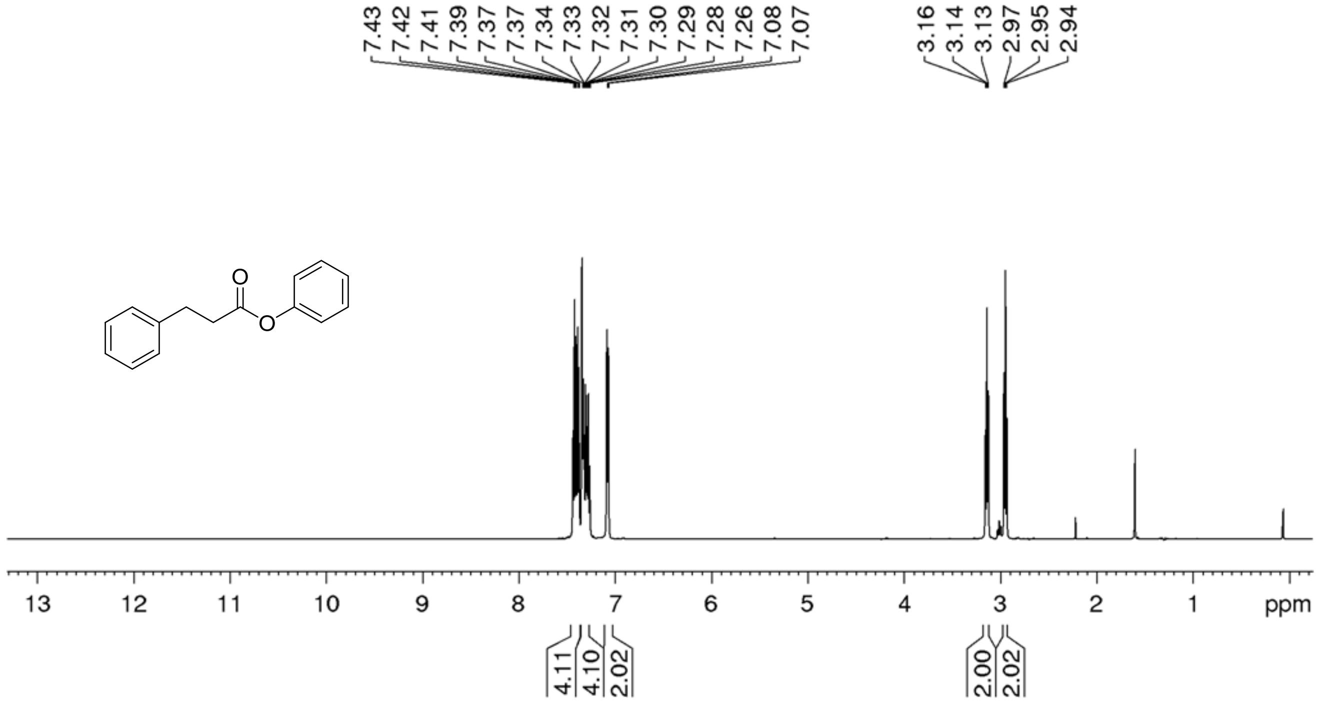

^{1}H NMR of compound **15**

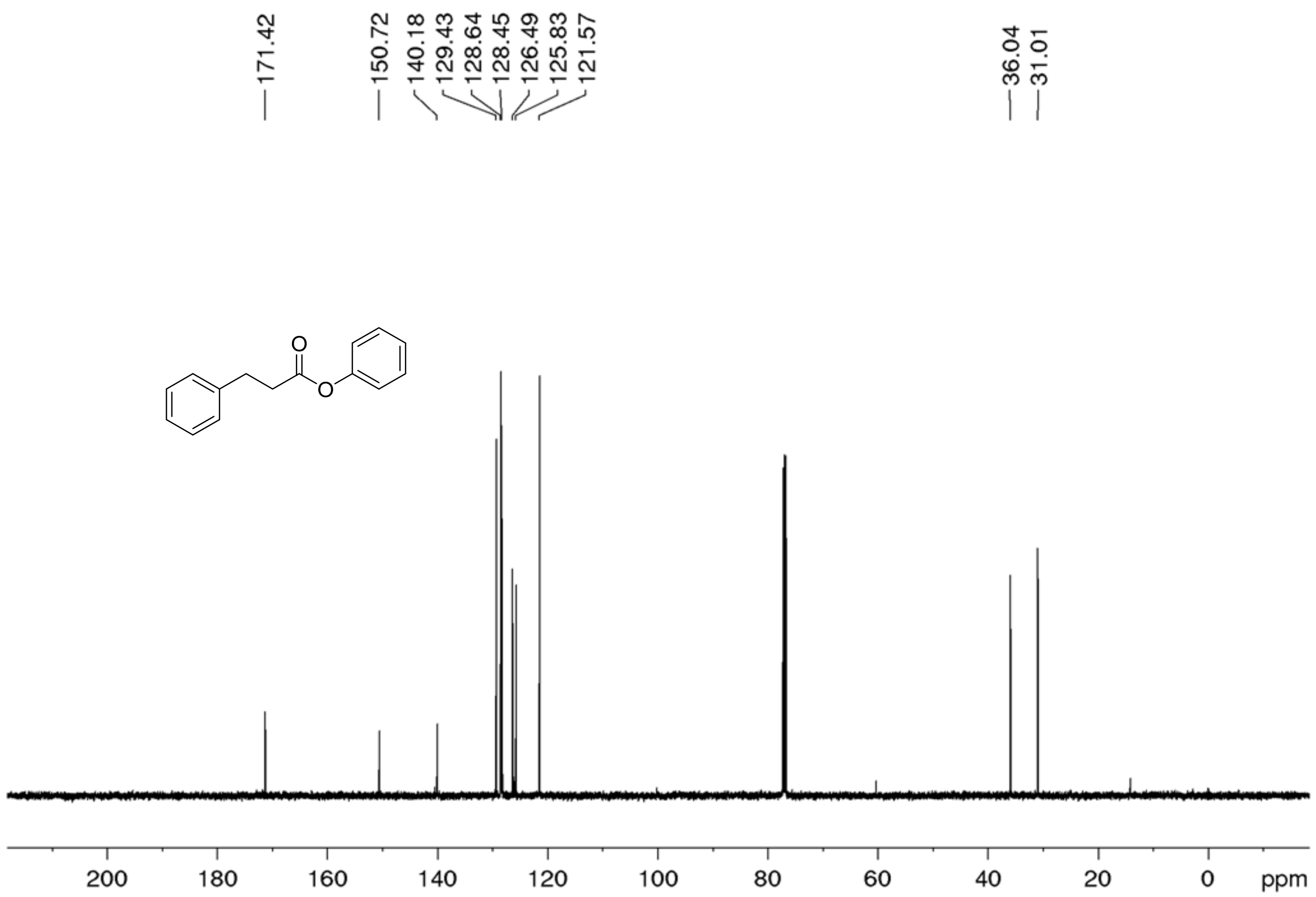

^{13}C NMR of compound **15**

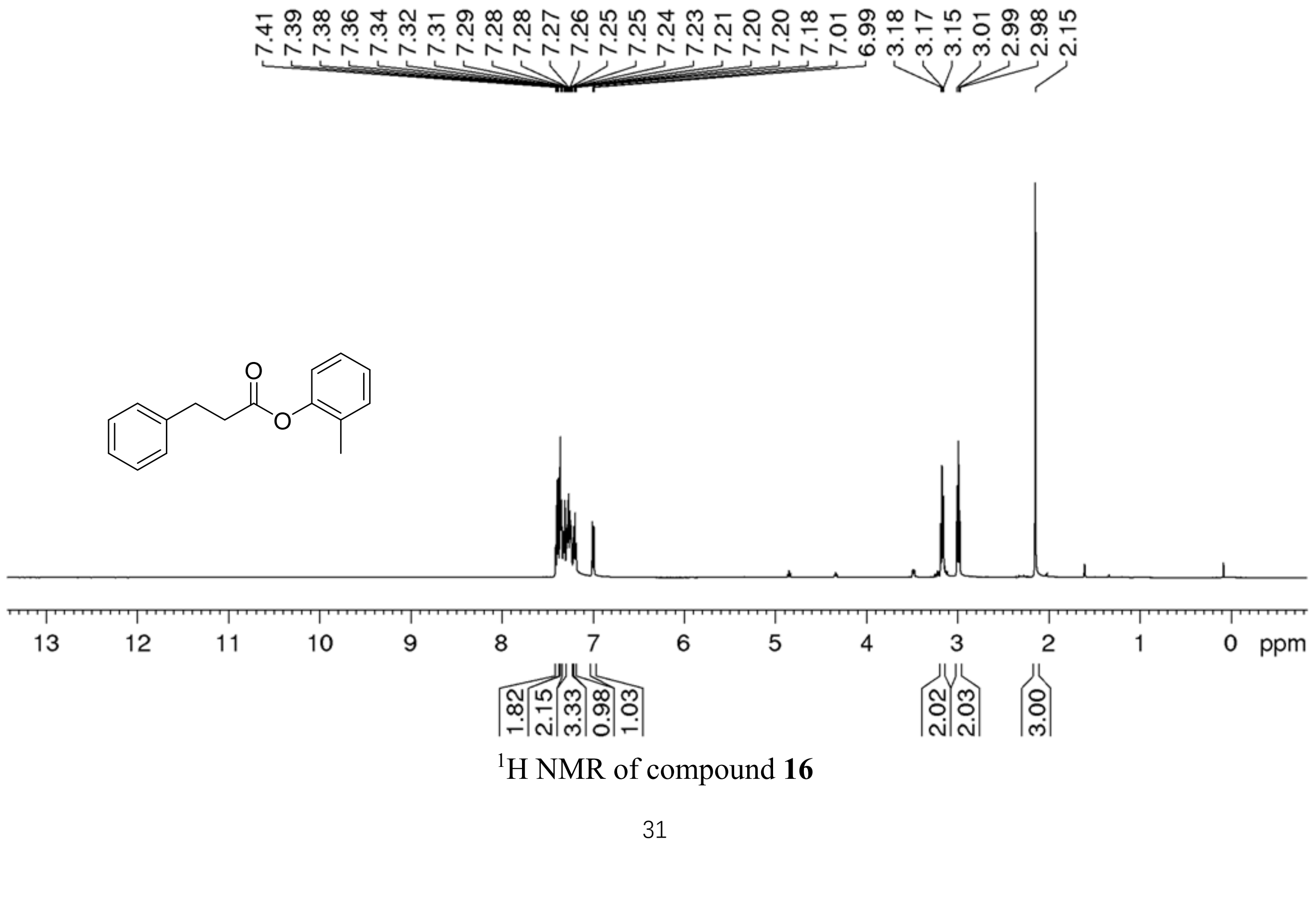

^{1}H NMR of compound **16**

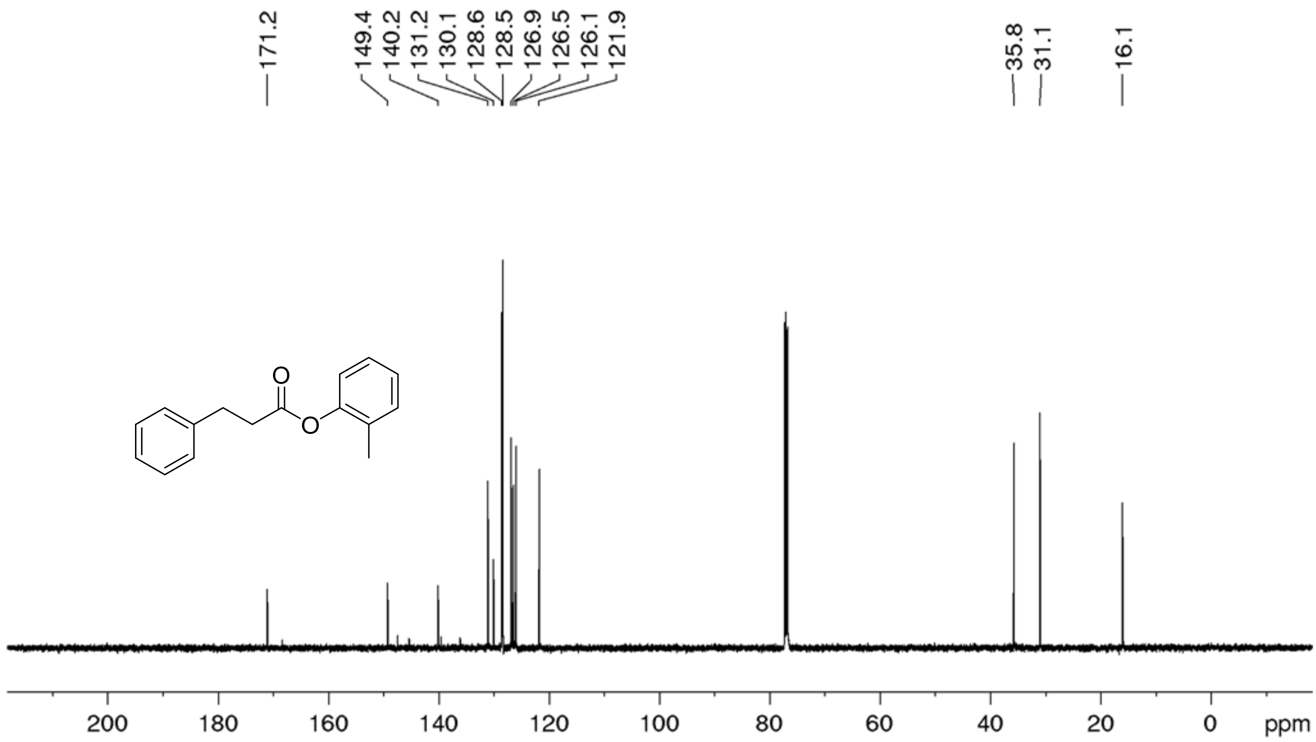

^{13}C NMR of compound **16**

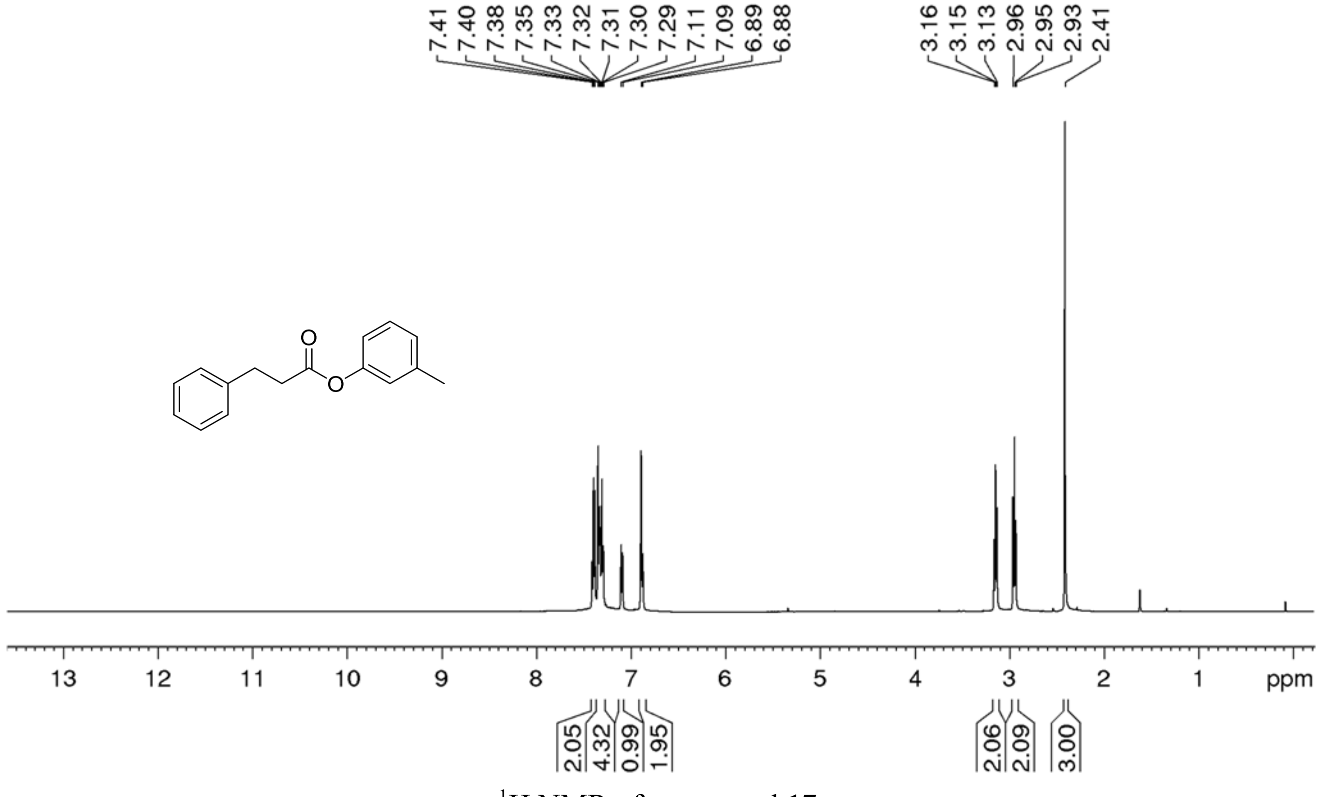

^{1}H NMR of compound **17**

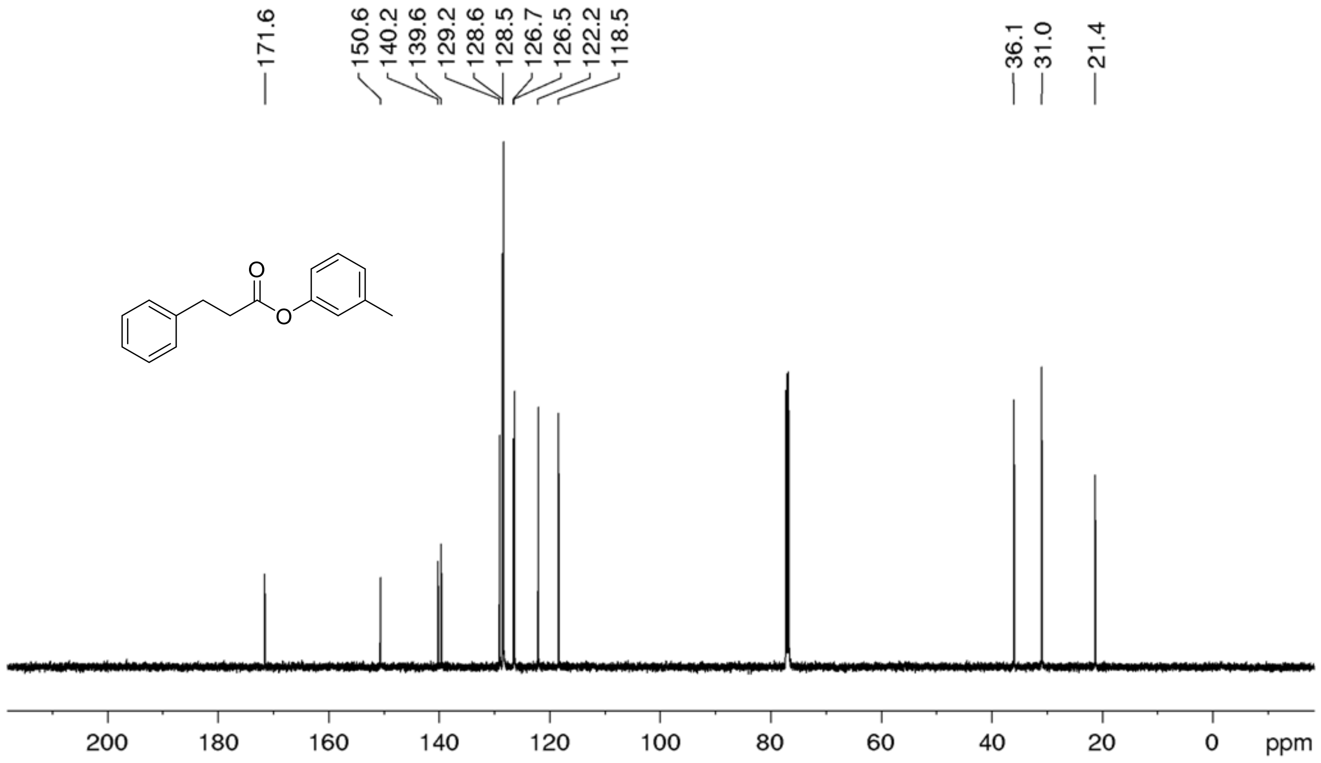

^{13}C NMR of compound **17**

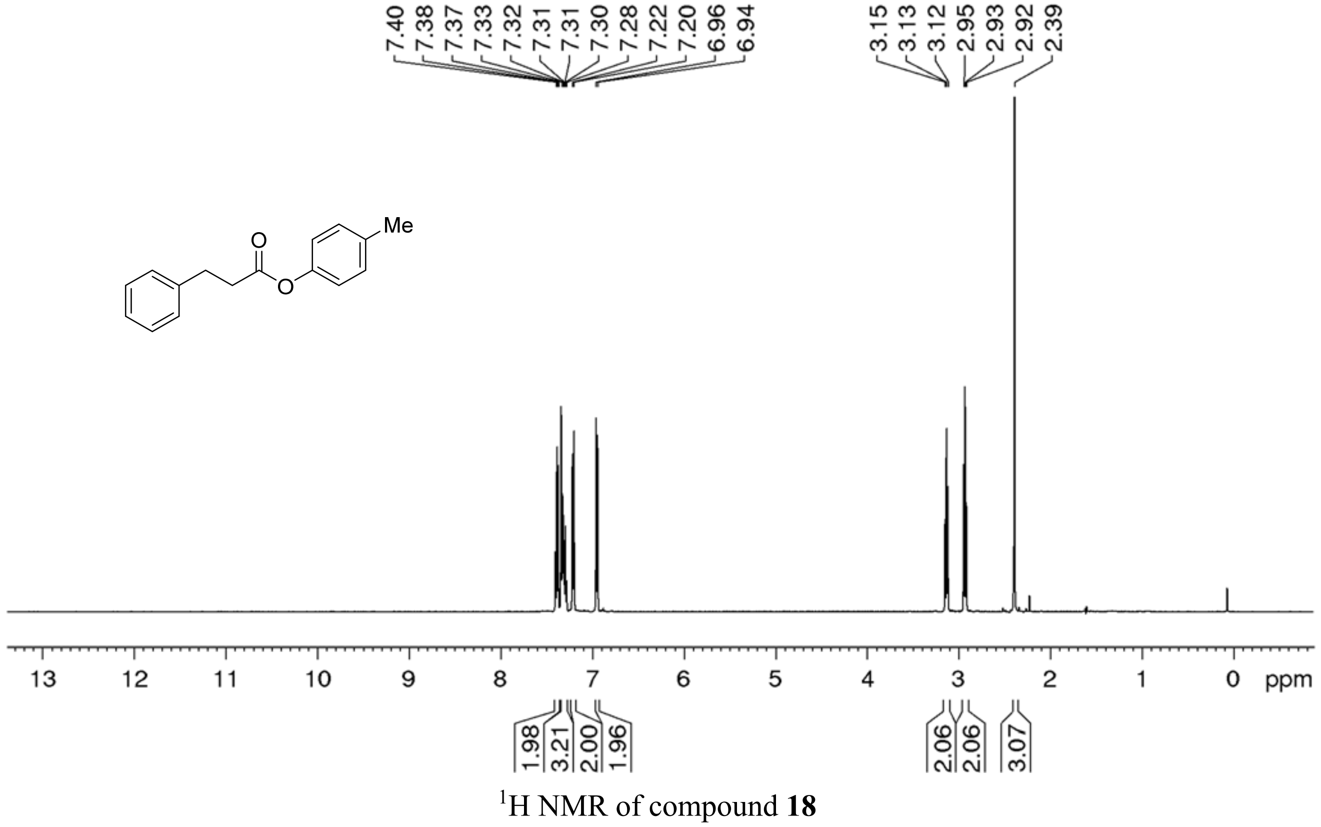

^{1}H NMR of compound **18**

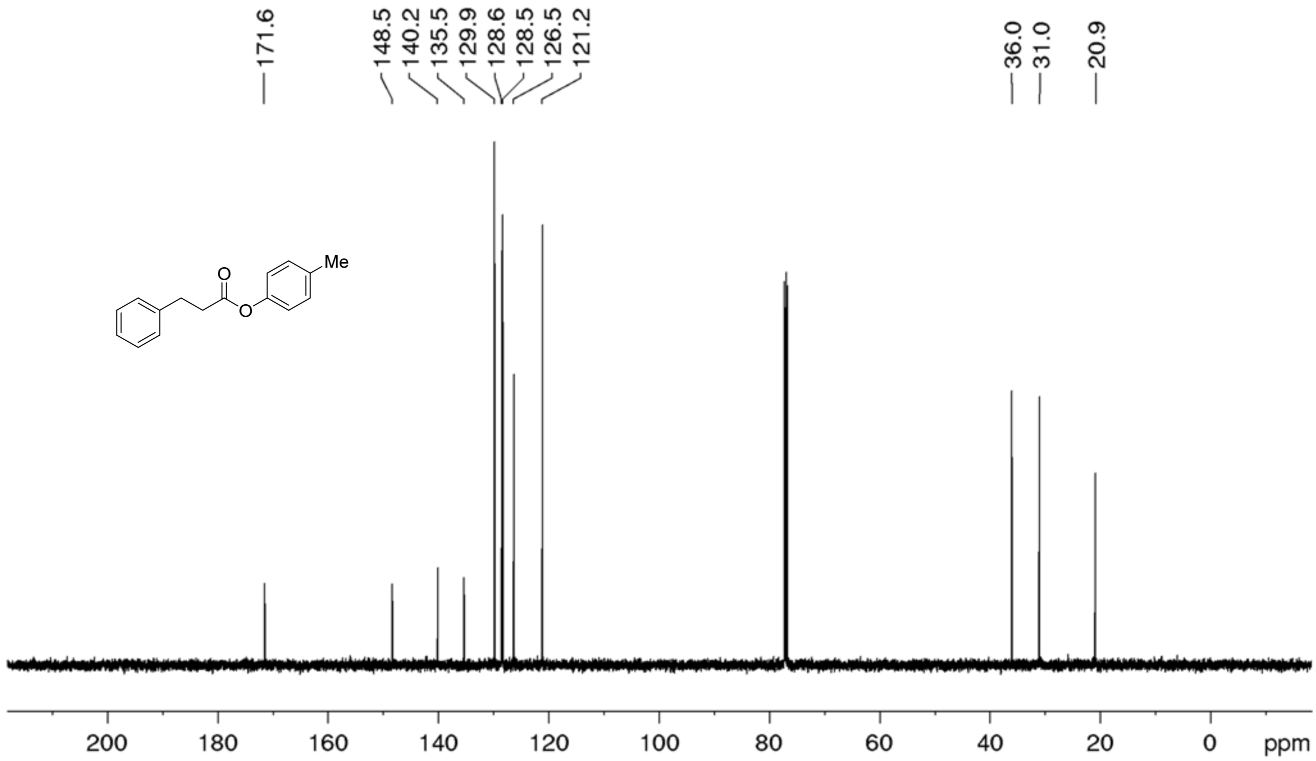

^{13}C NMR of compound **18**

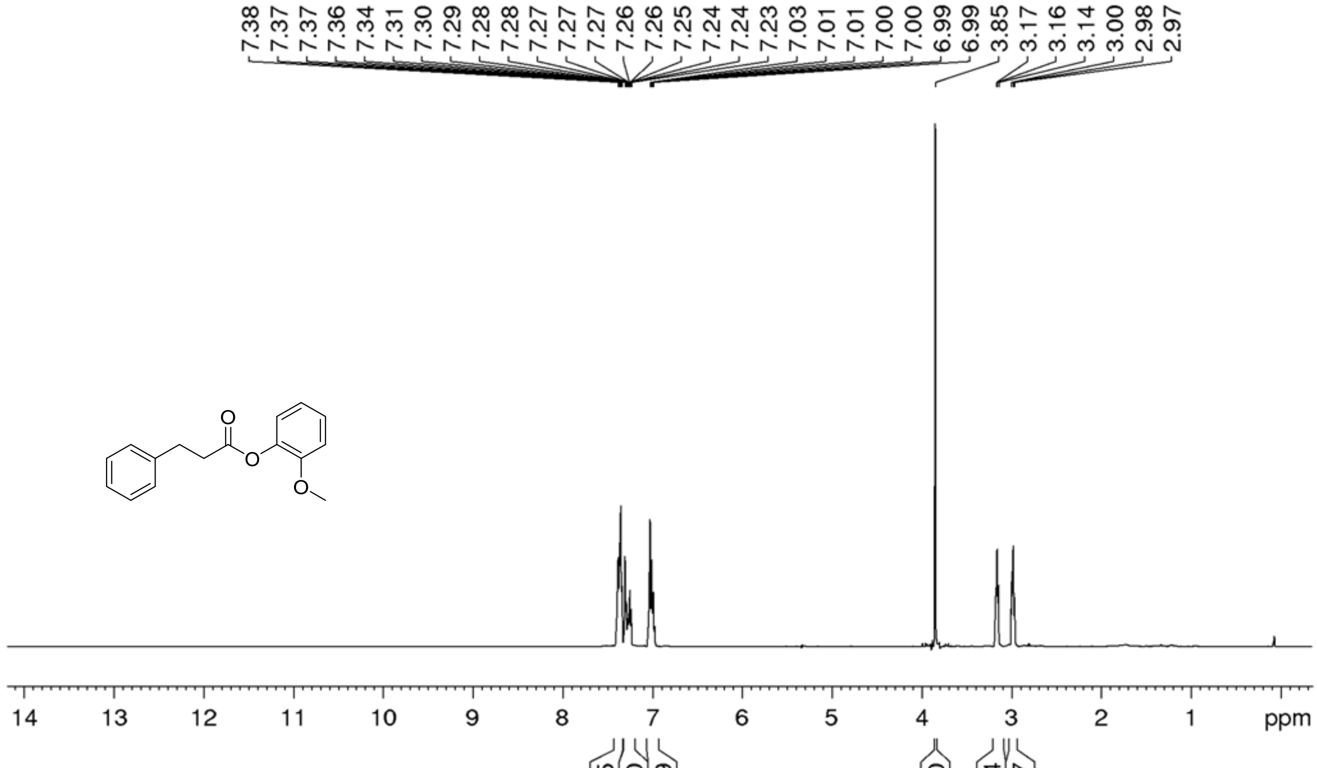

^{1}H NMR of compound **19**

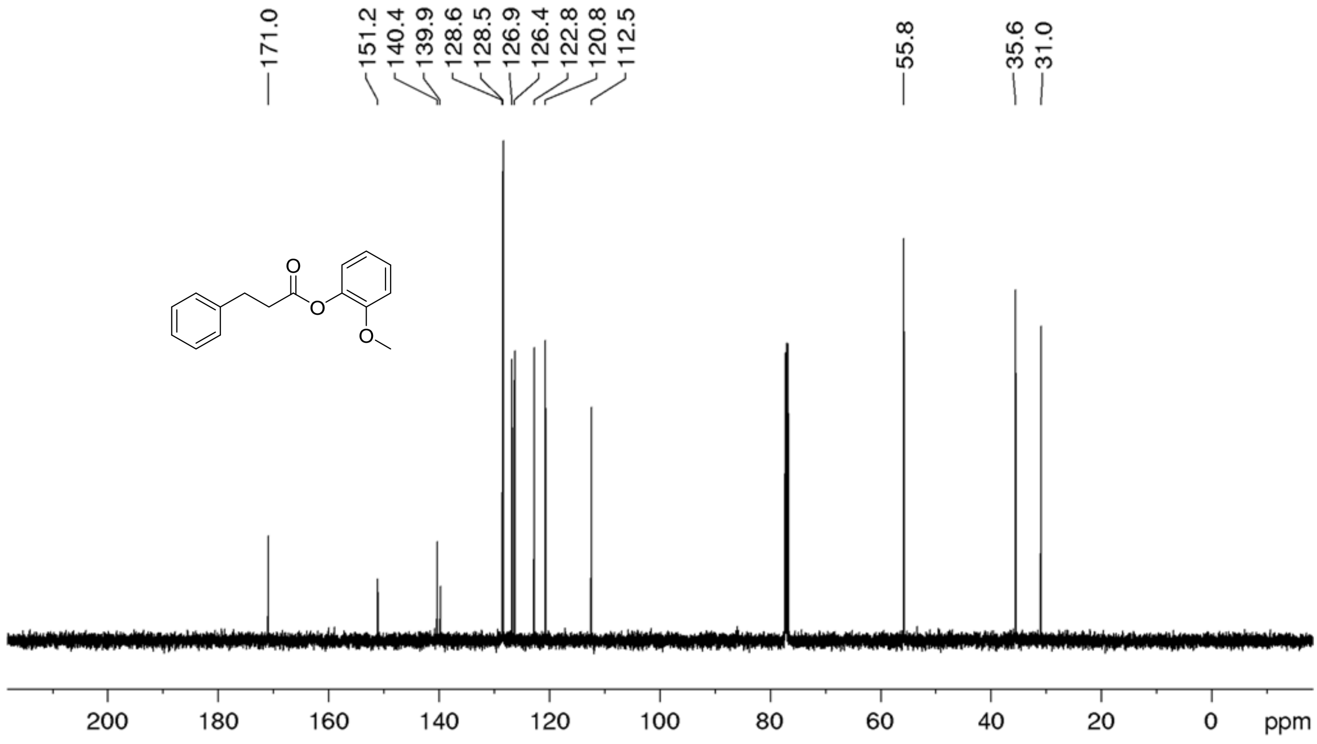

^{13}C NMR of compound **19**

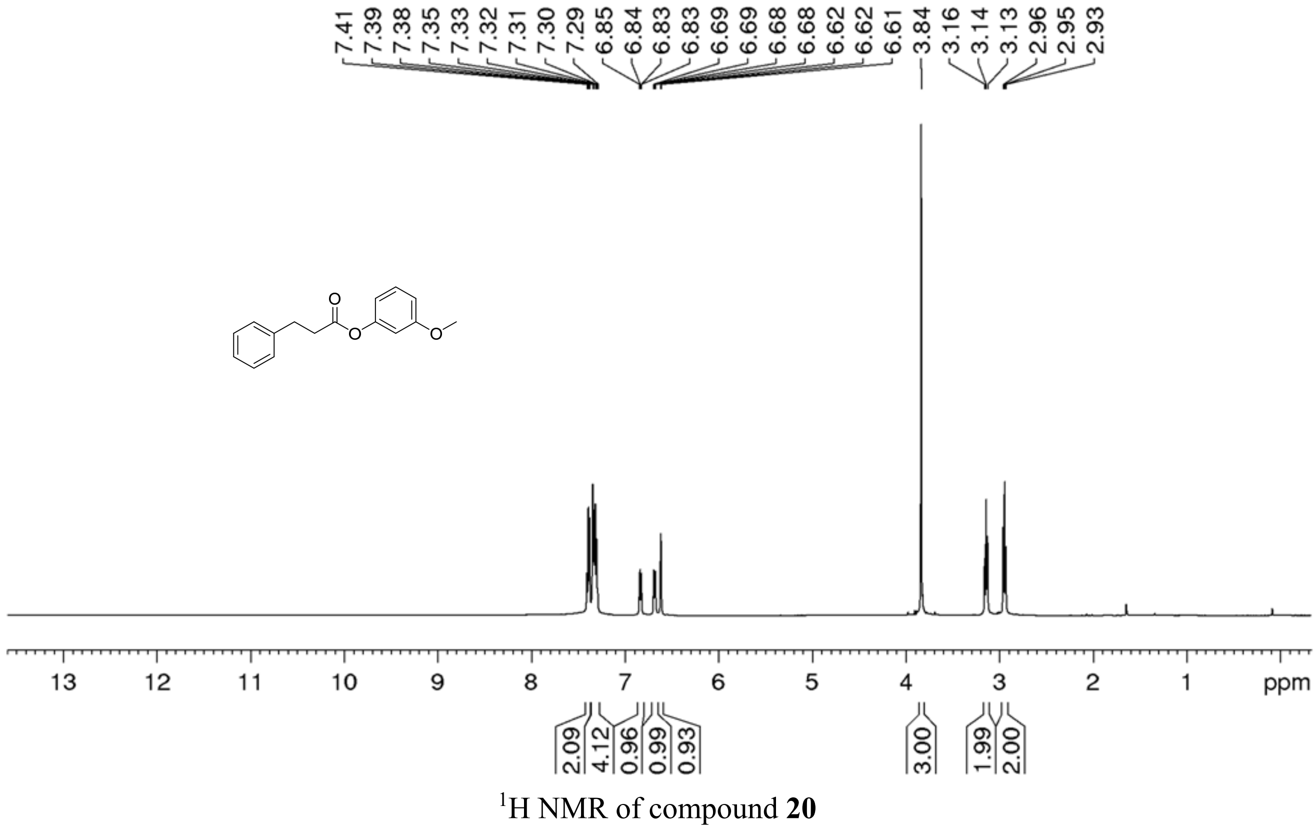

^{1}H NMR of compound **20**

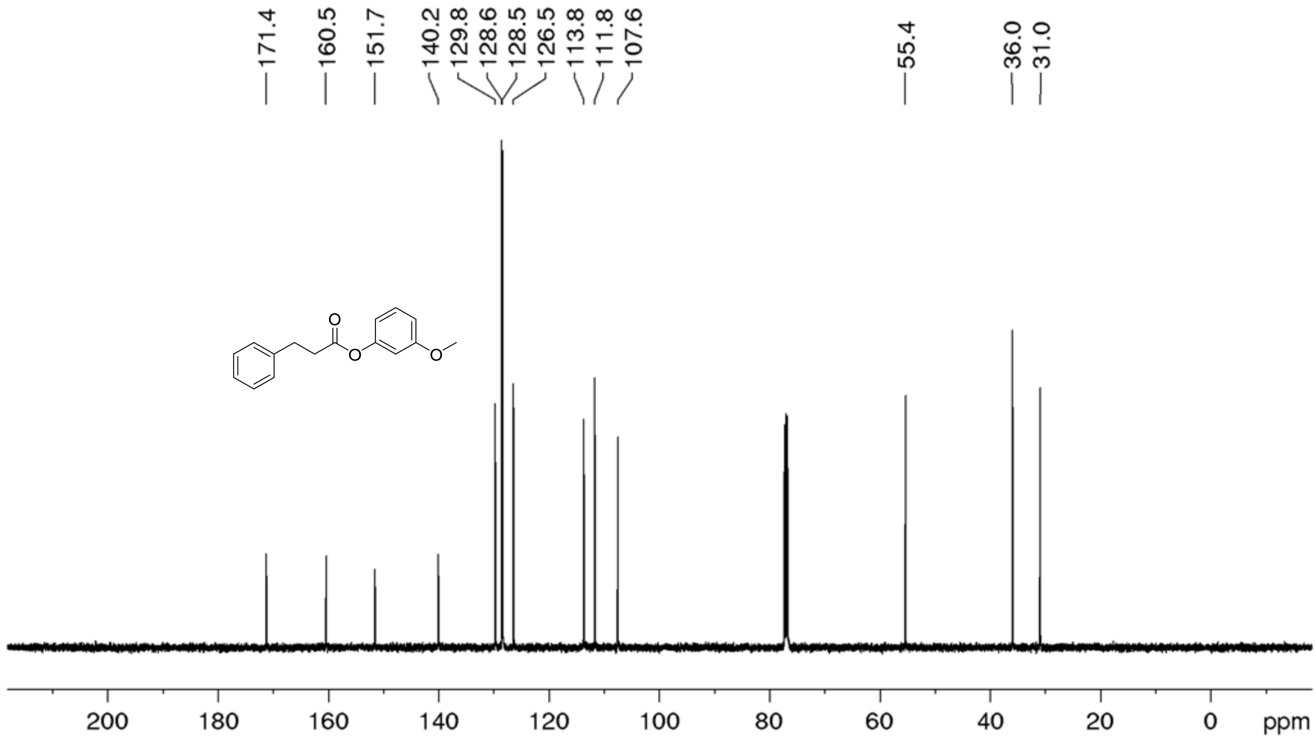

^{13}C NMR of compound **20**

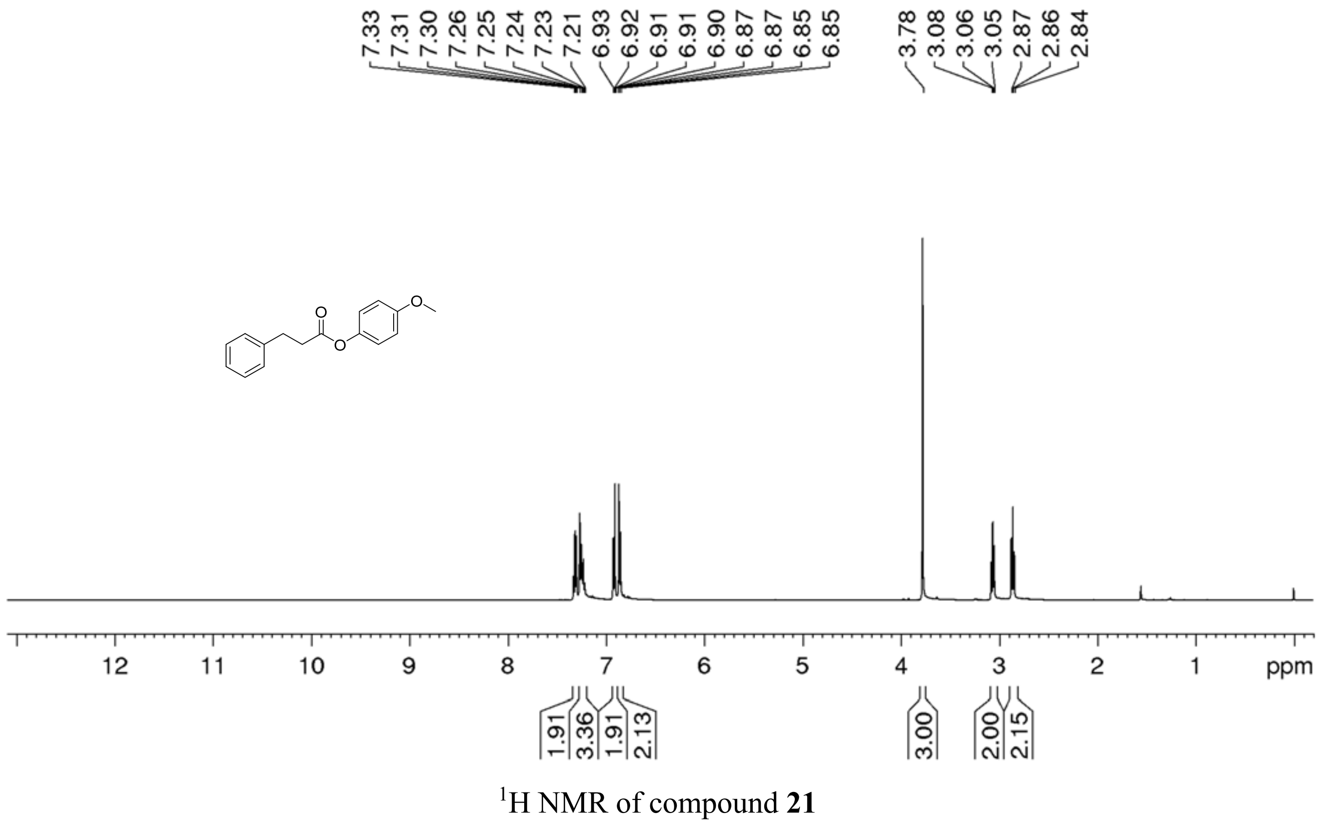

^{1}H NMR of compound **21**

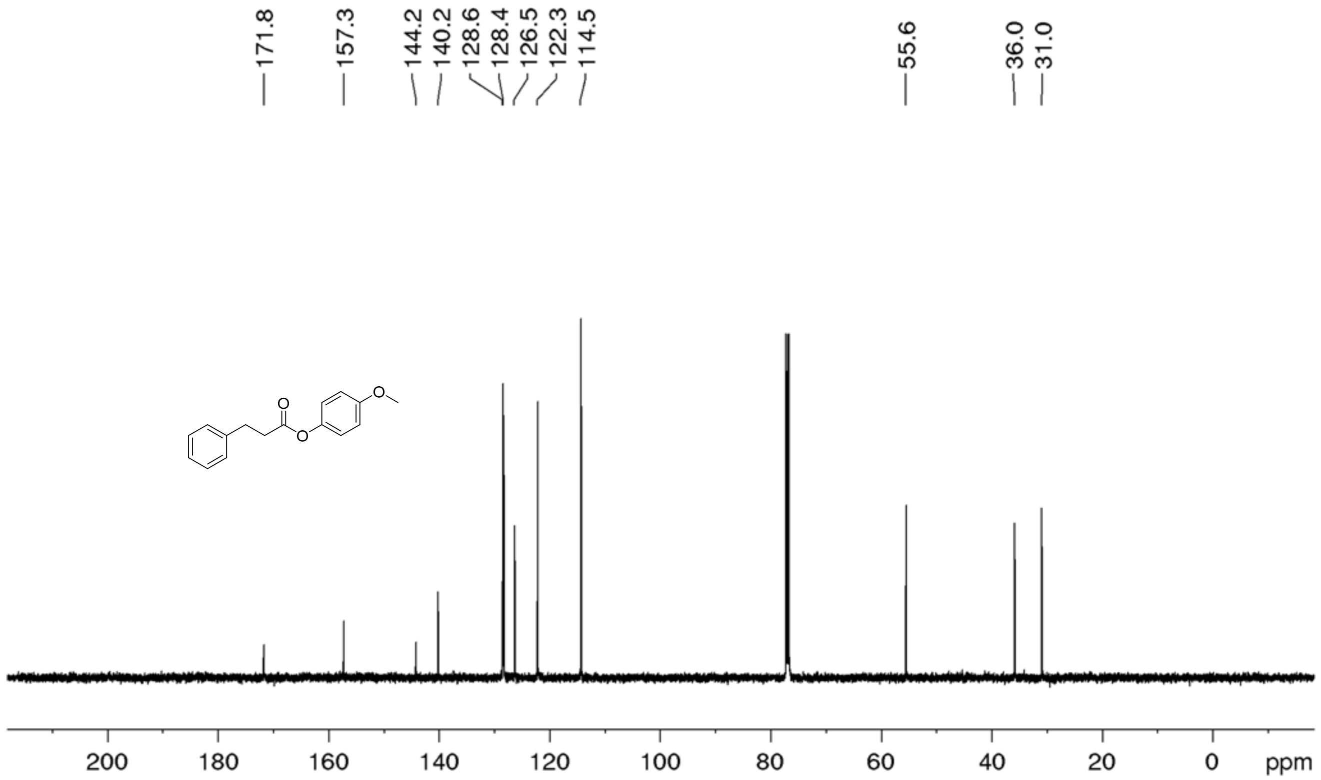

^{13}C NMR of compound **21**

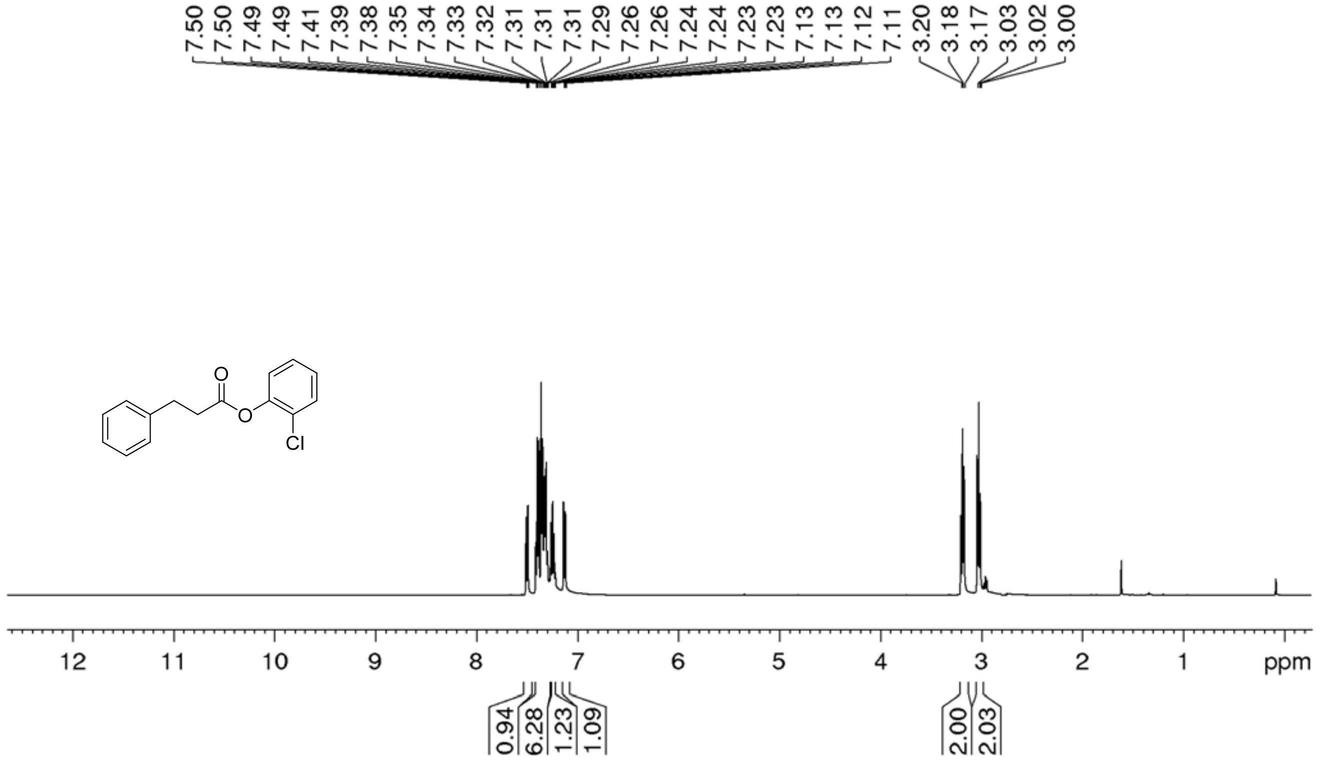

^{1}H NMR of compound **22**

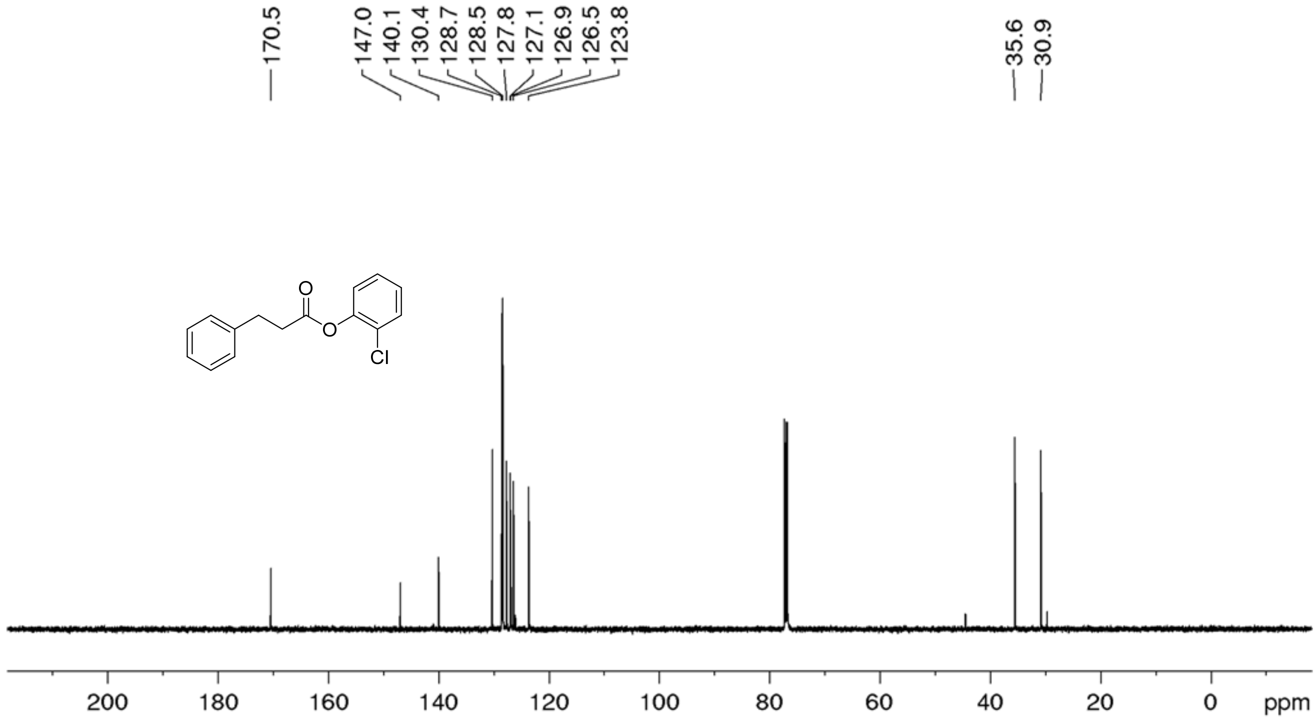

^{13}C NMR of compound **22**

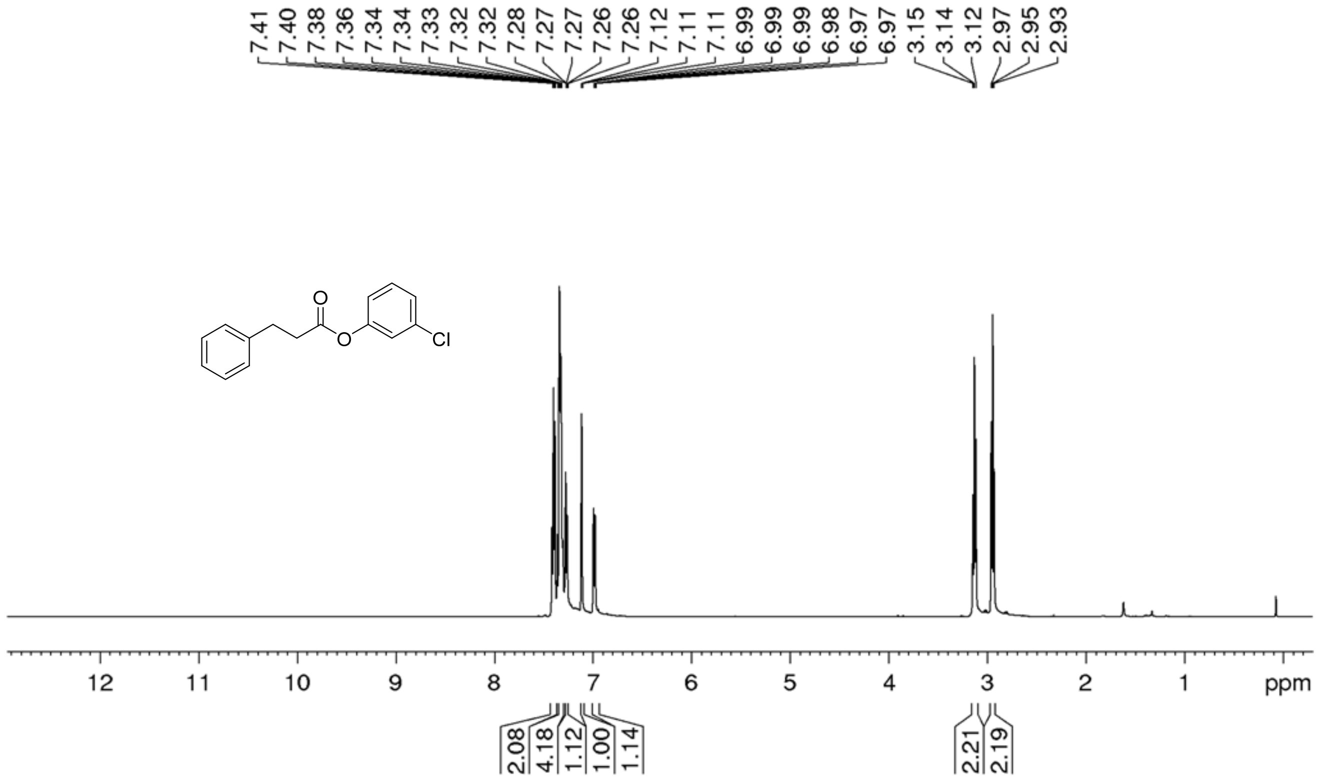

^{1}H NMR of compound **23**

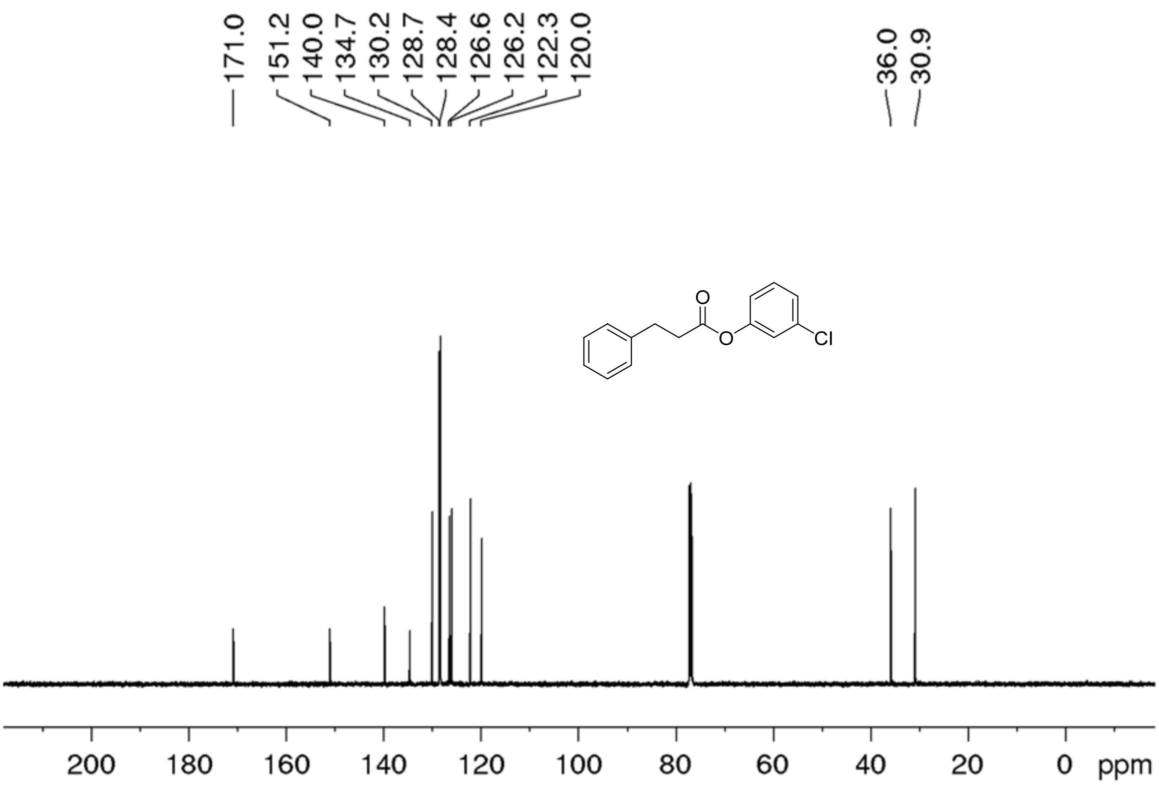

^{13}C NMR of compound **23**

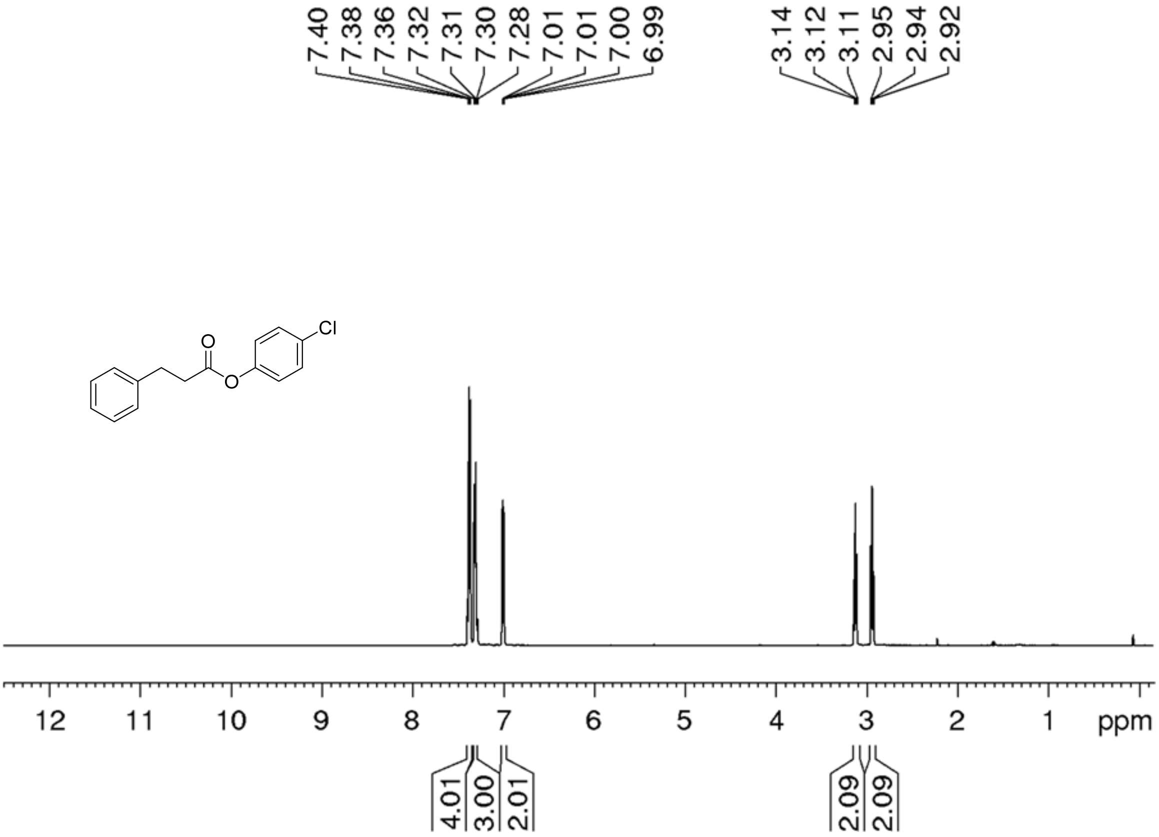

^{1}H NMR of compound **24**

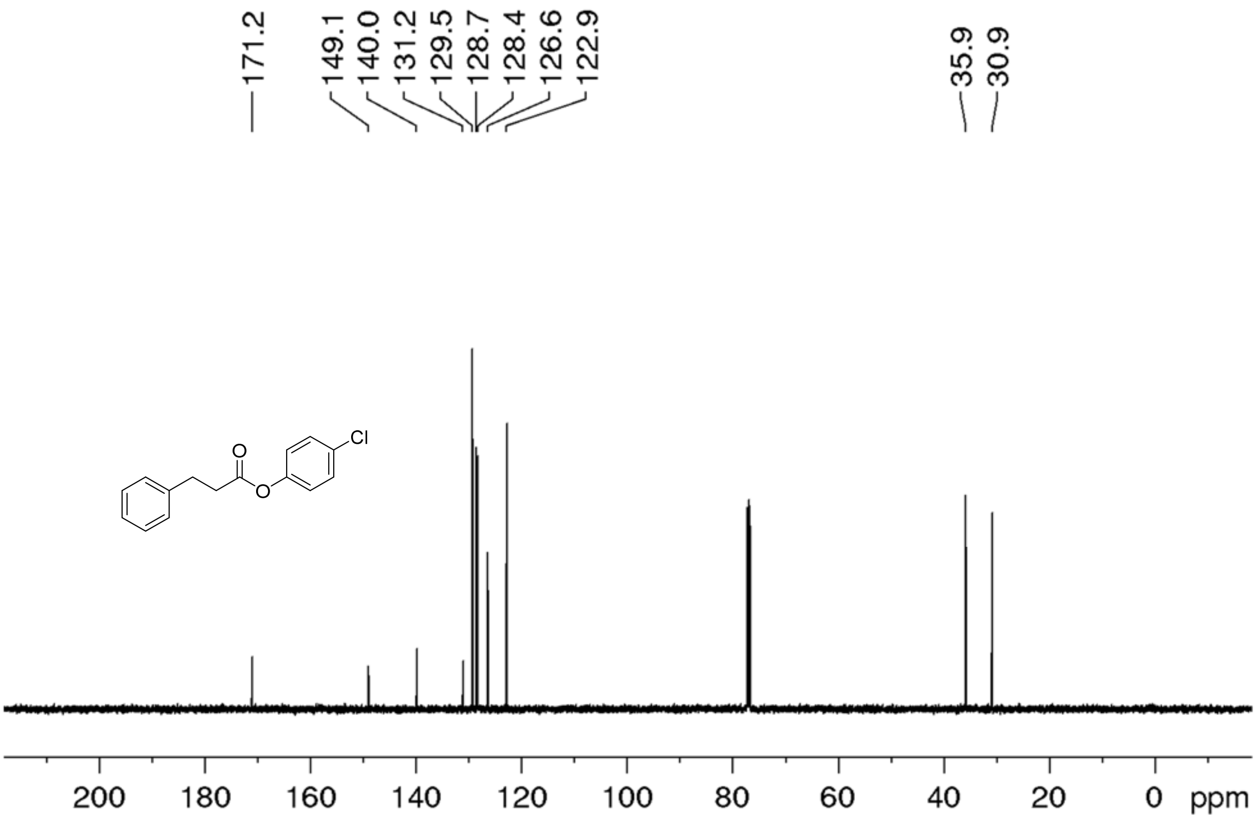

^{13}C NMR of compound **24**

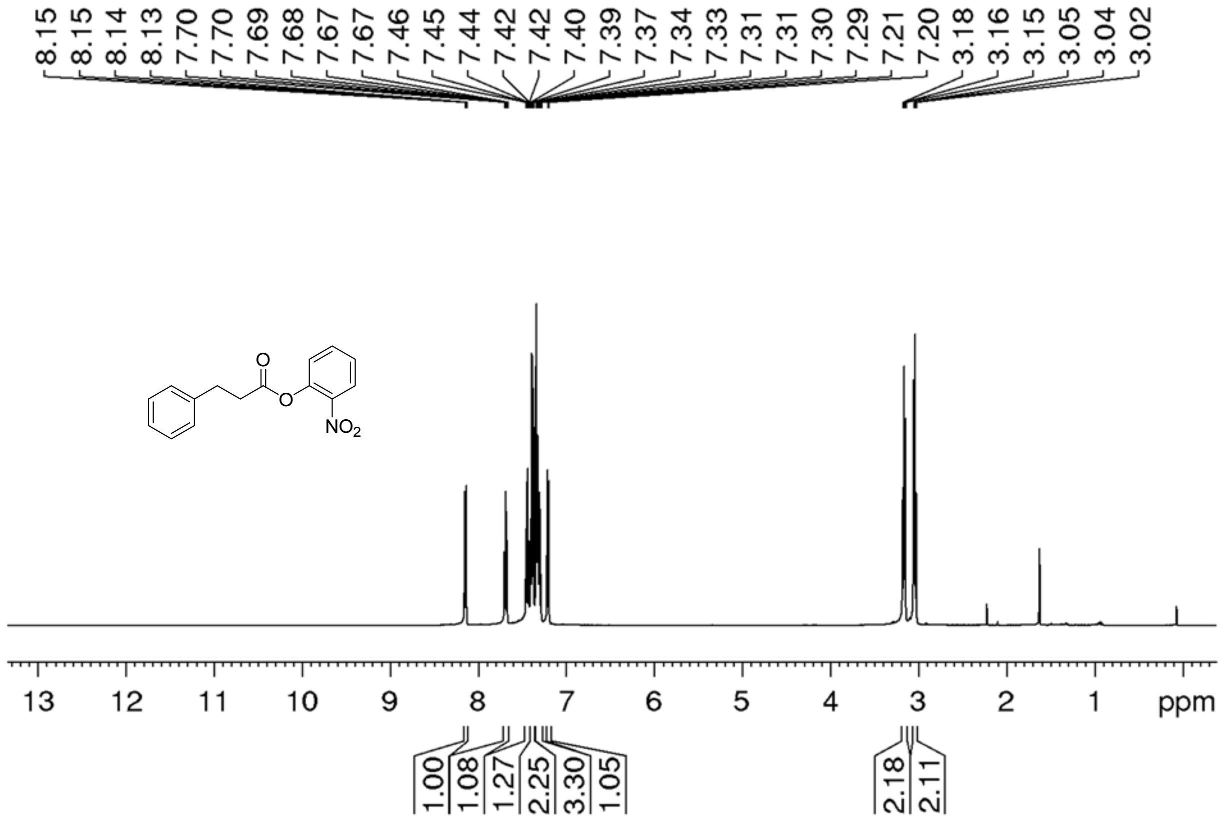

^{1}H NMR of compound **25**

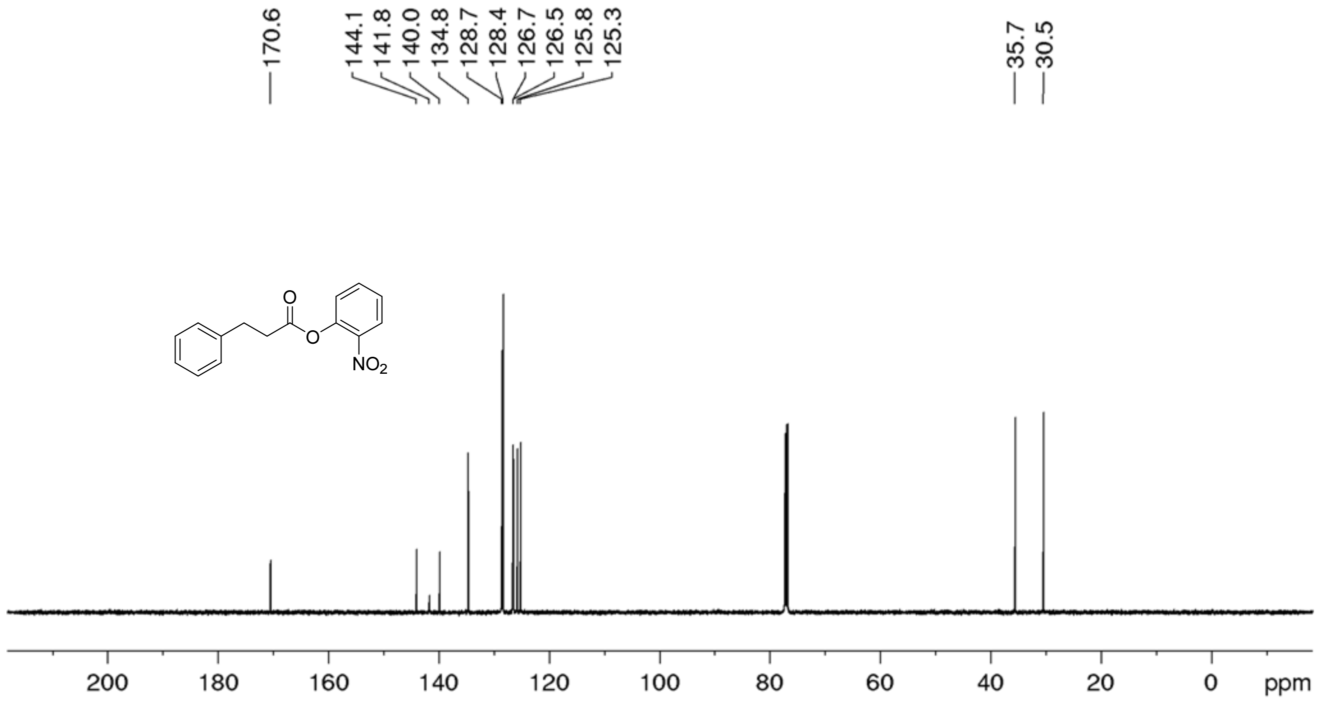

^{13}C NMR of compound **25**

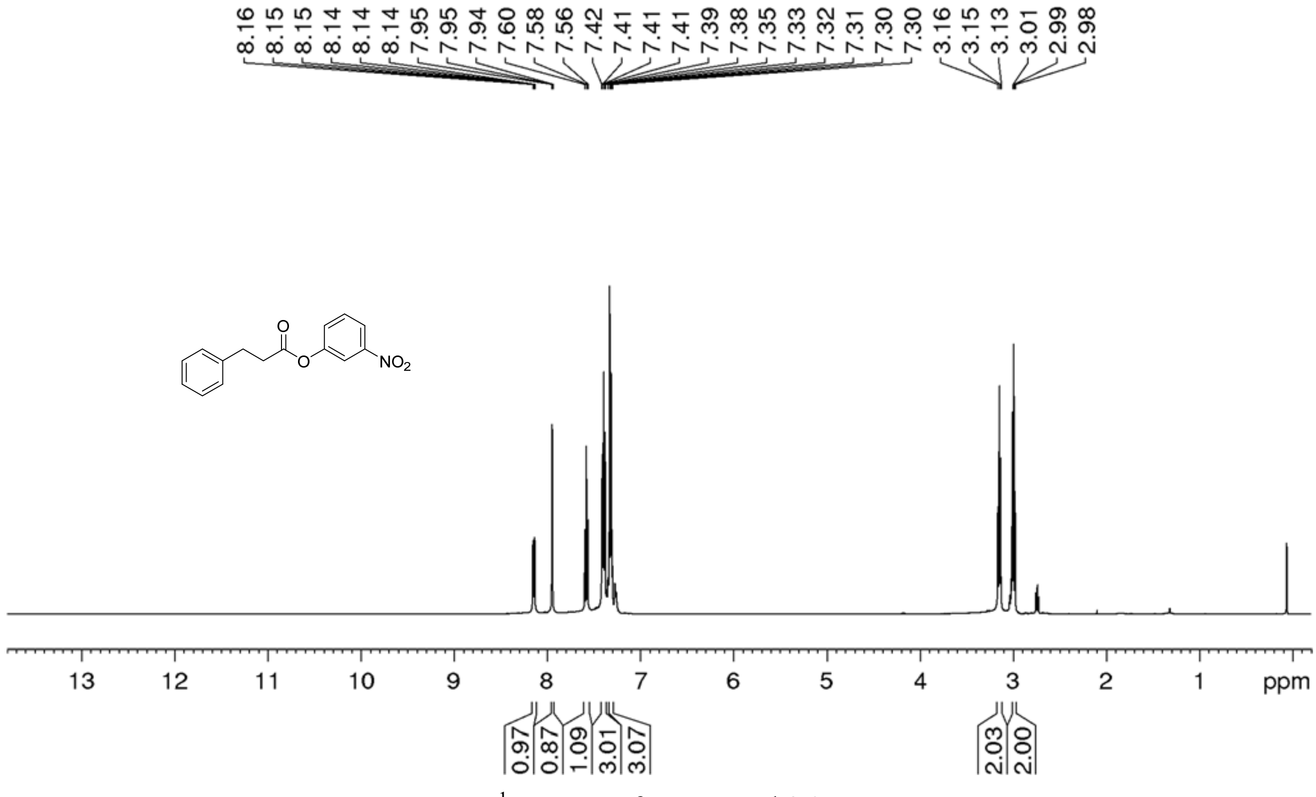

^{1}H NMR of compound **26**

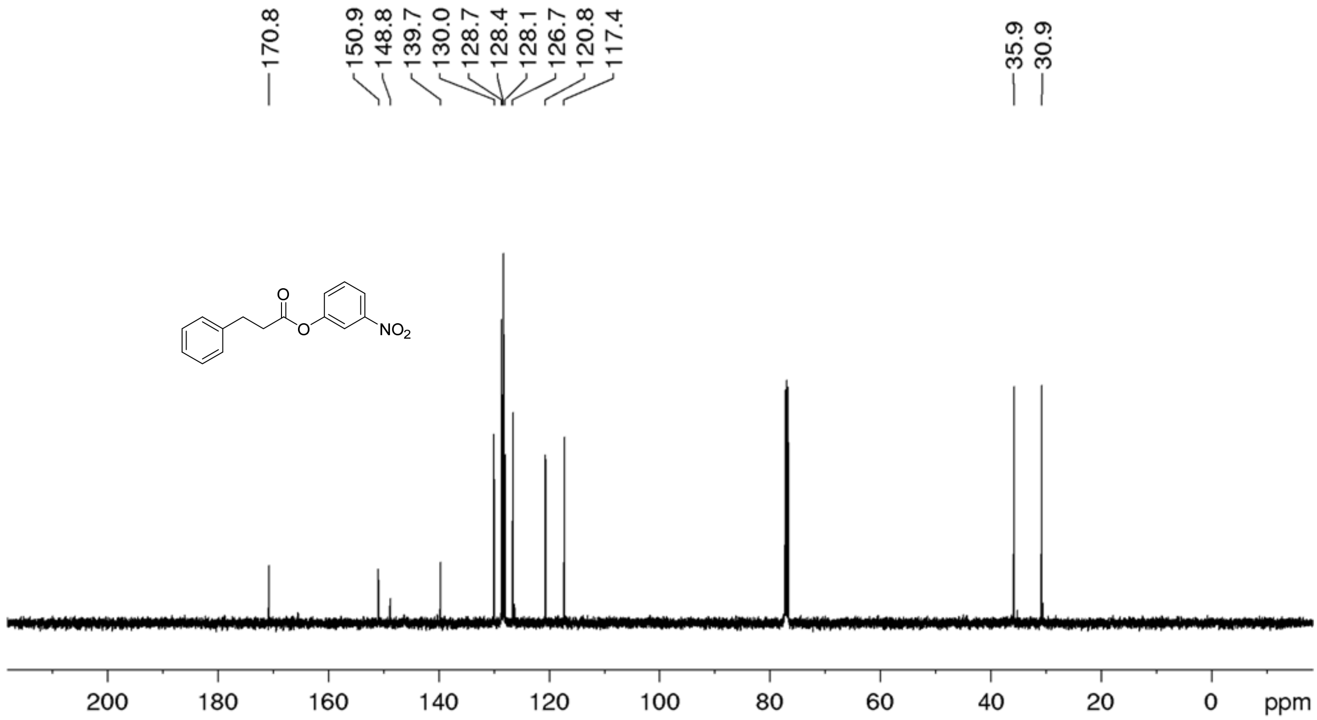

^{13}C NMR of compound **26**

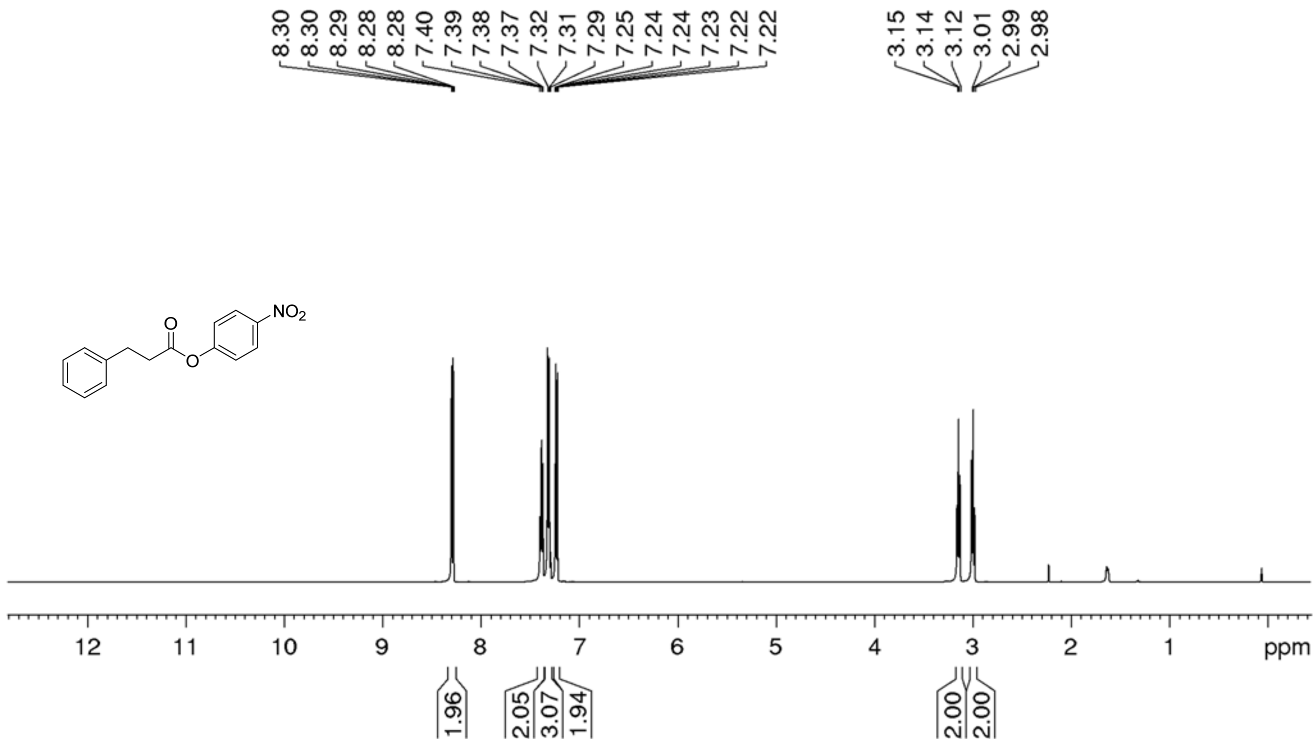

^{1}H NMR of compound **27**

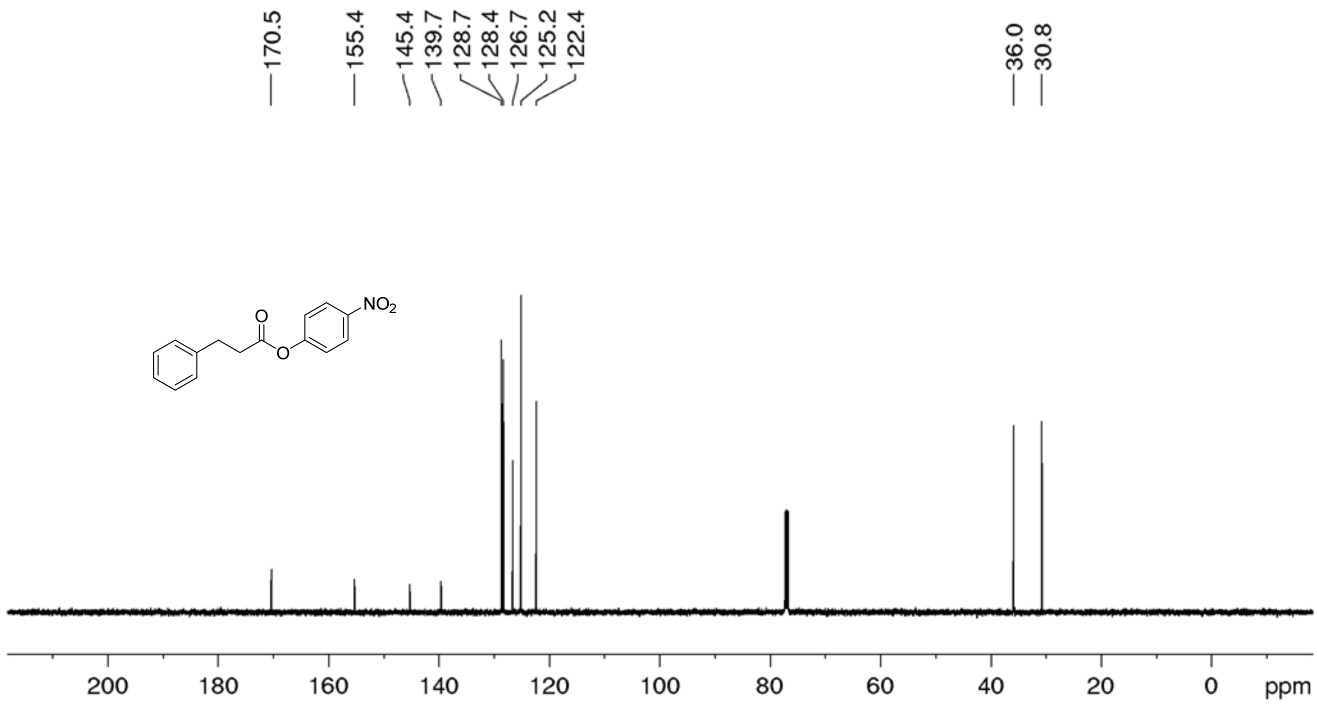

^{13}C NMR of compound **27**

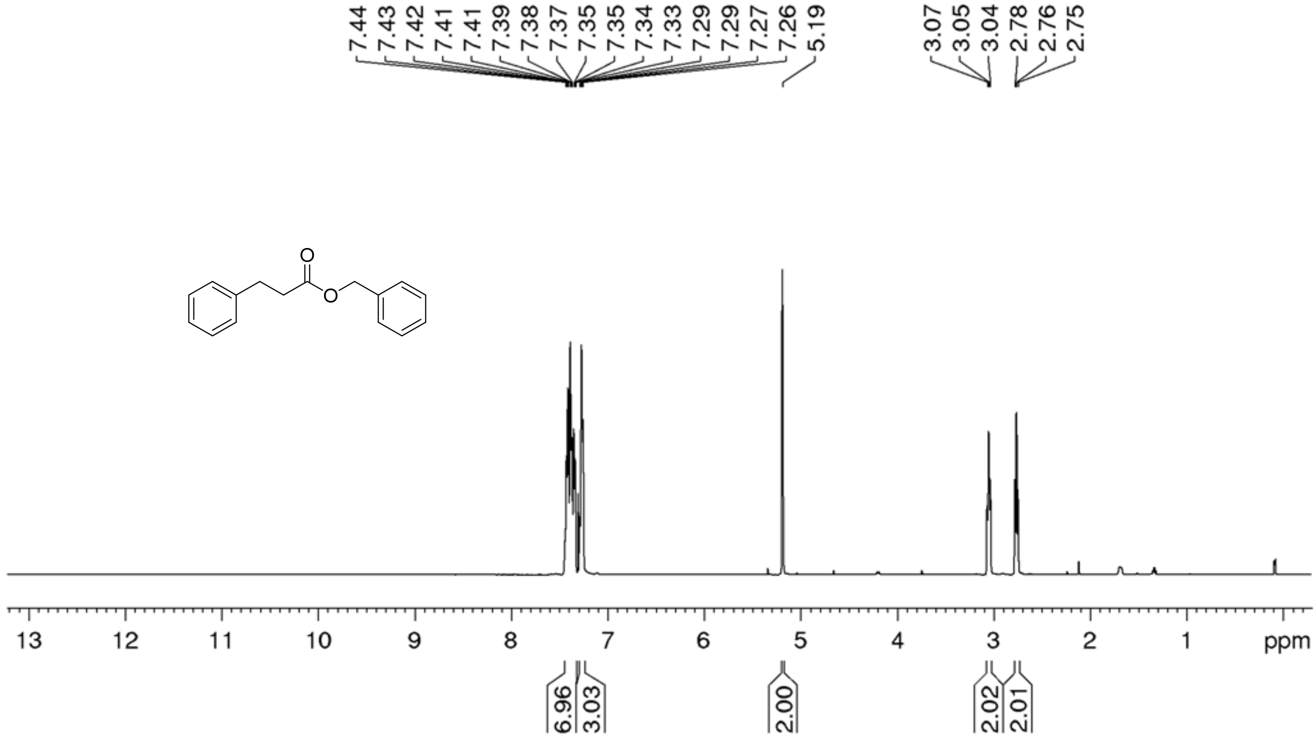

^{1}H NMR of compound **28**

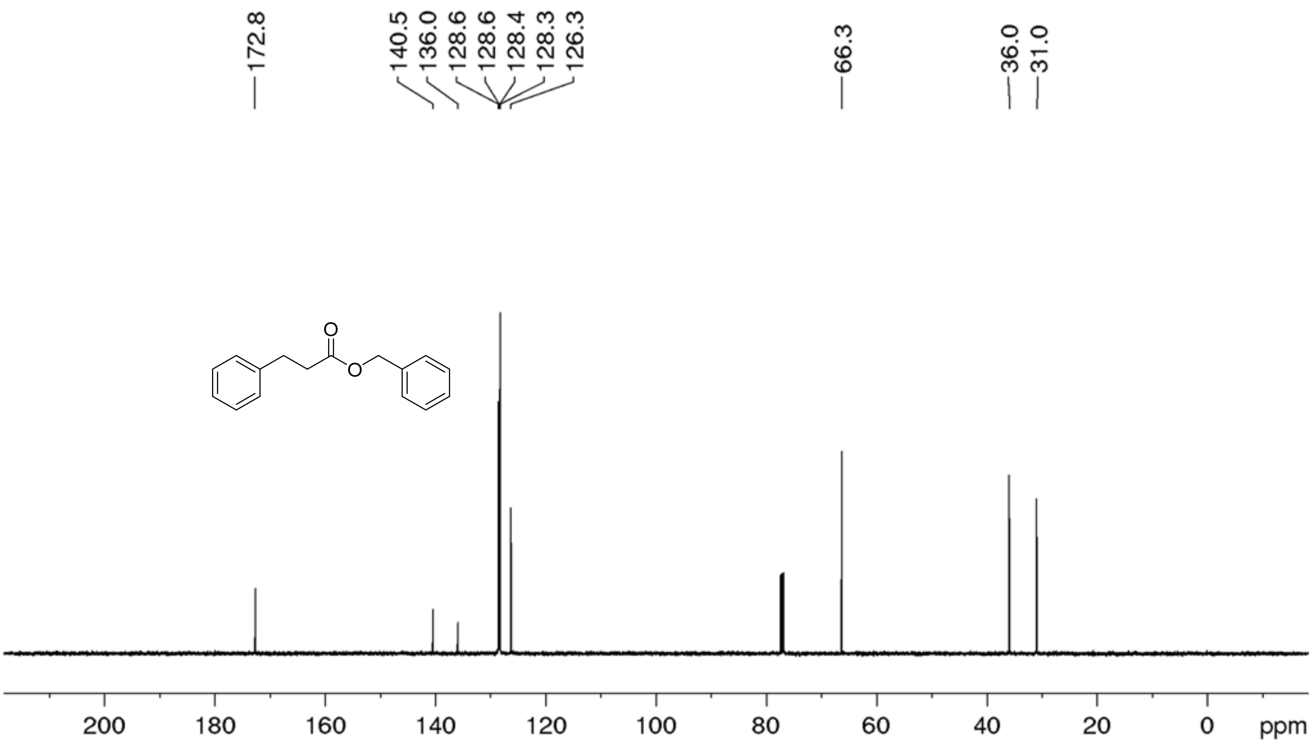

^{13}C NMR of compound **28**

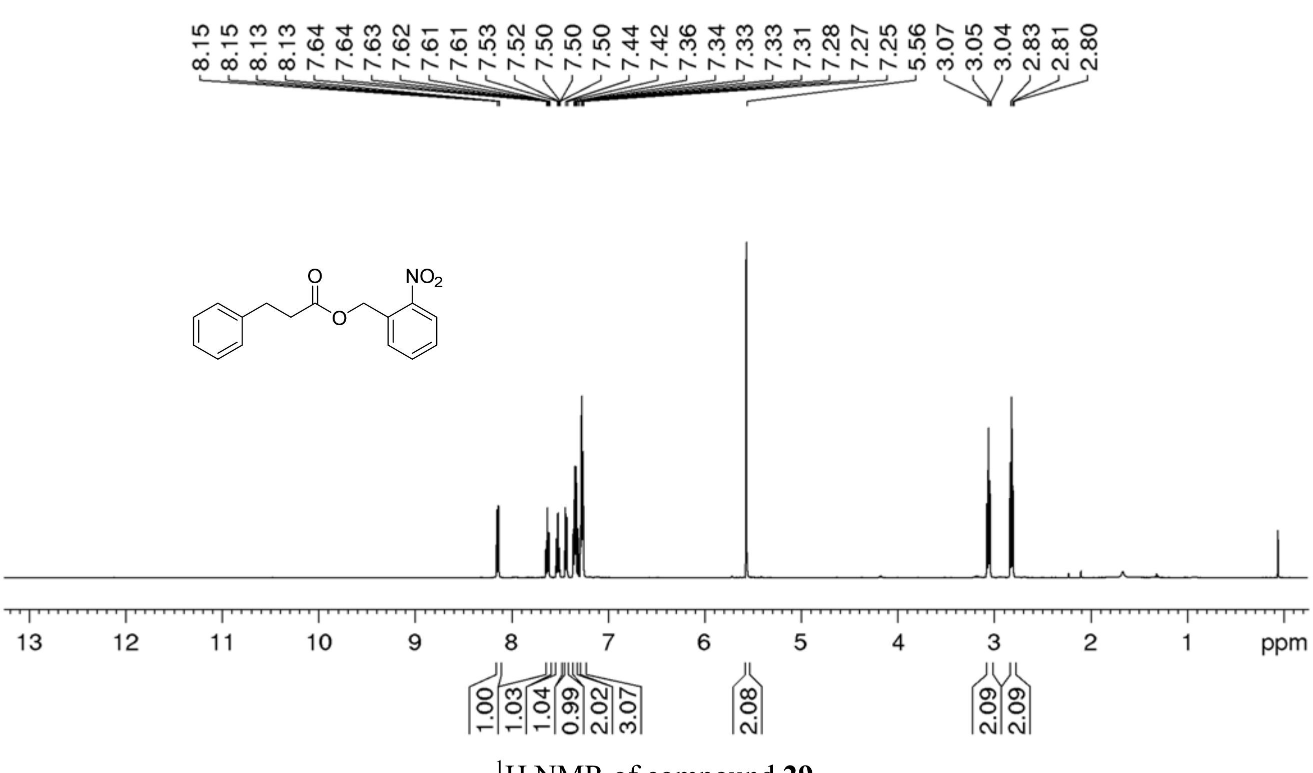

^{1}H NMR of compound **29**

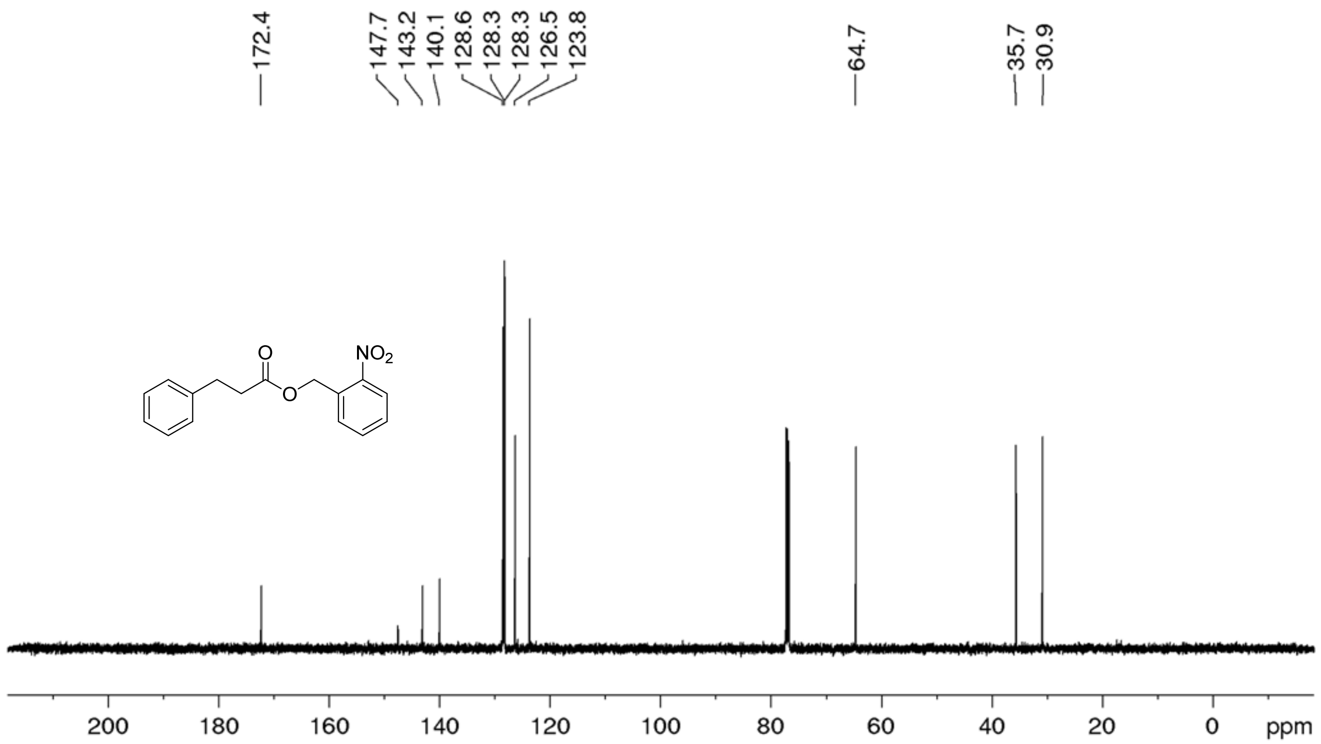

^{13}C NMR of compound **29**

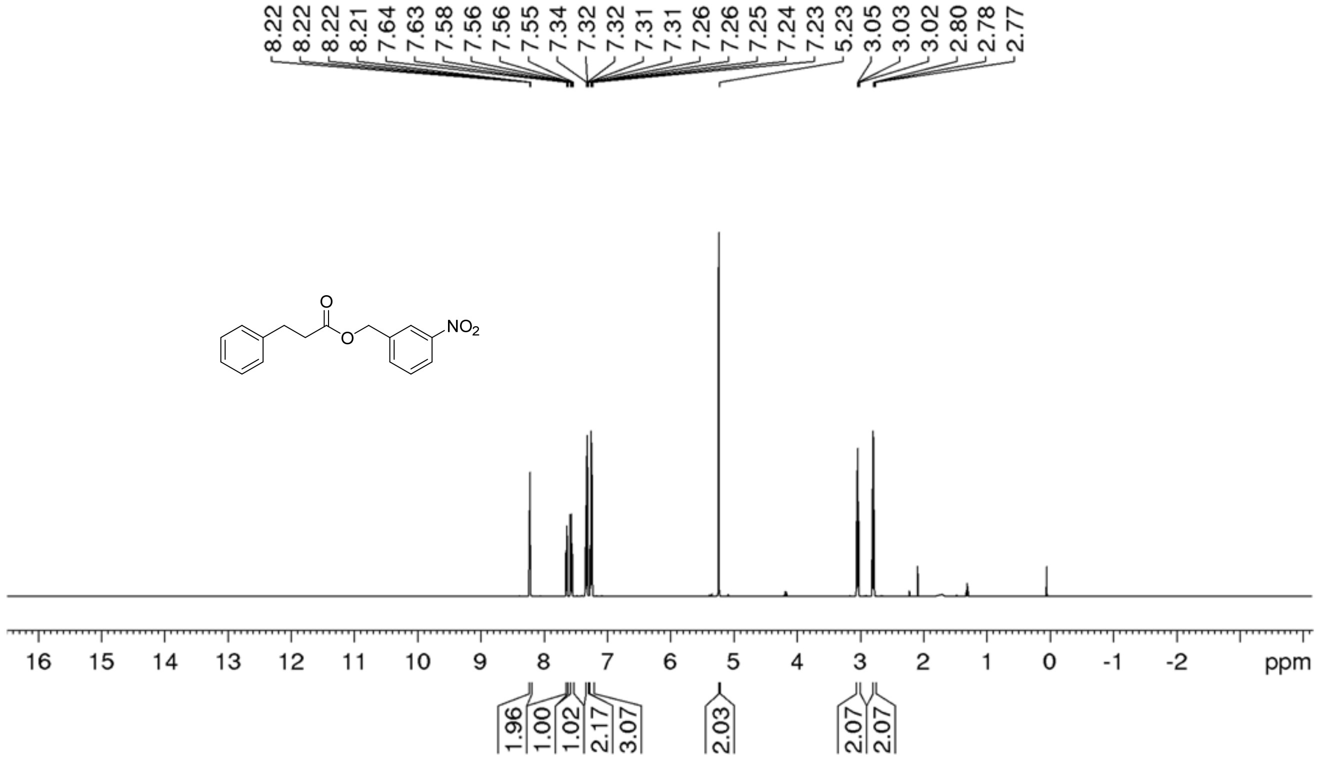

^{1}H NMR of compound **30**

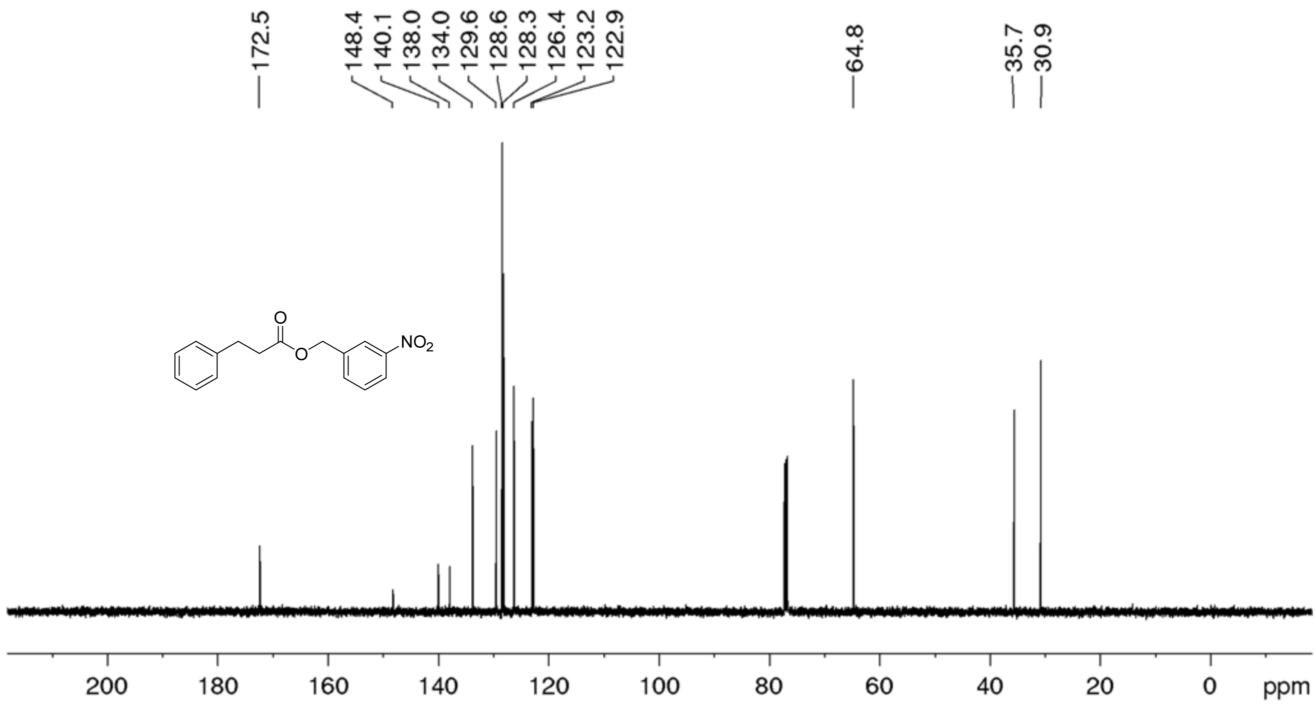

^{13}C NMR of compound **30**

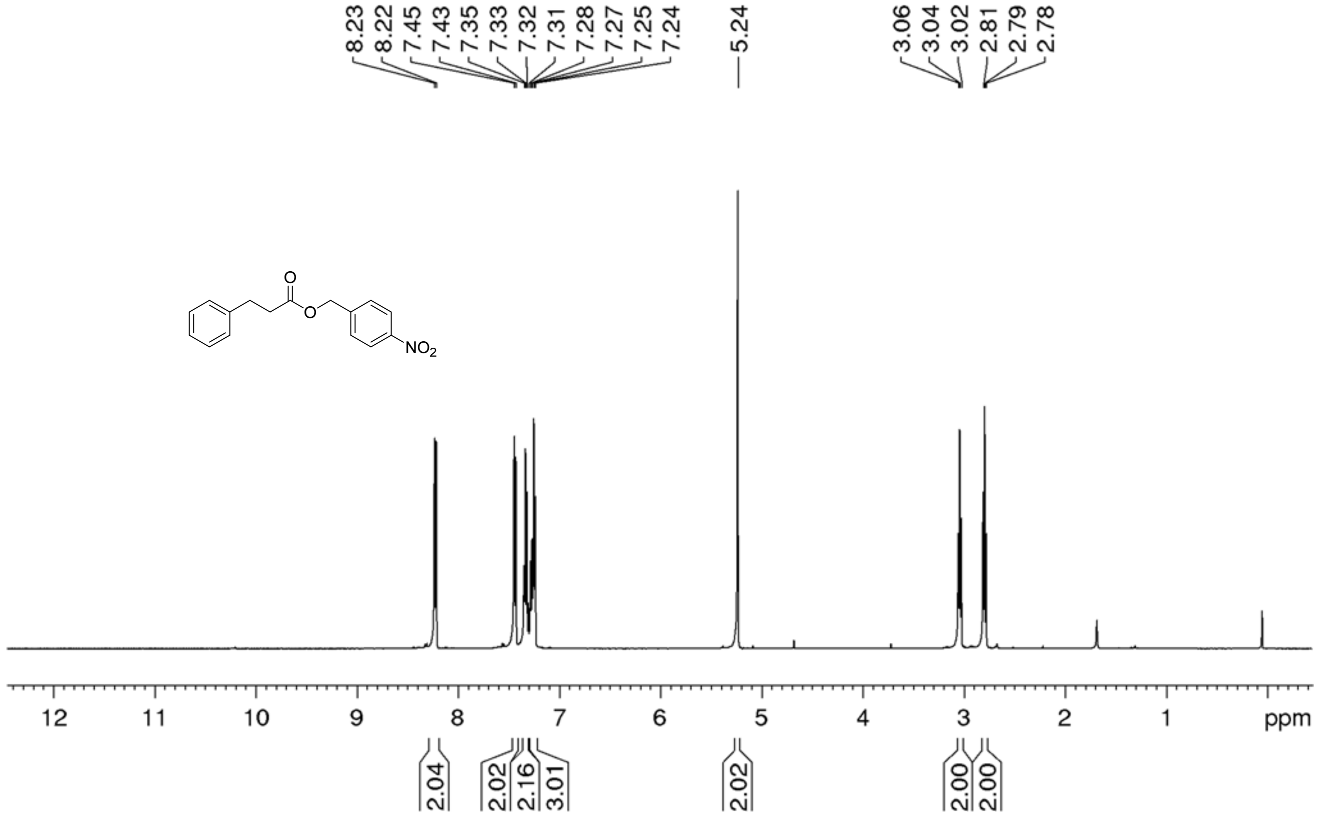

^{1}H NMR of compound **31**

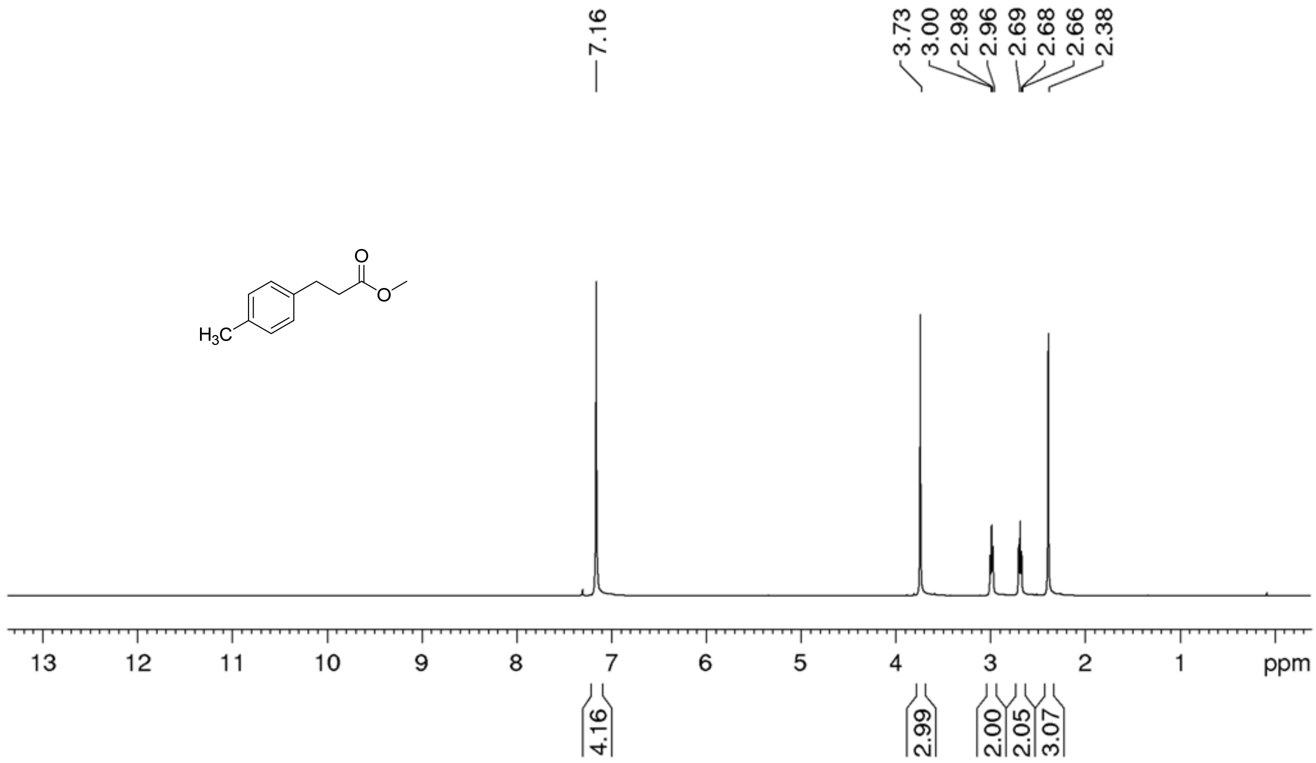

^{1}H NMR of compound **32**

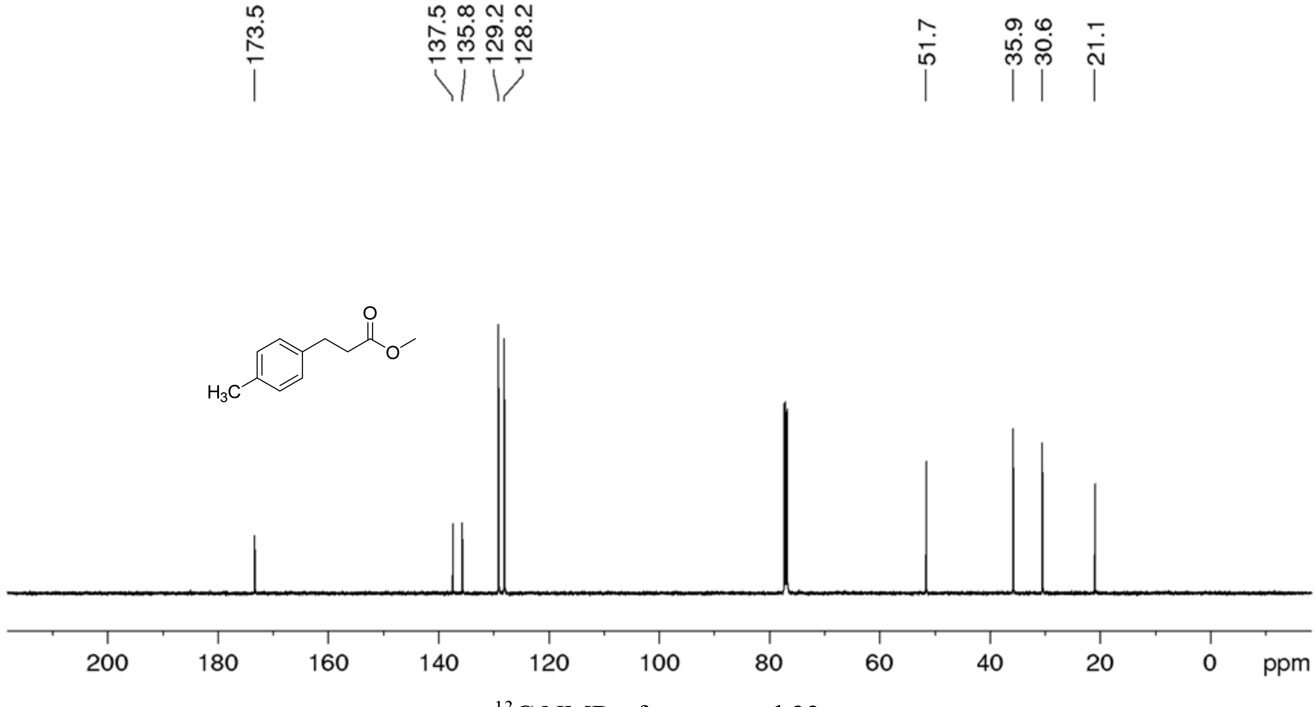

^{13}C NMR of compound **32**

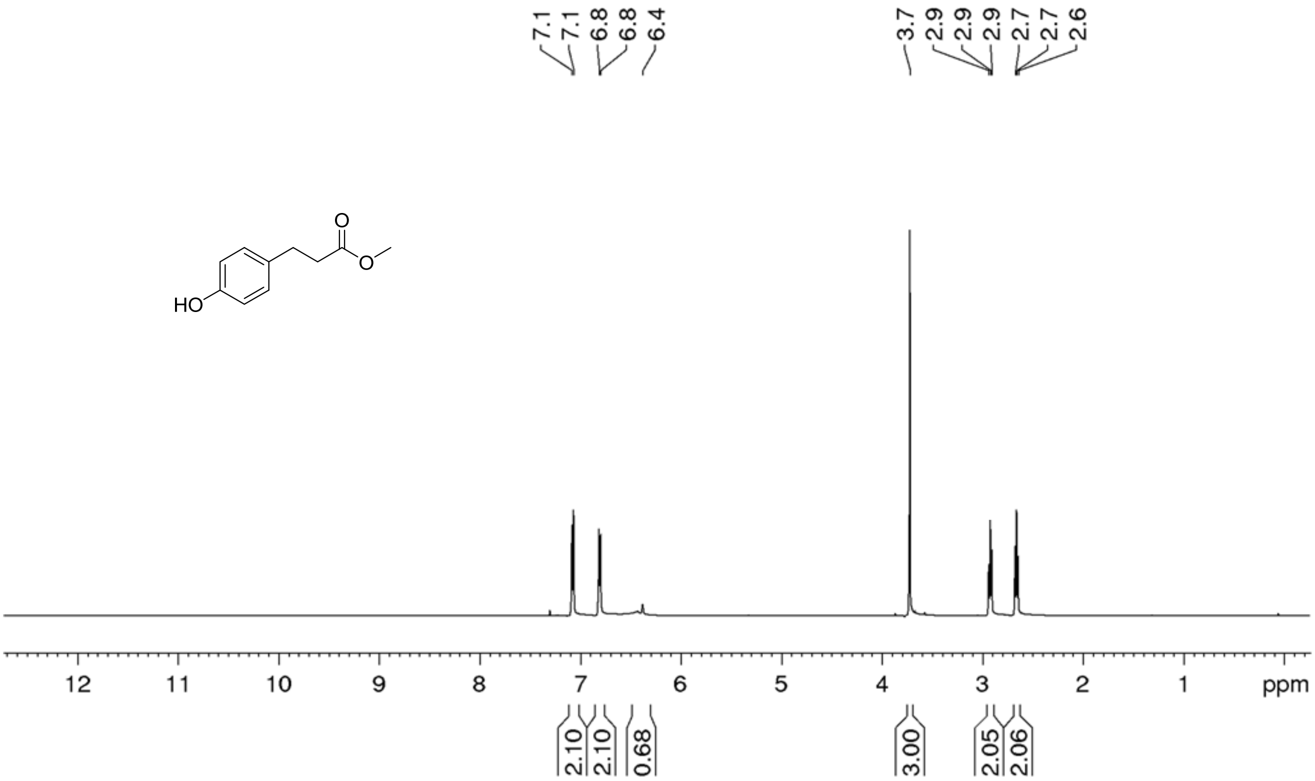

^{1}H NMR of compound **33**

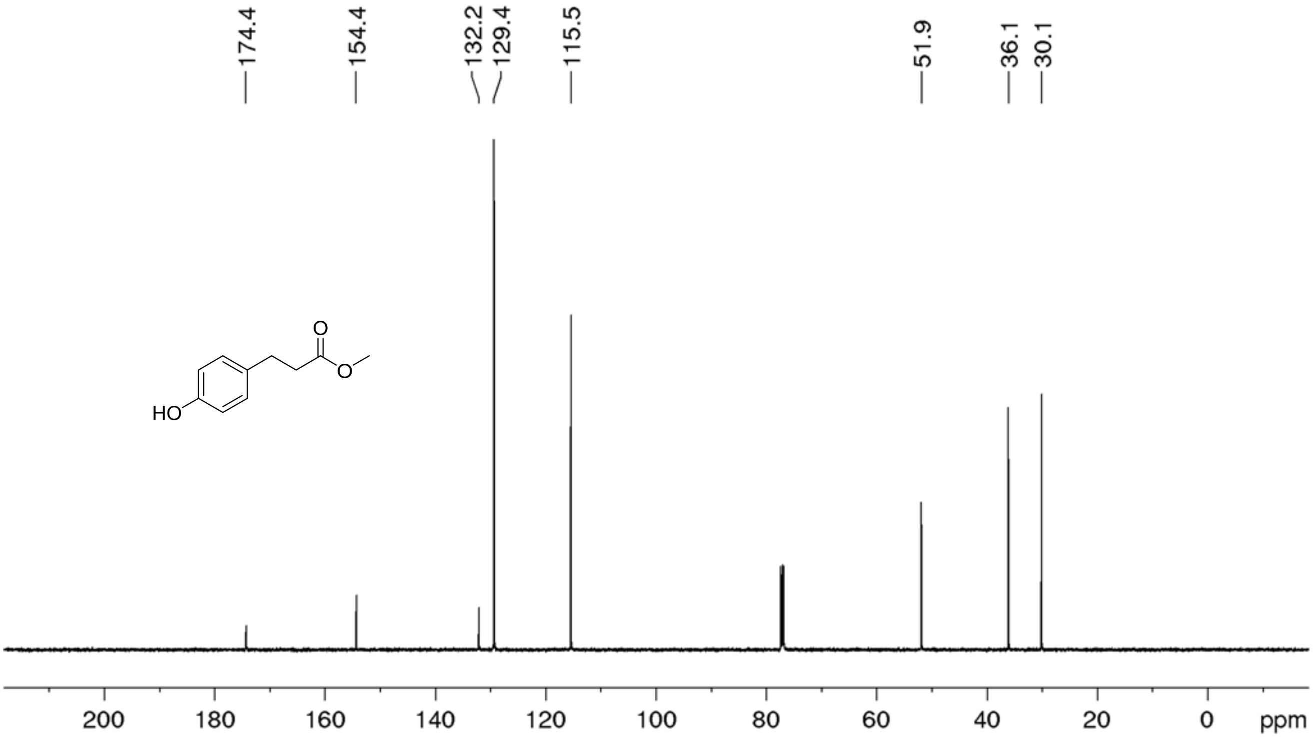

^{13}C NMR of compound **33**

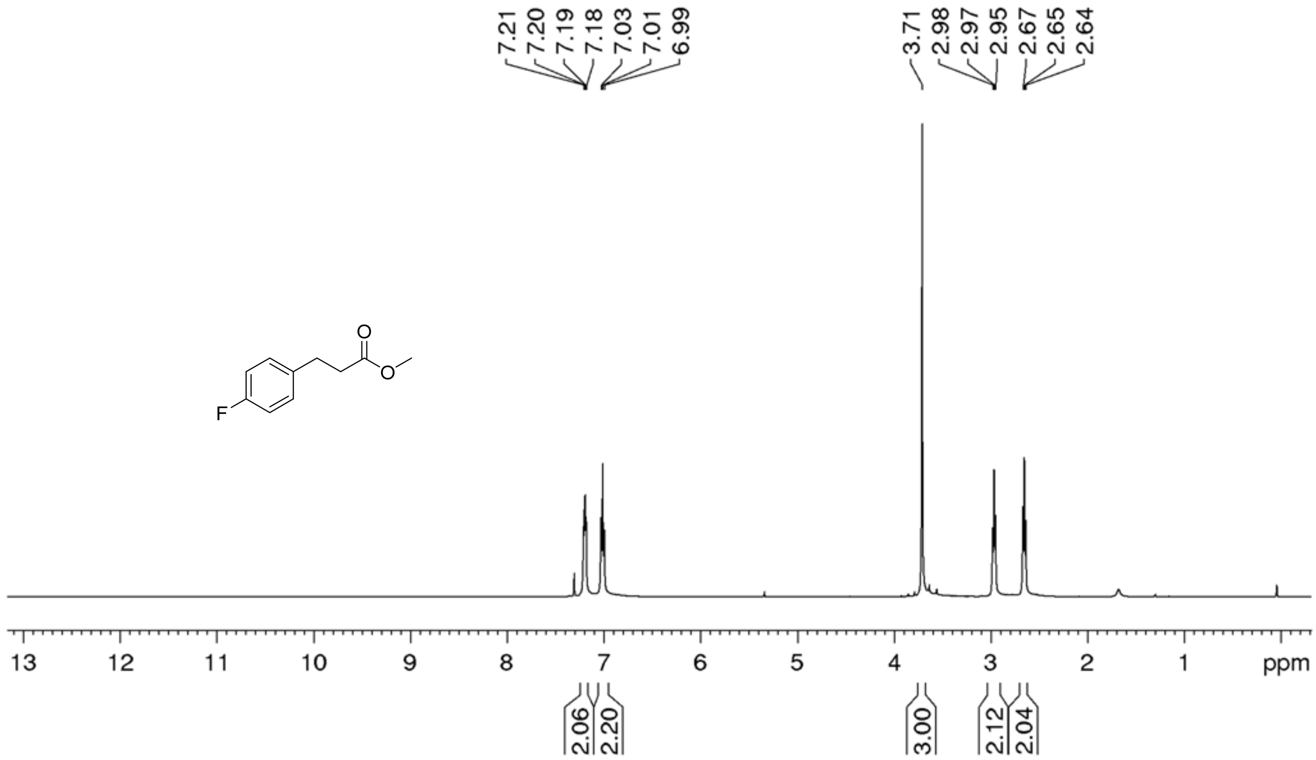

^{1}H NMR of compound **34**

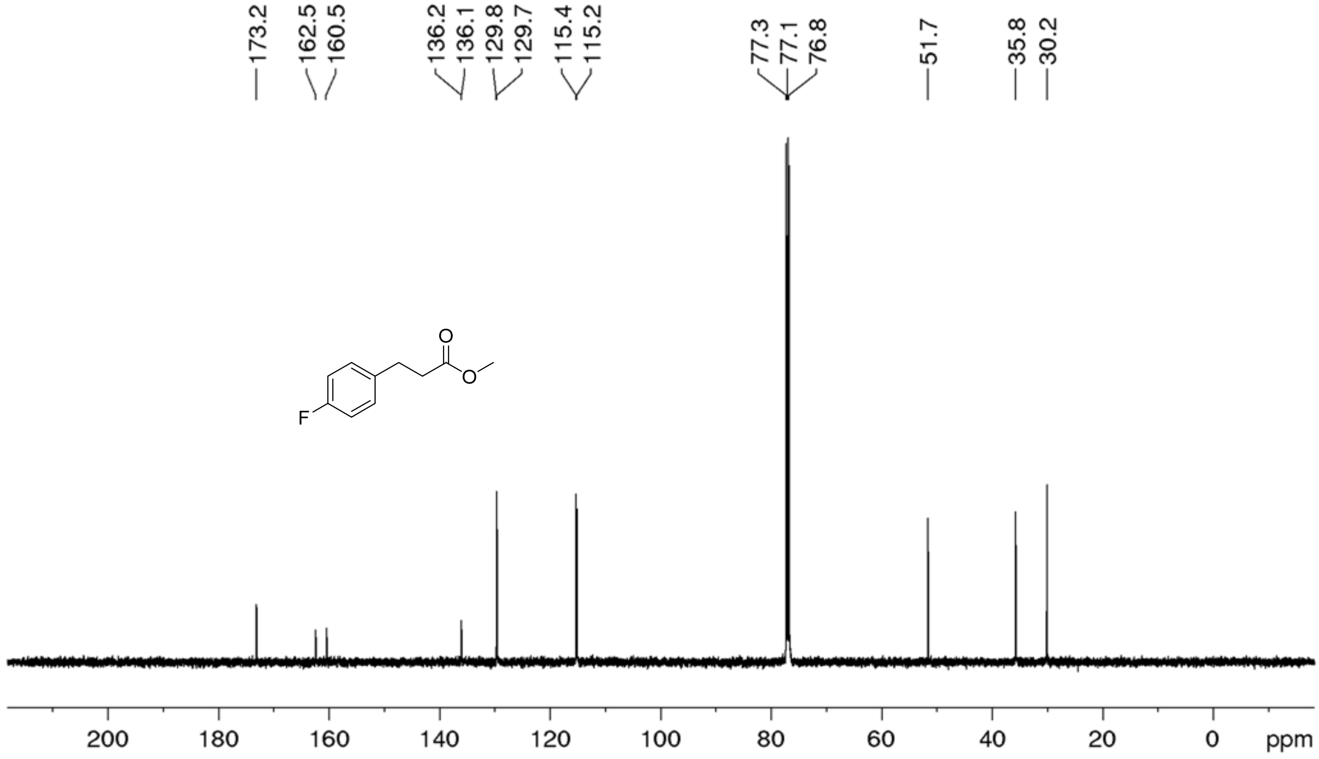

^{13}C NMR of compound **34**

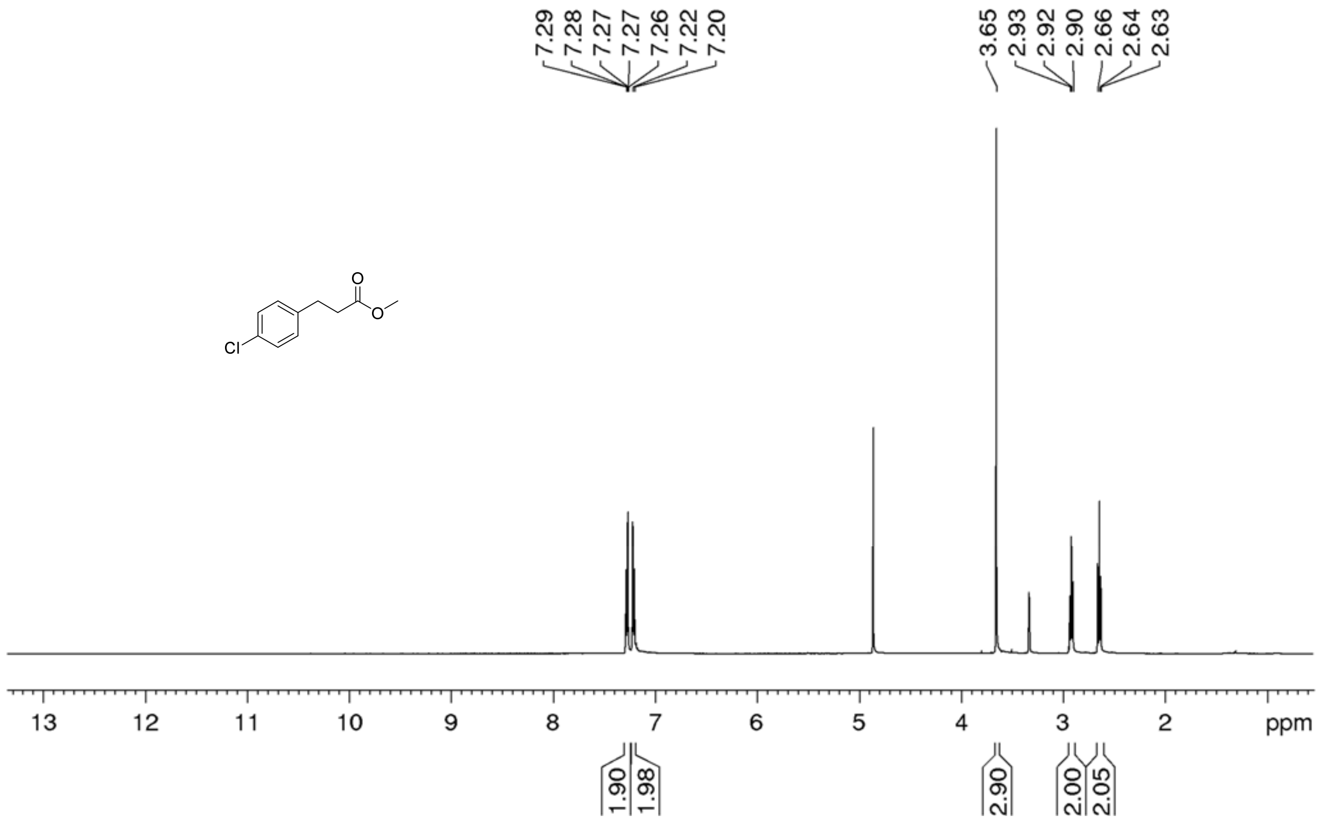

^{1}H NMR of compound **35**

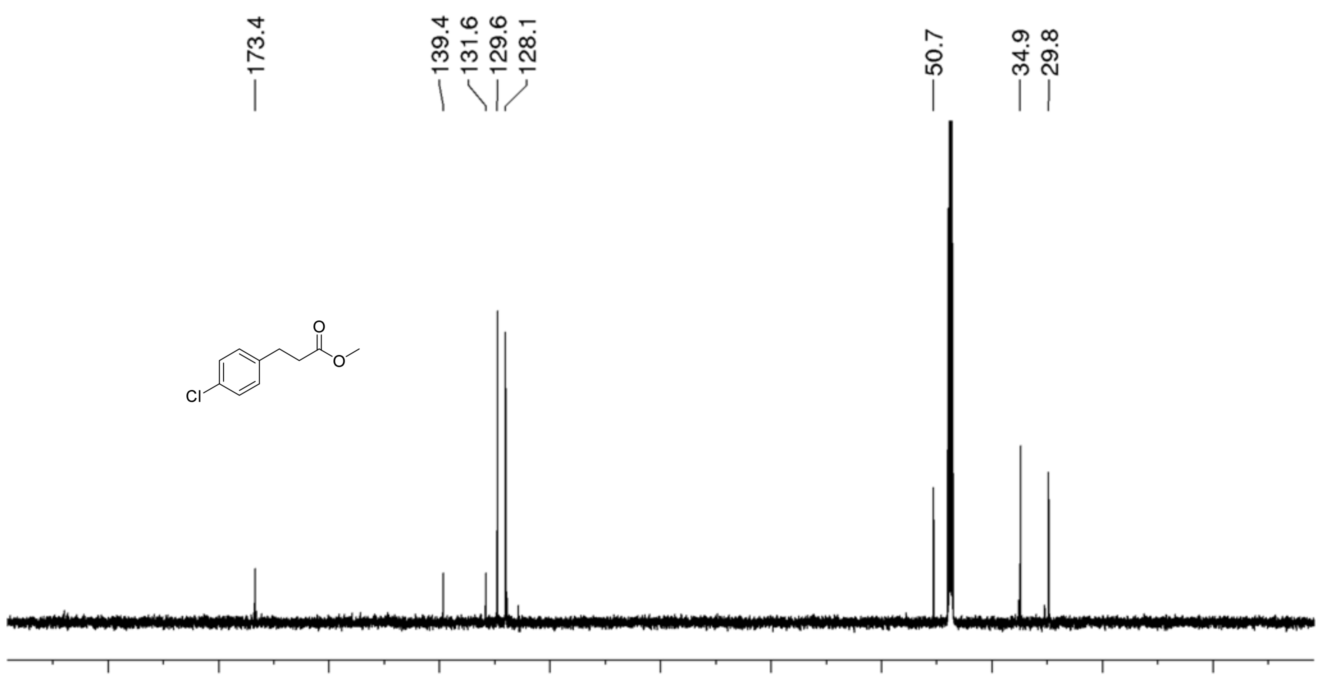

^{13}C NMR of compound **35**

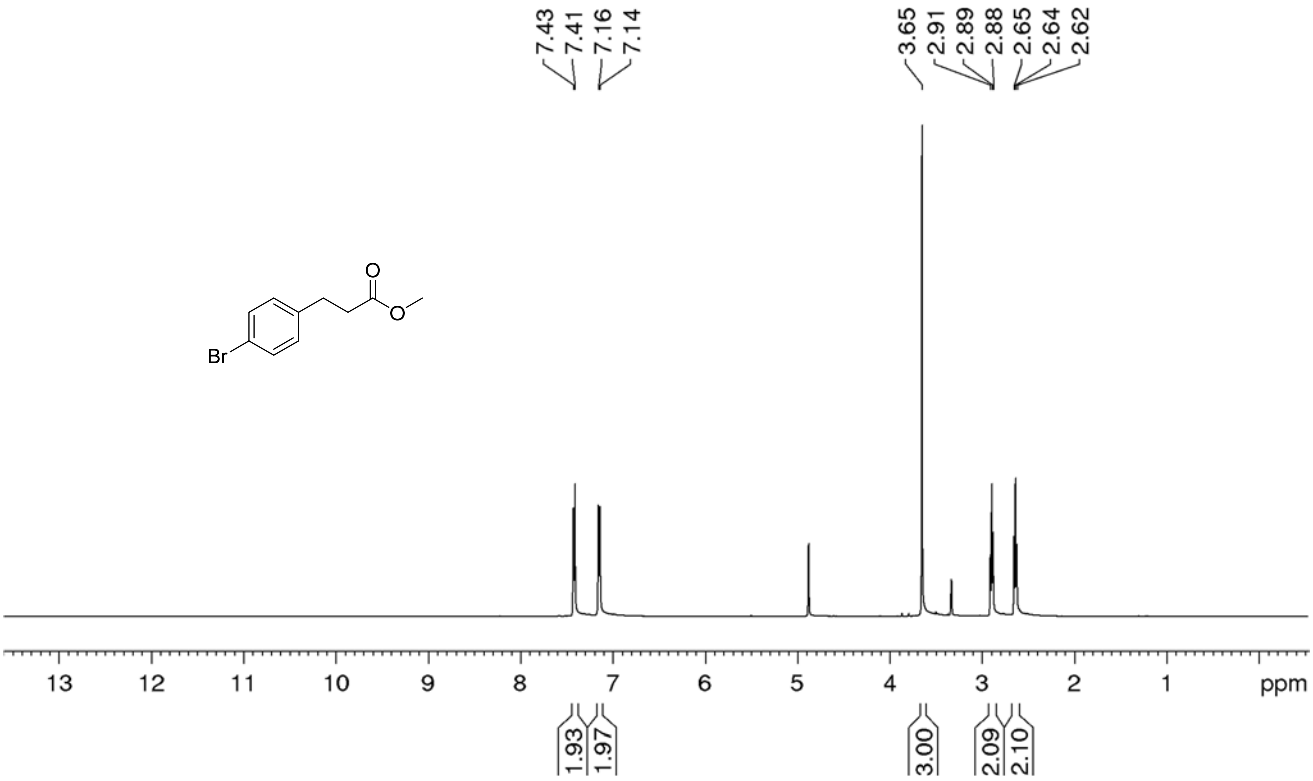

^{1}H NMR of compound **36**

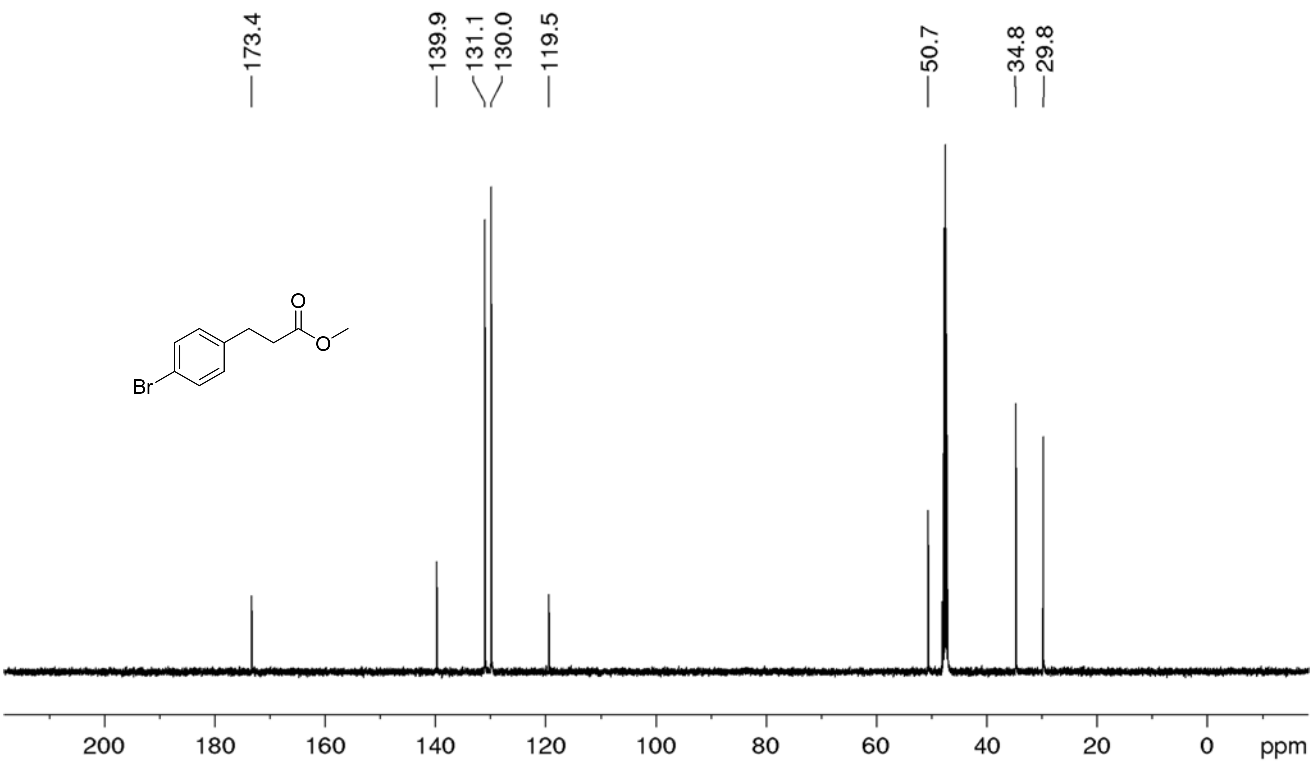

^{13}C NMR of compound **36**

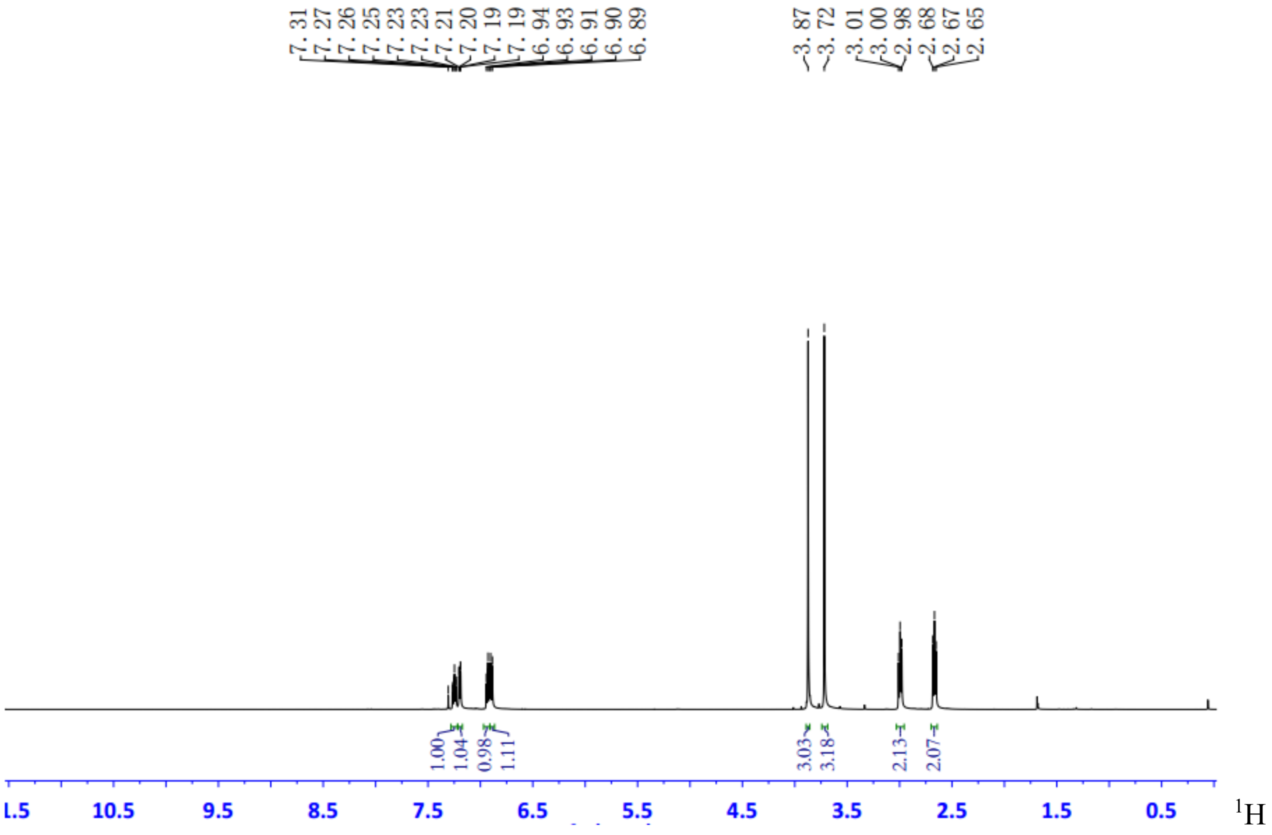

NMR of compound **37**

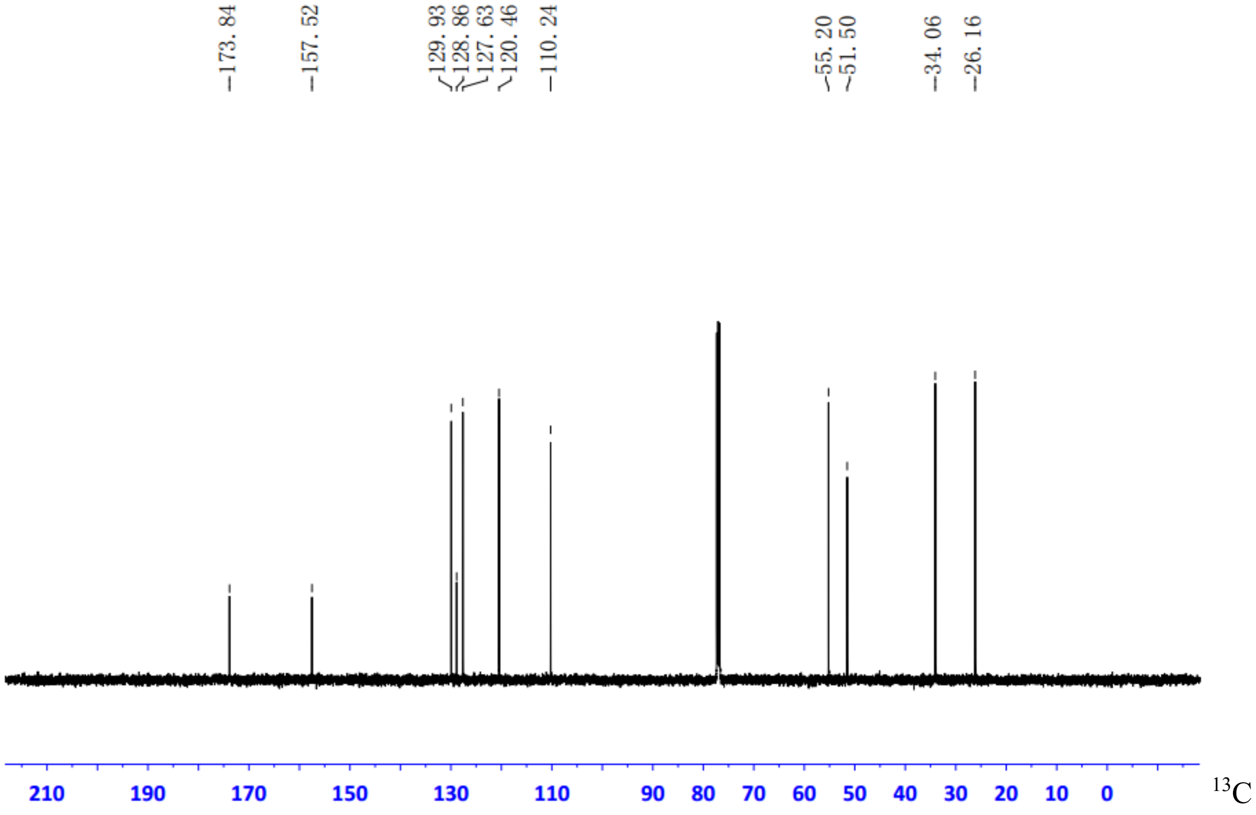

NMR of compound **37**

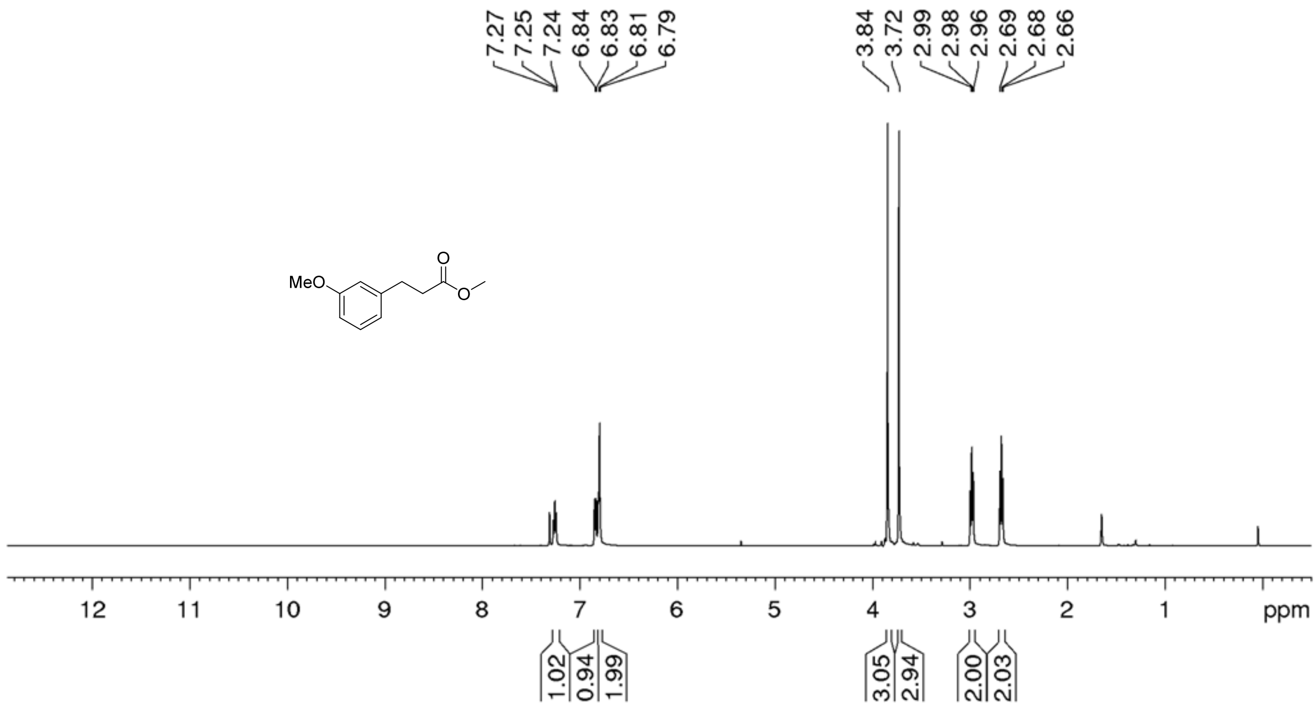

^{1}H NMR of compound **38**

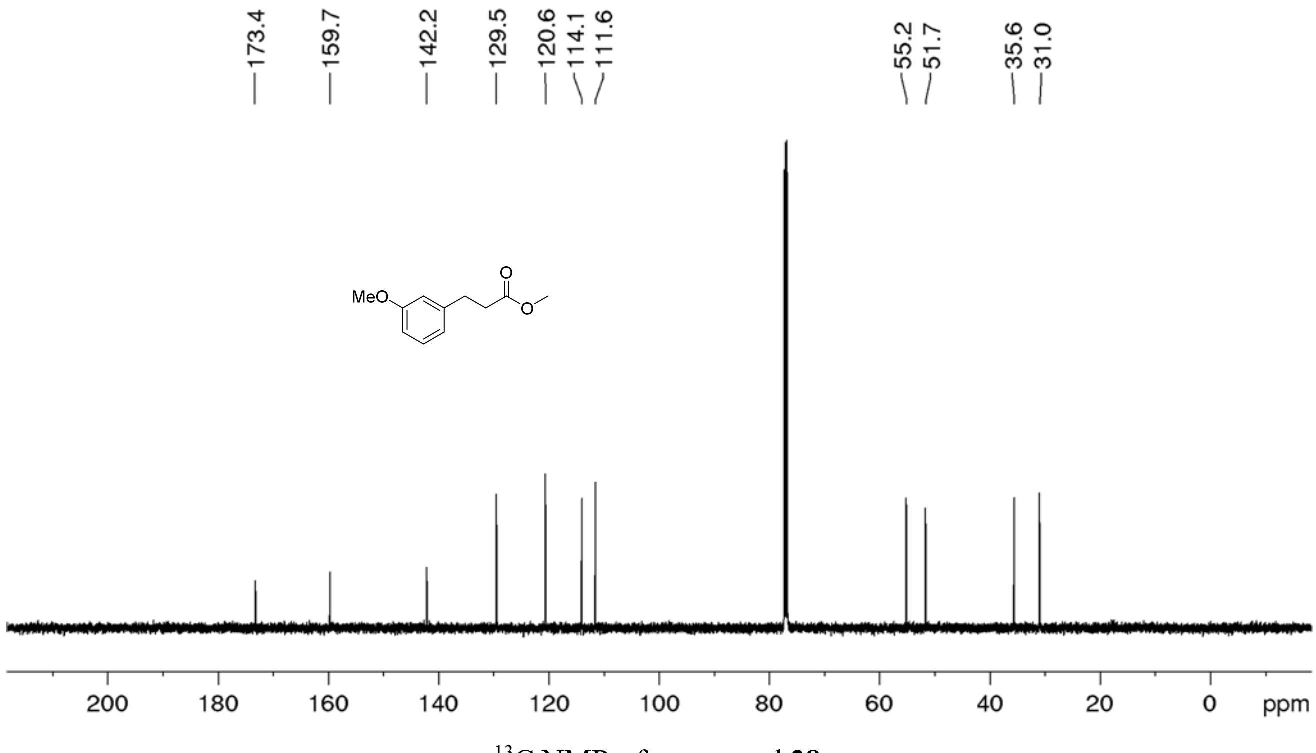

^{13}C NMR of compound **38**

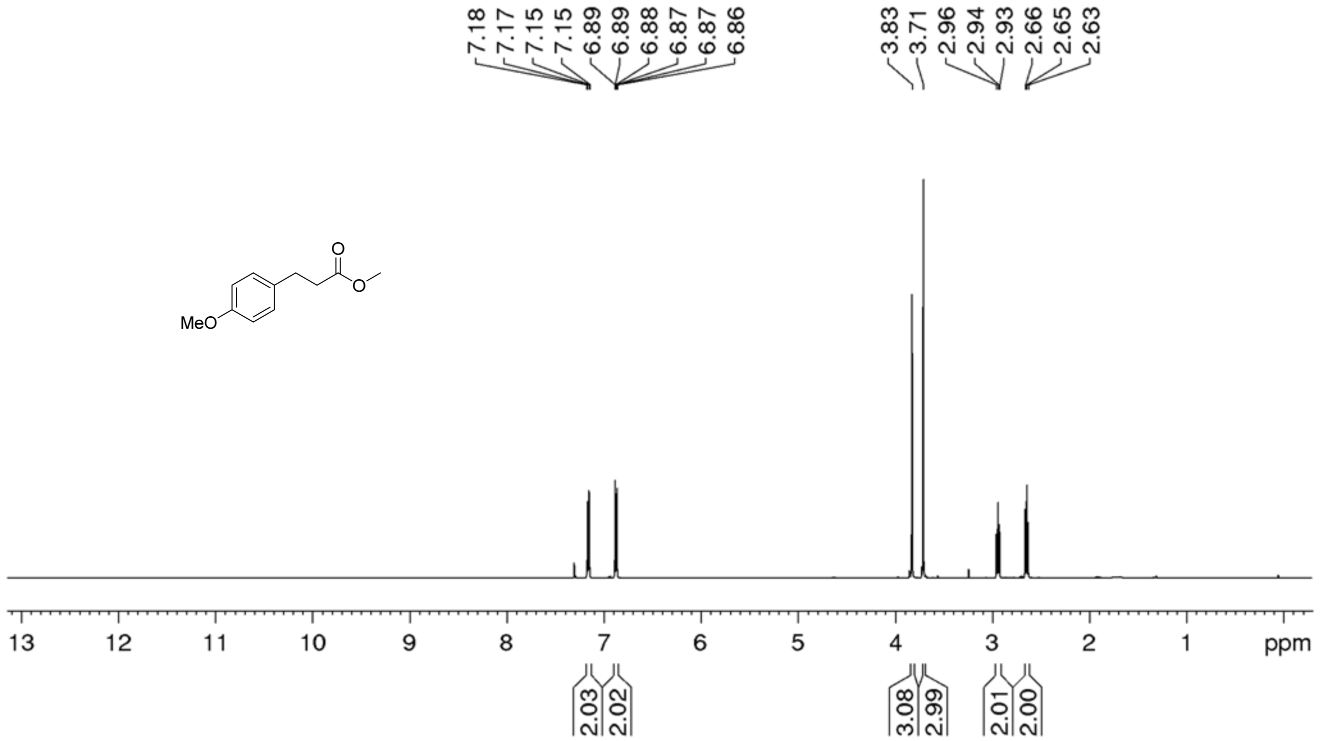

^{1}H NMR of compound **39**

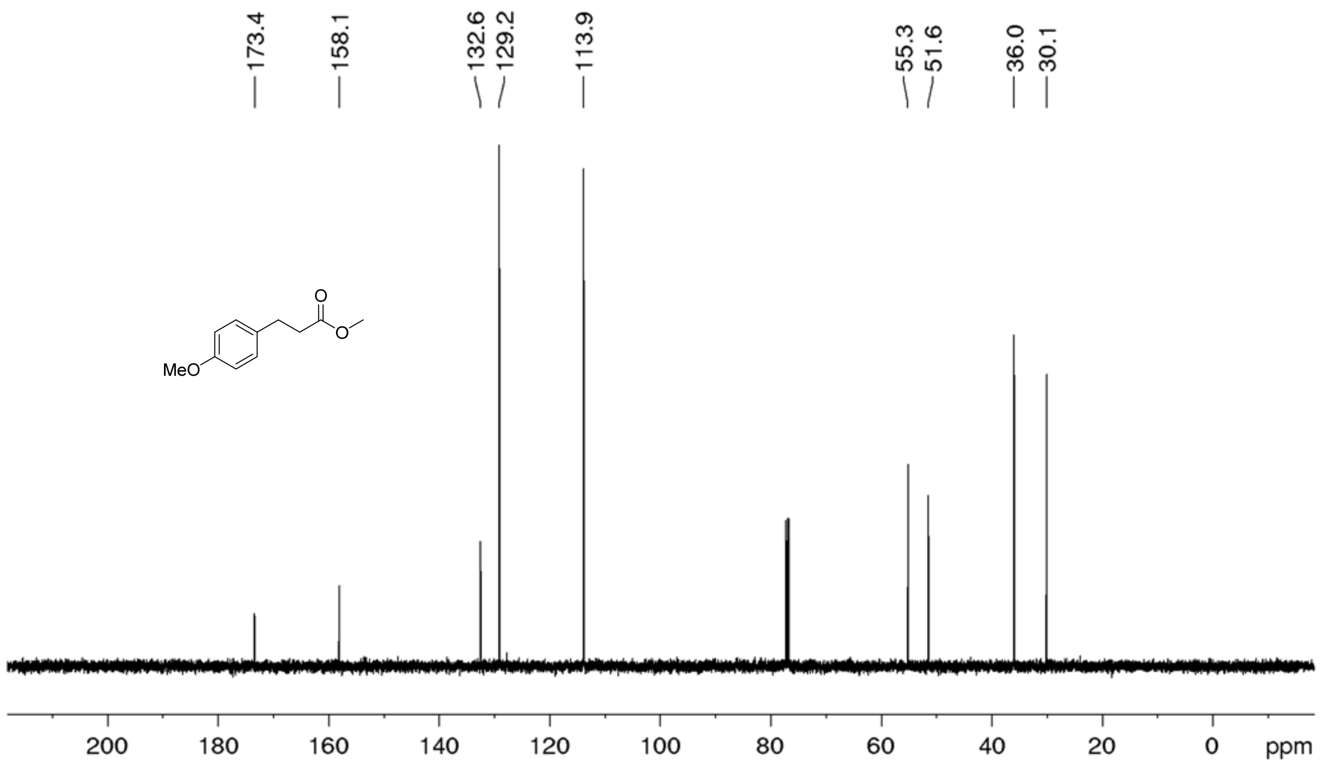

^{13}C NMR of compound **39**

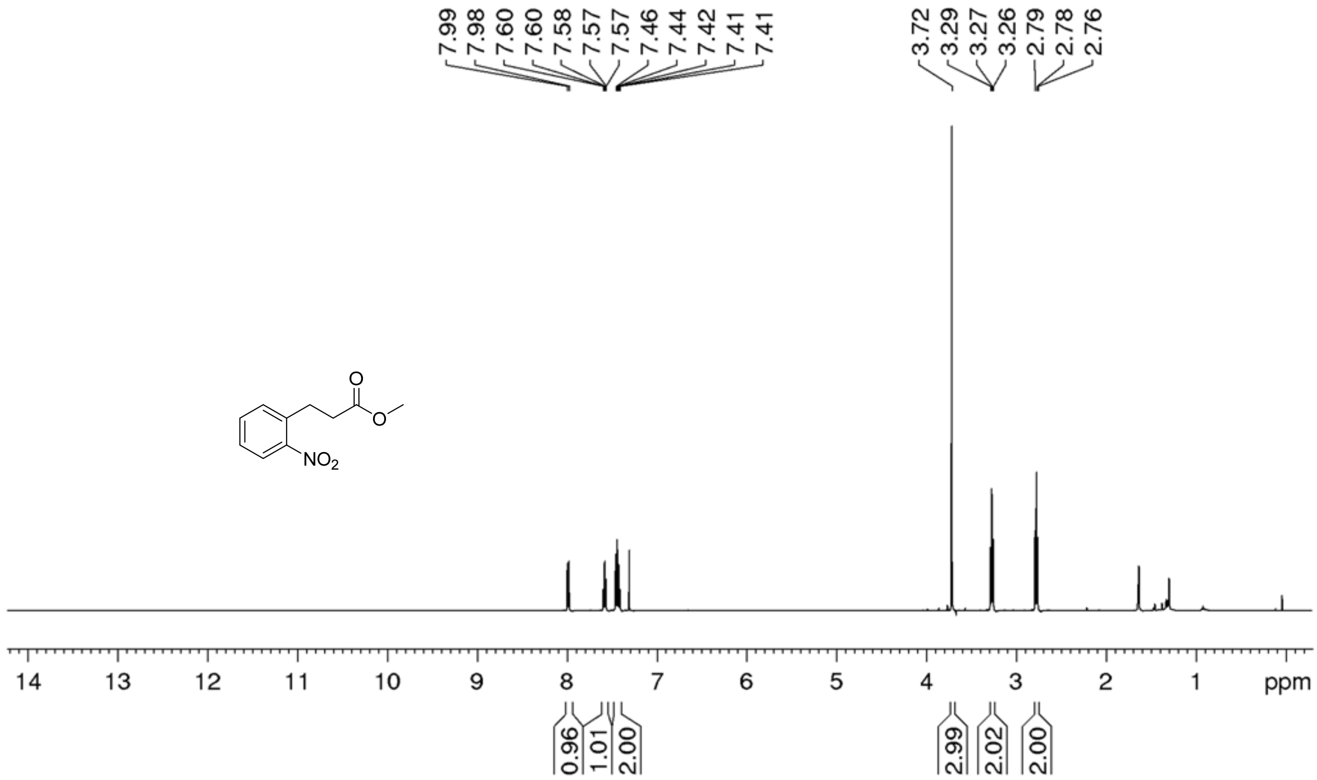

^{1}H NMR of compound **40**

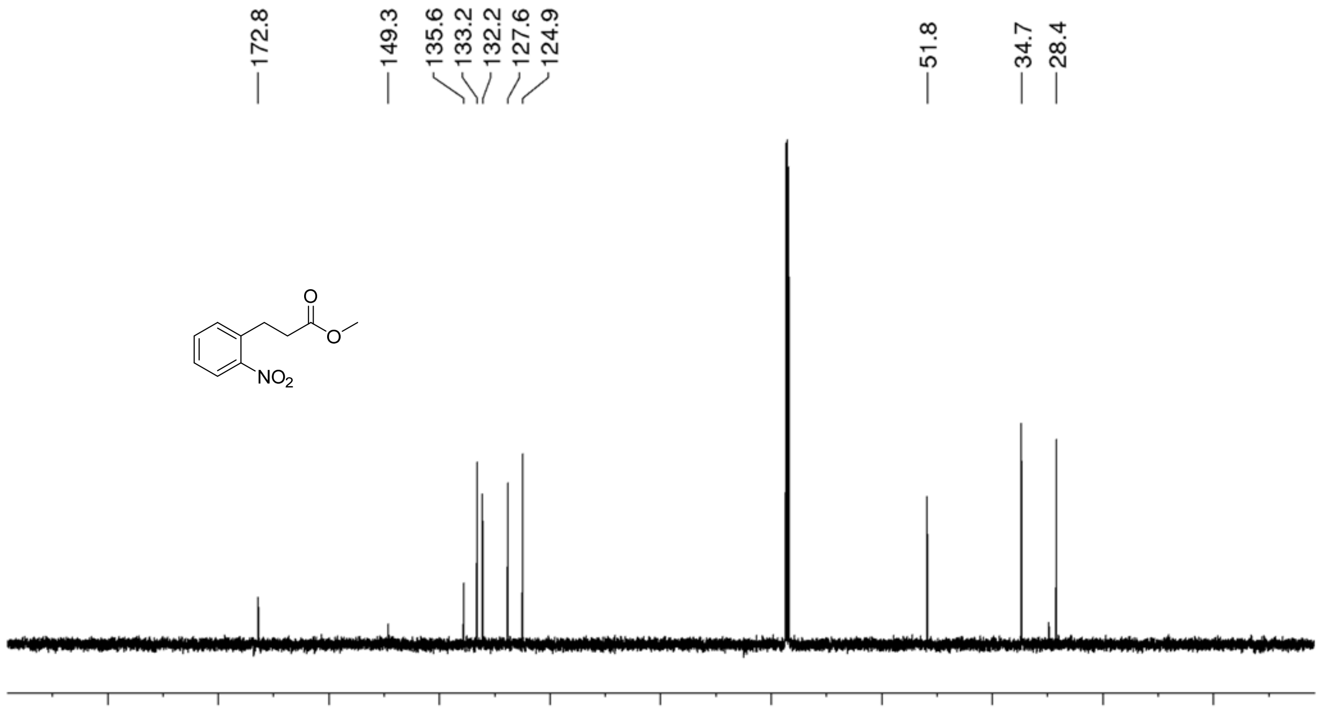

^{13}C NMR of compound **40**

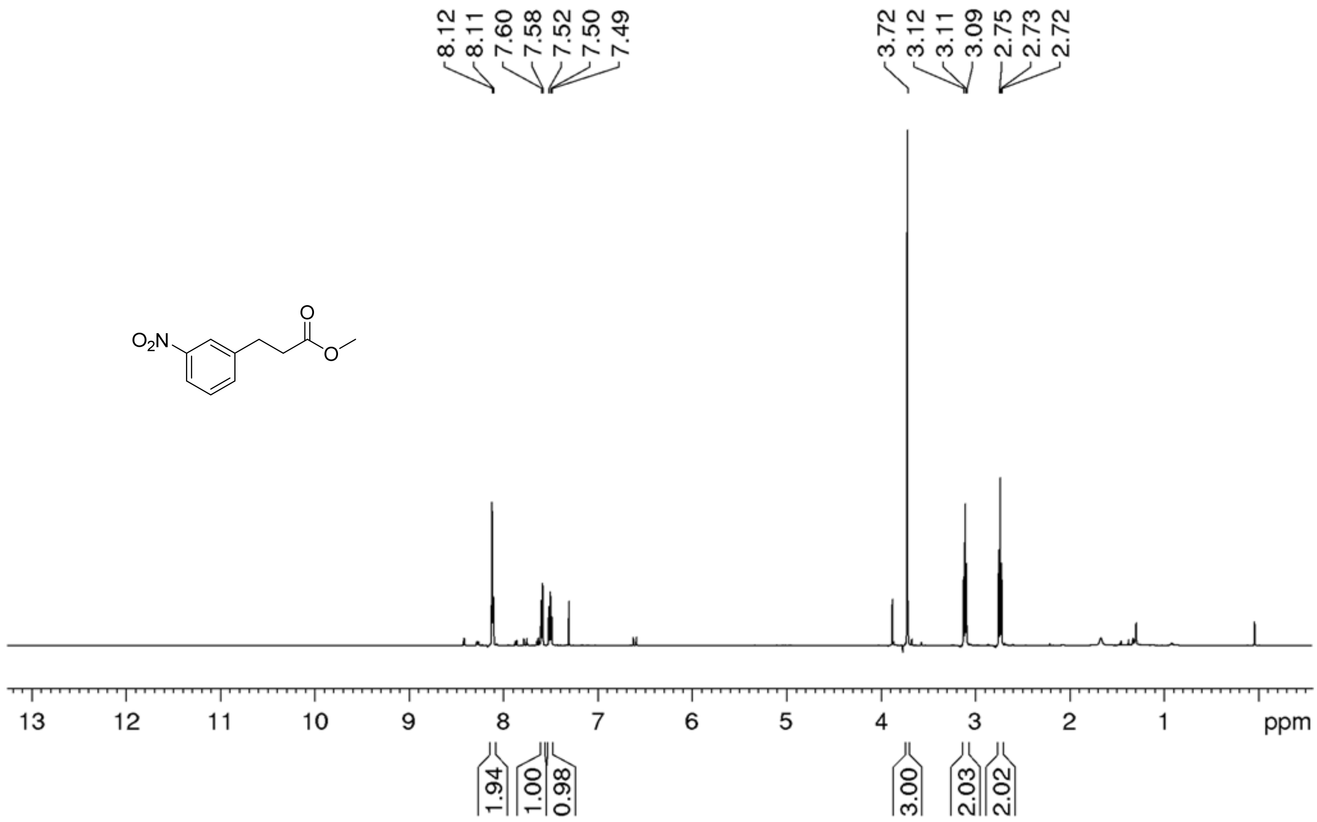

^{1}H NMR of compound **41**

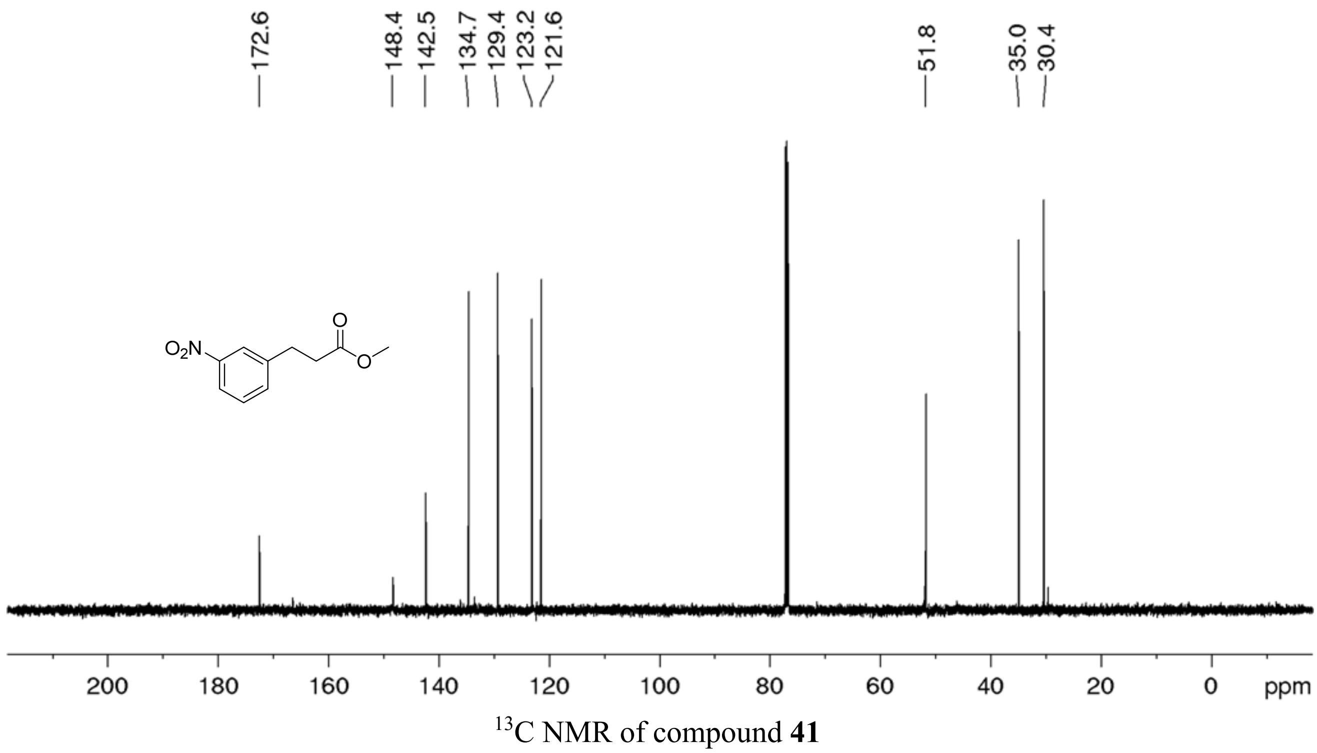

^{13}C NMR of compound **41**

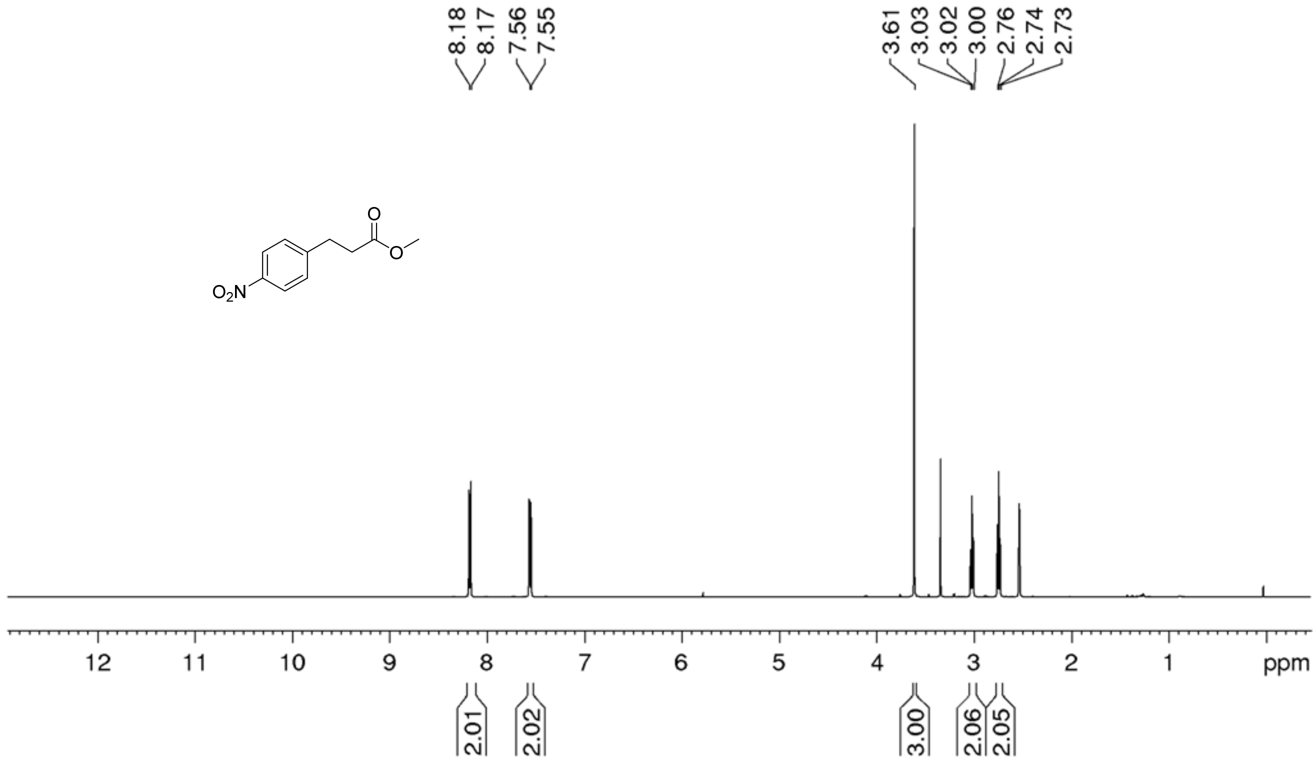

^{1}H NMR of compound **42**

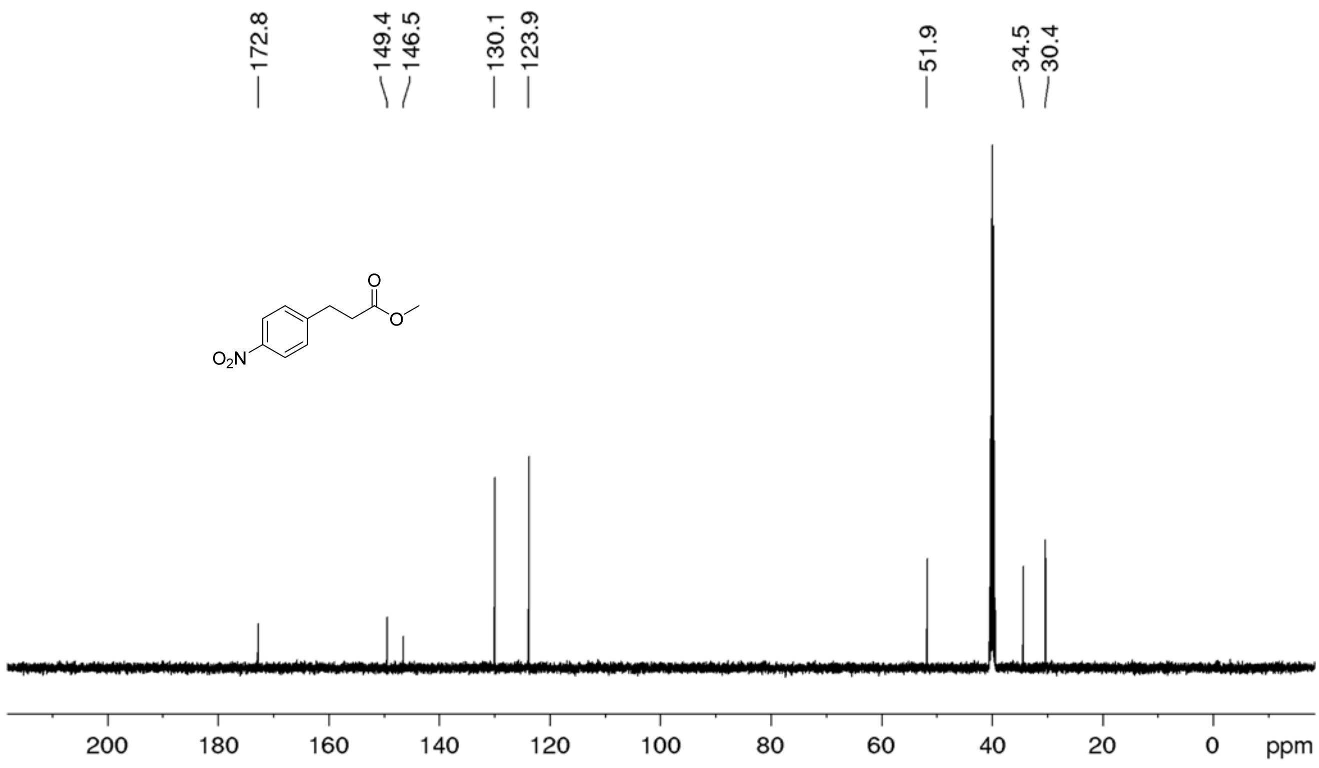

^{13}C NMR of compound **42**

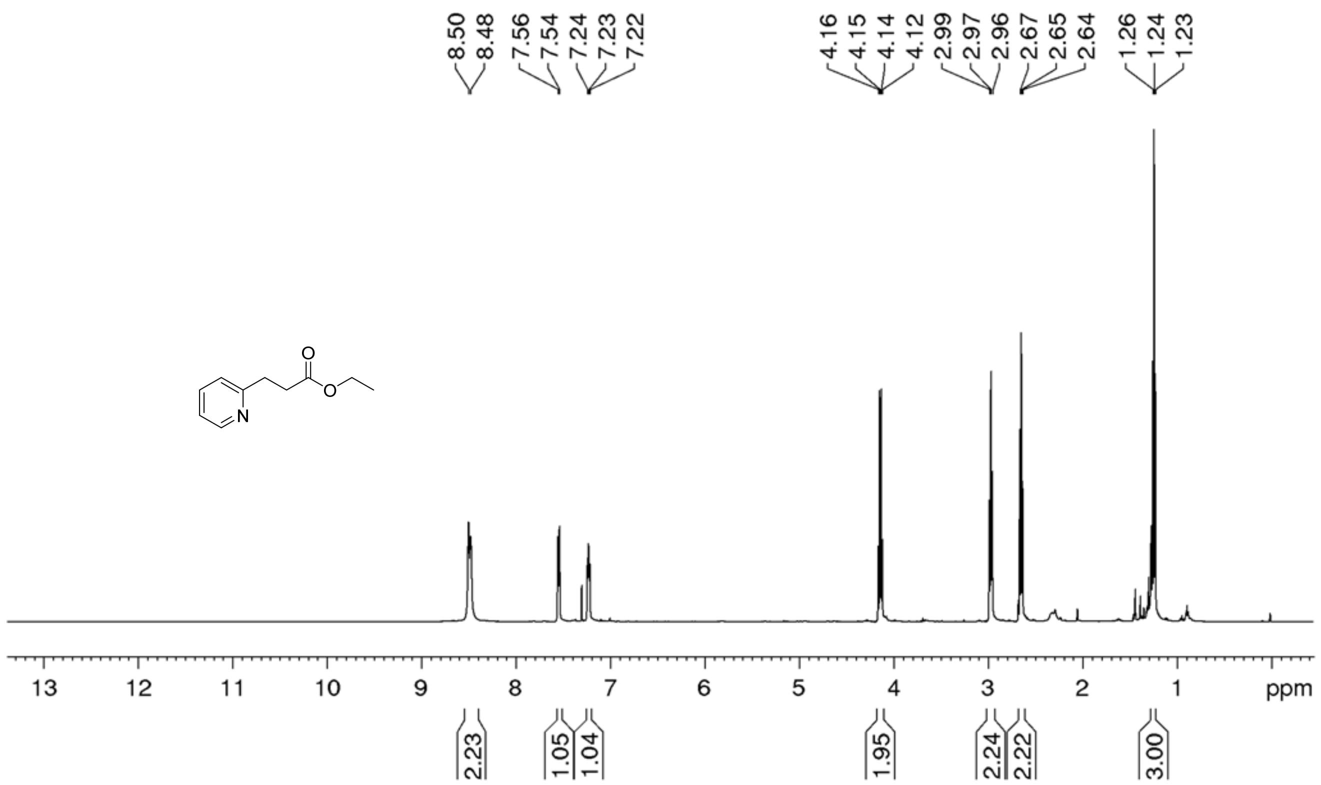

^{1}H NMR of compound **43**

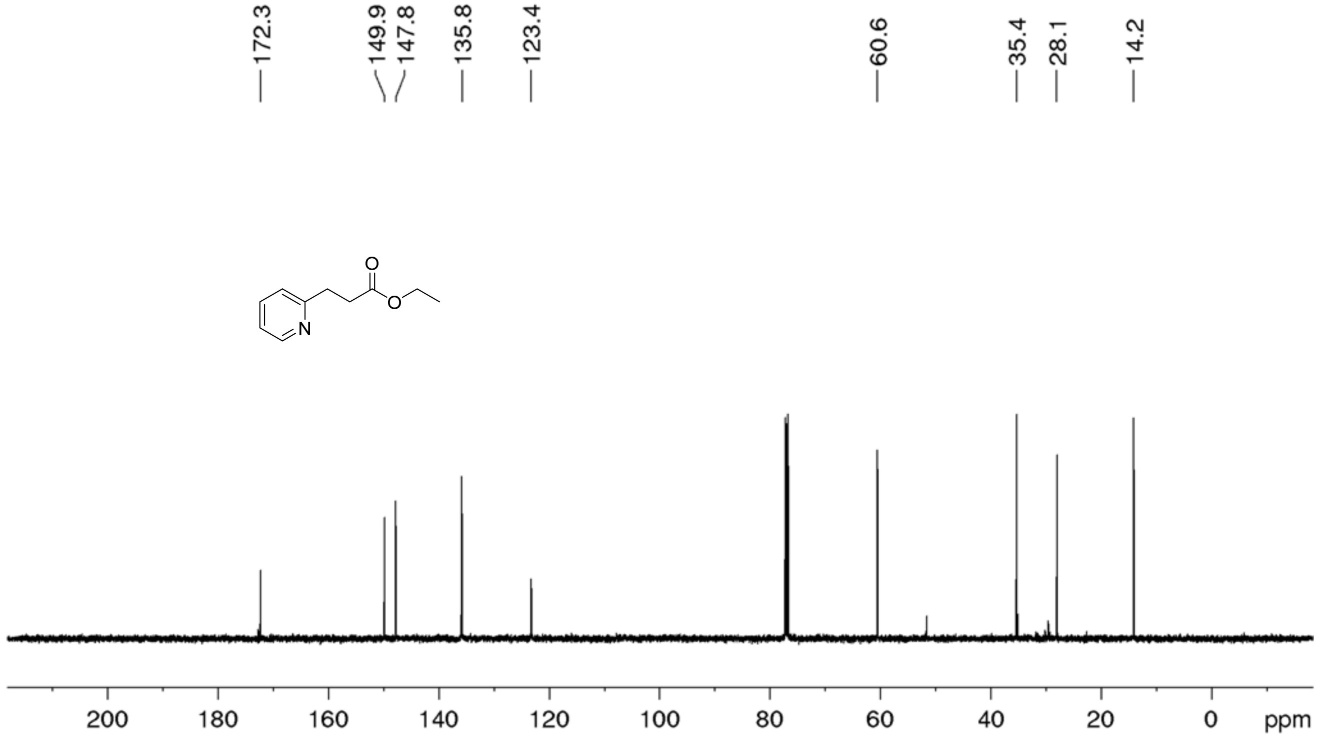

^{13}C NMR of compound **43**

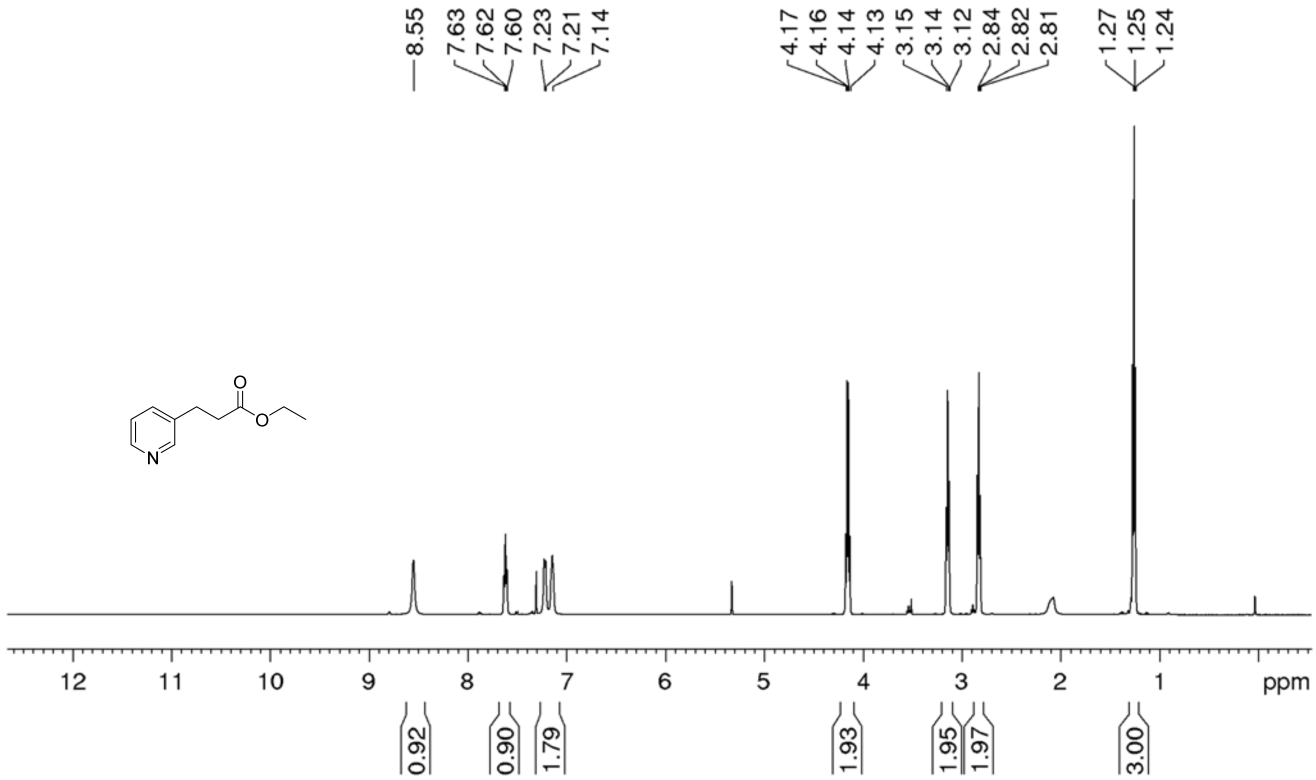

^{1}H NMR of compound **44**

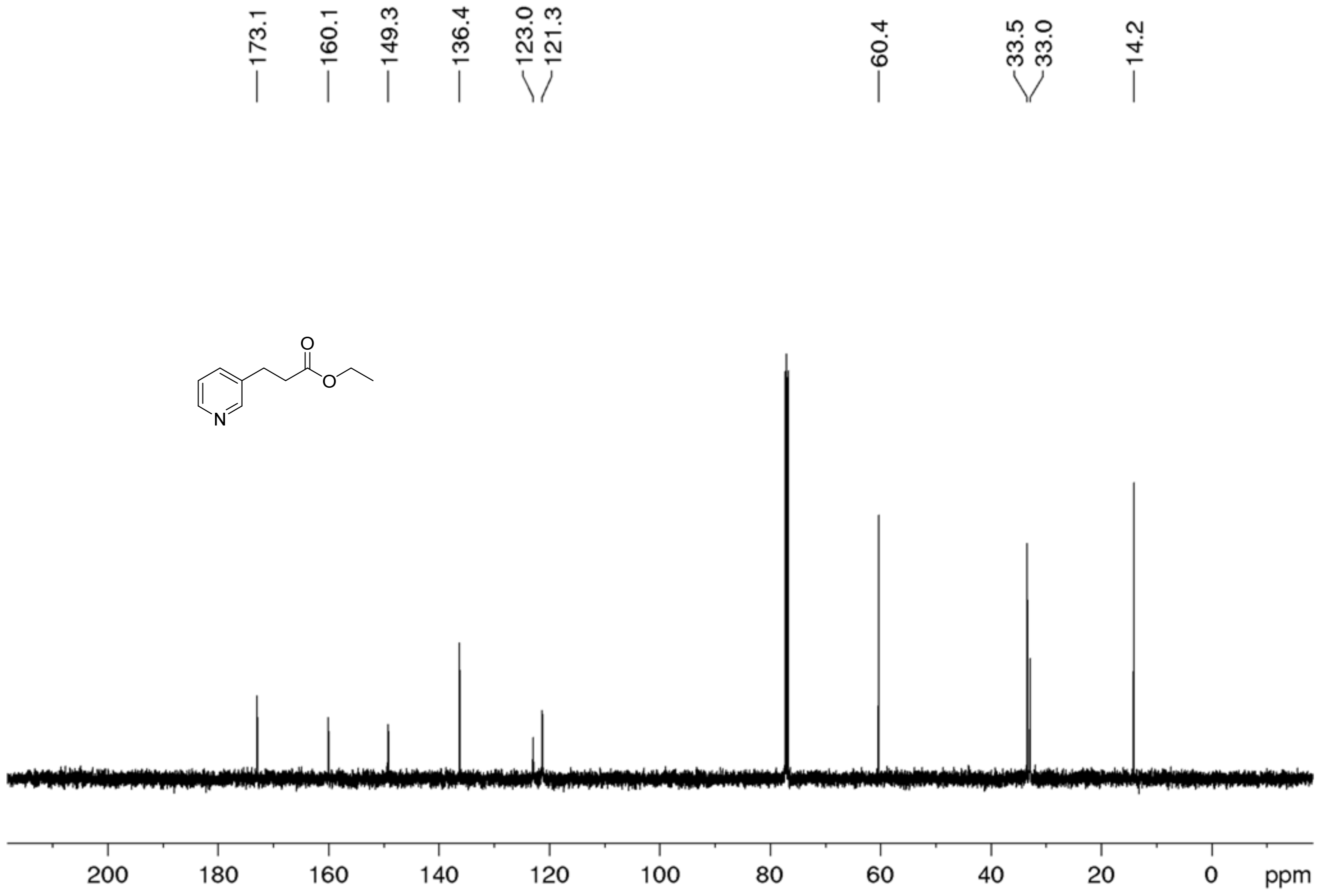

^{13}C NMR of compound **44**

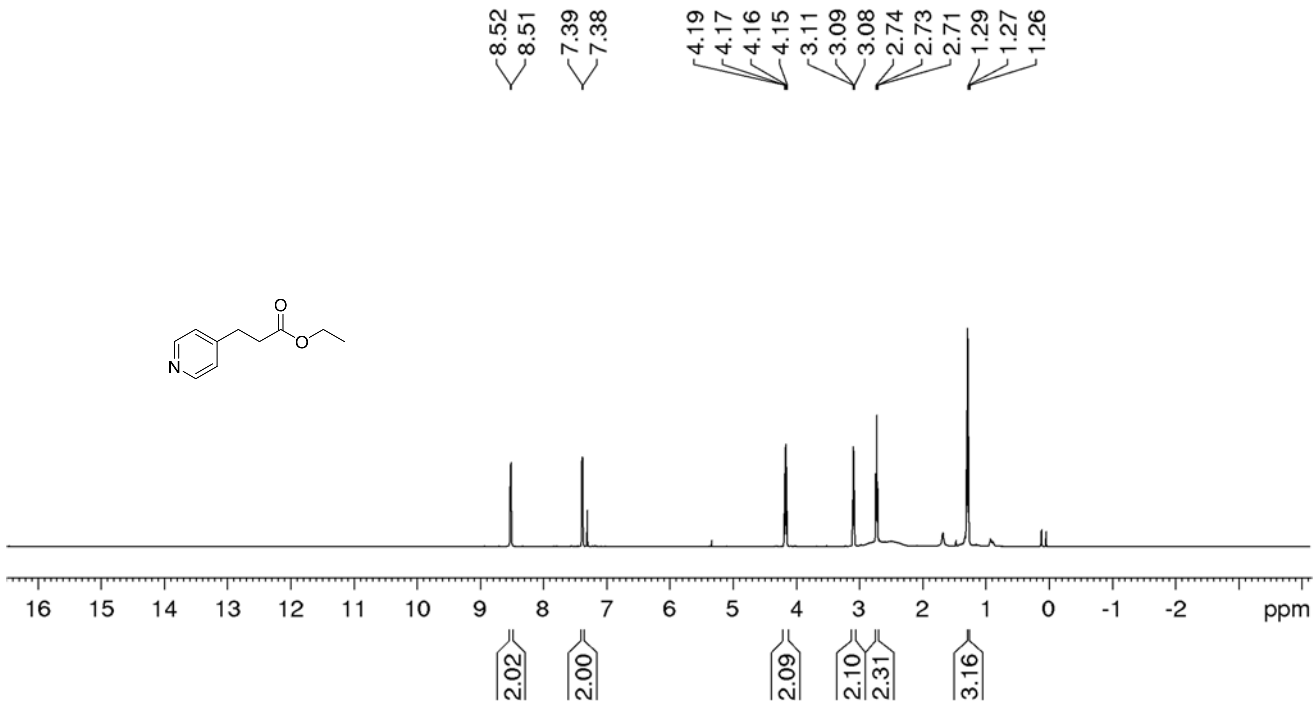

^{1}H NMR of compound **45**

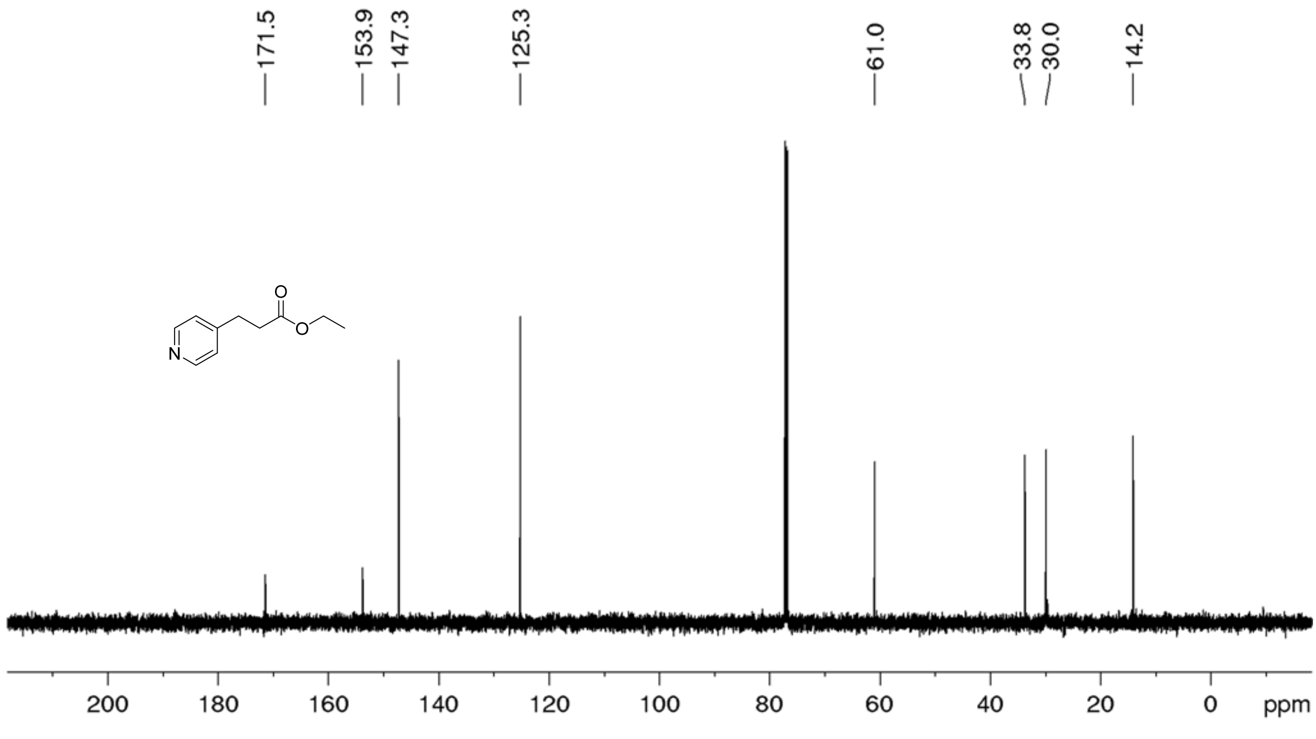

^{13}C NMR of compound **45**

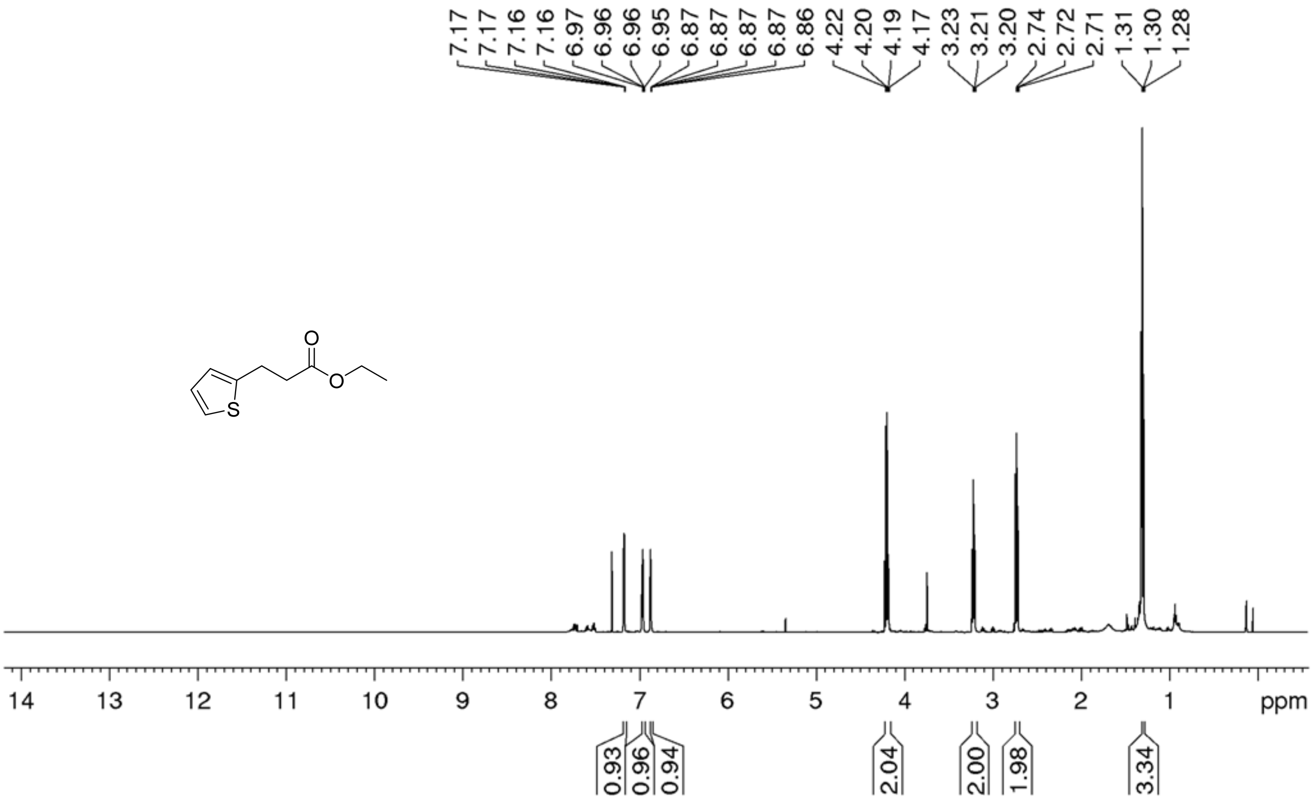

^{1}H NMR of compound **46**

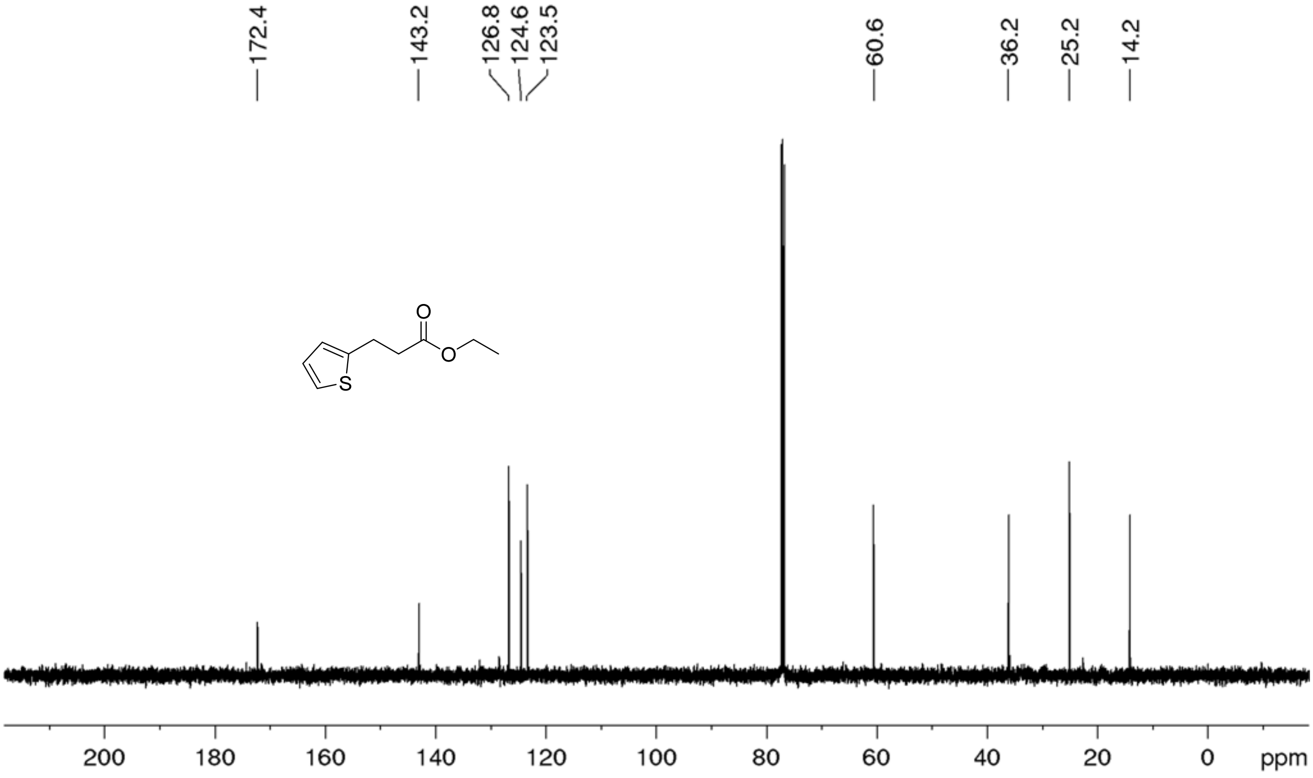

^{13}C NMR of compound **46**

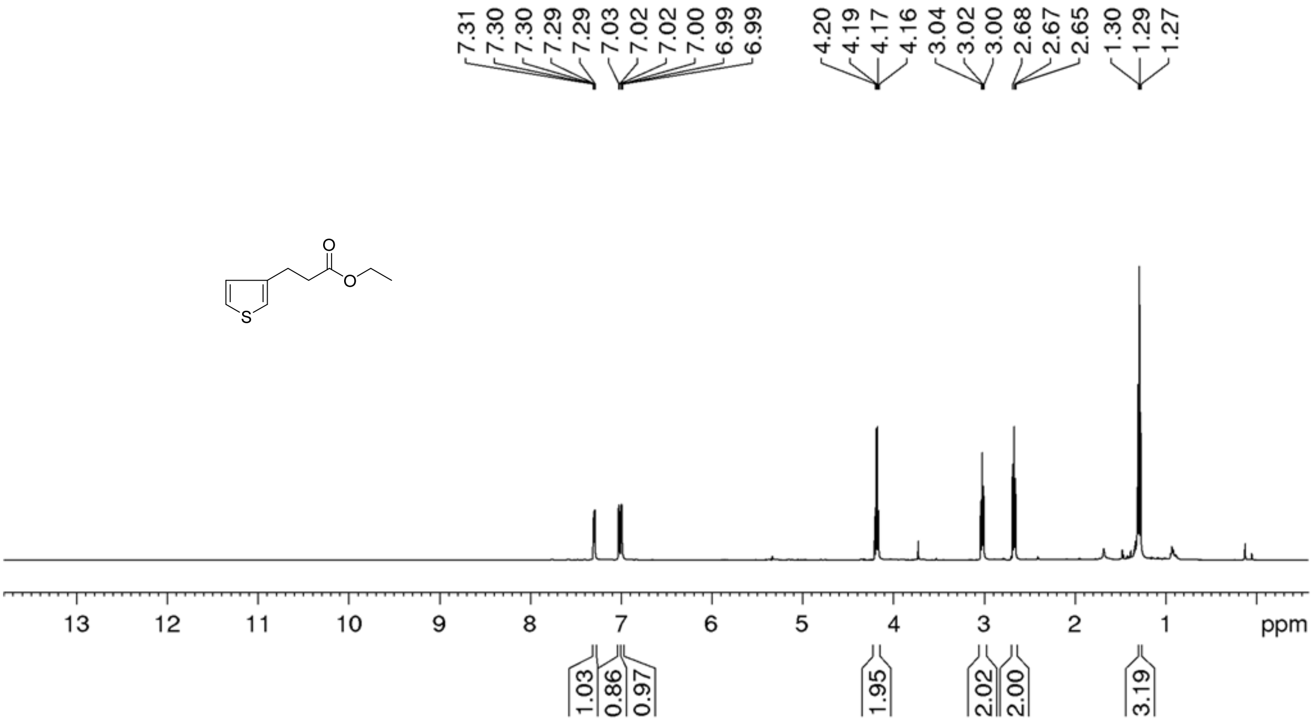

^{1}H NMR of compound **47**

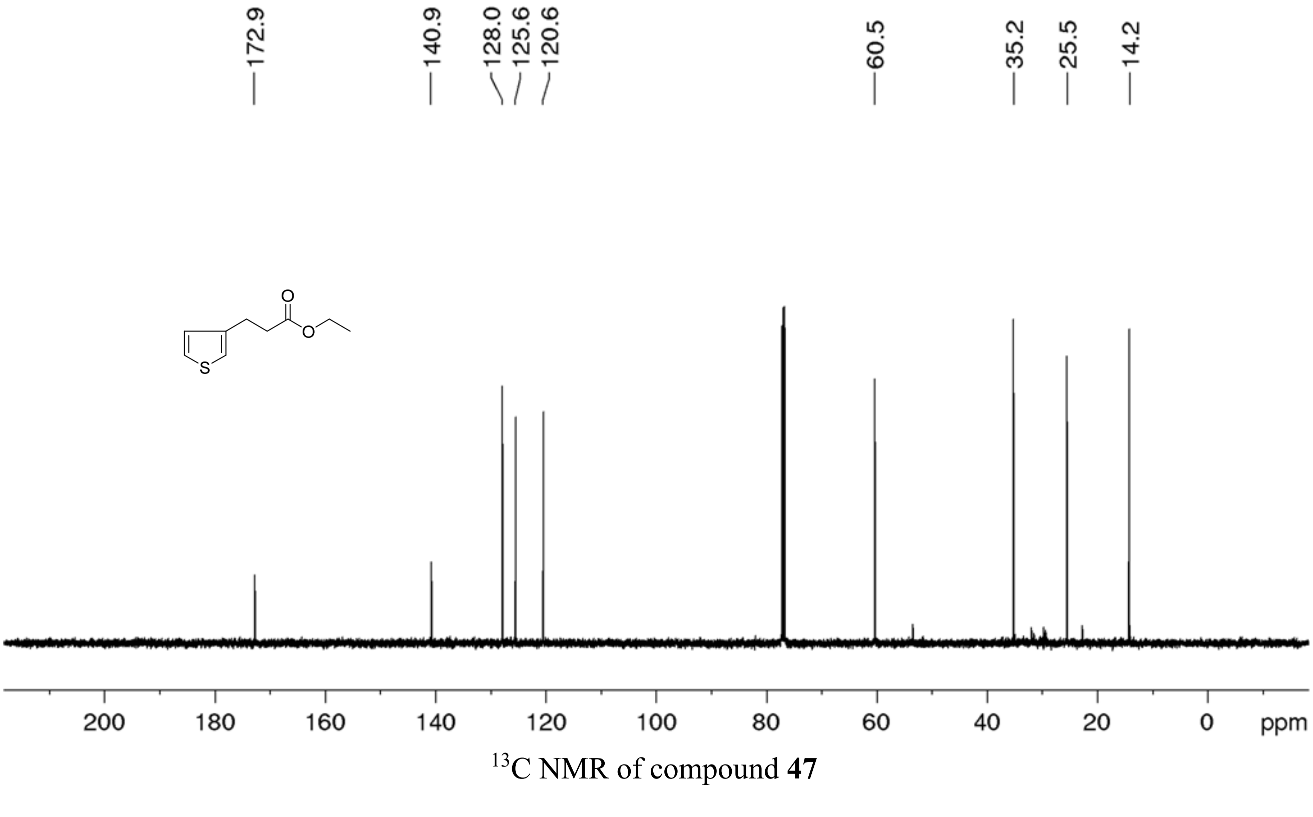

^{13}C NMR of compound **47**

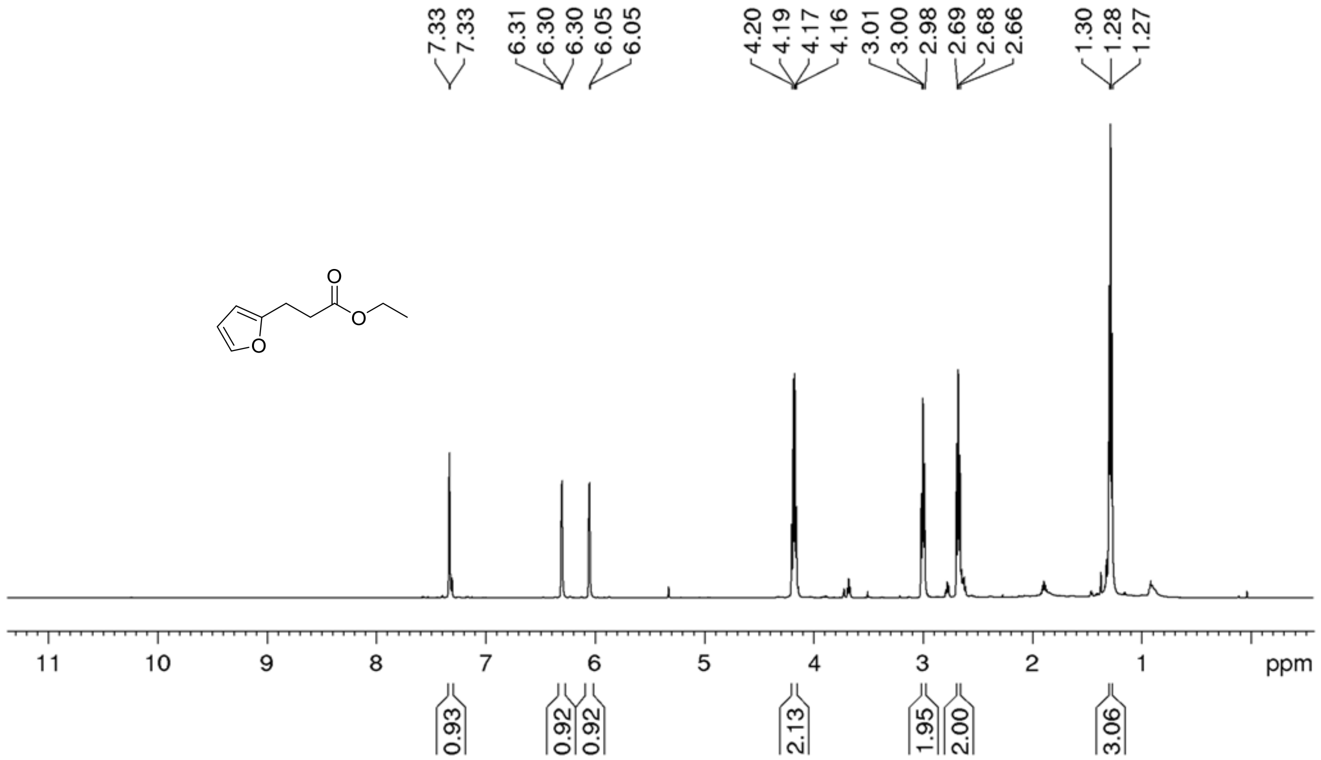

^{1}H NMR of compound **48**

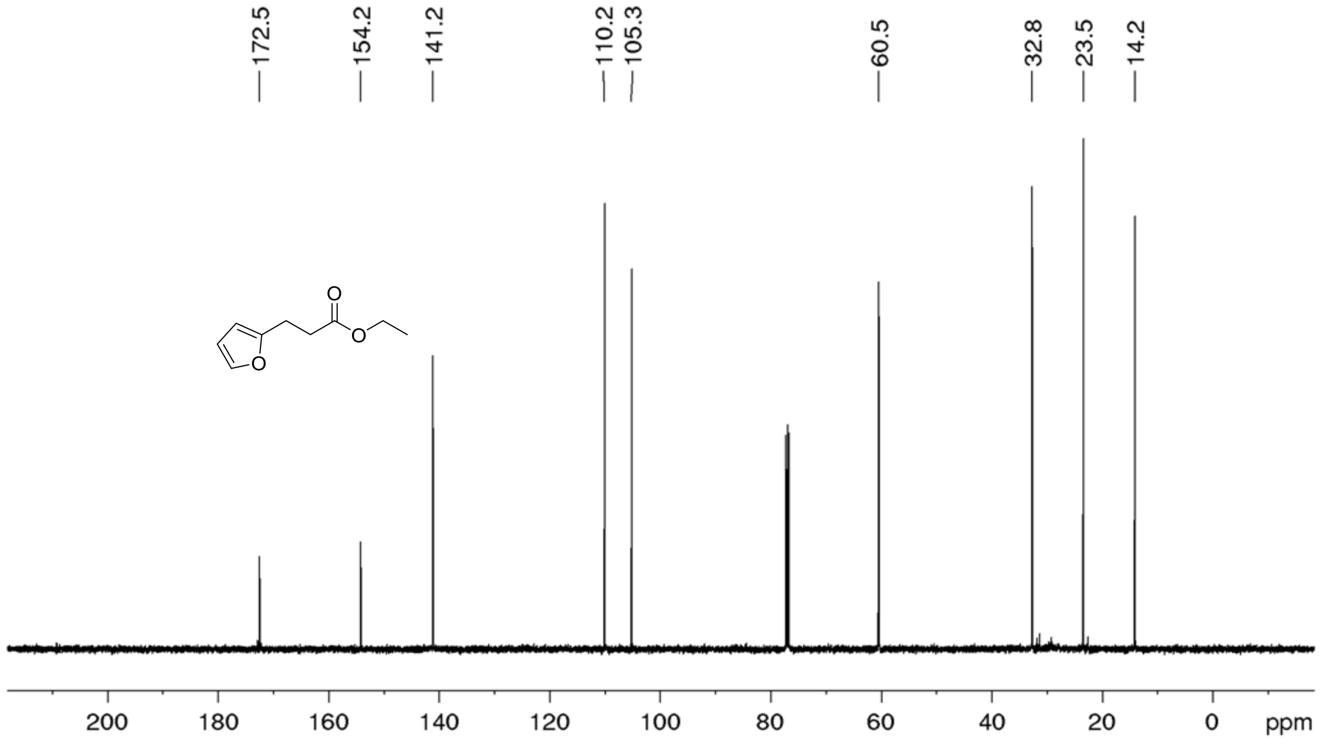

^{13}C NMR of compound **48**

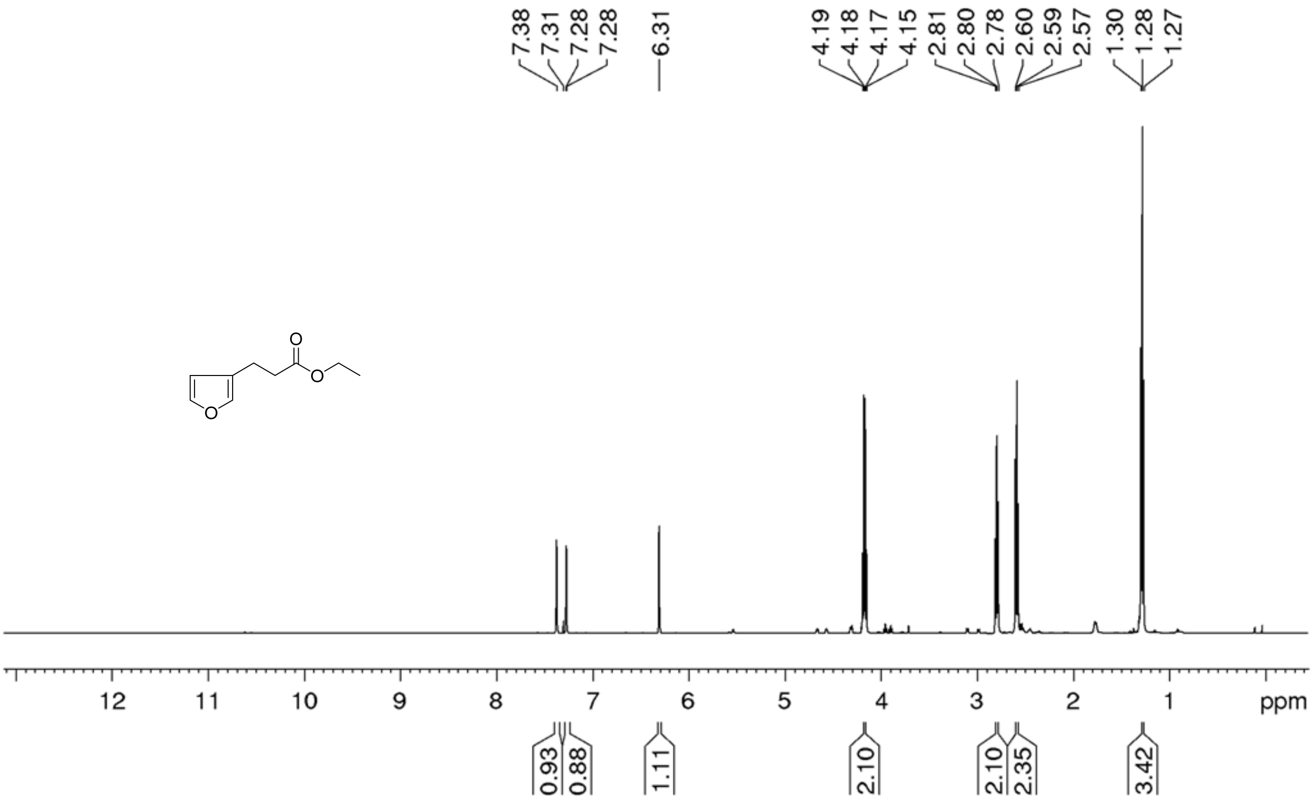

^{1}H NMR of compound **49**

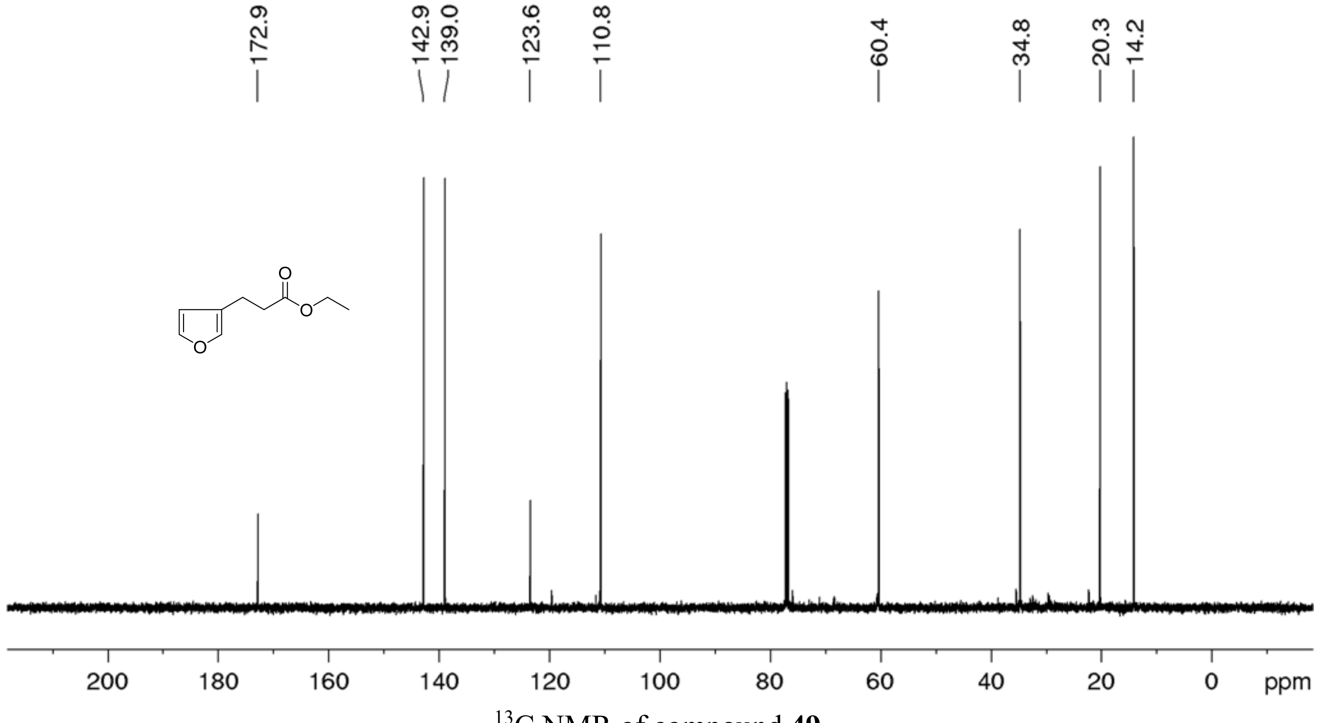

^{13}C NMR of compound **49**

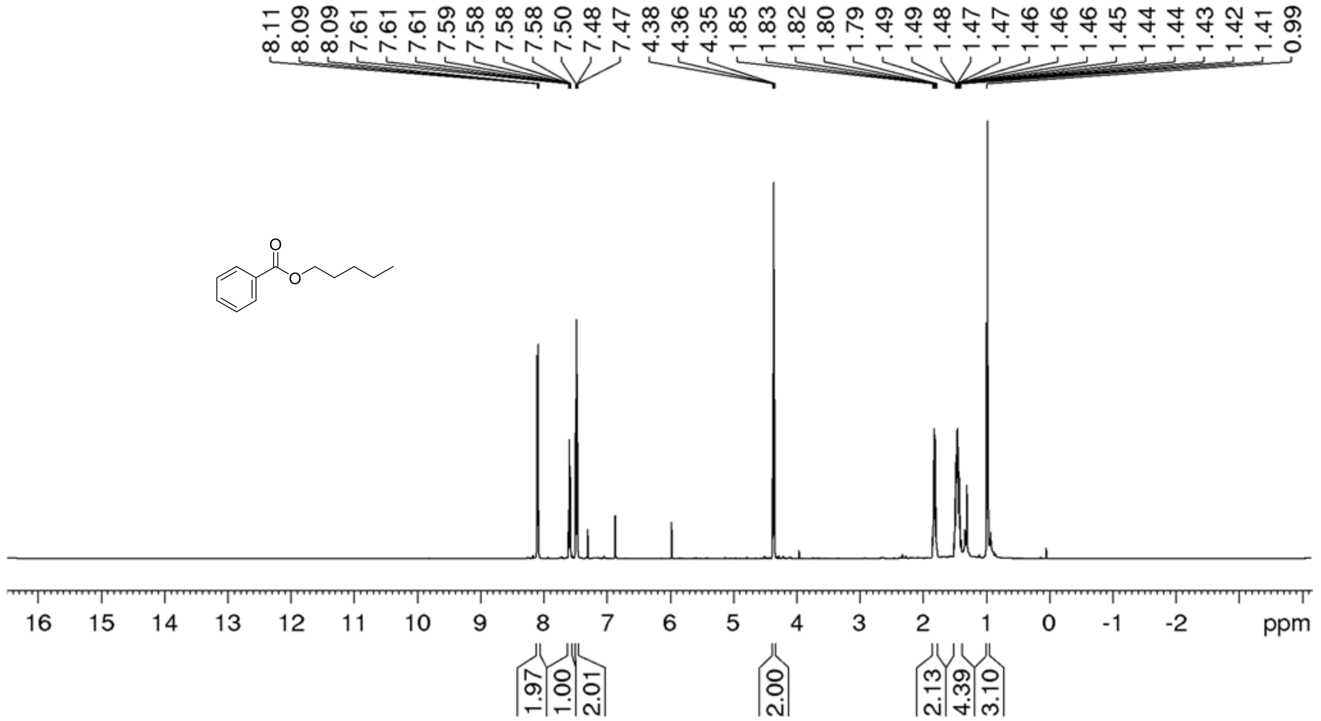

^{1}H NMR of compound **50**

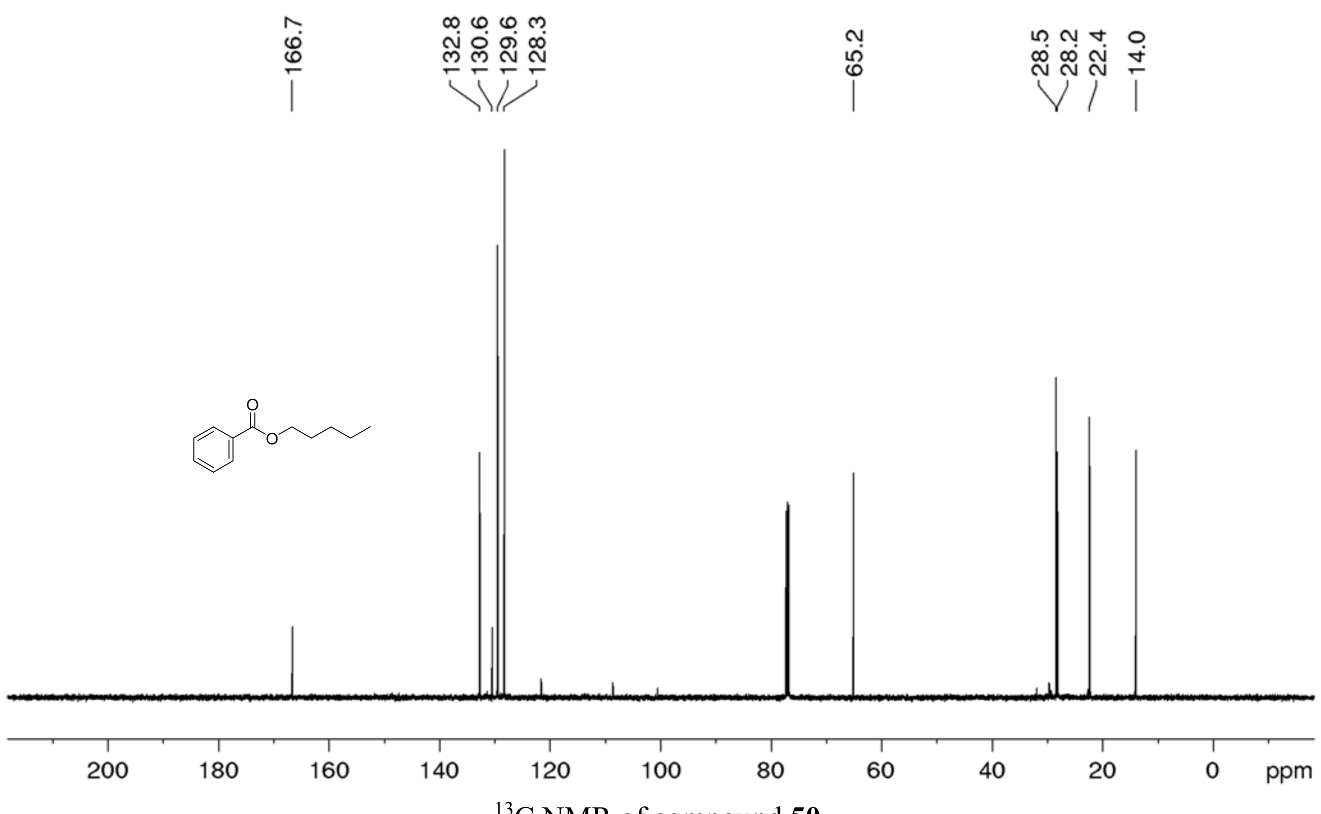

^{13}C NMR of compound **50**

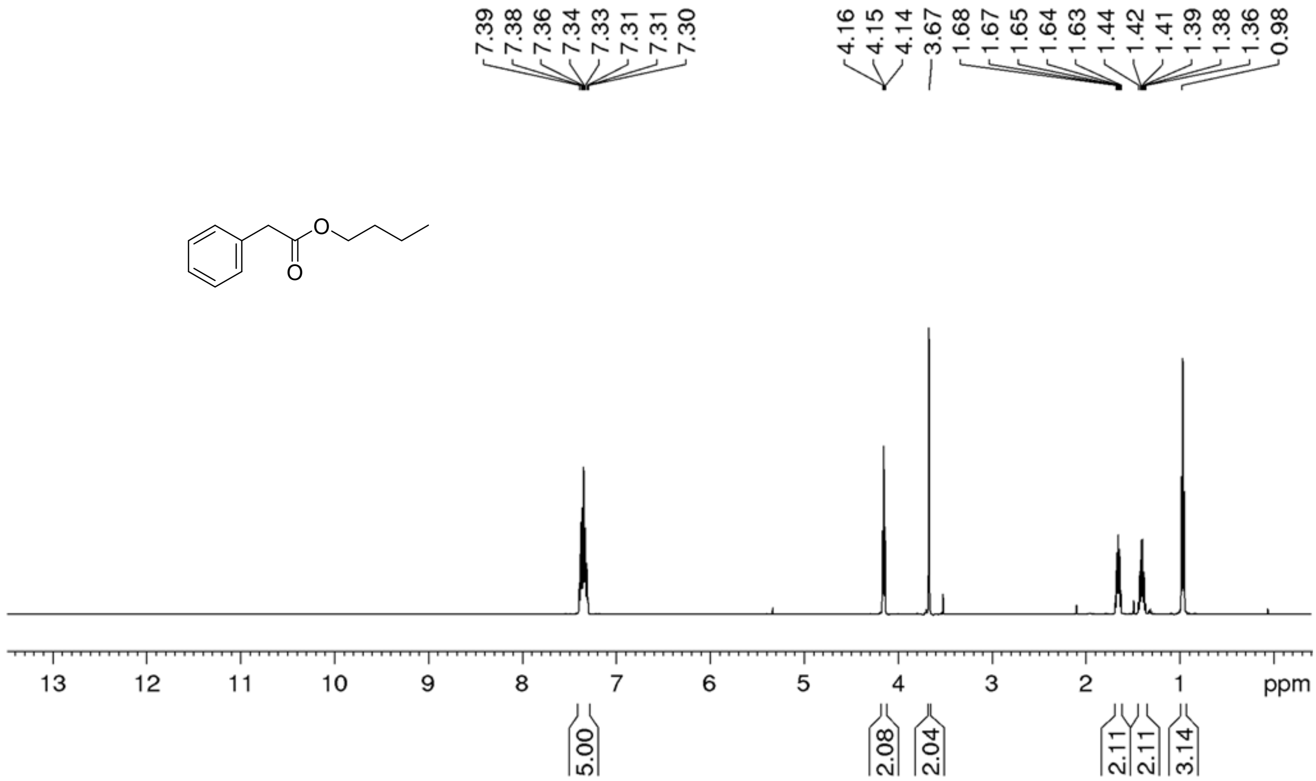

^{1}H NMR of compound **51**

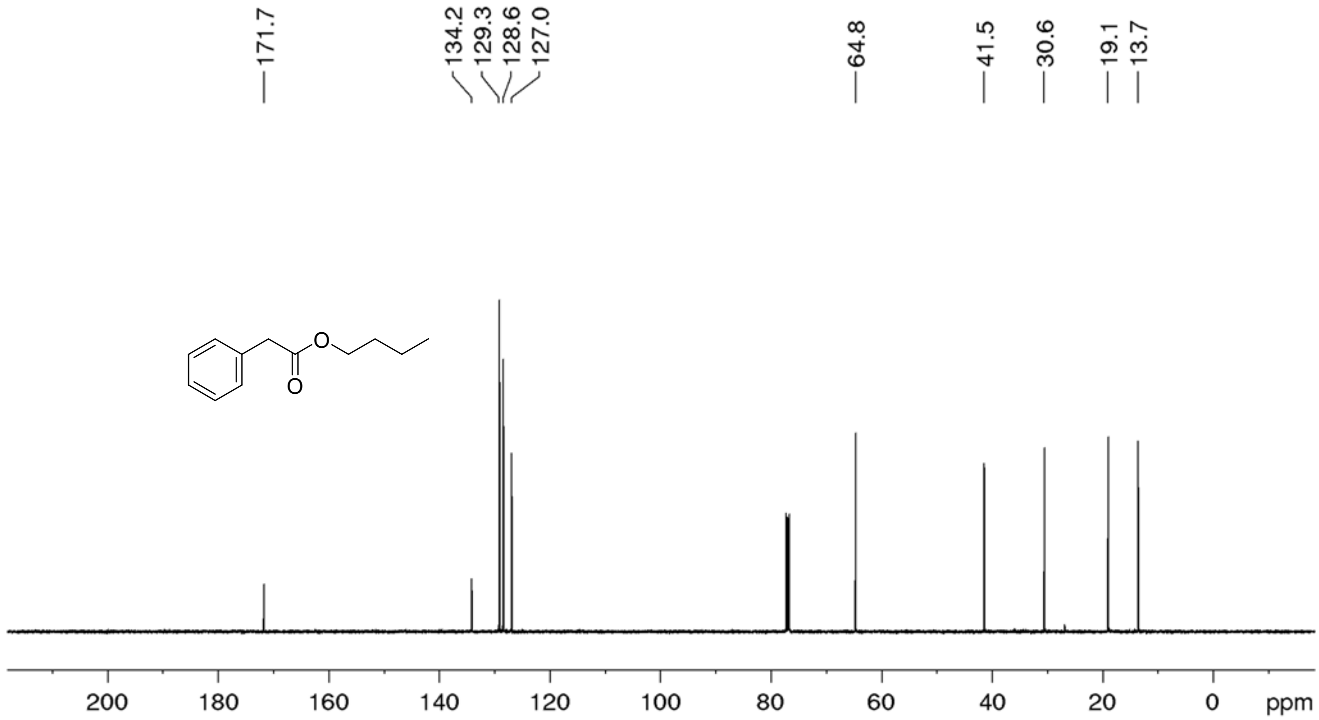

^{13}C NMR of compound **51**

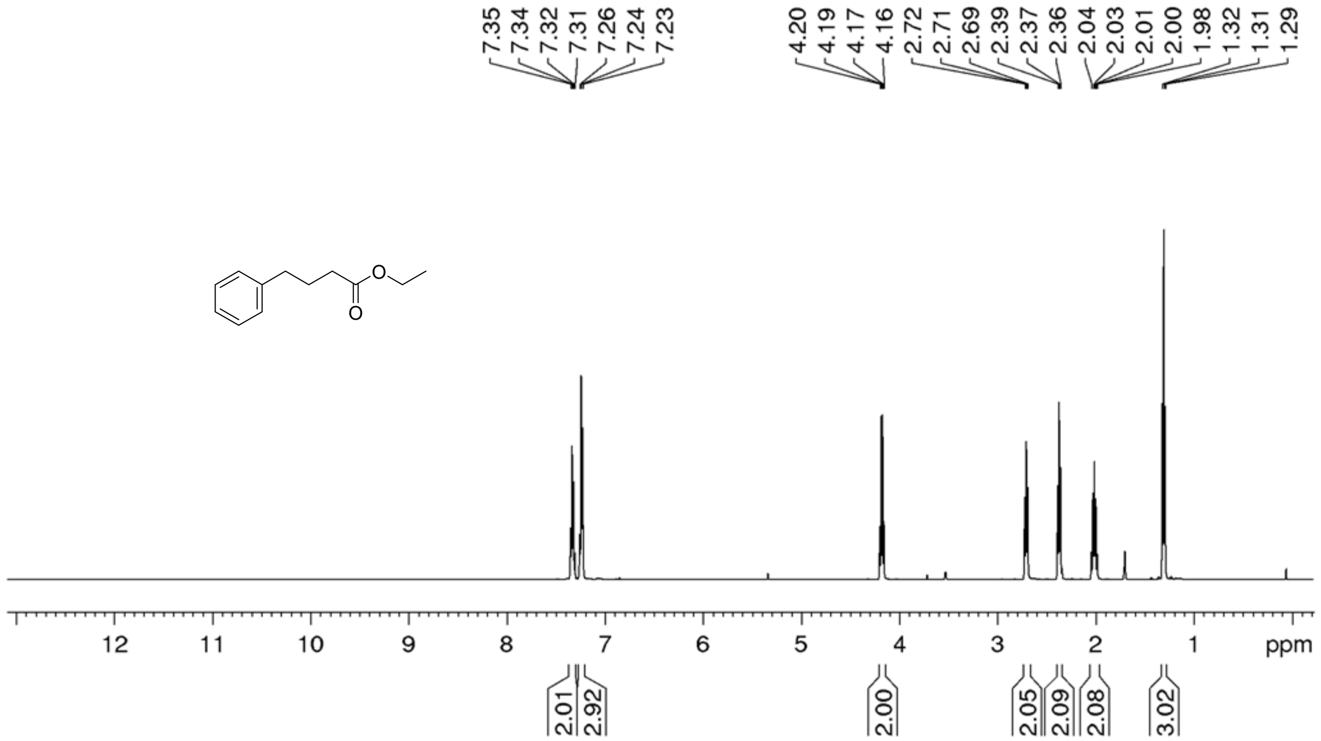

^{1}H NMR of compound **52**

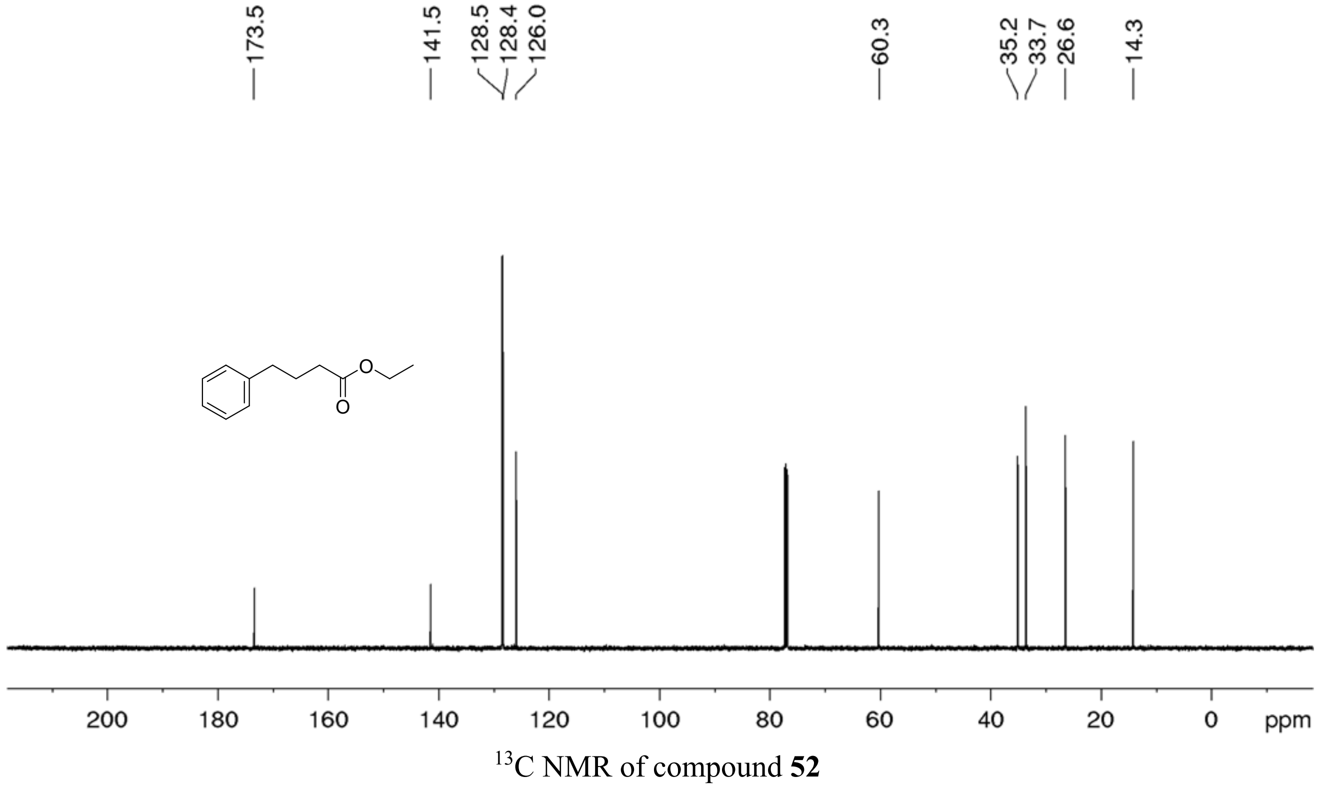

^{13}C NMR of compound **52**

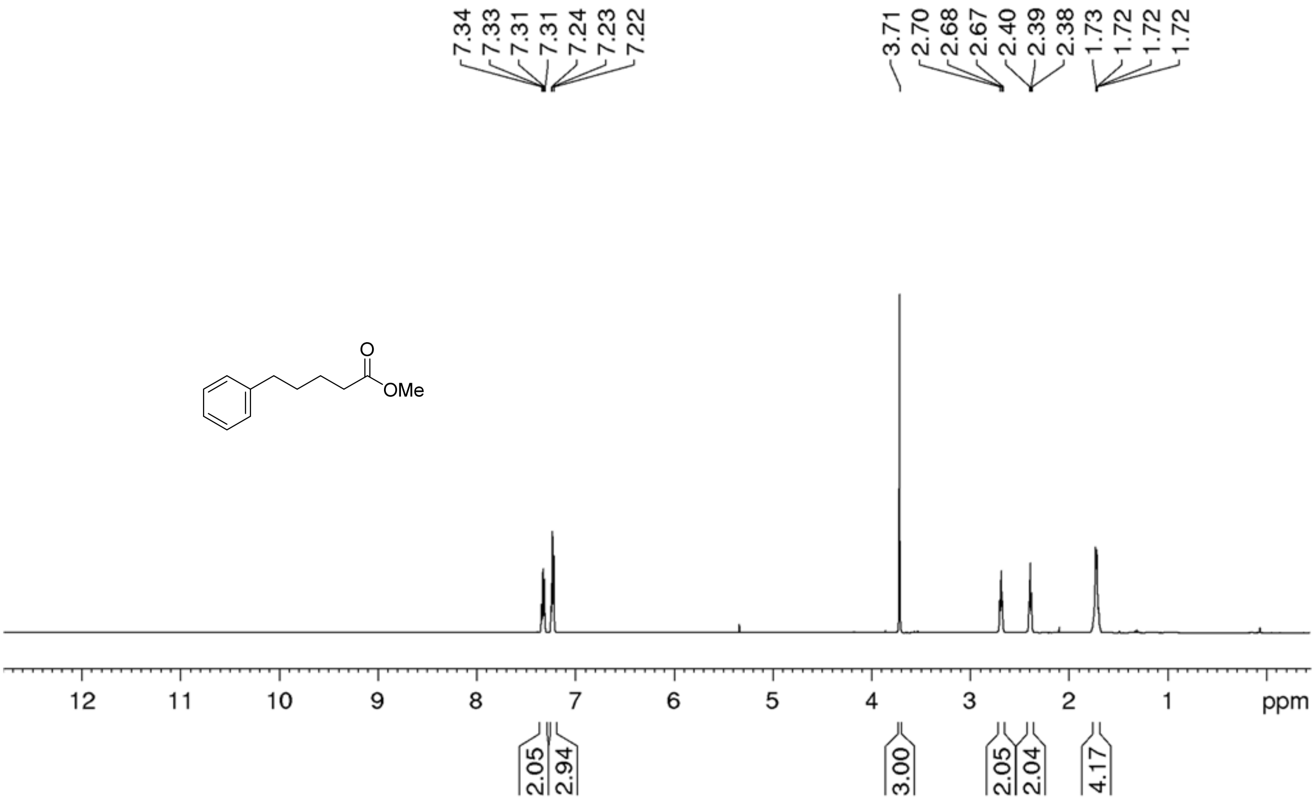

^{1}H NMR of compound **53**

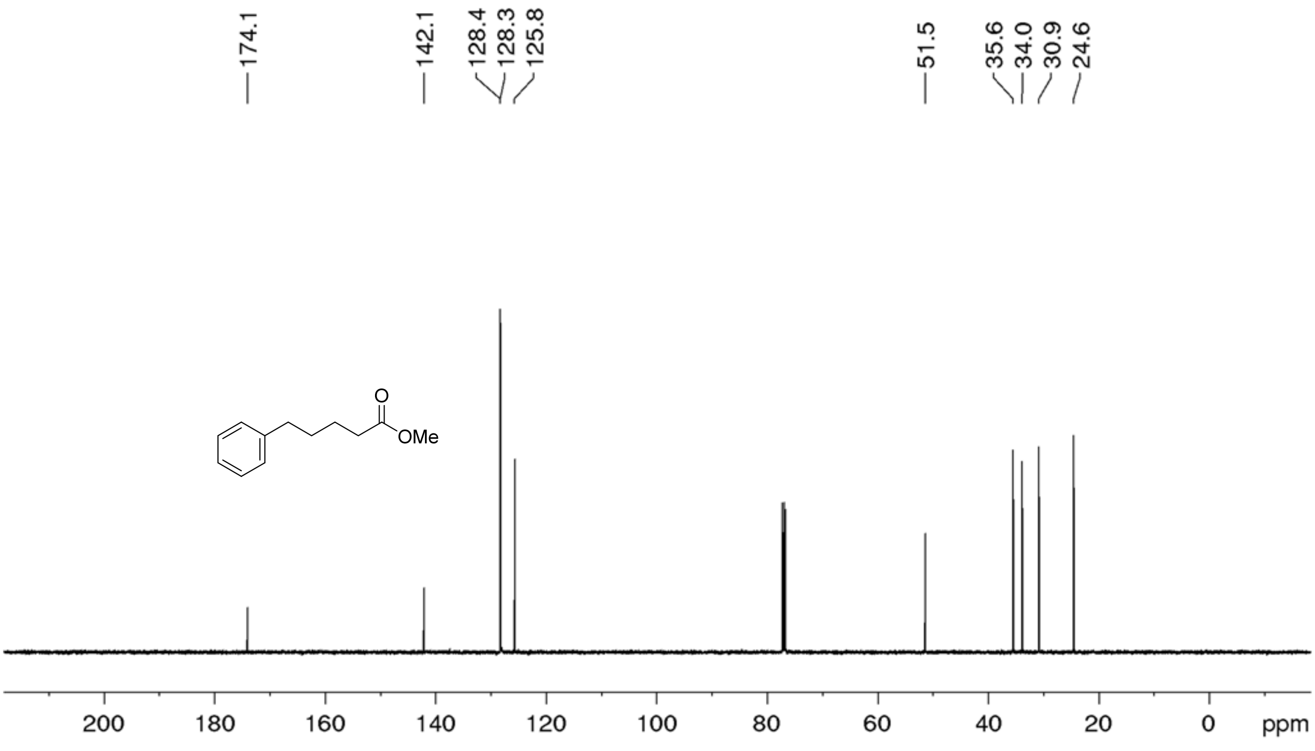

^{13}C NMR of compound **53**

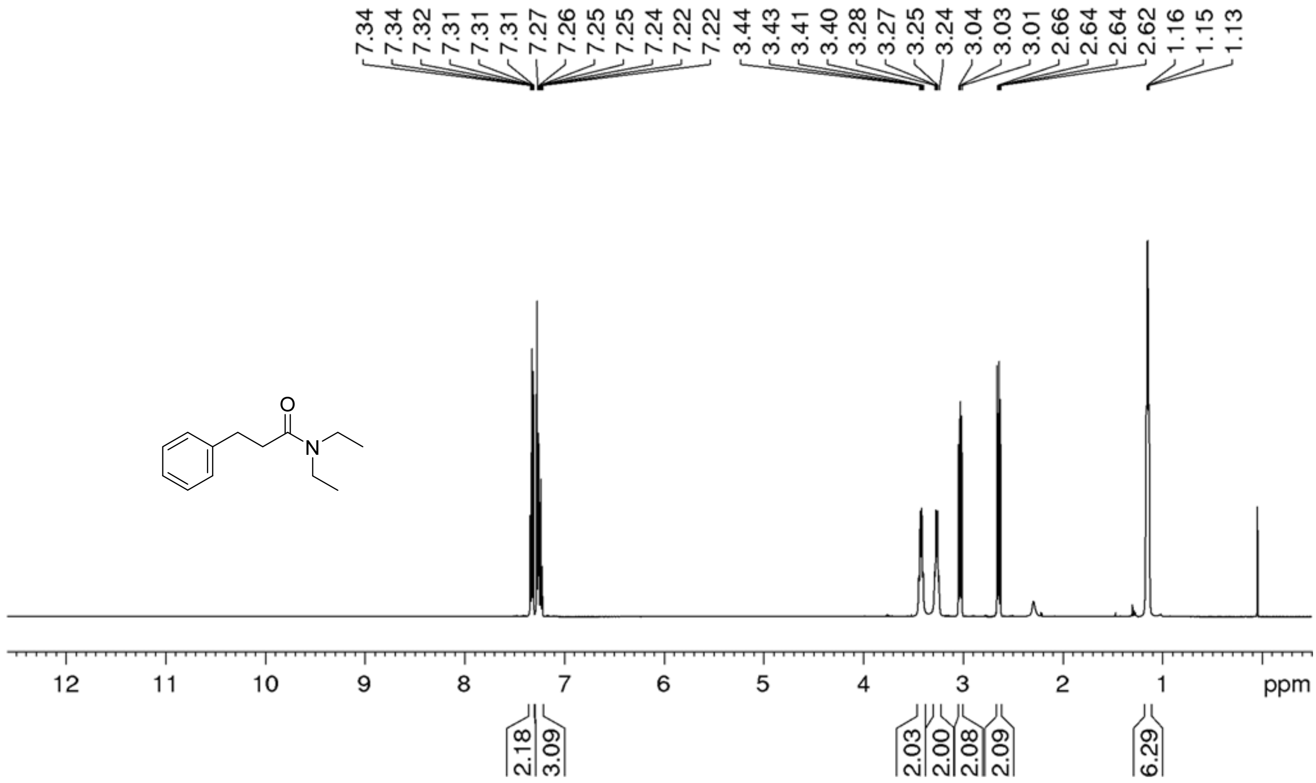

^{1}H NMR of compound **55**

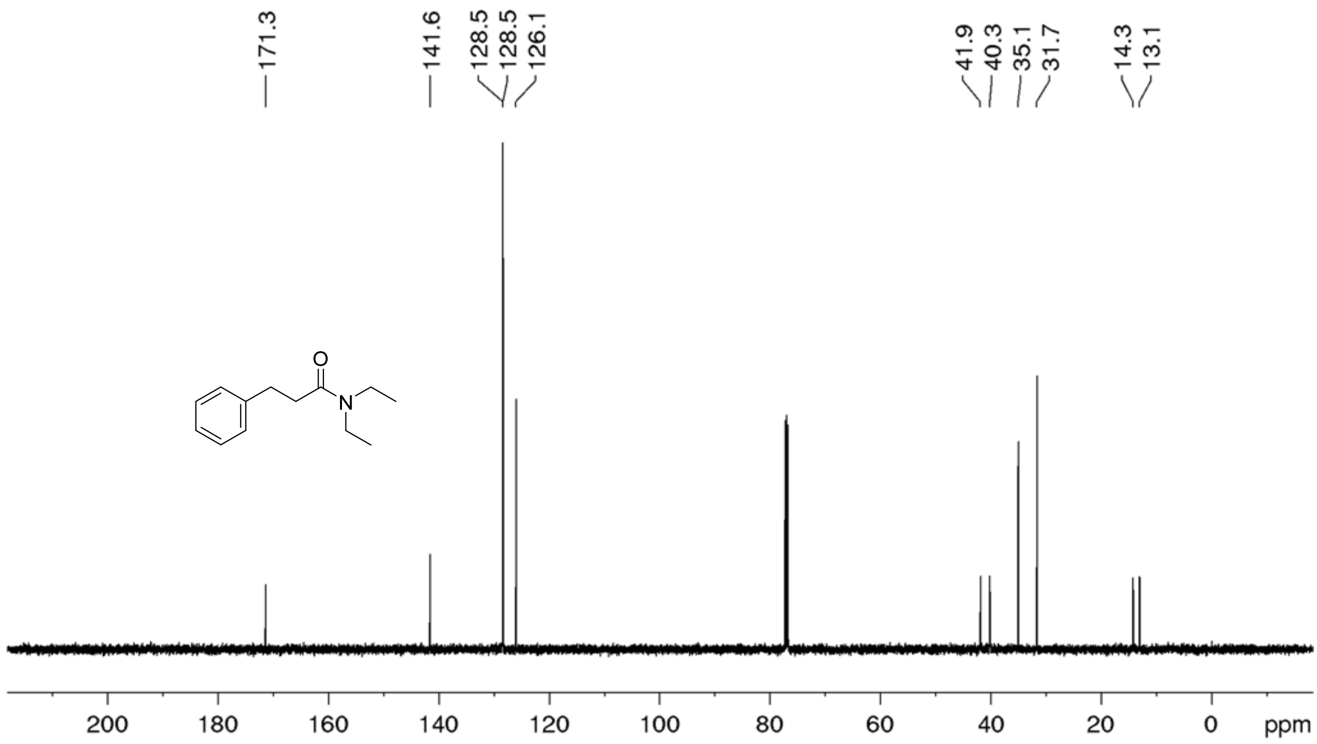

^{13}C NMR of compound **55**

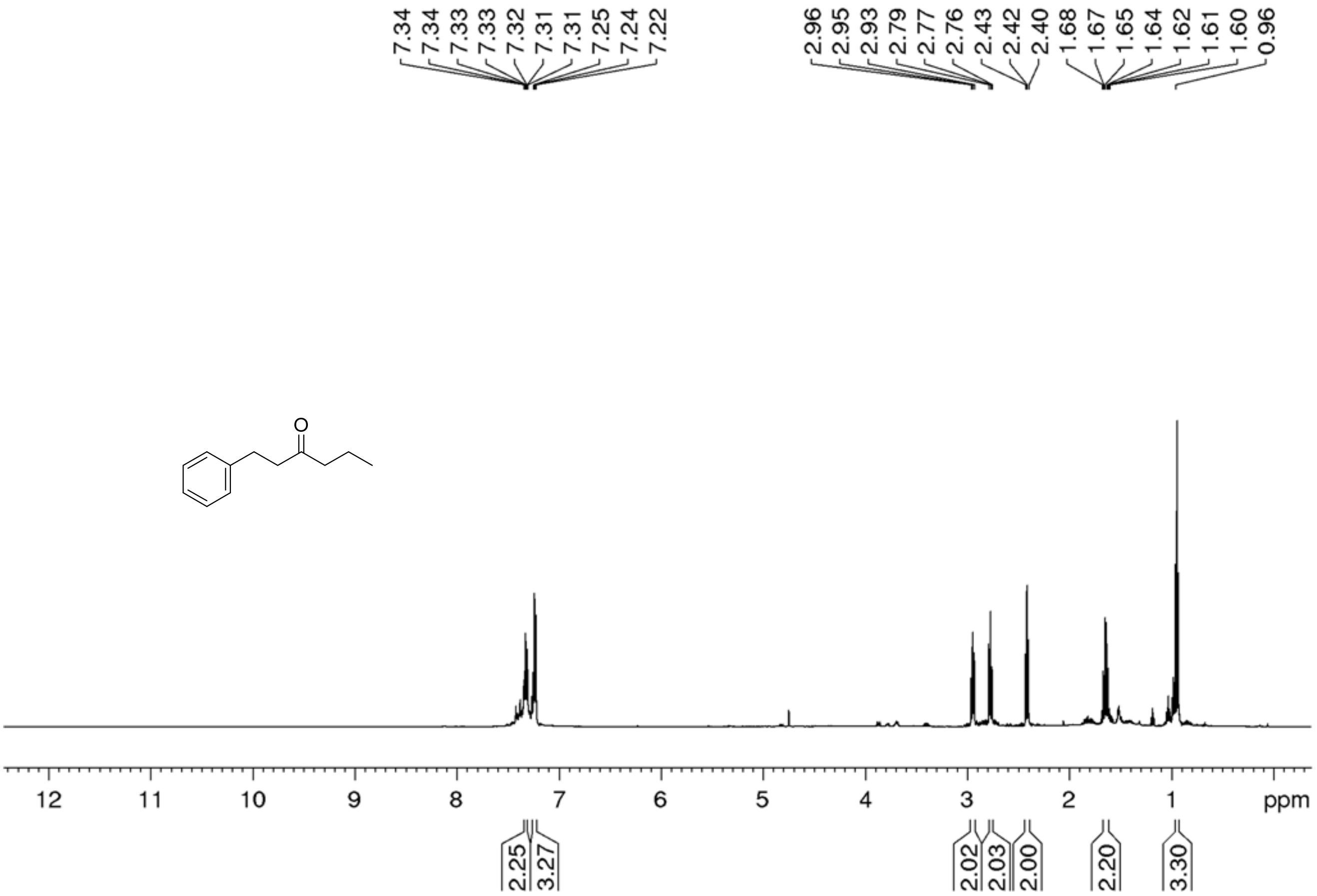

^{1}H NMR of compound **56**

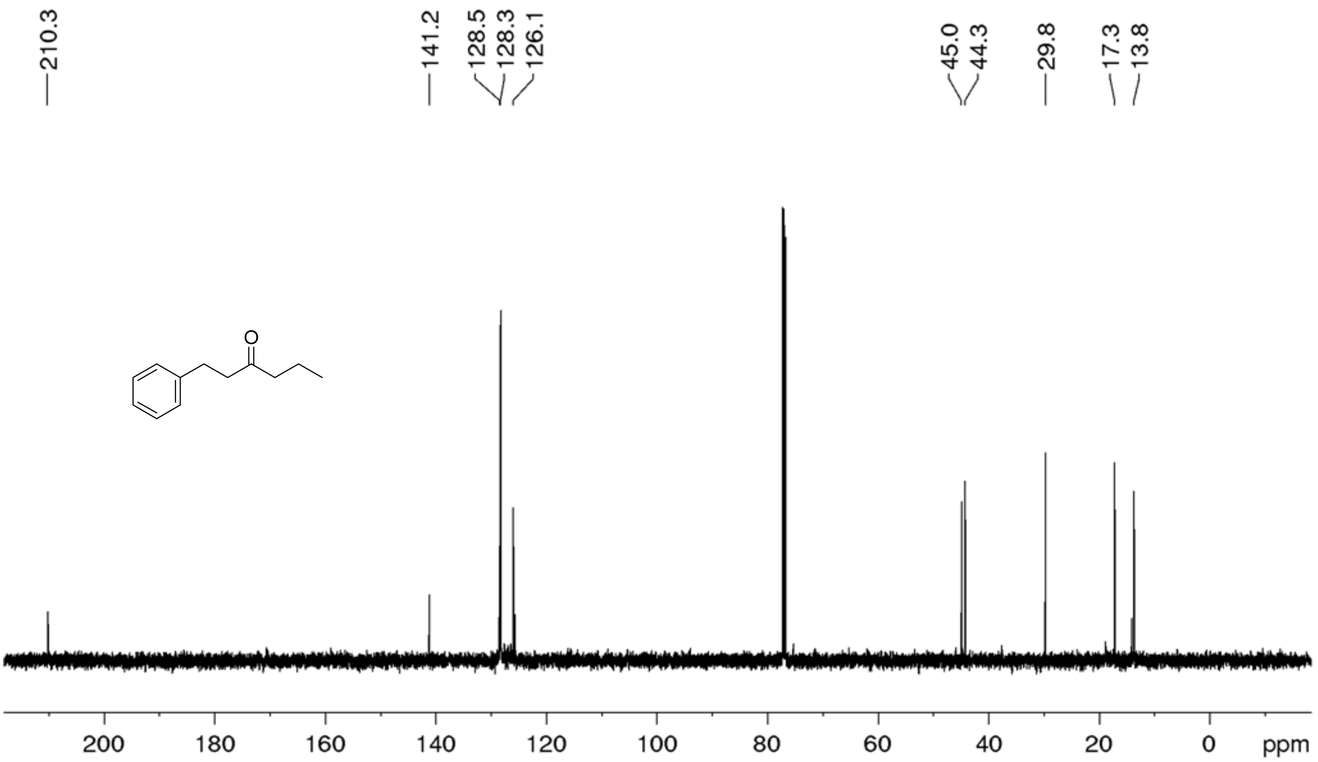

^{13}C NMR of compound **56**

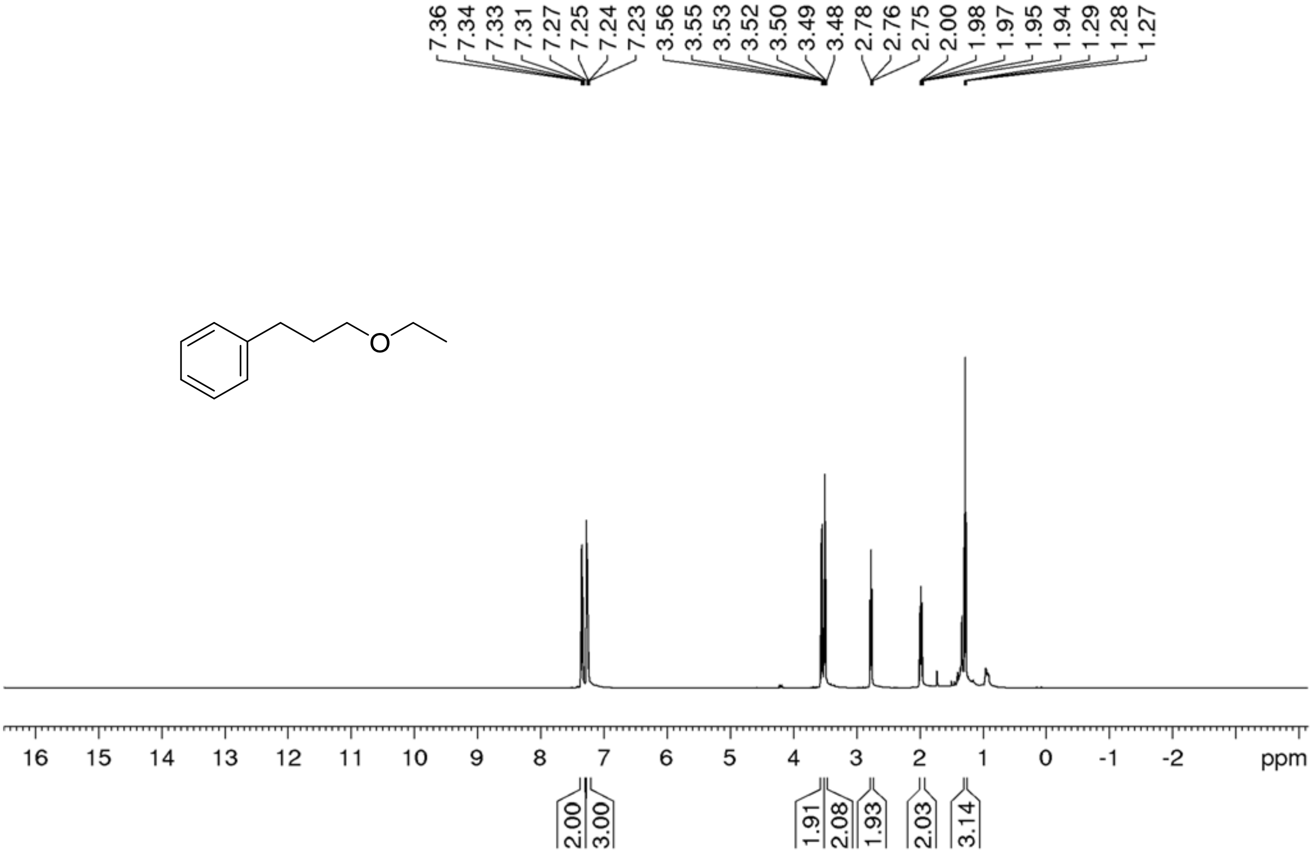

^{1}H NMR of compound **57**

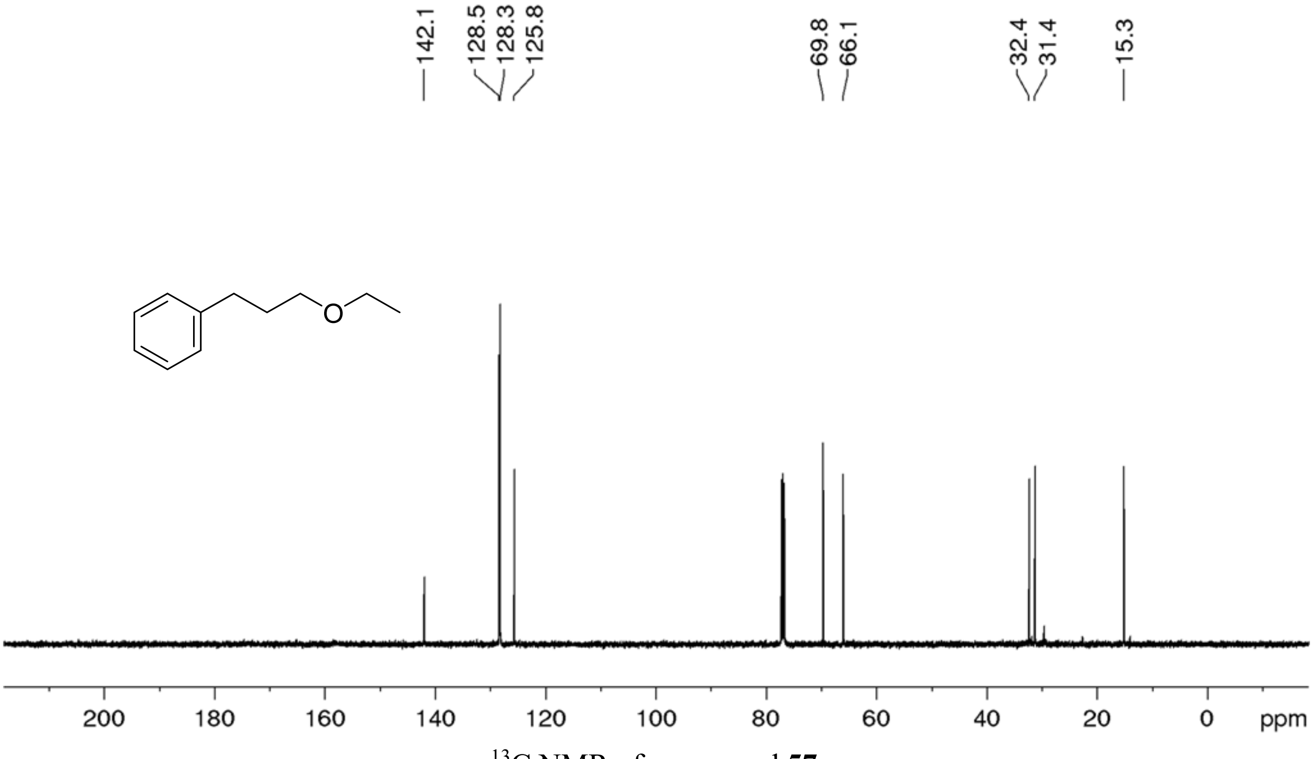

^{13}C NMR of compound **57**

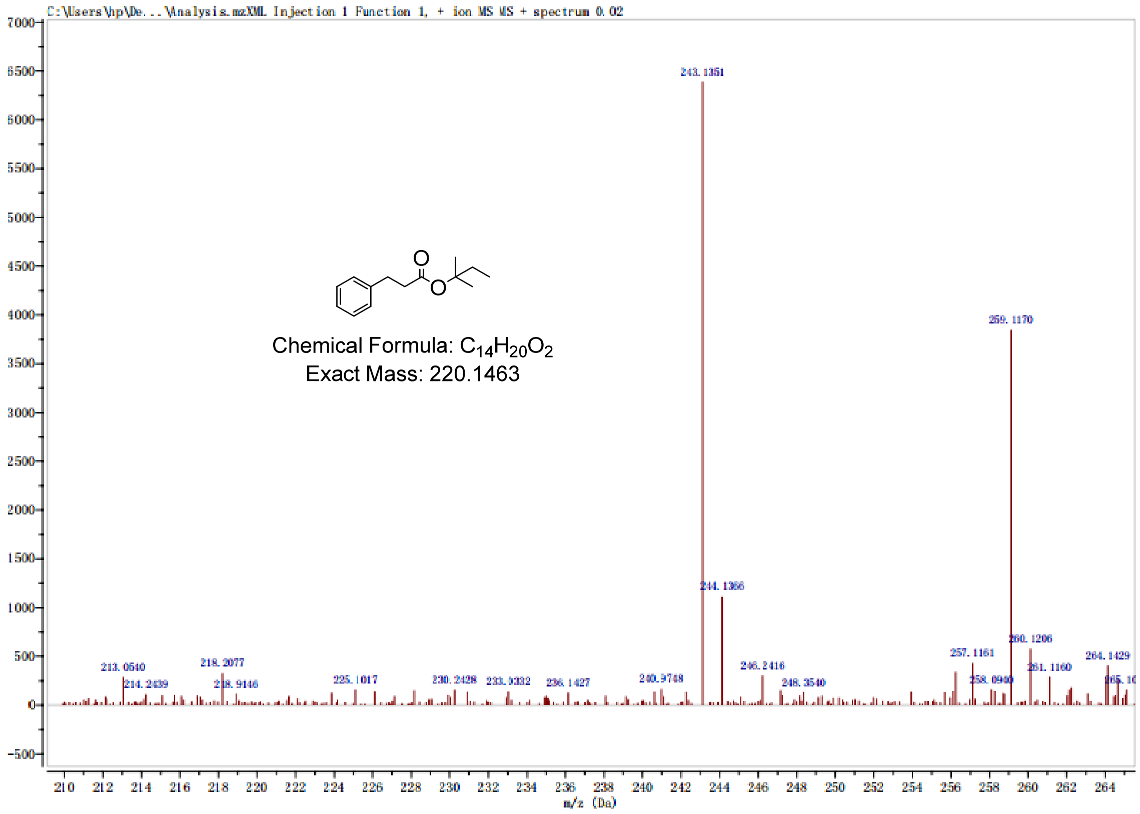

HR-ESI-MS of compound **13**

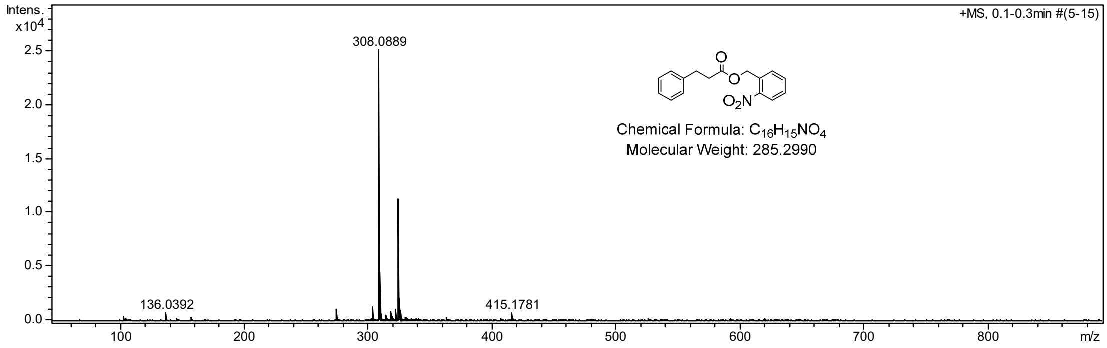

HR-ESI-MS of compound **29**

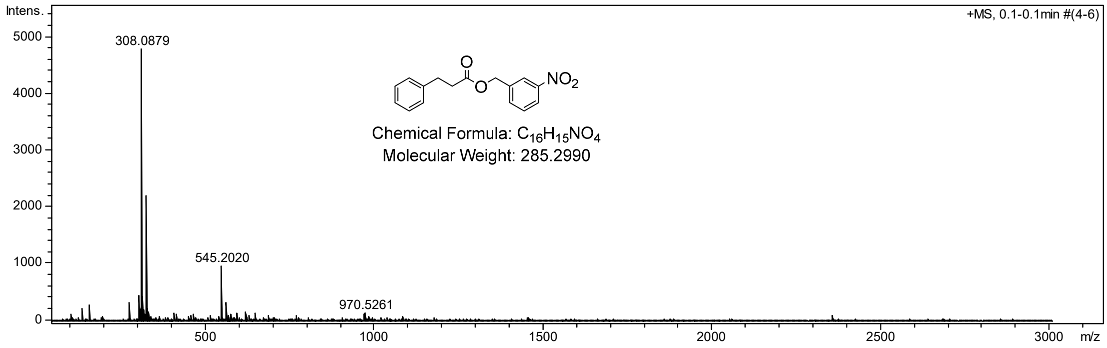

HR-ESI-MS of compound **30**

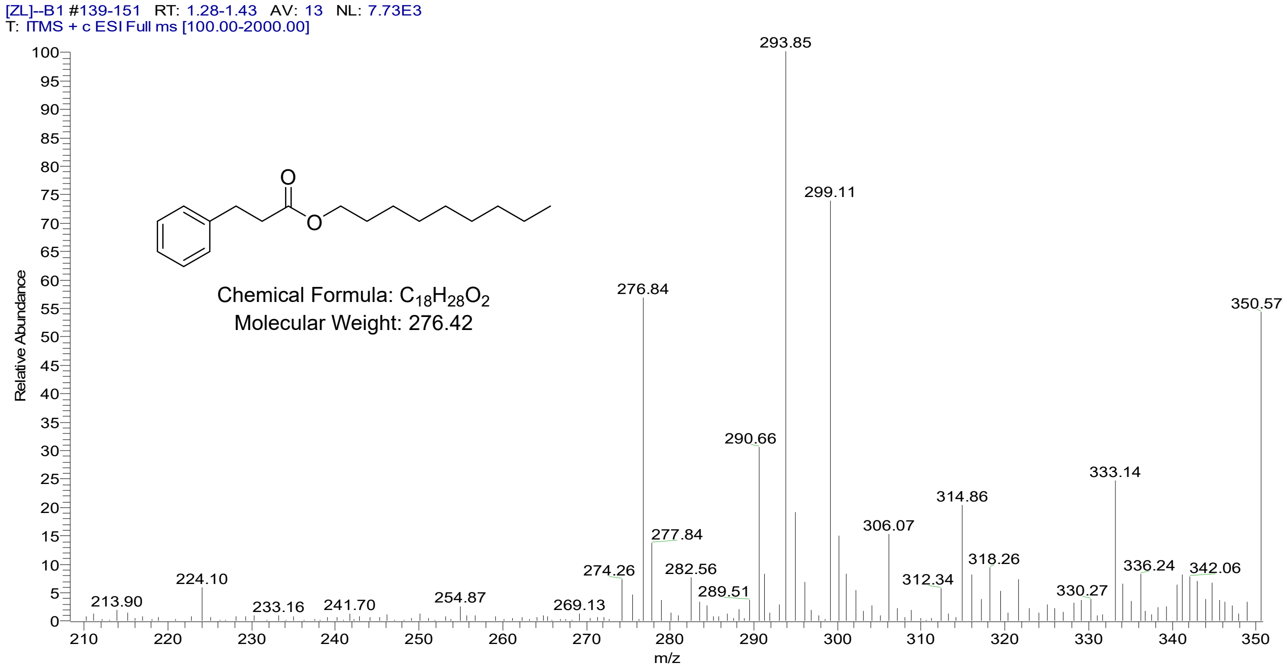

Positive ESI-MS of Compound **9**

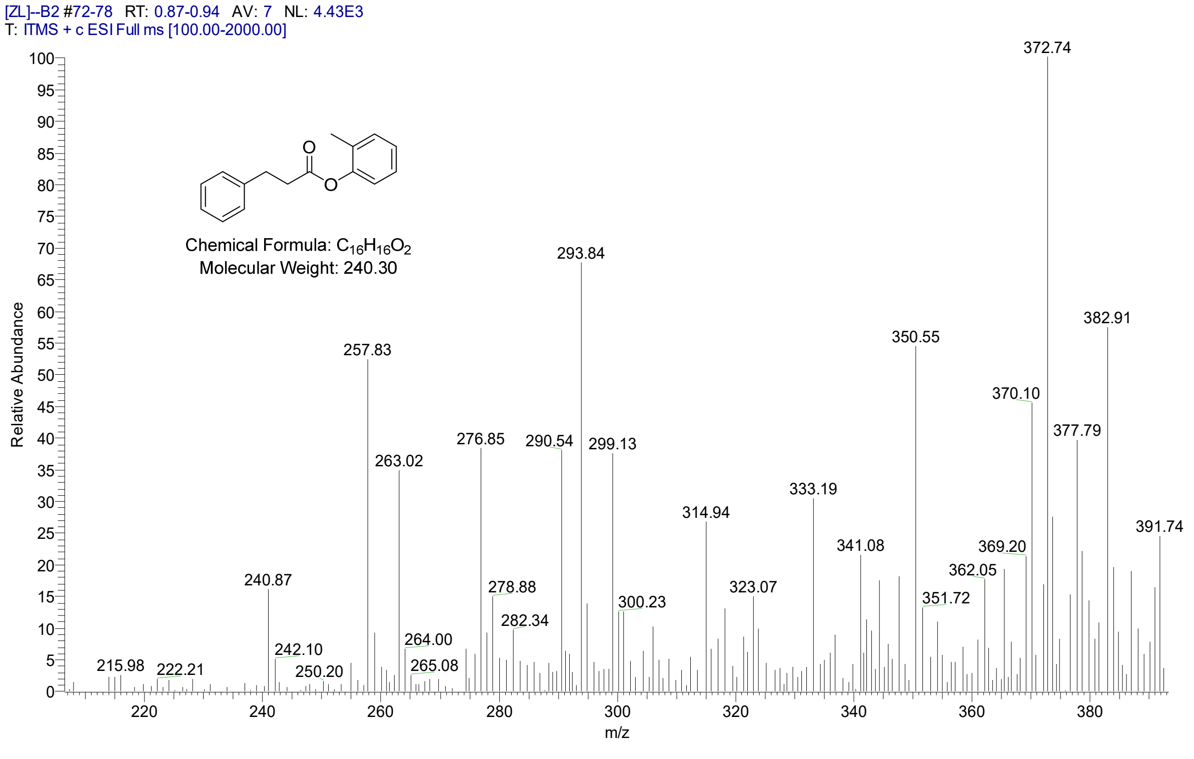

Positive ESI-MS of Compound **16**

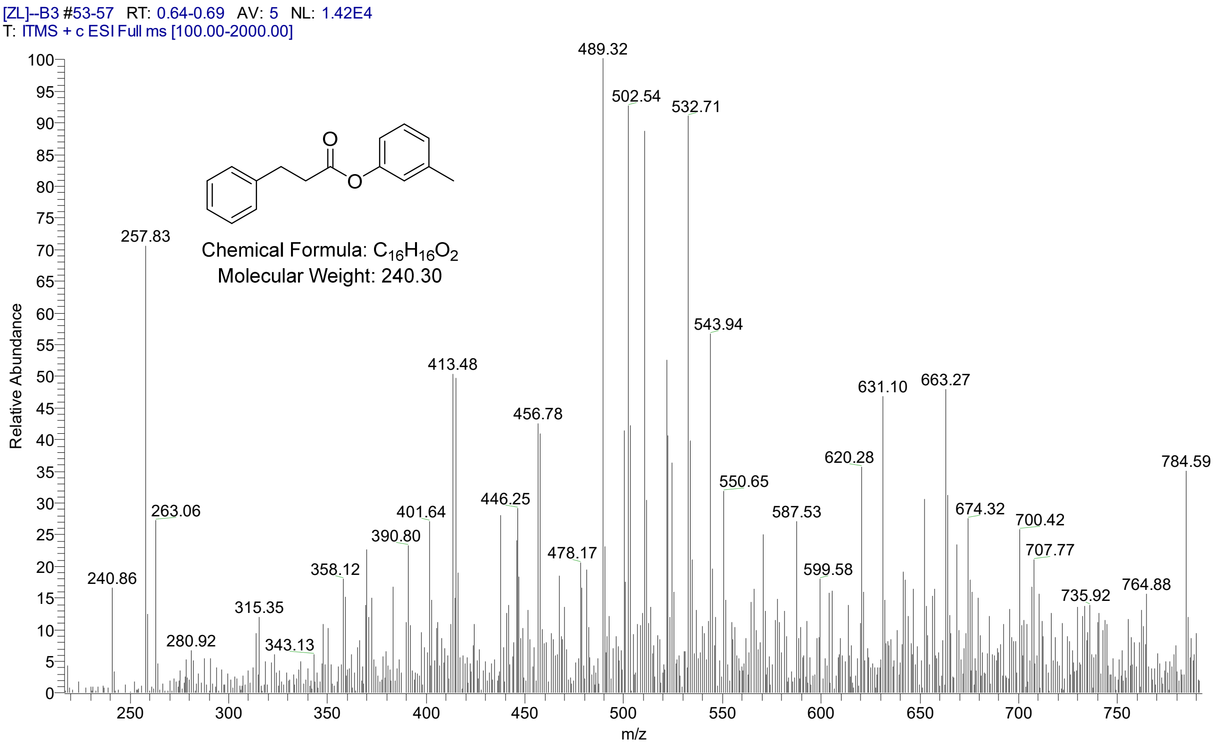

Positive ESI-MS of Compound **17**

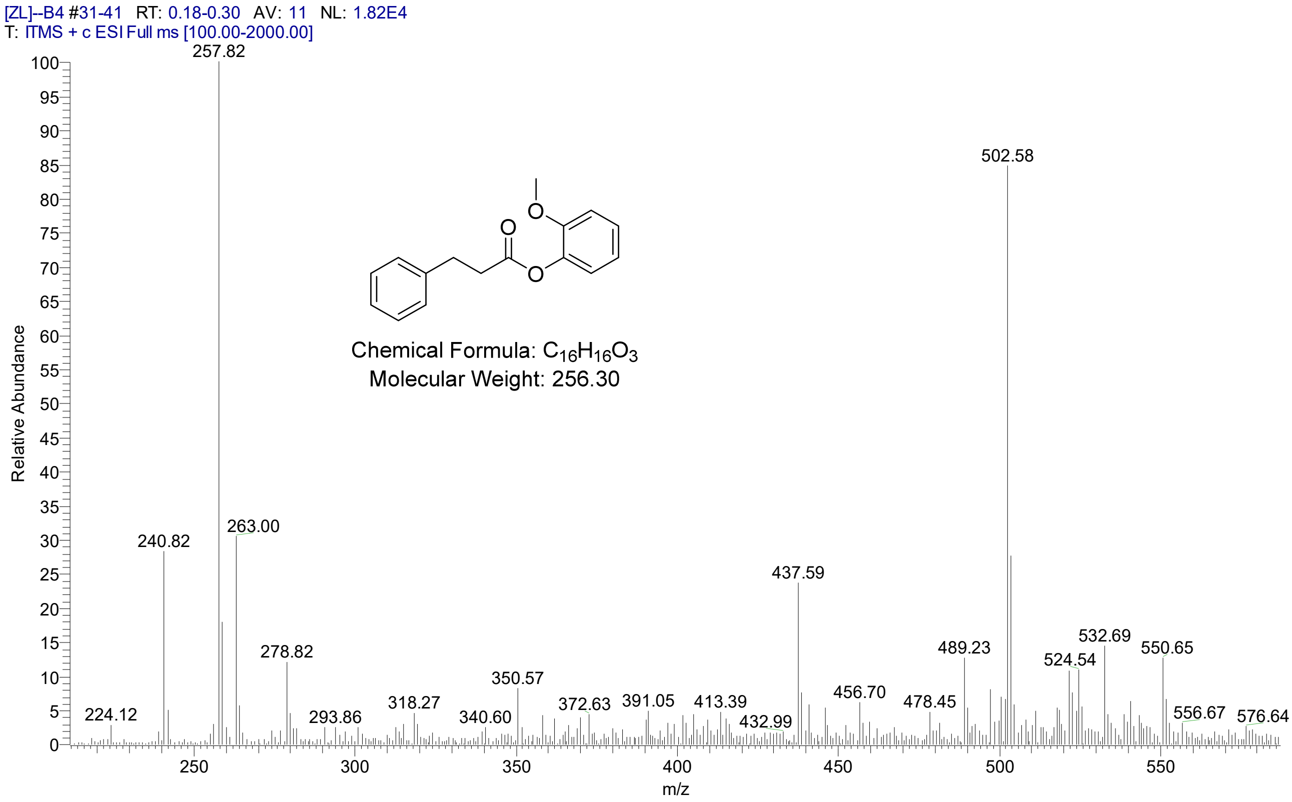

Positive ESI-MS of Compound **19**

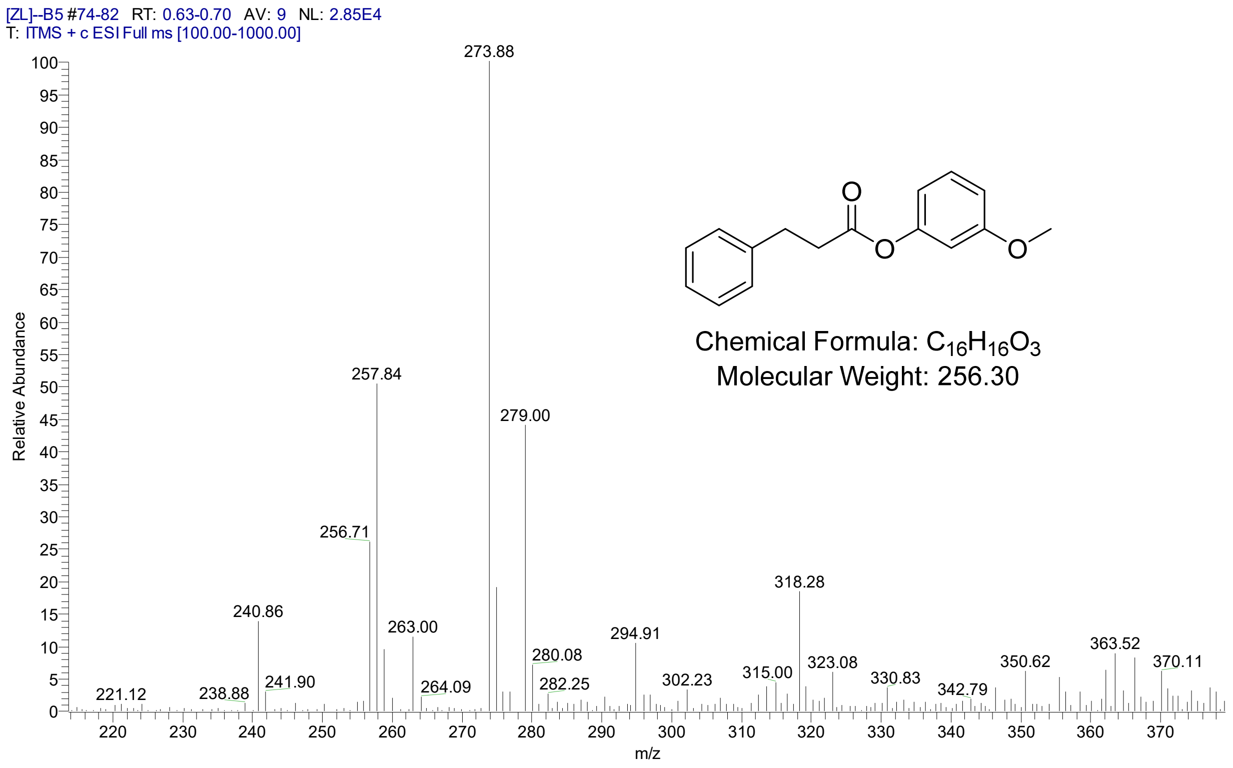

Positive ESI-MS of Compound **20**

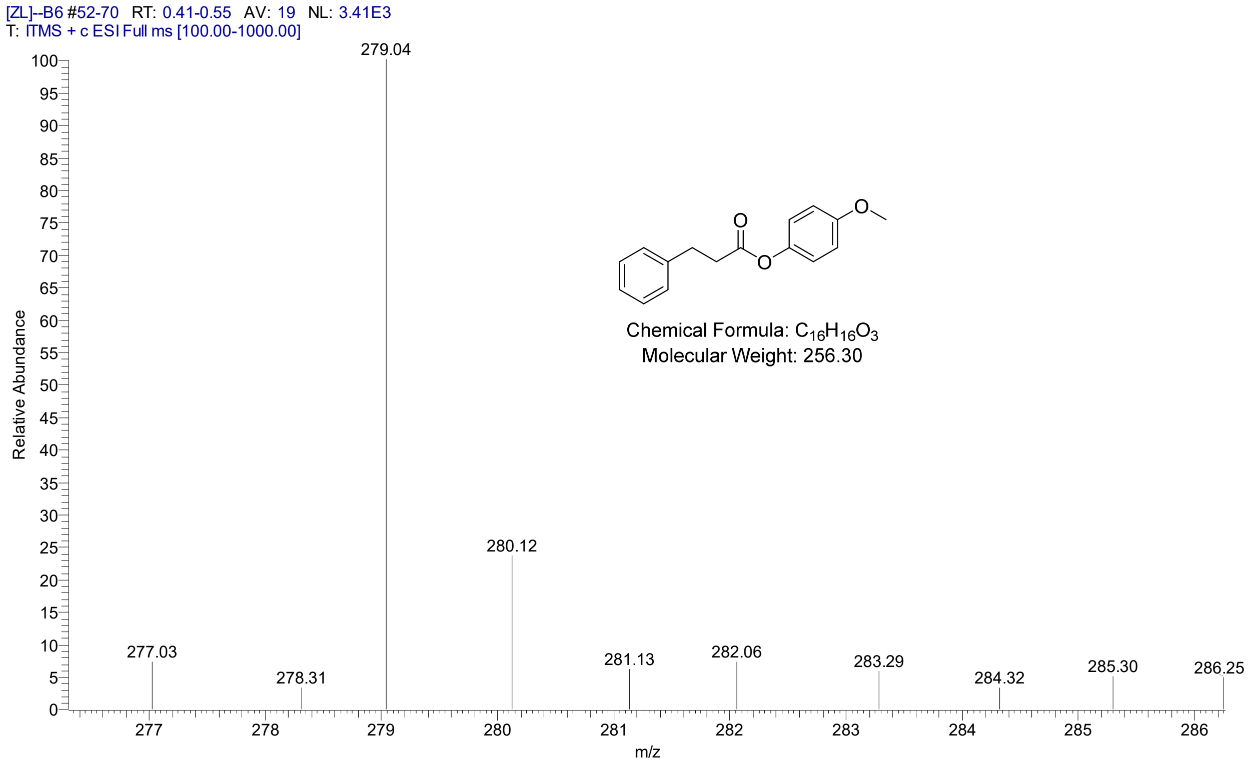

Positive ESI-MS of Compound **21**

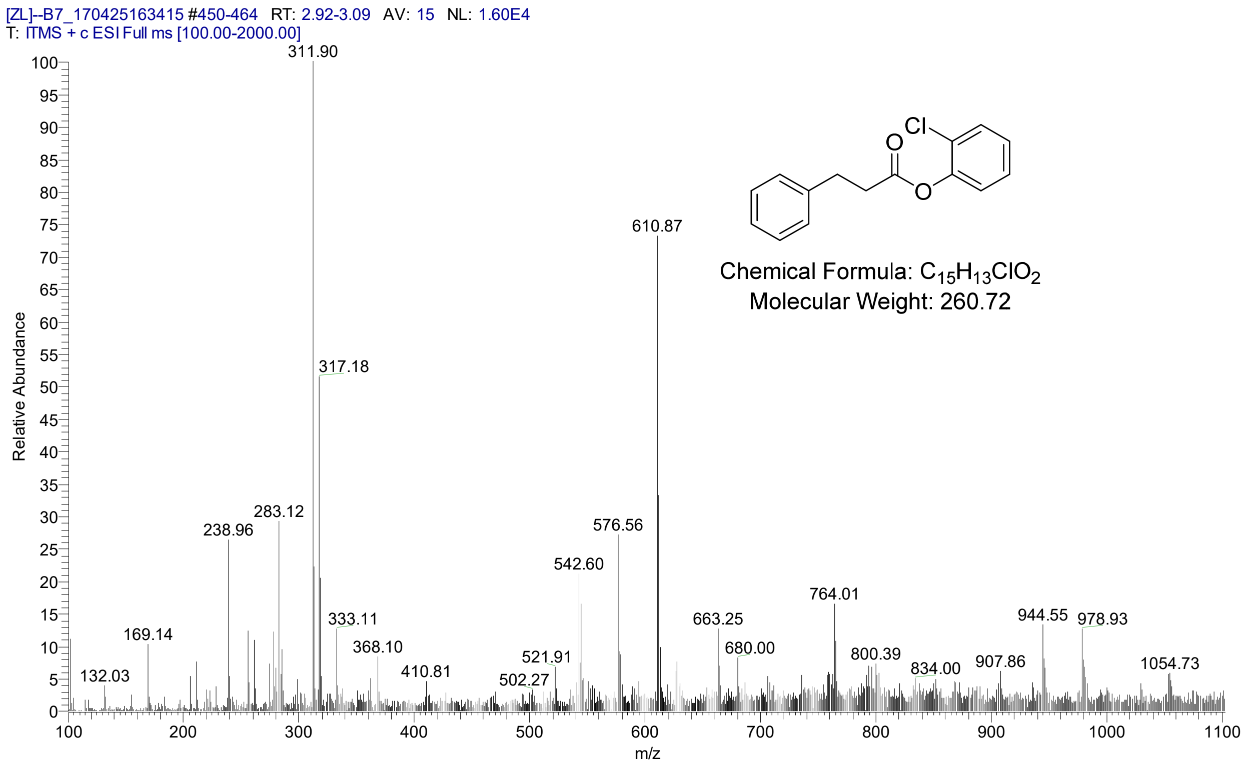

Positive ESI-MS of Compound **22**

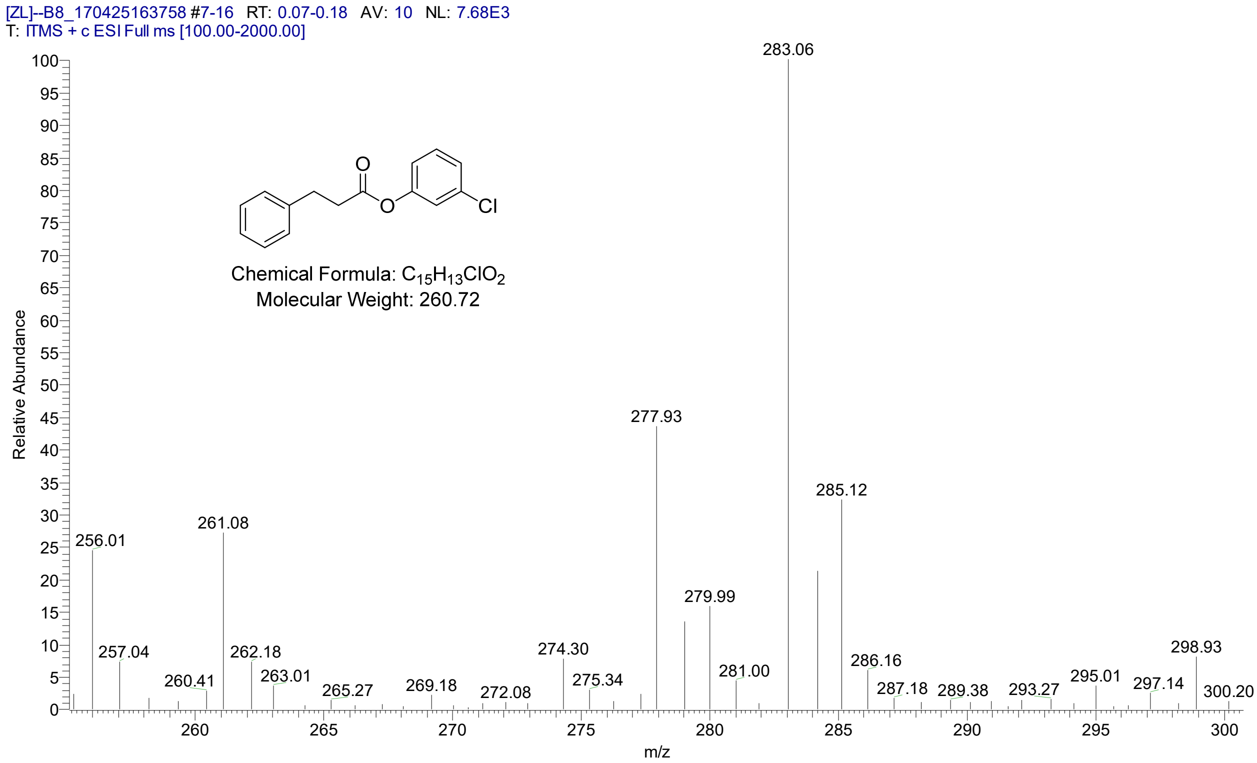

Positive ESI-MS of Compound **23**

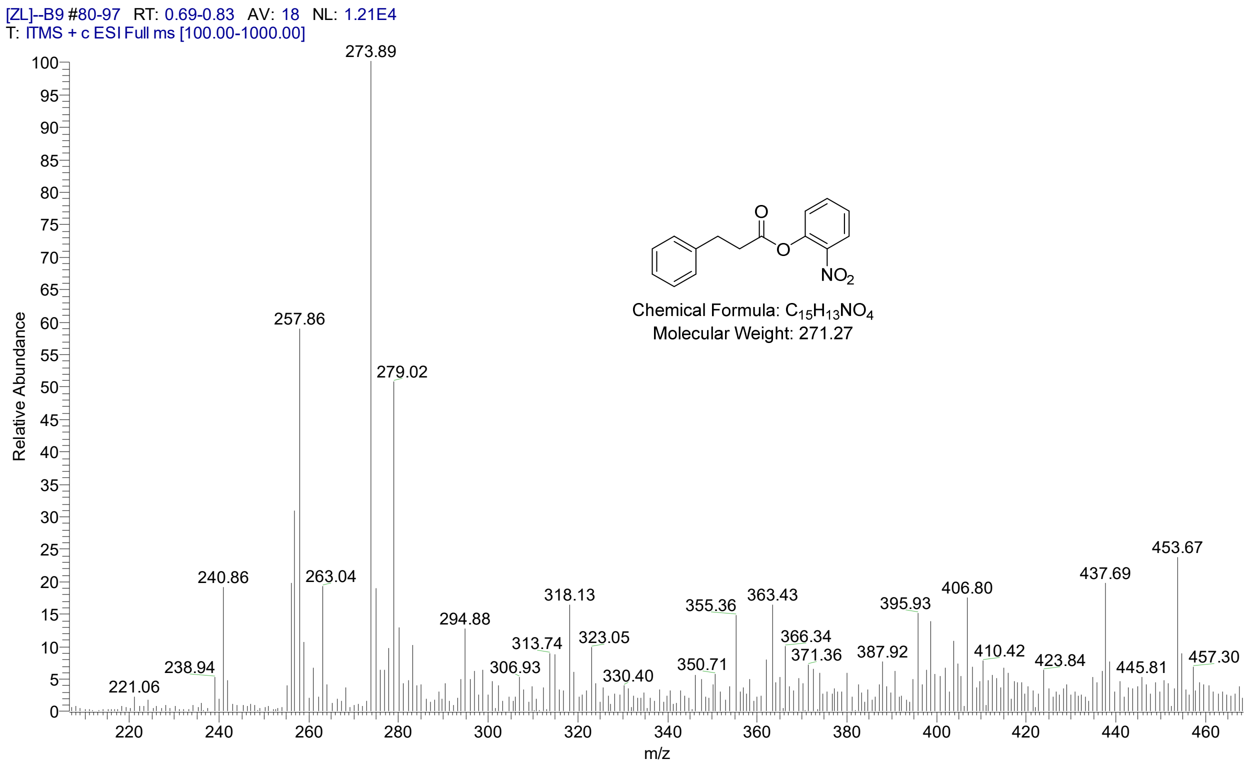

Positive ESI-MS of Compound **25**

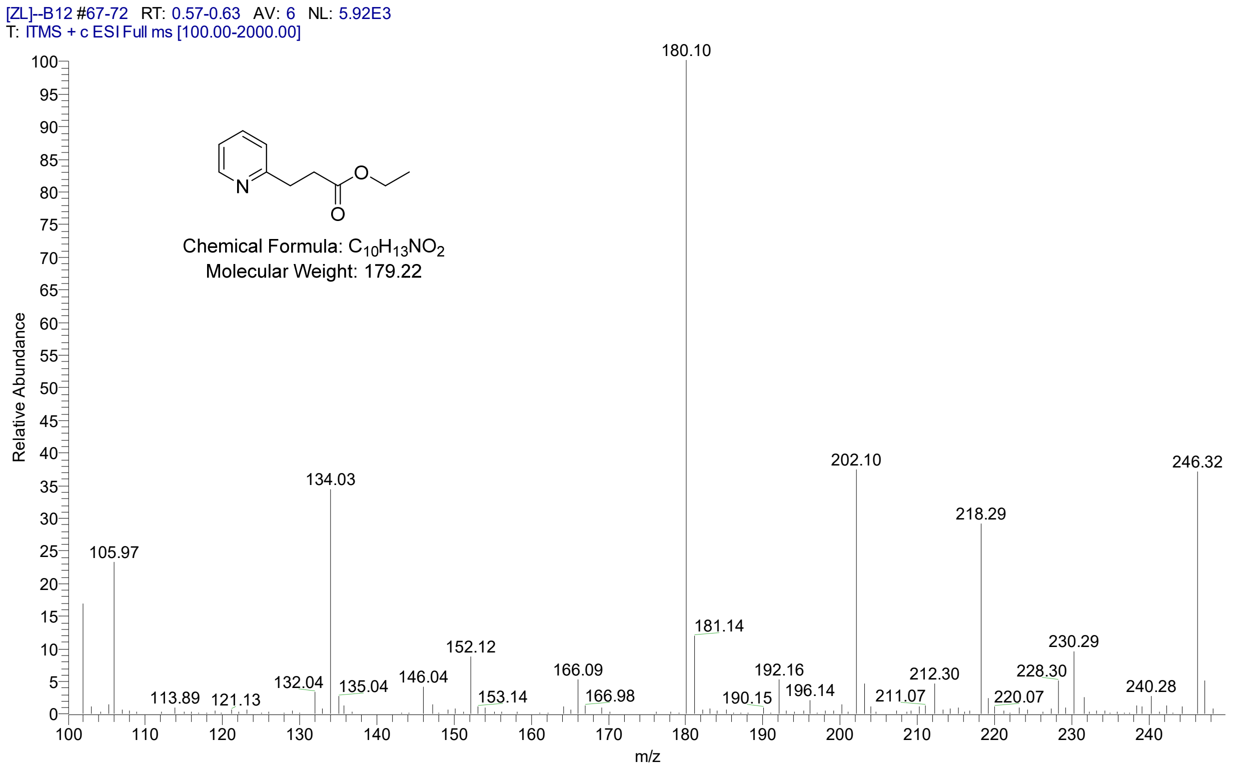

Positive ESI-MS of Compound **43**

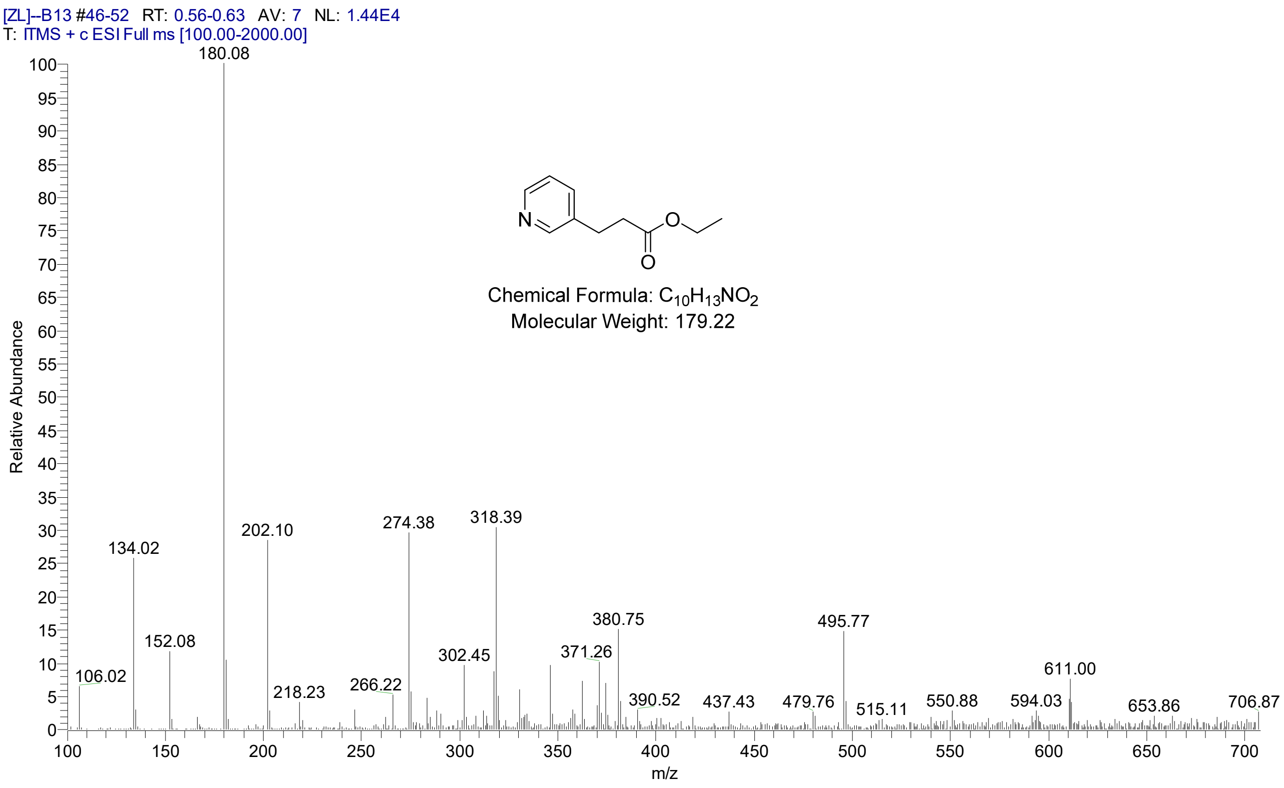

Positive ESI-MS of Compound **44**

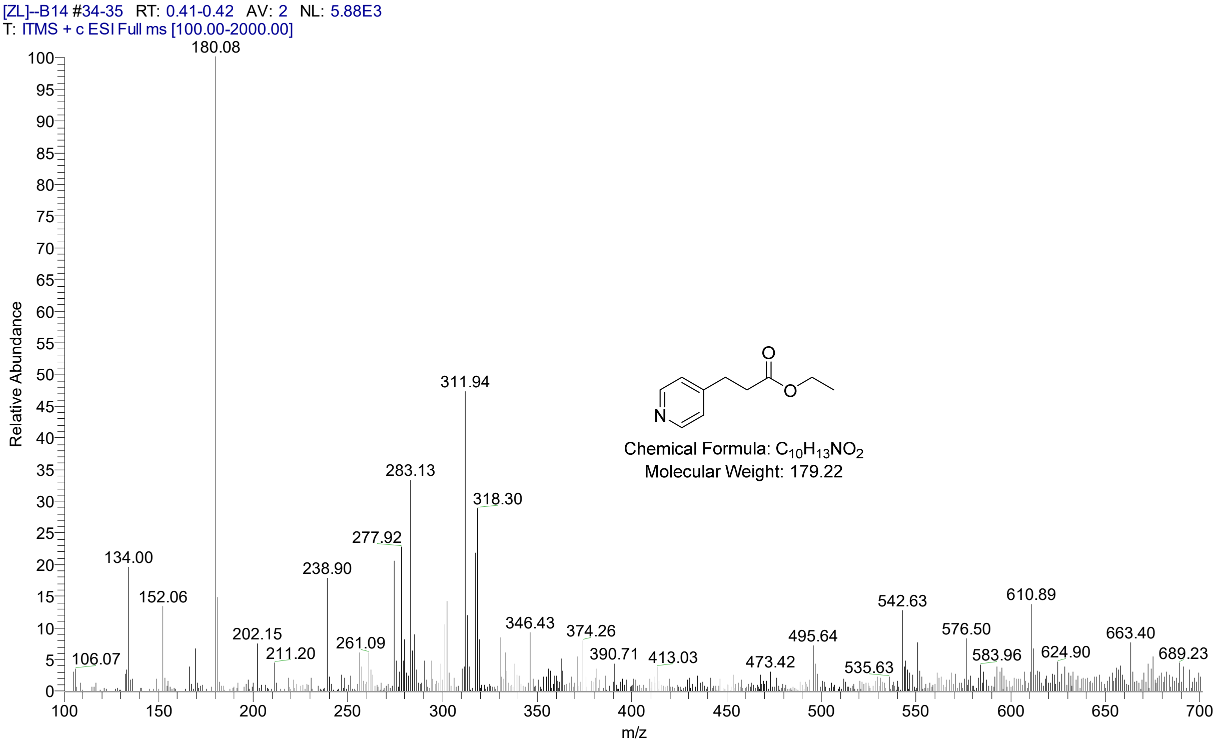

Positive ESI-MS of Compound **45**

References

1. Guo, L.; Ma, X.; Fang, H.; Jia, X.; Huang, Z. A general and mild catalytic α-alkylation of unactivated esters using alcohols. *Angew. Chem. Int. Edit.*, **54**, 4023-4027 (2015). doi: 10.1002/anie.201410293 PMID: 25651099
2. Monguchi, Y.; Marumoto, T.; Ichikawa, T.; Miyake, Y.; Nagae, Y.; Yoshida, M.; Oumi, Y.; Sawama, Y.; Sajiki, H. Unique Chemoselective hydrogenation using a palladium catalyst immobilized on ceramic. *Chemcatchem,* **7**, 2155–2160 (2015). doi: 10.1002/cctc.201500193
3. Liu, J., Shao, C., Zhang, Y., Shi, G., Pan, S. Copper-catalyzed highly efficient ester formation from carboxylic acids/esters and formats. *Org. Biomol. Chem.*, **12**, 2637-2640 (2014). doi: 10.1039/c4ob00193a PMID: 24668215
4. Liu, X.; Hu, W. 4-(Nitro)-diphenylammonium triflate(NDPAT) catalyzed esterification of carboxylic acids with alcohols. *J. Chem. Res.*, **8**, 564-565 (2004).
5. Xu, X.-H.; Azuma, A.; Taniguchi, M.; Tokunaga, E.; Shibata, N. Efficient direct ester condensation between equimolar amounts of carboxylic acids and alcohols catalyzed by trifluoromethanesulfonic acid (TfOH) in Solkane365mfc. *RSC Adv.*, **3**, 3848-3852 (2013). doi: 10.1039/c3ra00132f
6. Emmanuel, T.; Aristide, B.; Leopold, T. N.; Benoit, N. M.; Duplex, W. J.; Gbaveng, Y.; Bernard, D.; Alain, M.; Joseph, M. T.; Roch, A. Antimicrobial activity of phenylpropanoyle from the rhizome of *Zingiber officinale Roscoe* (Zingiberaceae). *Eur. J. Med. Plant.*, **4**, 126-134 (2014). doi: 10.9734/EJMP/2014/6884
7. Deguchi, T., Xin, H.-L.; Morimoto, H.; Ohshima, T. Direct catalytic alcoholysis of unactivated 8-aminoquinoline amides. *ACS Catal.*, **7**, 3157-3161 (2017). doi: 10.1021/acscatal.7b00442
8. Miyazaki, T.; Kasai, S.; Ogiwara, Y.; Sakai, N. Indium-catalyzed reductive sulfidation of esters by using thiols: an approach to the diverse synthesis of sulfides. *Eur. J. Org. Chem.*, **2016**, 1043-1049 (2016). doi: 10.1002/ejoc.201501559
9. Miyashita, M.; Shiina, I.; Miyoshi, S.; Mukaiyama, T. A new and efficient esterification reaction via mixed anhydrides by the promotion of a catalytic amount of Lewis acid. *Bull. Chem. Soc. Jpn.*, **66**, 1516-1527 (1993). doi: 10.1246/bcsj.66.1516
10. Liu, H. X.; Dang, Y. Q.; Yuan, Y. F.; Xu, Z. F.; Qiu, S. X.; Tan, H. B. Diacyl disulfide: a reagent for chemoselective acylation of phenols enabled by 4-(N,N-Dimethylamino)pyridine catalysis. *Org. Lett.,* **18**, 5584–5587 (2016). doi: 10.1021/acs.orglett.6b02818 PMID: 27767318
11. Qian, L.; Liu, J. Y.; Liu, J. Y.; Yu, H. L.; Li, C. X.; Xu, J. H. Fingerprint lipolytic enzymes with chromogenic p-nitrophenyl esters of structurally diverse carboxylic acids. *J. Mol. Cat. B-Enzym.,* **73**, 22–26 (2011). doi: 10.1016/j.molcatb.2011.07.010
12. Yamada, K.; Karuo, Y.; Tsukada, Y.; Kunishima, M. Mild amide-cleavage reaction mediated by electrophilic benzylation. *Chem.-Eur. J.*, **22**, 14042-14047 (2016). doi: 10.1002/chem.201603120 PMID: 27529837
13. Feroci, M.; Chiarotto, I.; Orsini, M.; Pelagalli, R.; Inesi, A. Umpolung reactions in an ionic liquid catalyzed by electrogenerated N-heterocyclic carbenes. Synthesis of saturated esters from activated α,β-unsaturated aldehydes. *Chem. Comm.* (Cambridge, United Kingdom), **48**, 5361-5363 (2012). doi: 10.1039/c2cc30371j PMID: 22517053
14. Takakura, H.; Kojima, R.; Kamiya, M.; Kobayashi, E.; Komatsu, T.; Ueno, T.; Terai, T.; Hanaoka, K.; Nagano, T.; Urano, Y. New Class of Bioluminogenic Probe Based on Bioluminescent Enzyme-Induced Electron Transfer: BioLeT. *J. Amer. Chem. Soc.,* **137**, 4010–4013 (2015). doi: 10.1021/ja511014w PMID: 25761130
15. Botta, G.; Bizzarri, B. M.; Garozzo, A.; Timpanaro, R.; Bisignano, B.; Amatore, D.; Palamara, A. T.; Nencioni, L.; Saladino, R. Carbon nanotubes supported tyrosinase in the synthesis of lipophilic hydroxytyrosol and dihydrocaffeoyl catechols with antiviral activity against DNA and RNA viruses. *Bioorg. Med. Chem.,* **23**, 5345–5351 (2015). doi: 10.1016/j.bmc.2015.07.061 PMID: 26260341
16. Dong, K.; Sang, R.; Liu, J.; Razzaq, R.; Franke, R.; Jackstell, R.; Beller, M. Palladium-catalyzed carbonylation of sec- and tert-alcohols. *Angew. Chem. Int. Edit.*, **56**, 6203-6207 (2017). doi: 10.1002/anie.201701950 PMID: 28429424

17. Zysk, M.; Zadlo, A.; Brodzka, A.; Wisniewska, C.; Ostaszewski, R. The unexpected kinetic effect of enzyme mixture: The case of enzymatic esterification. *J. Mol. Catal. B-Enzym.,* **102**, 225–229 (2014). doi: 10.1016/j.molcatb.2014.02.020
18. Vieira, T. O.; Green, M. J.; Alper, H. Highly regioselective anti-markovnikov palladium-borate-catalyzed methoxycarbonylation reactions: unprecedented results for aryl olefins. *Org. Lett.,* **38**, 6143–6145 (2007). doi: 10.1021/ol062646n PMID: 17165950
19. Leow, D.; Chen, Y.-H.; Hung, T.-H.; Su, Y.; Lin, Y.-Z. Photodriven transfer hydrogenation of olefins. *Eur. J. Org. Chem.*, **2014**, 7347-7352 (2014). doi: 10.1002/ejoc.201403021
20. Liu, B.; Hu, L. 5'-(2-Nitrophenylalkanoyl)-2'-deoxy-5-fluorouridines as potential prodrugs of FUDR for reductive activation. *Bioorg. Med. Chem.,* **11**, 3889–3899 (2003). doi: 10.1016/S0968-0896(03)00426-7 PMID: 12927849
21. Allegretta, G.; Weidel, E.; Empting, M.; Hartmann, R. W. Catechol-based substrates of chalcone synthase as a scaffold for novel inhibitors of PqsD. *Eur. J. Med. Chem.,* **90**, 351–359 (2015). doi: 10.1016/j.ejmech.2014.11.055 PMID: 25437621
22. Tran, G.; Hesp, K. D.; Mascitti, V.; Ellman, J. A. Base-controlled completely selective linear or branched rhodium(I)-catalyzed C-H ortho-alkylation of azines without preactivation. *Angew. Chem. Int. Edit.*, **56**, 5899-5903 (2017). doi: 10.1002/anie.201702409 PMID: 28429455
23. Shen, Z.-L.; Goh, K. K. K.; Wong, C. H. A.; Yang, Y.-S.; Lai, Y.-C.; Cheong, H.-L.; Loh, T. P. Direct synthesis of ester-containing indium homoenolate and its application in palladium-catalyzed cross-coupling with aryl halide. *Chem. Comm.* (Cambridge, United Kingdom), **47**, 4778-4780 (2011). doi: 10.1039/c0cc05597b PMID: 21412540
24. Amatore, M.; Gosmini, C.; Perichon, J. CoBr2(Bpy): An efficient catalyst for the direct conjugate addition of aryl halides or triflates onto activated olefins. *J. Org. Chem.*, **71**, 6130-6134 (2006). doi: 10.1021/jo060855f PMID: 16872196
25. Panda, S.; Coffin, A.; Nguyen, Q. N.; Tantillo, D. J.; Ready, J. M. Synthesis and utility of dihydropyridine boronic esters. *Angew. Chem.Int. Edit.*, **55**, 2205-2209 (2016). doi: 10.1002/anie.201510027 PMID: 26694785
26. Weyerstahl, P.; Schenk, A.; Marschall, H. Structure-odor correlation. Part XXI. Olfactory properties and convenient synthesis of furans and thiophenes related to rosefuran and perillene and their isomers. *Liebigs Ann.*, (10), 1849-1853 (1995).
27. Davis, C. J.; Hurst, T. E.; Jacob, A. M.; Moody, C. J. Microwave-mediated claisen rearrangement followed by phenol oxidation: A simple route to naturally occurring 1,4-benzoquinones. The first syntheses of verapliquinones A and B and panicein A. *J. Org. Chem*., **70**, 4414-4422 (2005). doi: 10.1021/jo050336x PMID: 15903320
28. Ray, R.; Jana, R. D.; Bhadra, M.; Maiti, D.; Lahiri, G. K. Efficient and simple approaches towards direct oxidative esterification of alcohols. *Chem. Eur. J.*, **20**, 15618-15624 (2014). doi: 10.1002/chem.201403786 PMID: 25284591
29. Gruenberg, M. F.; Goossen, L. J. Synthesis of arylacetates from benzylic alcohols and oxalate esters through decarboxylative coupling. *Chem. Eur. J.*, **19**, 7334-7337 (2013). doi: 10.1002/chem.201301033 PMID: 23625882
30. Vechorkin, O.; Proust, V.; Hu, X. Functional group tolerant Kumada-Corriu-Tamao Coupling of nonactivated alkyl halides with aryl and heteroaryl nucleophiles: catalysis by a nickel pincer complex permits the coupling of functionalized grignard reagents. *J. Amer. Chem. Soc.*, **131**, 9756-9766 (2009). doi: 10.1021/ja9027378 PMID: 19552426
31. Dakarapu, U. S.; Bokka, A.; Asgari, P.; Trog, G.; Hua, Y.; Nguyen, H. H.; Rahman, N.; Jeon, J. Lewis base activation of silyl acetals: iridium-catalyzed reductive Horner-Wadsworth-Emmons olefination. *Org. Lett.* **17**, 5792-5795 (2015). doi: 10.1021/acs.orglett.5b02901 PMID: 26566189
32. Kerr, W. J.; Mudd, R. J.; Brown, J. A. Iridium(I) N-heterocyclic carbene (NHC)/phosphine catalysts for mild and chemoselective hydrogenation processes. *Chem. Eur. J.*, **22**, 4738-4742 (2016). doi: 10.1002/chem.201504825 PMID: 26854284

33. Liao, J.; Zhang, Z.; Tang, X.; Wu, W.; Guo, W.; Jiang, H. Palladium-catalyzed desulfitative oxidative coupling between arenesulfinic acid salts and allylic alcohols: a strategy for the selective construction of β-aryl ketones and aldehydes. *J. Org. Chem.*, **80**, 8903-8909 (2015). doi: 10.1021/acs.joc.5b01463 PMID: 26282572
34. Das, S.; Li, Y.; Junge, K.; Beller, M. Synthesis of ethers from esters via Fe-catalyzed hydrosilylation. *Chem. Comm.* (Cambridge, United Kingdom), **48**, 10742-10744 (2012). doi: 10.1039/c2cc32142d PMID: 23024977

Table 1. Toxicity regression equations for concentration-effect of the compounds and their LC_{50} values (24 h)

No.	Regression equation [a)]	R^2	LC_{50} (μg/mL)	95% Confidence interval of LC_{50} (μg/mL)	Linear scope (μg/mL)
**1**	$y = 2.7087x + 1.1103$	0.9784	27.3	24.3–30.2	15–60
**2**	$y = 2.6042x + 0.7768$	0.9891	41.9	38.4–45.4	20–100
**3**	$y = 2.5692x + 1.0293$	0.9879	35.1	32.4–37.8	20–80
**4**	$y = 3.9526x – 1.6371$	0.9540	47.8	40.9–55.1	30–80
**5**	$y = 7.0889x – 9.1825$	0.9790	100.2	95.1–105.2	80–130
**6**	$y = 2.6090x – 0.3241$	0.9868	109.8	100.0–120.8	60–200
**10**	$y = 3.0936x – 1.1383$	0.9912	96.4	91.6–101.4	50–200
**11**	$y = 4.8292x – 3.7570$	0.9854	65.1	60.5–70.3	40–100
**24**	$y = 3.7818x – 3.5653$	0.9831	184.0	167.1–202.3	100–300
**27**	$y = 4.9056x – 4.5286$	0.9945	87.6	83.4–91.6	60–150
**32**	$y = 4.2484x – 3.4738$	0.9858	98.8	91.8–105.4	60–200
**37**	$y = 4.3019x – 3.6490$	0.9932	102.5	97.5–107.4	60–250
**38**	$y = 3.9089x – 2.8000$	0.9864	99.0	92.3–105.7	60–200
**39**	$y = 4.4982x – 3.8351$	0.9801	92.1	83.8–99.8	60–200
**40**	$y = 4.2928x – 2.3912$	0.9974	52.7	51.2–54.2	30–120
**41**	$y = 3.6334x – 0.7089$	0.9847	37.3	33.1–40.7	30–80
**42**	$y = 3.1031x – 0.6290$	0.9925	65.2	61.1–69.0	40–150
**43**	$y = 3.4494x – 1.9059$	0.9940	100.5	96.4–105.2	50–150
**46**	$y = 1.5387x + 2.1705$	0.9775	69.0	62.8–75.5	30–150
**47**	$y = 4.3679x – 3.4361$	0.9886	85.4	80.5–90.4	50–150
**48**	$y = 3.5616x – 2.1440$	0.9838	101.3	94.4–108.4	50–250
**51**	$y = 3.0350x – 0.4369$	0.9673	61.9	54.2–69.8	30–125
**52**	$y = 3.5768x – 1.2484$	0.9809	55.8	50.2–61.2	30–125
**53**	$y = 3.9432x – 1.4580$	0.9627	43.4	34.8–50.5	30–125
Ivermectin	$y = 1.3165x + 1.8491$	0.9804	247.4	197.9–310.2	50–1600

[a] y: Probability of average mortality; x: lg[C(μg/mL)].

Table 2. Toxicity regression equations for time-effect of the compounds at 4.5 μmol/mL and their LT_{50} values

No.	Regression equation [a]	R^2	LT_{50} (h)	95% CI [b]	RA [c]	Linear range (h)
**1**	$y = 9.0984x + 0.6113$	0.9848	3.0	3.0–3.1	2.9	2.33 – 3.83
**1** [d]	$y = 4.4261x + 4.5606$	0.9951	1.3	1.2–1.3	6.8	1.00 – 1.83
**2**	$y = 11.1101x – 0.5604$	0.9903	3.2	3.1–3.2	2.8	2.67 – 4.00
**2** [d]	$y = 5.5747x + 4.0191$	0.9672	1.5	1.4–1.6	5.9	1.33 – 2.50
**3**	$y = 19.6945x – 5.1898$	0.9635	3.3	3.2–3.4	2.7	2.83 – 4.00
**4**	y = 29.8386x – 17.1665	0.9840	5.5	5.5–5.6	1.6	5.17 – 6.17
**5**	$y = 14.3808x – 5.1383$	0.9805	5.1	5.0–5.1	1.7	4.50 – 6.33
**6**	$y = 17.8608x – 10.3128$	0.9873	7.2	7.2–7.2	1.2	6.50 – 8.33
**10**	$y = 27.3392x – 17.0537$	0.9664	6.4	6.3–6.5	1.4	6.00 – 7.17
**11**	$y = 16.5099x – 5.0836$	0.9801	4.1	4.0–4.1	2.2	3.33 – 5.00
**24**	$y = 4.0214x + 3.5413$	0.9809	2.3	2.2–2.4	3.9	1.33 – 3.00
**27**	--	--	<0.7		＞12.7	
**27** [d]	--	--	<0.7		＞12.7	
**27** [e]	$y = 8.5344x + 0.2645$	0.9947	3.6	3.6–3.6	2.5	2.83 – 4.83
**32**	$y = 23.2223x – 13.2799$	0.9750	6.1	6.1–6.2	1.5	5.33 – 7.33
**37**	$y = 18.4570x – 9.6374$	0.9896	6.2	6.2–6.3	1.5	5.33 – 7.50
**38**	$y = 17.5680x – 8.1745$	0.9738	5.6	5.5–5.7	1.6	5.33 – 7.17
**39**	$y = 17.2689x – 7.8647$	0.9904	5.6	5.5–5.6	1.6	4.83 – 6.67
**40**	$y = 14.1647x – 4.5156$	0.9818	4.7	4.6–4.8	1.9	4.00 – 5.67
**41**	$y = 14.0942x – 2.6710$	0.9837	3.5	3.4–3.6	2.5	3.00 – 4.08
**42**	$y = 11.9343x – 2.4564$	0.9843	4.2	4.2–4.3	2.1	3.83 – 5.50
**43**	$y = 5.1206x + 1.7615$	0.9752	4.3	4.1–4.6	2.1	2.00 – 5.67
**46**	$y = 6.3928x + 2.0329$	0.9924	2.9	2.8–3.0	3.1	1.67 – 5.17
**47**	$y = 5.8606x + 2.1464$	0.9767	3.1	2.9–3.2	2.9	2.00 – 5.67
**48**	$y = 13.931x - 7.8543$	0.9870	8.4	8.2–8.5	1.1	7.00 – 10.5
**51**	$y = 7.8552x + 2.2284$	0.9872	2.3	2.2–2.3	3.9	1.33 – 3.33
**52**	$y = 8.4775x – 2.3306$	0.9775	7.3	7.0–7.7	1.2	4.30 – 10.0
**53**	$y = 5.6738x + 0.5479$	0.9935	6.1	5.8–6.2	1.5	3.16 – 9.00
Ivermectin	$y = 5.5047x – 0.2254$	0.9840	8.9	8.8–9.0	1.0	8.00 – 17.00

[a] *y*: Probability of average mortality; *x*: lg[*T*(*h*)]. LC_{50} values were determined at post-treatment 24 h. LT_{50} values were obtained at the test concentration of 4.5 mM. [b] 95% Confidence interval. [c] Relative activity = LT_{50} of ivermectin / LT_{50} of the tested compound. [d] The test concentration of the compound is 2.0 mM. [e] The test concentration of the compound is 1.0 mM.
